# Noncovalent interaction–driven regio- and enantioselective hydroalkynylation of unactivated alkenes to access remote chiral nitriles

**DOI:** 10.1126/sciadv.ady4028

**Published:** 2025-11-28

**Authors:** Fanling Meng, Xian He, Rui He, Guodong Ju, Genping Huang, Chao Wang

**Affiliations:** ^1^ Tianjin Key Laboratory of Structure and Performance for Functional Molecules, College of Chemistry, Tianjin Normal University, Tianjin 300387, P. R. China.; ^2^ Department of Chemistry, School of Science, Tianjin University, Tianjin 300072, P. R. China.

## Abstract

Unactivated alkenes represent a challenging substrate for selective functionalization, particularly in achieving both regioselectivity and enantioselectivity. In this study, we present a strategy for the regio- and enantioselective hydroalkynylation of unactivated alkenes through the integration of noncovalent π⋯π interactions between the cyano group of the substrate and a chiral bis(oxazoline) ligand. The weak π⋯π interaction was supported by control experiments and computational studies. The method provides a reliable approach for synthesizing chiral nitriles with remote (β-, γ-, and δ-) stereocenters, which exhibit orthogonal reactivities and serve as valuable intermediates for further derivatization into otherwise difficult-to-access compounds. Our work provides a strategy for olefin functionalization and opens up avenues for the selective synthesis of chiral nitriles with remote stereocenters.

## Teaser

A mild olefin functionalization reaction uses weak molecular interactions to access remote chiral nitriles.

## INTRODUCTION

**Fig. 1.**
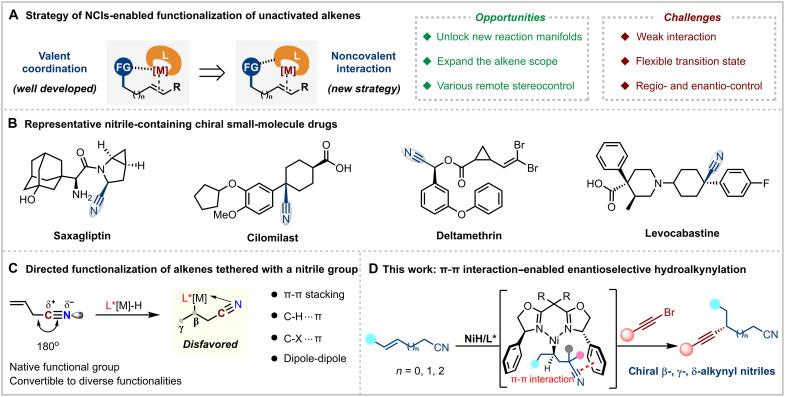
Strategy toward functionalization of unactivated alkenes. (**A**) Strategy of NCIs-enabled functionalization of unactivated alkenes. (**B**) Representative nitrile-containing functionalization of unactivated alkenes. (**C**) Directed functionalization of alkenes tethered with a nitrile group. (**D**) This work: π-π interaction–enabled enantioselective hydroalkynylation.

The functionalization of unactivated alkenes remains a cornerstone challenge in organic synthesis, with efforts over the past decade focused on improving reactivity and selectivity in these transformations (*1*–*7*). While substantial progress has been made through covalent substrate-metal coordination strategies, the scope of applicable alkenes remains limited by the availability of only a few coordinating groups, such as 8-aminoquinoline, picolinamide, amide, imine, and pyridyl derivatives (*8*–*17*). As a complementary approach, nondirected functionalization of unactivated alkenes, particularly in asymmetric transformations, remains underdeveloped, with only a limited number of examples reported to date (*18*–*22*). Noncovalent interactions (NCIs) are ubiquitous in nature (*23*–*27*) and have found widespread applications in crystal engineering, supramolecular chemistry, and organic synthesis, such as C─H functionalization (*28*–*36*) and the hydrogenation of unsaturated bonds (*37*–*42*). Given the increasing demand for asymmetric methodologies, we propose that integrating NCIs between the substrate and ligand with transition metal catalysis could provide a strategy for olefin functionalization (Fig. 1A). While density functional theory (DFT) calculations have revealed the presence of NCIs for controlling enantioselectivity (*43*–*46*), no reports have explored the stabilization of the transition state as a means to simultaneously control both regioselectivity and enantioselectivity. This strategy holds great promise for unlocking diverse reaction manifolds, expanding the alkene scope, and thereby broadening the product space. Moreover, remote stereocontrol with varying alkene distances can be achieved to construct positionally diverse remote stereocenters, although these situations typically require the use of different chiral ligands or catalysts (*47*–*49*). However, several challenges remain: First, careful ligand selection is critical for both molecular recognition and enantiodiscrimination. In addition, the inherently weak nature of NCIs results in increased flexibility in the transition states, complicating the stabilization of the alkyl-metal intermediate and making precise control over both regioselectivity and stereoselectivity more difficult.

Chiral nitriles are pivotal intermediates in the synthesis of a variety of functionalized compounds, including amines, aldehydes, ketones, carboxylic acids, and heterocycles, and are widely used in materials, pharmaceuticals, and agrochemicals (Fig. 1B) (*50*). Transition metal–catalyzed cyanation reactions, using toxic cyanide sources like metal cyanides or hydrogen cyanide, as well as less hazardous organic nitriles through C─CN bond activation, have emerged as effective synthetic strategies (*51*–*53*). However, these approaches predominantly yield α-branched nitriles (*54*–*60*), which limits their utility. In contrast, enantioselective functionalization of simple and readily accessible nitrile-containing alkenes provides an ideal route to access nitriles with β- or remote stereocenters, which would be highly beneficial for drug discovery. This method, however, remains underexplored because of several hurdles (Fig. 1C). First, the linear geometry of nitrile-containing alkenes (C─C≡N) makes it difficult to adopt the necessary bent conformation for facilitating directed olefin functionalization via a metallacyclic intermediate. Moreover, controlling both regioselectivity and enantioselectivity is challenging because of the lack of stabilization of adjacent polar groups, which often leads to undesired migratory products via β-H elimination/reinsertion (*61*–*65*), and impedes effective enantioselective discrimination.

Seeking an NCI-enabled strategy for olefin functionalization, we were inspired by the role of nitrile derivatives in drug design. Nitriles, recognized for their electron-deficient nature and asymmetric charge distribution across the C≡N bond, are frequently incorporated into pharmaceutical agents due to their ability to engage in a variety of NCIs with target proteins, such as dipole-dipole, π–π, and hydrogen bonding interactions (*66*, *67*). These interactions are fundamental to the biological activity of nitriles and have been extensively explored in the design of small molecules with enhanced selectivity and affinity (*68*, *69*). Motivated by this, we hypothesized that leveraging the interaction between the cyano group and a suitable ligand could enable enantioselective olefin functionalization. Herein, we report the development of a π⋯π interaction–enabled regio- and enantioselective hydroalkynylation of unactivated alkenes with readily accessible alkynyl bromides (*70*–*86*), facilitated by the recognition of the π electrons of the cyano group through the selection of a chiral bis(oxazoline) ligand (Fig. 1D). Control experiments confirmed that this interaction plays a critical role in simultaneously controlling both regio- and enantioselectivity, with computational studies further validating the π⋯π interaction. Moreover, this mild and reliable protocol enables the synthesis of alkynyl-functionalized β-, γ-, and δ-branched chiral nitriles with orthogonal synthetic reactivities, offering three versatile handles for further derivatization to access valuable intermediates.

## RESULTS

### Optimization of the reaction conditions

**Fig. 2.**
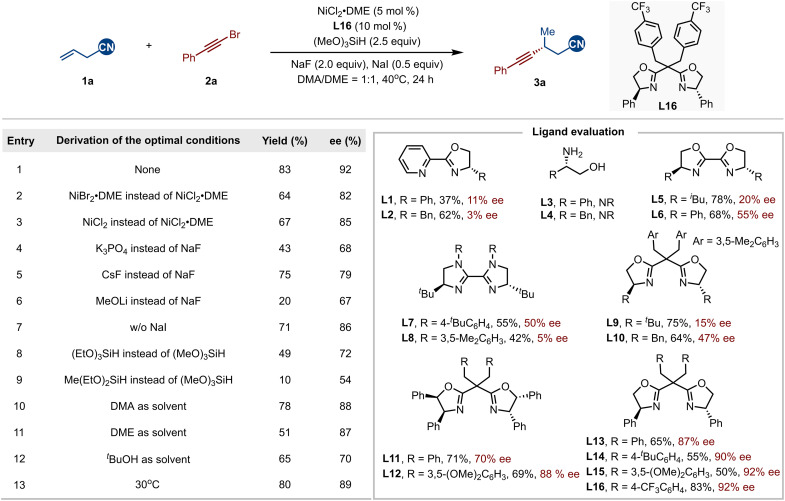
Variation of reaction parameters. Standard conditions: **1a** (0.2 mmol), **2a** (1.5 equiv), NiCl_2_•DME (5 mol%), **L16** (10 mol%), NaF (2.0 equiv), NaI (0.5 equiv), (MeO)_3_SiH (2.5 equiv), DMA/DME (1:1, 1 mL), 40°C, and 24 hours (h). Yields were determined by the isolated products. DMA, *N*,*N*-dimethylacetamide; DME, 1,2-dimethoxyethane; N.R., no reaction.

Our investigation into this NiH-catalyzed hydroalkynylation reaction began by selecting allylic nitrile **1a** and 1-(bromoethynyl)-benzene **2a** as model components to explore and optimize reaction parameters (Fig. 2). Considering that the NCIs between the ligand and substrate were anticipated to promote the reaction, we hypothesized that the careful selection of chiral ligand would be essential for modulating both the efficiency and enantioselectivity of the process. Initial ligand screening was carried out using NiCl_2_•DME as the catalyst, (MeO)_3_SiH as the hydride source, NaF as the base, and NaI as the additive in *N*,*N*-dimethylacetamide (DMA)/1,2-dimethoxyethane (DME). It was found that ligands played a crucial role in the success of this reaction. A series of privileged ligands, which may engage in C─H⋯π interactions, π-π stacking, or provide steric substituents, including pyridine-oxazoline (**L1** and **L2**), β-amino alcohol (**L3** and **L4**) (*87*, *88*), bioxazoline (**L5** and **L6**), and biimidazoline (**L7** and **L8**), all led to excellent selectivity toward the Markovnikov product **3a**, but the enantioselectivity remained low. We then screened bis(oxazoline) ligands (**L9** to **L16**), and to our satisfaction, the phenyl-substituted versions exhibited a notable improvement in enantiomeric excess (ee). Ultimately, **L16** delivered the best results, achieving an 83% isolated yield with 92% ee. Other nickel sources such as NiBr_2_•DME or NiCl_2_ resulted in lower yields and enantioselectivities (entries 2 and 3). After carefully examining various bases, we found that NaF was the most effective base for mediating the asymmetric transformation (entries 4 to 6). The addition of NaI improved both the yield and ee, likely by facilitating the regeneration of the NiH species (entry 7) (*86*). Other silanes, such as triethoxysilane and diethoxymethylsilane, led to inferior results (entries 8 and 9). Similarly, using a single solvent resulted in moderate yields and lower ee (entries 10 to 12). A slight decrease in both yield and ee was observed when the reaction was conducted at 30°C (entry 13).

### Substrate scope

**Fig. 3.**
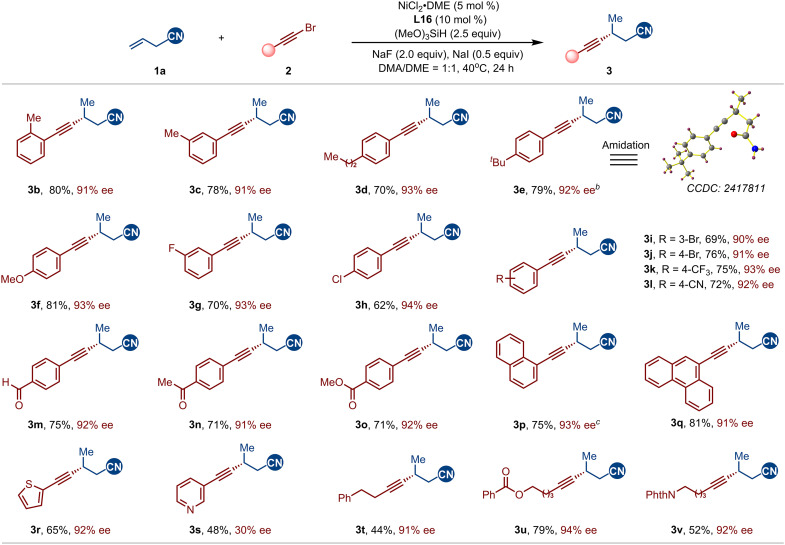
Scope of alkynyl bromides. Standard conditions: **1a** (0.2 mmol), **2** (1.5 equiv), NiCl_2_•DME (5 mol%), **L16** (10 mol %), NaF (2.0 equiv), NaI (0.5 equiv), (MeO)_3_SiH (2.5 equiv), DMA/DME (1:1, 1 ml), 40°C, and 24 hours. Isolated yield. *^b^*ee was determined by converting to the corresponding amide. *^c^*at 30°C. rr represents the ratio of the desired product to the sum of all other isomers, as determined by gas chromatography–mass spectrometry (GC-MS) analysis of the crude reaction mixture; all products were obtained with rr > 20:1.

With the optimized conditions in hand, we next explored the scope of alkynyl bromides in the NCI-enabled asymmetric hydroalkynylation with allylic nitriles (Fig. 3). Overall, a diverse range of aromatic and aliphatic alkynyl bromides effectively participated as coupling partners, yielding the desired chiral homopropargyl nitrile with high enantioselectivity (**3b** to **3t**). The reaction proved largely insensitive to the steric and electronic effects of aryl and heteroaryl alkynyl bromides, with substituents at various positions being well-tolerated. Moreover, the reaction demonstrated excellent functional group compatibility, accommodating alkynyl bromides bearing fluoride (**3g**), chloride (**3h**), bromide (**3i** and **3j**), trifluoromethyl (**3k**), cyano (**3l**), aldehyde (**3m**), ketone (**3n**), and ester (**3o**) groups, thereby showcasing its potential for further functionalization. Heteroaryl alkynyl bromides, including thiophene (**3r**) and pyridine (**3s**) derivatives, also proved compatible. The lower enantioselectivity observed for **3s** is likely due to coordination of the pyridine nitrogen to the nickel center, which may disrupt the NCIs between the substrate and the chiral ligand. In addition, alkyl alkynyl bromides were successfully incorporated, affording the products in good yield and high ee (**3t** to **3v**). The absolute configuration was determined by x-ray crystallographic analysis of the corresponding amidation product derived from the nitrile (**3e**), confirming the configuration as R.

**Fig. 4.**
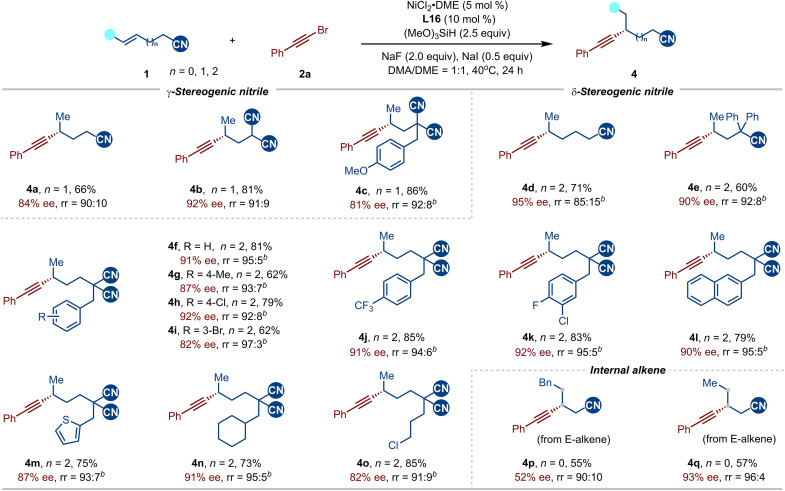
Scope of nitrile-containing alkenes. Reactions were conducted on a 0.2 mmol scale in DMA/DME (1:1, 1 ml), isolated yield; rr represents the ratio of the desired product to the sum of all other isomers, as determined by GC-MS analysis of the crude reaction mixture. *^b^*NiBr_2_•DME (10 mol %), **L16** (11 mol %), Na_2_CO_3_ (2 equiv).

Next, we explored the scope of nitrile-containing alkenes, beginning with homoallylic nitriles (Fig. 4), which led to the formation of chiral nitriles with a γ-stereocenter (**4a** to **4c**). Notably, 2-allylmalononitrile proved to be an excellent substrate, reacting efficiently to yield enantioenriched dinitriles in good yields (**4b** and **4c**). We next explored more challenging bishomoallylic nitriles, incorporating an additional carbon atom (**4d** to **4o**). To our surprise, after slight modifications to the standard conditions to improve regioselectivity and yield, these substrates reacted efficiently to yield δ-stereogenic nitriles. This result underscores the robustness and scalability of the NCIs-enabled asymmetric olefin functionalization, which can tolerate the formation of larger metallacycle intermediates while still delivering high yields and enantioselectivity. A wide range of substituents at the α-position of the malononitrile moiety were tested, leading to the corresponding products with high enantioselectivity. Moderate yield and ee were observed for sterically hindered internal alkene (**4p**), indicating that olefin migratory insertion process might be the rate- and enantio-determining step (*89*), which is consistent with the DFT calculations discussed later. In contrast, internal alkene bearing a less bulky methyl substituent afforded the desired product **4q** in 57% yield with 93% ee. Unfortunately, alkenes with a more distant nitrile group (*n* = 3) showed dramatically decreased regioselectivity, suggesting that NCIs weaken due to the increased distance from the ligand.

**Fig. 5.**
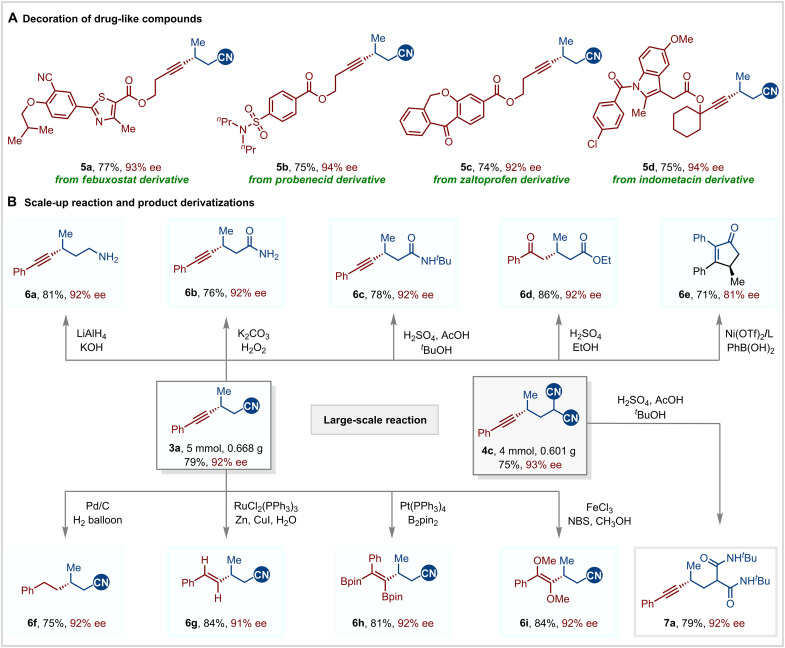
Synthetic applications. (**A**) Decoration of drug-like compounds. (**B**) Scale-up reaction and product derivatizations.

To highlight the practical applicability of our methodology in medicinal chemistry, we investigated its potential for late-stage modification of natural products and drug-like compounds (Fig. 5A). The alkynyl bromides derived from febuxostat, probenecid, zaltoprofen, and indometacin underwent smooth hydroalkynylation with **1a**, yielding the desired products (**5a** to **5d**). These reactions demonstrated high regioselectivity and enantioselectivity, showcasing the versatility of our approach in enabling the seamless integration of diverse functional groups into complex bioactive molecules while preserving stereochemical fidelity.

To further exemplify the synthetic utility of this enantioselective protocol, gram-scale reactions and subsequent product derivatizations were performed (Fig. 5B). Notably, the NCI-enabled asymmetric reaction can be easily scaled up to a 5.0 mmol scale, yielding **3a** in 79% yield while maintaining the enantioselectivity (92% ee). The cyano group in the product served as a versatile functional handle for subsequent derivatization. For instance, it could be reduced to the free primary amine **6a**, converted into the amide **6b** using basic hydrogen peroxide, transformed into an N-substituted amide **6c** through a Ritter reaction, turned into ester **6d** via a Pinner reaction, or cyclized to form cycloenone **6e** in the presence of phenyl boronic ester (*90*). Furthermore, the alkyne moiety can undergo a variety of transformations. Complete hydrogenation of the alkyne yielded β-alkyl–substituted chiral nitrile **6f**. Stereocontrolled semihydrogenation selectively afforded the corresponding *E*-olefin **6g**. Diboration and dimethoxylation produced the tetra-substituted alkenes **6h** and **6i**. In addition, dinitrile **4c** can be readily scaled up and efficiently converted into chiral malonamide **7a**.

### Mechanistic studies

**Fig. 6.**
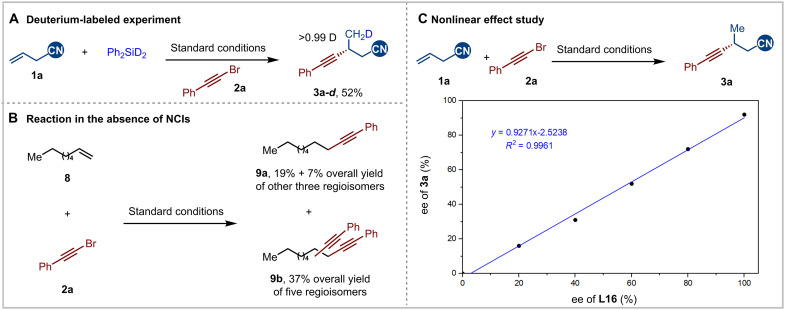
Mechanistic studies. (**A**) Deuterium labeling experiment. (**B**) Reaction in the absence of NCIs. (**C**) Nonlinear effect study.

To gain insight into the mechanism of this asymmetric hydroalkynylation of unactivated alkenes, we conducted a series of preliminary mechanistic experiments (Fig. 6). Deuterium-labeling experiment was first conducted using Ph_2_SiD_2_ instead (Fig. 6A), and the deuterium atom was incorporated (>99% D) at the terminal position. This result suggested that the hydrogen source exclusively comes from hydrosilane, and the hydrometallation step is stereospecific (Markovnikov addition) and irreversible. Unactivated alkenes in the absence of cyano group were tested to probe its role (Fig. 6B). Octene reacted with very poor regioselectivity, giving a mixture of four hydroalkynylation regioisomers and five dialkynylation regioisomers, which could not be identified (see the Supplementary Materials for details). In addition, we investigated the relationship between product enantioselectivity and the ee of the ligand (Fig. 6C), which revealed a direct linear relationship. This suggests that the active nickel catalyst was likely coordinated to a single ligand during the catalytic process.

**Fig. 7.**
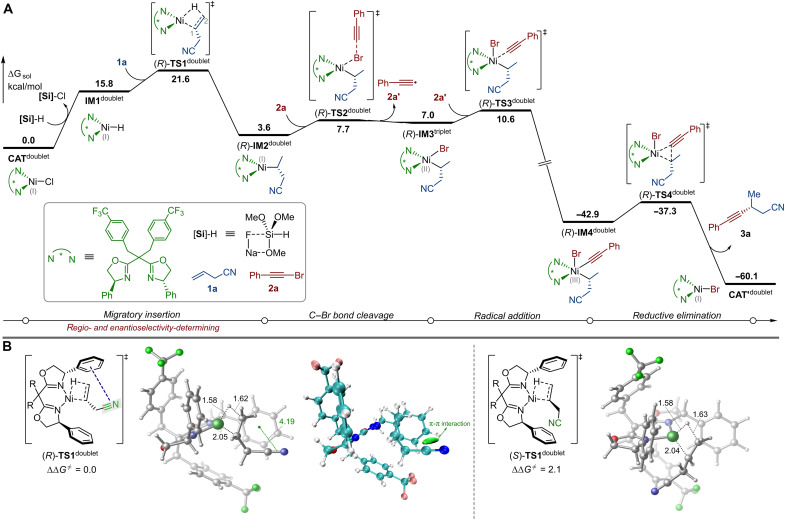
DFT Calculations. (**A**) Calculated energy profile of the most favorable reaction pathway leading to product **3a**. (**B**) Optimized geometries and NCI analysis of (*R*)-**TS1**^doublet^ and (*S*)-**TS1**^doublet^. Energies and bond distances are given in kcal/mol and Å, respectively. The structures of the transition states are marked with ‡. ∆∆G^≠^ refers to the energy difference between (*R*)-**TS1**^doublet^ and (*S*)-**TS1**^doublet^.

DFT calculations were conducted to investigate the detailed reaction mechanism and the origins of selectivity. The experimentally used allylic nitrile **1a** and 1-(bromoethynyl)-benzene **2a** were selected as model substrates, with **L16** as the chiral ligand. The energy profile of the most favorable reaction pathway leading to product **3a** was calculated and is depicted in Fig. 7A. The reaction is initiated with the formation of the Ni(I)-H species **IM1**^doublet^ from the commonly proposed active catalyst species **CAT**^doublet^ and (MeO)_3_SiH (*15*, *91*), which was calculated to be endergonic by 15.8 kcal/mol. Notably, the possibility of a reaction pathway initiated by C─Br bond cleavage of **2a** was also examined but was found to be kinetically less favorable (see fig. S8). A complete overview of the proposed catalytic cycle based on the DFT results is provided in fig. S13.

The subsequent 1,2-insertion of the C═C double bond of **1a** into the Ni─H bond occurs via transition state (*R*)-**TS1**^doublet^, leading to the formation of the Ni(I)-alkyl intermediate (*R*)-**IM2**^doublet^. Then, the C─Br bond cleavage was found to proceed via the halogen-atom transfer transition state (*R*)-**TS2**^doublet^, resulting in the formation of the Ni(II) intermediate (*R*)-**IM3**^triplet^ and an alkynyl radical **2a′**. The ensuing recombination of **2a′** with the Ni center occurs through transition state (*R*)-**TS3**^doublet^, leading to the formation of the Ni(III) intermediate (*R*)-**IM4**^doublet^, from which the final product **3a** is obtained via C─C reductive elimination through transition state (*R*)-**TS4**^doublet^.

The computations show that the migratory insertion is the rate- and selectivity-determining step of the reaction. Scrutiny of the optimized geometries and the NCI analysis reveal the presence of the π⋯π interaction the cyano group of **1a** and the phenyl group of **L16** in (*R*)-**TS1**^doublet^ (Fig. 7B), which turns out to be a key factor that contributes to the selectivity. Transition state (*S*)-**TS1**^doublet^, in which no such interaction was observed, was calculated to be higher in energy than (*R*)-**TS1**^doublet^ by 2.1 kcal/mol, aligning well with the experimentally observed enantioselectivity. The 2,1-insertion of the C═C double bond into the Ni─H bond was also evaluated, which was calculated to be higher in energy than (*R*)-**TS1**^doublet^ by 3.1 kcal/mol, in accordance with the experimentally observed regioselectivity (see fig. S9). The preference of the 1,2-insertion via (*R*)-**TS1**^doublet^ over the 2,1-insertion is mainly attributed to the combination of the π⋯π interaction and the electron-withdrawing character of the cyano group. The latter can stabilize the developing negative charge at the C1 atom, thereby facilitating the 1,2-insertion.

## DISCUSSION

In conclusion, we have developed an NCIs-enabled strategy for the regio- and enantioselective hydroalkynylation of unactivated alkenes using the π⋯π interaction between the cyano group of the substrate and a chiral bis(oxazoline) ligand. This approach provides a reliable method for the synthesis of chiral nitriles with remote stereocenters, which exhibit orthogonal reactivities, making them valuable intermediates for further functionalization into complex compounds. The methodology is applicable to a wide range of substrates, demonstrating excellent functional group compatibility and scalability. Moreover, this work highlights the potential of NCIs in catalysis, offering possibilities for controlling both regioselectivity and enantioselectivity in alkene functionalization. Our findings open up avenues for the design of selective catalytic processes in organic synthesis, with implications in materials science, medicinal chemistry, and beyond. More applications of this strategy for olefin functionalization are ongoing in our laboratory.

## MATERIALS AND METHODS

### Procedure for the enantioselective hydroalkynylation of unactivated alkenes

In an argon-filled glovebox, NiCl_2_•DME (2.2 mg, 0.01 mmol, and 5.0 mol %), ligand (12.9 mg, 0.02 mmol, and 10 mol %), relevant alkene substrate (0.2 mmol and 1.0 equiv), NaF (16.8 mg, 0.6 mmol, and 3.0 equiv), NaI (15.0 mg 0.1 mmol, and 0.5 equiv), DMA/DME (1:1, 1.0 ml) were added to a 4-ml reaction tube. Then, relevant acetylene bromide (0.3 mmol and 1.5 equiv), (MeO)_3_SiH (64 μl, 0.5 mmol, and 2.5 equiv) were added to the mixture. The reaction mixture was stirred at 40°C for 24 hours. Upon completion, the solvent was removed under reduced pressure, and the crude residue was purified by column chromatography on silica gel using a mixture of ethyl acetate and petroleum ether as eluent. More experimental details and characterization are available in the Supplementary Materials.

## Supplementary Materials

### This PDF file includes:

Supplementary Text Figs. S1 to S13 Tables S1 to S12 Spectral Data References
