# Supplementary material for: Noncovalent interaction–driven regio- and enantioselective hydroalkynylation of unactivated alkenes to access remote chiral nitriles
Source: Sci Adv. 2025 Nov 28;11(48):eady4028. doi: 10.1126/sciadv.ady4028 (PMC12662212; doi:10.1126/sciadv.ady4028)
Supplement: Supplementary file 1 — Supplementary Text Figs. S1 to S13 Tables S1 to S12 Spectral Data References [file sciadv.ady4028_sm.pdf]

Science Advances
AAAS

Supplementary Materials for

Noncovalent interaction–driven regio- and enantioselective hydroalkynylation of unactivated alkenes to access remote chiral nitriles

Fanling Meng *et al.*

Corresponding author: Guodong Ju, gdju@tjnu.edu.cn; Genping Huang, gphuang@tju.edu.cn; Chao Wang, chwang@tjnu.edu.cn

Sci. Adv. **11**, eady4028 (2025)
DOI: 10.1126/sciadv.ady4028

This PDF file includes:

Supplementary Text
Figs. S1 to S13
Tables S1 to S12
Spectral Data
References

1. General information

All the manipulations were performed in an argon-filled glovebox, unless mentioned otherwise. Anhydrous solvent was purchased from commercial sources and transferred under argon atmosphere. Alkene substrates and alkynyl bromide were prepared according to previously reported procedures. $NiCl_2$•DME (CAS 29046-78-4) was purchased from Leyan. com. Allyl cyanide (CAS 109-75-1) was purchased from Meryer (Shanghai)Biochemical Technology Co., Ltd. and stored under nitrogen in glove box. Other reagents were purchased from Adamas-beta®, Energy Chemicals, Bidepharm and used directly without further purification unless otherwise specified. ^{1}H NMR, ^{13}C NMR spectra were recorded using Bruker 400 MHz NMR and 600 MHz NMR spectrometer. ^{1}H NMR and ^{13}C NMR spectra were referenced to resonances of the residual protons in the deuterated solvents. Multiplicities are recorded as: s = singlet, d = doublet, t = triplet, h = hextet and m = multiplet. GC-MS analysis was performed on Shimadzu GC-2010 gas chromatography coupled to a Shimadzu QP2010 mass selective detector. Analytical HPLC/MS was performed with an Agilent 6520 Series HPLC; X-Ray Diffraction(XRD) was carried out on an Bruker D8 Venture Metaljet Photon II at Shiyanjia lab. Reactions carried out at elevated temperature were heated using oil bath.

Medium-sized screw-cap test tubes (4 mL) were used for all 0.20 mmol scale reactions:15 x 45 mm tubes.

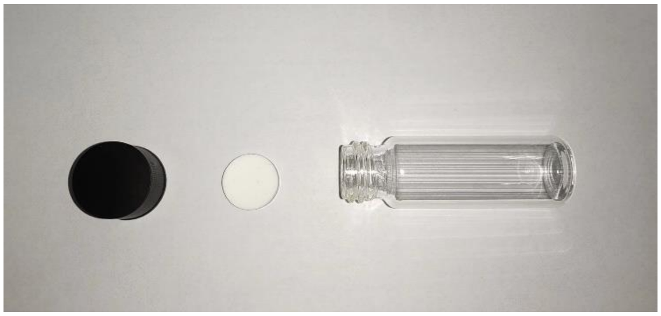

Fig. S1: Reaction tube (4 mL)

100 mL round bottom pressure vessel (Synthware) was used for 5.0 mmol-scale reaction.

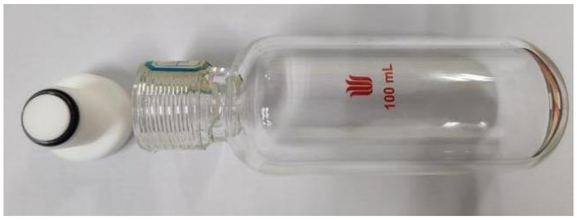

Fig. S2. Reaction tube (100 mL)

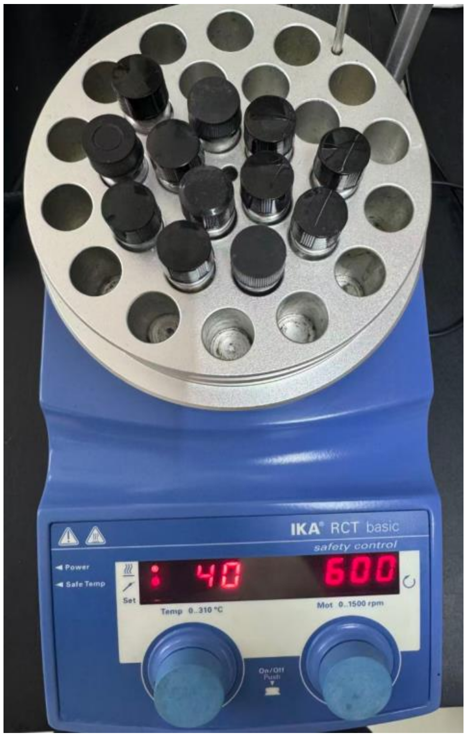

Fig. S3. Reaction on process

1. Reaction condition optimizations

Table S1. Evaluation of ligands[a]

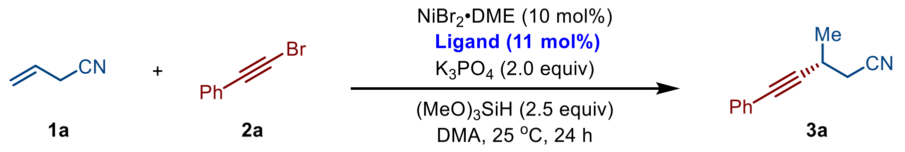

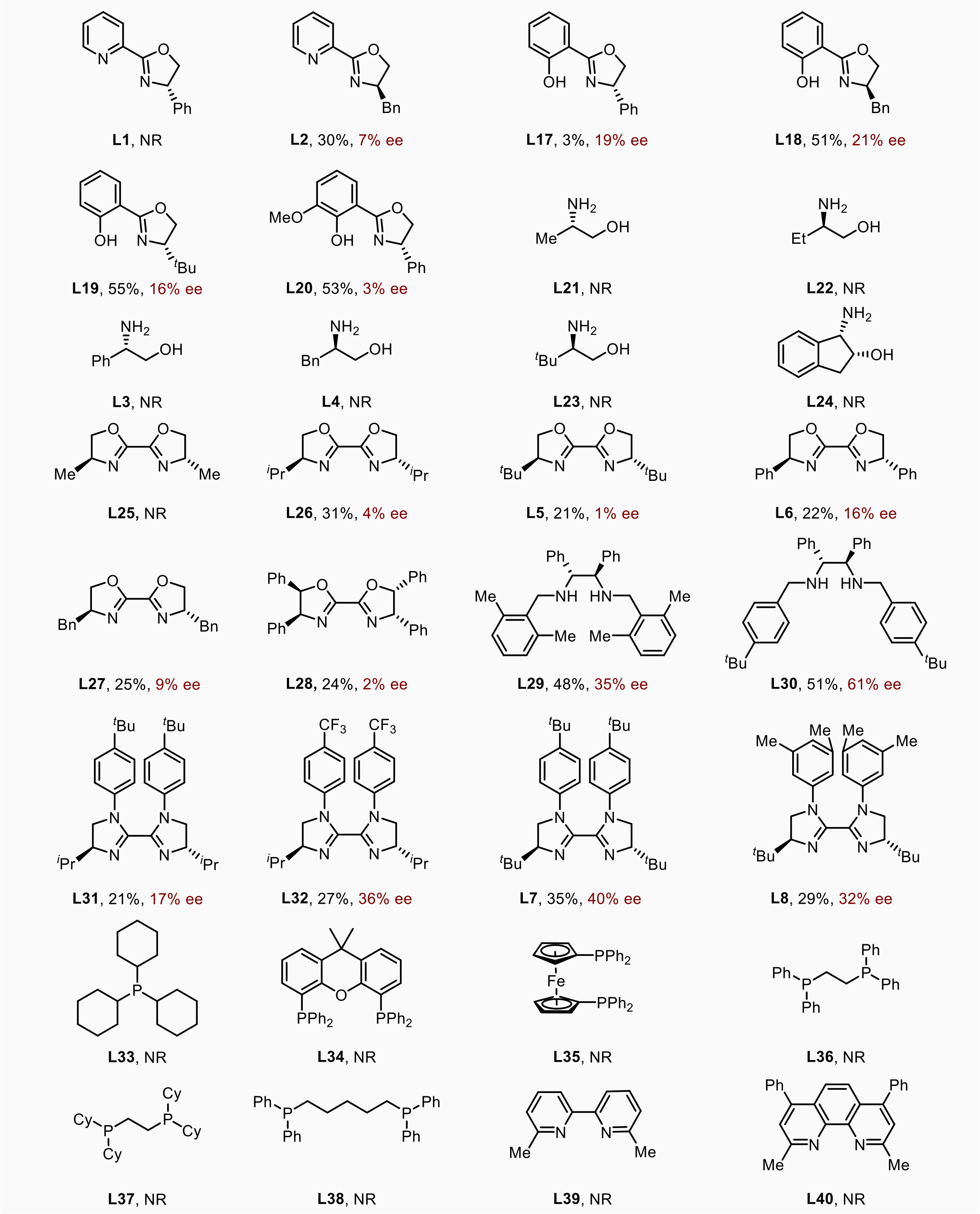

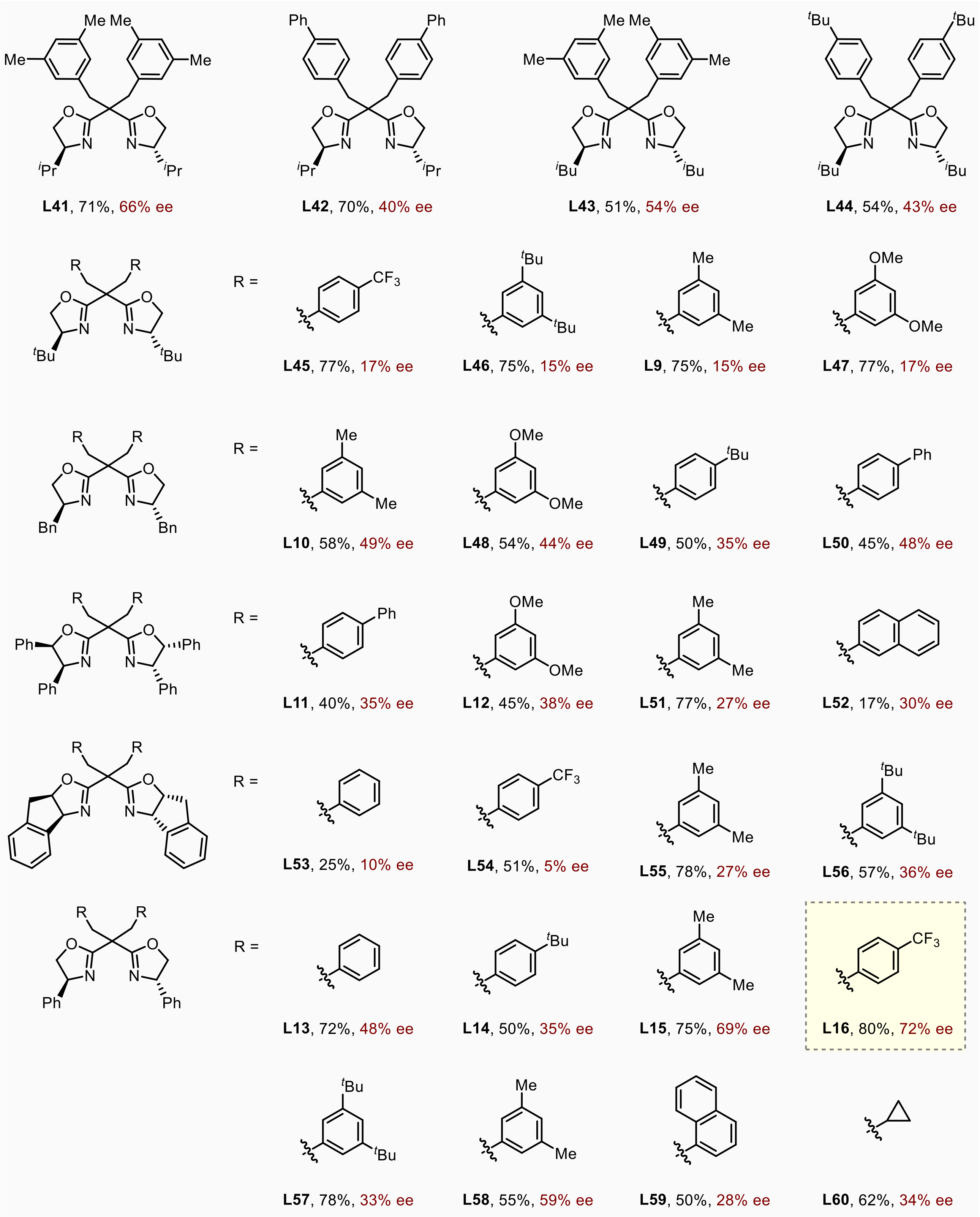

[a]Standard conditions: **1a** (0.2 mmol, 1.0 equiv), **2a** (1.5 equiv), $NiBr_2$·DME (10 mol%), Ligand (11 mol%), K_3PO_4 (2.0 equiv), $(MeO)_3SiH$ (2.5 equiv) and DMA (1.0 mL), stirred at 25 °C for 24 h. Determined by GC or HPLC using chiral columns. DMA = *N*,*N*-Dimethylacetamide,

Table S2. Evaluation of base[a]

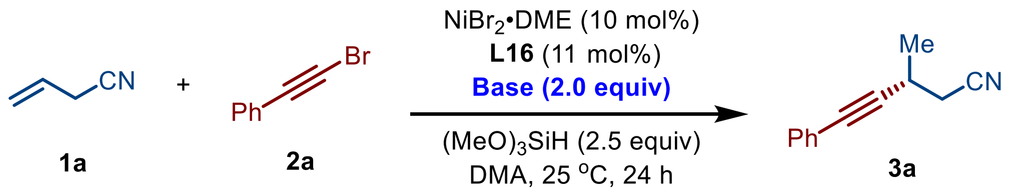

Entry	Base	Yield (%)	ee (%)
1	none	N.R.	/
2	Na_2CO_3	78	57
3	$NaHCO_3$	77	52
4	K_2CO_3	81	68
5	$KHCO_3$	80	51
6	^{t}BuOLi	N.R.	/
7	^{t}BuONa	N.R.	/
8	^{t}BuOK	N.R.	/
9	LiOH	N.R.	/
10	NaOH	N.R.	/
11	$Mg(OEt)_2$	N.R.	/
12	NaF	80	76
13	CsF	79	54
14	KOAc	N.R.	/

[a]Standard conditions: **1a** (0.2 mmol, 1.0 equiv), **2a** (1.5 equiv), $NiBr_2$·DME (10 mol%), **L16** (11 mol%), Base (2.0 equiv), $(MeO)_3SiH$ (2.5 equiv) and DMA (1.0 mL), stirred at 25 °C for 24 h. Determined by GC or HPLC using chiral columns. DMA = *N*,*N*-Dimethylacetamide, N.R. = No reaction.

Table S3. Screening of solvents[a]

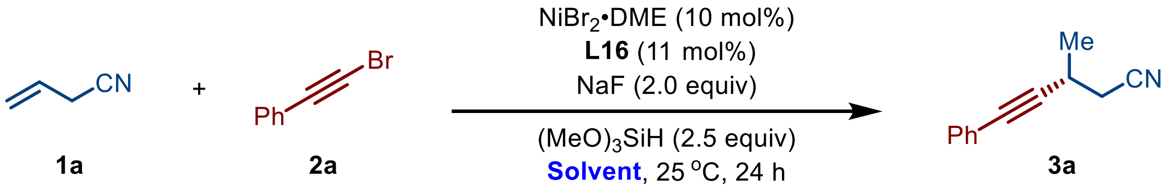

Entry	Solvent	Yield (%)	ee (%)
1	THF	25	65
2	MeOH	40	74
3	^{t}BuOH	33	71
4	CH_3CN	N.R	/
5	Toluene	45	/
6	NMP	39	72
7	DCM	42	71
8	Et_2O	N.R.	/
9	DMA	80	76
10	DME	75	71
11	DMF	74	72
12	DMSO	N.R.	/
13	1,4-Dioxane	44	62

[a]Standard conditions: **1a** (0.2 mmol, 1.0 equiv), **2a** (1.5 equiv), $NiBr_2$·DME (10 mol%), **L16** (11 mol%), NaF (2.0 equiv), $(MeO)_3SiH$ (2.5 equiv) and solvent (1.0 mL), stirred at 25 °C for 24 h. Determined by GC or HPLC using chiral columns. NMP = N - Methyl - 2 – pyrrolidone; DME = 1,2-Dimethoxyethane; DMF = N,N-Dimethylformamide; DMA = *N*,*N*-Dimethylacetamide; DMSO = Dimethyl sulfoxide; N.R. = No reaction.

Table S4. Screening of silanes[a]

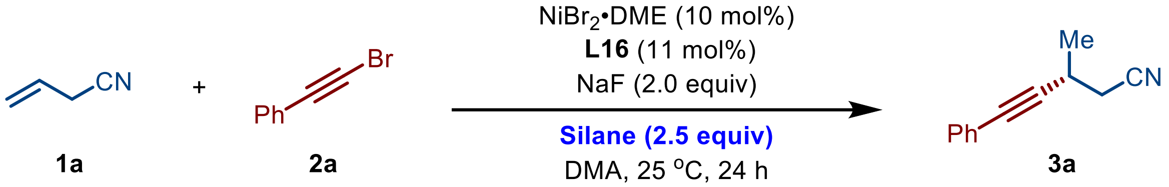

Entry	Silane	Yield (%)	ee (%)
1	$Me(EtO)_2SiH$	51	64
2	$Me(MeO)_2SiH$	65	56
3	$(Et)_3SiH$	N.R.	/
4	$Et(Me)_2SiH$	20	70
5	$(MeO)_3SiH$	80	76
6	$(EtO)_3SiH$	55	70
7	$Ph(Me)_2SiH$	19	71
8	$MePhSiH_2$	11	75
9	Ph_2SiH_2	68	75
10	$PhSiH_3$	66	74

[a]Standard conditions: **1a** (0.2 mmol, 1.0 equiv), **2a** (1.5 equiv), $NiBr_2$·DME (10 mol%), **L16** (11 mol%), NaF (2.0 equiv), Silane (2.5 equiv) and DMA (1.0 mL), stirred at 25 °C for 24 h. Determined by GC or HPLC using chiral columns. DMA = *N*,*N*-Dimethylacetamide, N.R. = No reaction.

Table S5. Screening of catalysts[a]

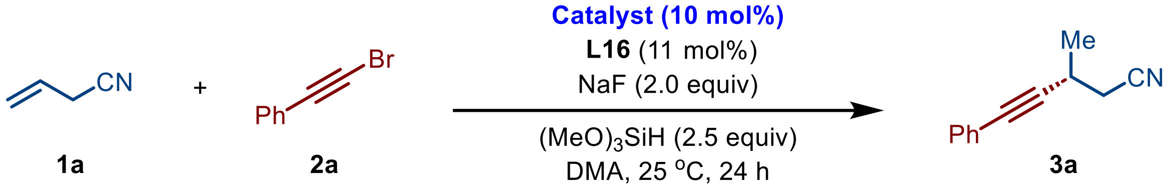

Entry	Catalyst	Yield (%)	ee (%)
1	$NiCl_2$	45	75
2	$NiBr_2$	69	75
3	NiI_2	50	73
4	$NiBr_2$•DME	80	76
5	$NiCl_2$•DME	81	79
6	$NiCl_2$•$6H_2O$	80	69
7	$Ni(NO_3)_2$•$6H_2O$	79	47

[a]Standard conditions: **1a** (0.2 mmol, 1.0 equiv), **2a** (1.5 equiv), Catalyst (10 mol%), **L16** (11 mol%), NaF (2.0 equiv), $(MeO)_3SiH$ (2.5 equiv) and DMA (1.0 mL), stirred at 25 °C for 24 h. Determined by GC or HPLC using chiral columns. DMA = *N*,*N*-Dimethylacetamide.

Table S6. Screening of additives[a]

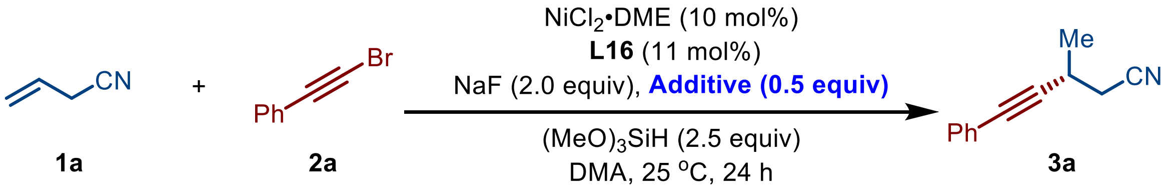

Entry	Additive	Yield (%)	ee (%)
1	none	80	76
2	LiI	81	83
3	NaI	81	85
4	KI	80	84
5	Na_2CO_3	40	79
6	K_2CO_3	61	71
7	$NaHCO_3$	63	78
8	$KHCO_3$	72	70
9	^{t}BuONa	N.R.	/
10	KF	79	77
11	LiOH	80	79
12	NaOH	81	78

[a]Standard conditions: **1a** (0.2 mmol, 1.0 equiv), **2a** (1.5 equiv), $NiCl_2$·DME (10 mol%), **L16** (11 mol%), NaF (2.0 equiv), Additive (0.5 equiv), $(MeO)_3SiH$ (2.5 equiv) and DMA (1.0 mL), stirred at 25 °C for 24 h. Determined by GC or HPLC using chiral columns. DMA = *N*,*N*-Dimethylacetamide.

Table S7. Screening of mixed solvents[a]

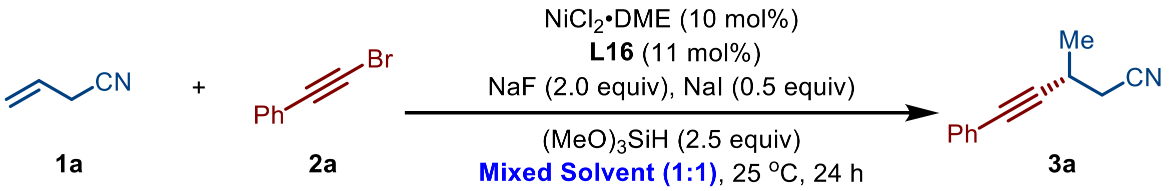

Entry	Mixed Solvent	Yield (%)	ee (%)
1	DMA/DMF	78	77
2	DMA/DME	83	89
3	DMA/DMSO	40	21
4	DMA/NMP	71	65
5	DMA/DCM	78	41
6	DMA/DCE	76	66
7	DMA/Et_2O	79	69
8	DMA/EtOH	66	43
9	DMA/$(CH_2OH)_2$	64	67
10	DMA/Toluene	72	79
11	DMA/1,4-Dioxane	74	88
12	DMA/THF	77	84

[a]Standard conditions: **1a** (0.2 mmol, 1.0 equiv), **2a** (1.5 equiv), $NiCl_2$·DME (10 mol%), **L16** (11 mol%), NaF (2.0 equiv), NaI (0.5 equiv), $(MeO)_3SiH$ (2.5 equiv) and mixed solvent (1:1, 1.0 mL), stirred at 25 °C for 24 h. Determined by GC or HPLC using chiral columns. DMA = *N*,*N*-Dimethylacetamide, DME = 1,2-Dimethoxyethane.

Table S8. Screening of temperature[a]

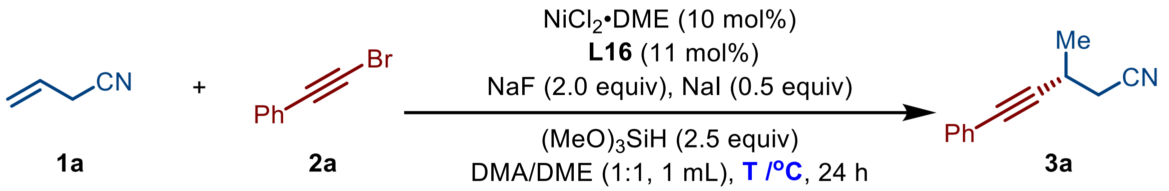

Entry	Temperature/°C	Yield (%)	ee (%)
1	30	80	89
2	40	83	91
3	50	82	89
4	60	78	85

Standard conditions: **1a** (0.2 mmol, 1.0 equiv), **2a** (1.5 equiv), $NiCl_2$·DME (10 mol%), **L16** (11 mol%), NaF (2.0 equiv), NaI (0.5 equiv), $(MeO)_3SiH$ (2.5 equiv) and DMA/DME (1:1, 1.0 mL), stirred at corresponding temperature for 24 h. Determined by GC or HPLC using chiral columns.

Table S9. Examination of the catalyst loading and Ni/L ratio[a]

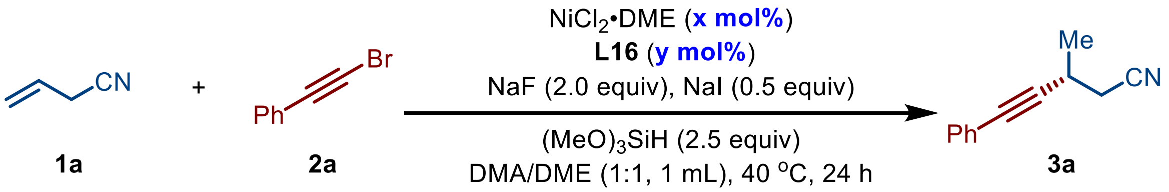

Entry	Catalyst (x mol%)	Ligand (y mol%)	Yield (%)	ee (%)
1	10 mol%	12 mol%	81	90
2	10 mol%	15 mol%	80	90
3	10 mol%	20 mol%	79	71
4	5 mol%	6 mol%	77	90
5	5 mol%	10 mol%	83	92
6	5 mol%	12 mol%	83	91

[a]Standard conditions: **1a** (0.2 mmol, 1.0 equiv), **2a** (1.5 equiv), $NiCl_2$·DME (x mol%), **L16** (y mol%), NaF (2.0

equiv), NaI (0.5 equiv), $(MeO)_3SiH$ (2.5 equiv) and DMA/DME (1:1, 1.0 mL), stirred at 40 °C for 24 h. Determined by GC or HPLC using chiral columns. DMA = *N*,*N*-Dimethylacetamide, DME = 1,2-Dimethoxyethane.

Table S10. Examination of the amounts of alkynyl bromides and NaI

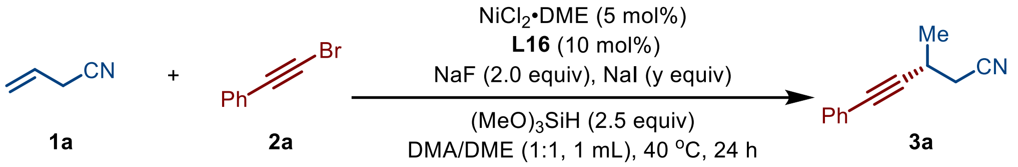

Entry	2a (x equiv)	NaI (y equiv)	Yield (%)	ee (%)
1	1.5 equiv	0.5 equiv	83	93
2	1.0 equiv	0.5 equiv	81	92
3	2.0 equiv	0.5 equiv	80	92
4	3.0 equiv	0.5 equiv	79	90
5	1.5 equiv	0.2 equiv	83	90
6	1.5 equiv	1.0 equiv	80	92
7	1.5 equiv	1.5 equiv	70	88

Standard conditions: **1a** (0.2 mmol, 1.0 equiv), **2a** (1.5 equiv), $NiCl_2$·DME (10 mol%), **L16** (11 mol%), NaF (2.0 equiv), NaI (0.5 equiv), $(MeO)_3SiH$ (2.5 equiv) and DMA/DME (1:1, 1.0 mL), stirred at corresponding temperature for 24 h. Determined by GC or HPLC using chiral columns.

2. Synthesis of substrates

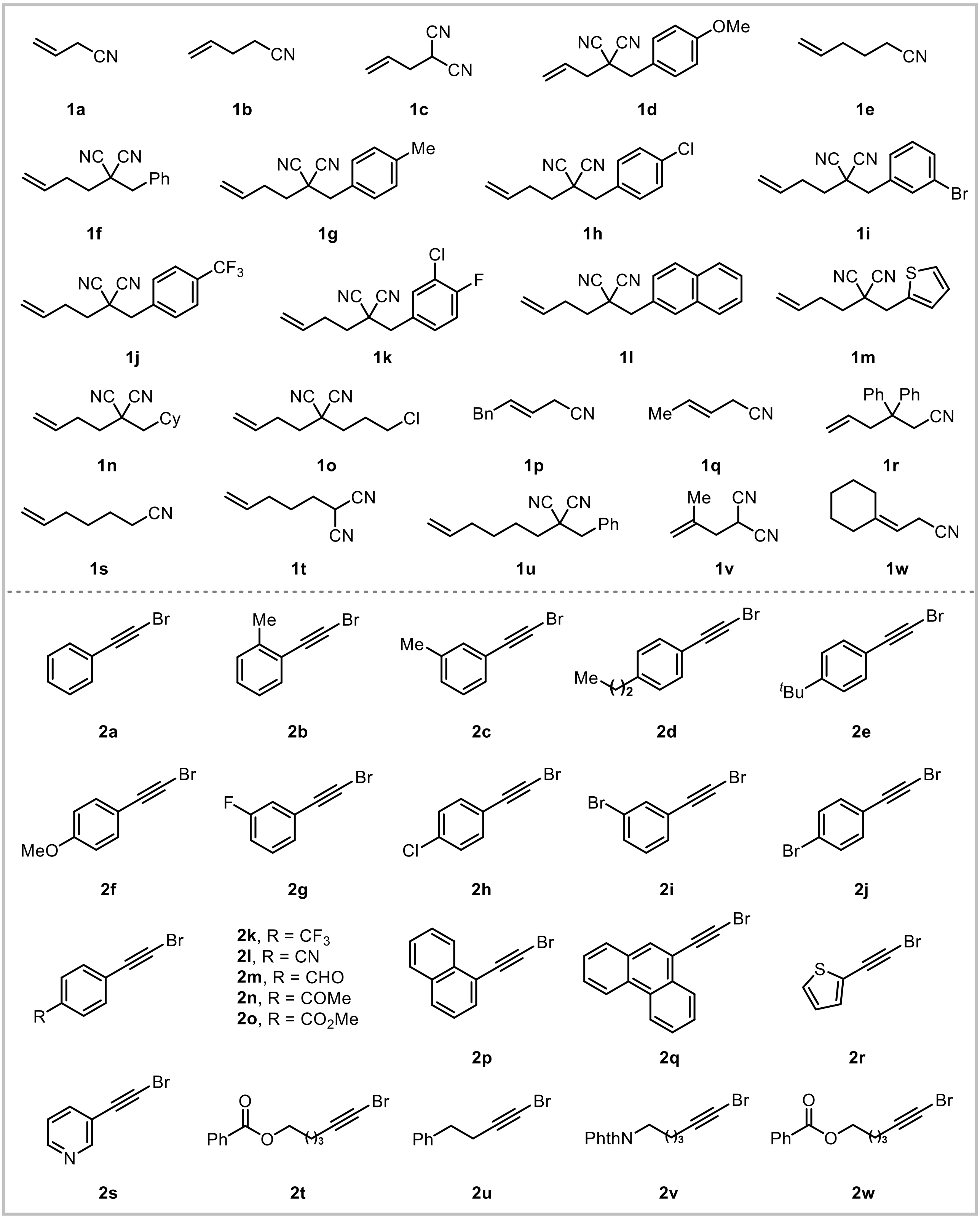

Fig. S4. List of alkenes and alkynes used in this study

The substrates **1a**, **1b**, **1e, 1q** and **1s** were purchased from Meryer. Other substrates were prepared according to reference *(17, 90, 92-95)*

Synthesis of 2-allylmalononitrile (1c)

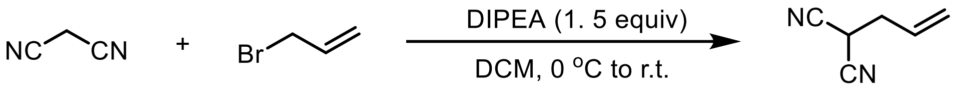

To a suspension of malononitrile (10.0 mmol, 1.0 equiv) in dicholoroethane (20.0 mL) under nitrogen atmosphere cooled to 0 °C, DIPEA (12.0 mmol, 1.2 equiv) and the appropriate 3-bromoprop-1-ene (12.0 mmol, 1.2 equiv) were added sequentially dropwise and the reaction mixture was left for stirring at room temperature for 6 h. After completion of the reaction (monitored by TLC) the reaction mixture was quenched with adding water (5.0 mL). After separating the organic layer, the aqueous layer was extracted with EtOAc (5 × 3.0 mL). The combined organic extract was washed with brine, dried over anhydrous sodium sulphate and the solvent was removed under reduced pressure. The crude concentrate was purified by flash column chromatography on silica gel (200-300 mesh) using EtOAc in petroleum ether (5-10%) as the eluent to afford the pure products as thick colorless liquids *(92)*.

General procedures for the preparation of dinitriles (1f-1n)

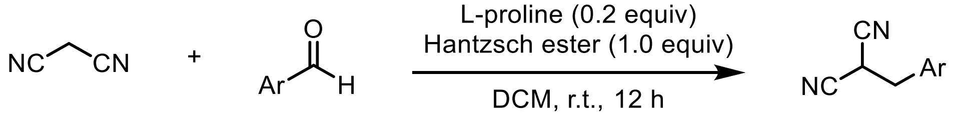

To a solution of benzaldehyde derivatives (30 mmol, 3.0 equiv) in DCM (30 mL) were added malononitrile (661 mg, 10.0 mmol, 1.0 equiv), L-proline (231 mg, 2.0 mmol, 0.2 equiv), and Hantzsch ester (2.53 g, 10.0 mmol, 1.0 equiv) sequentially at room temperature. The mixture was stirred at the room temperature for 12 h. The solvent was removed under reduced pressure to give a crude mixture. The crude mixture was purified by flash column chromatography to give the desired product *(90)*.

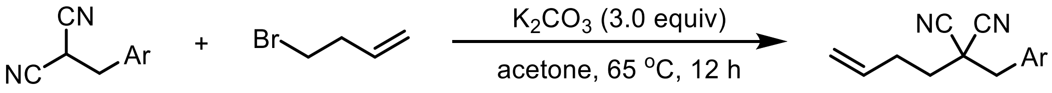

To a dry round bottom flask equipped with a magnetic stirring bar were added the Bromo-1-butene (6 mmol, 1.2 equiv), 2-benzylmalononitrile derivatives (5 mmol, 1.0 equiv), K_2CO_3 (15 mmol, 3.0 equiv) and 30 mL of acetone at room temperature. The flask was sealed and the mixture was stirred at the 65 0 °C for 12 h. Then the mixture was cooled to room temperature, filtered through a celite pad and washed with EA (3×10 mL). The solvents were removed under reduced pressure to give a crude mixture. The crude mixture was purified by flash column chromatography to give the desired product **1f-1n** *(93)*.

2-(but-3-en-1-yl)-2-(3-chloropropyl)malononitrile (1o)

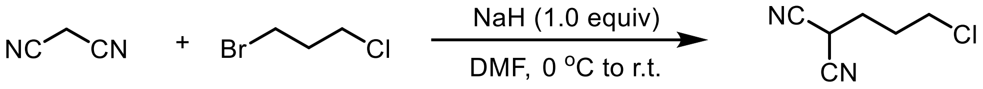

To a dry round bottom flask was added sodium hydride (10 mmol, 1.0 equiv), malononitrile (20 mmol, 2.0 equiv), and DMF (30 mL) at 0 °C. The reaction mixture was stirred at 0 °C for 2 h under N_2 and then 1-bromo-3-chloropropane (10 mmol, 1.0 equiv) was added dropwise. The reaction mixture was stirred at rt overnight. After the reaction was complete, the mixture was quenched with sat. NH_4Cl (20 mL). The reaction was extracted with ethyl acetate, and the combined organic layer was washed with brine, dried over $MgSO_4$, filtered, concentrated in vacuum, and purified by flash column chromatography on silica gel (ethyl acetate/ petroleum ether) to give 2-(3-chloropropyl)malononitrile.

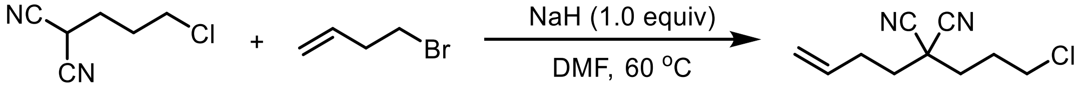

To a dry round bottom flask was sequentially added sodium hydride (6 mmol, 1.2 equiv), DMF (20 mL), and then 2-(3-chloropropyl)malononitrile (5 mmol, 1.0 equiv) at 0 °C. The reaction mixture was stirred at rt for 1 h under N_2 and then 4-bromo-1-butene (6 mmol, 1.2 equiv) was added dropwise at rt. The reaction mixture was stirred at 0 °C overnight and quenched with sat. NH_4Cl (20 mL). The reaction was extracted with ethyl acetate, concentrated in vacuum, and purified by flash column chromatography on silica gel (ethyl acetate/ petroleum ether) to give 2-(but-3-en-1-yl)-2-(3-chloropropyl)malononitrile *(94)*.

(*E*)-5-phenylpent-3-enenitrile (1p)

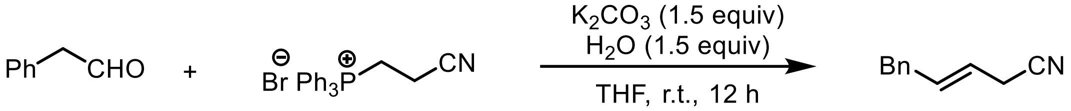

To a Schlenk flask equipped with a magnetic stirring bar were added phenylacetaldehyde (2.4 g, 20.0 mmol, 1.0 equiv), phosphonium salt (9.5 g, 24.0 mmol, 1.2 equiv), K_2CO_3 (4.2 g, 30 mmol, 1.5 equiv), H_2O (360 μL, 1.0 equiv) and 60 mL of THF at room temperature. The flask was sealed and the mixture was stirred at room temperature for 12 h. Then the mixture was cooled to room temperature, filtered through a celite pad, and washed with EA (10 mL x 3). The solvents were removed under reduced pressure to give a crude mixture. The crude mixture was purified by flash column chromatography to give the desired product **1p** *(95)*.

General procedure for synthesis of acetylene bromide (2a-2o)

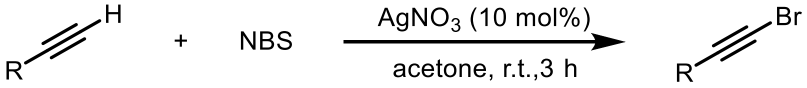

To a solution of alkyne (10 mmol, 1.0 equiv) was added N-bromosuccinimide (12 mmol, 1.2 equiv) and silver nitrate (1.0 mmol, 0.1 equiv). The mixture was allowed to stir at room temperature under protection from light until completion of the reaction. The mixture was then passed through short plug of silica and eluted with additional 20 mL of acetone and concentrated under reduced pressure to get crude product. The crude product was purified by column chromatography to give the desired bromoalkynes (**2a-2t**) *(17)*.

3. Procedure for the enantioselective hydroalkynylation of unactivated alkenes

General Procedure A:

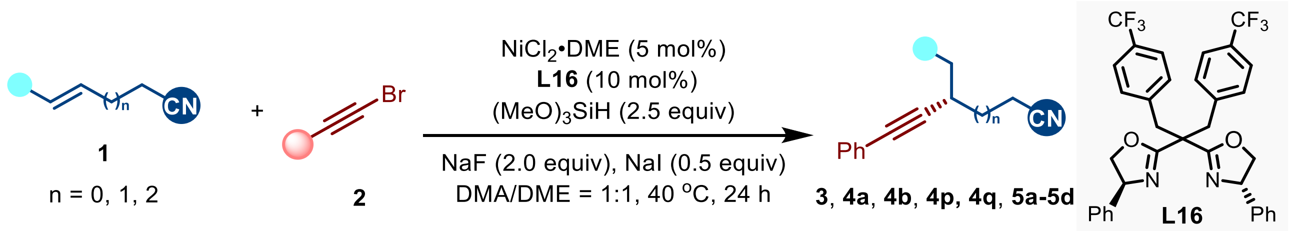

In an argon-filled glovebox, $NiCl_2$•DME (2.2 mg, 0.01 mmol, 5.0 mol%), Ligand (12.9 mg, 0.02 mmol, 10 mol%), relevant alkene substrate (0.2 mmol, 1.0 equiv), NaF (16.8 mg, 0.4 mmol, 2.0 equiv), NaI (15.0 mg 0.1 mmol, 0.5 equiv), DMA/DME (1:1, 1.0 mL) were added to a 4 mL reaction tube. Then relevant acetylene bromide (0.3 mmol, 1.5 equiv), $(MeO)_3SiH$ (64 µL, 0.5 mmol, 2.5 equiv) were added to the mixture. The reaction mixture was stirred at 40 °C for 24 h. Upon completion, the solvent was removed under reduced pressure, and the crude residue was purified by column chromatography on silica gel using a mixture of ethyl acetate and petroleum ether as eluent, yielding the desired chiral products **3**, **4a**, **4b**, **4p**, **4q**, **5a-5d**.

General Procedure B:

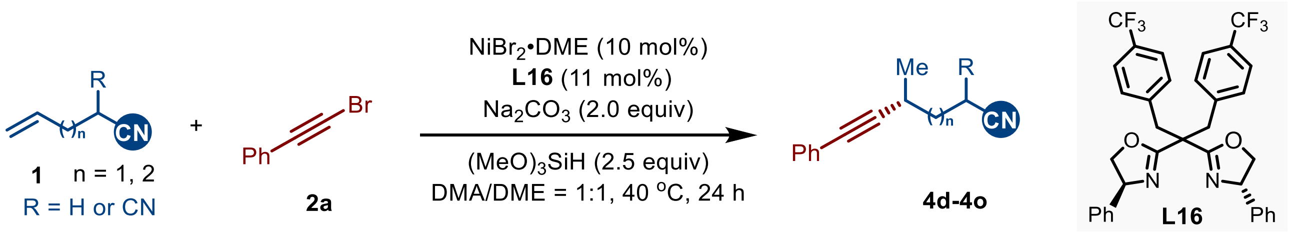

In an argon-filled glovebox, $NiBr_2$•DME (6.2 mg, 0.02 mmol, 10.0 mol%), Ligand (13.7 mg, 0.02 mmol, 11 mol%), relevant alkene substrate (0.2 mmol, 1.0 equiv), Na_2CO_3 (42.4 mg, 0.4 mmol, 2.0 equiv), DMA/DME (1:1, 1.0 mL) were added to a 4 mL reaction tube. Then relevant acetylene bromide (0.3 mmol, 1.5 equiv), $(MeO)_3SiH$ (64 µL, 0.5 mmol, 2.5 equiv) were added to the mixture. The reaction mixture was stirred at 40 °C for 24 h. Upon completion, the solvent was removed under reduced pressure, and the crude residue was purified by column chromatography on silica gel using a mixture of ethyl acetate and petroleum ether as eluent, yielding the desired chiral products **4d-4o**.

4. Characterization data of chiral products

(*R*)-3-methyl-5-phenylpent-4-ynenitrile (3a)

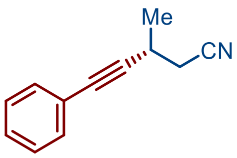

This compound was synthesized according to general procedure A. The residue was purified by column chromatography on silica gel (PE/EtOAc = 40:1) to afford the product **3a** (28.1 mg, 83% yield, 93% ee, rr > 20:1) as a yellow oil.

^{1}H NMR (400 MHz, Chloroform-*d*) δ 7.45 – 7.39 (m, 2H), 7.34 – 7.28 (m, 3H), 3.07 (h, *J* = 6.9 Hz, 1H), 2.68 – 2.52 (m, 2H), 1.44 (d, *J* = 6.9 Hz, 3H). **^{13}C NMR** (100 MHz, Chloroform-d) δ 131.8, 128.4 (two peaks overlap), 122.8, 117.8, 89.8, 82.9, 25.4, 24.2, 20.5. **HRMS (ESI)** m/z calculated for $C_{12}H_{11}N$ $[M+H]^+$ 170.0964; found: 170.0964. **Optical rotation**: $[\alpha]^{20}_D$ = 12.30 (c = 1.0 g/L, $CHCl_3$). The absolute configuration was assigned by analogy to that of **3e'**. **HPLC condition**: Chiral column OD-H, *n*-hexane/*i*-PrOH = 95:5, flow rate = 1.0 mL/min, wavelength = 254 nm, tR = 10.1 min for major isomer, tR = 8.7 min for minor isomer.

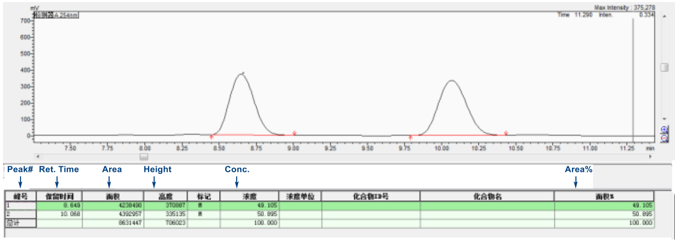

峰号	保留时间	面积	高度	标记	浓度	浓度单位	化合物ID号	化合物名	面积%
1	8.649	4238490	370887	M	49.105				49.105
2	10.068	4392957	335135	M	50.895				50.895
总计		8631447	706023		100.000				100.000

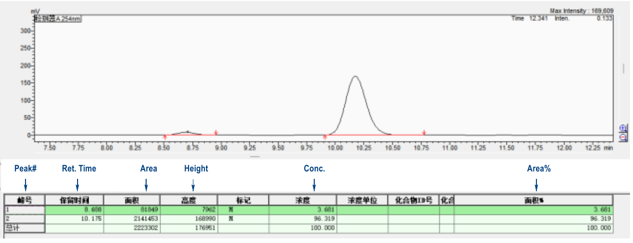

峰号	保留时间	面积	高度	标记	浓度	浓度单位	化合物ID号	化合	面积%
1	8.688	81849	7962	M	3.681				3.681
2	10.175	2141453	168990	M	96.319				96.319
总计		2223302	176951		100.000				100.000

(*R*)-3-methyl-5-(o-tolyl)pent-4-ynenitrile (3b)

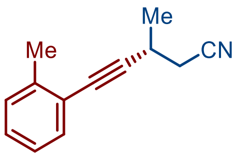

This compound was synthesized according to general procedure A. The residue was purified by column chromatography on silica gel (PE/EtOAc = 40:1) to afford the product **3b** (29.3 mg, 80% yield, 91% ee, rr > 20:1) as a yellow oil.

^{1}H NMR (400 MHz, Chloroform-*d*) δ 7.30 (d, *J* = 6.9 Hz, 1H), 7.14 – 7.08 (m, 2H), 7.06 – 7.01 (m, 1H), 3.03 (h, *J* = 6.7 Hz, 1H), 2.58 – 2.48 (m, 2H), 2.35 (s, 3H), 1.37 (d, *J* = 6.9 Hz, 3H). **^{13}C NMR** (100 MHz, Chloroform-d) δ 140.3, 131.9, 129.4, 128.3, 125.5, 122.5, 117.7, 93.7, 81.8, 25.5, 24.4, 20.7, 20.7. **HRMS (ESI)** m/z calculated for $C_{13}H_{13}N$ $[M+Na]^+$ 206.0940; found: 206.0938. **Optical rotation**: $[\alpha]^{20}_D$ =1.67 (c = 1.0 g/L, $CHCl_3$). The absolute configuration was assigned by analogy to that of **3e'**. **HPLC condition**: Chiral column OD-H, *n*-hexane/*i*-PrOH = 97:3, flow rate = 1.0 mL/min, wavelength = 254 nm, tR = 12.3 min for major isomer, tR = 10.6 min for minor isomer.

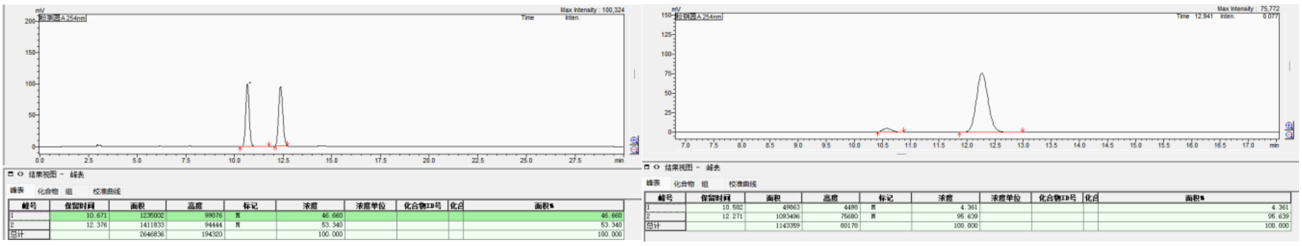

(*R*)-3-methyl-5-(m-tolyl)pent-4-ynenitrile (3c)

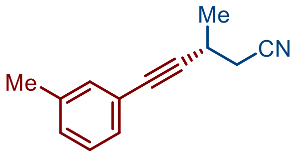

This compound was synthesized according to general procedure A. The residue was purified by column chromatography on silica gel (PE/EtOAc = 40:1) to afford the product **3c** (28.6 mg, 78% yield, 92% ee, rr > 20:1) as a yellow oil.

^{1}H NMR (600 MHz, Chloroform-*d*) δ 7.18 – 7.09 (m, 3H), 7.04 (d, *J* = 7.4 Hz, 1H), 2.98 (h, *J* = 6.9 Hz, 1H), 2.57 – 2.48 (m, 2H), 2.24 (s, 3H), 1.35 (d, *J* = 6.9 Hz, 3H). **^{13}C NMR** (100 MHz, Chloroform-d) δ 138.1, 132.4, 129.2, 128.8, 128.3, 122.6, 117.8, 89.4, 83.0, 25.4, 24.2, 21.3, 20.6. **HRMS (ESI)** m/z calculated for $C_{13}H_{13}N$ $[M+Na]^+$ 206.0940; found: 206.0939. **Optical rotation**: $[\alpha]^{20}_{D}$ = 1.67 (c = 1.0 g/L, $CHCl_3$). The absolute configuration was assigned by analogy to that of **3e'**. **HPLC condition**: Chiral column AD-H, *n*-hexane/*i*-PrOH = 99.5:0.5, flow rate = 0.8 mL/min, wavelength = 254 nm, tR = 15.0 min for major isomer, tR = 13.5 min for minor isomer.

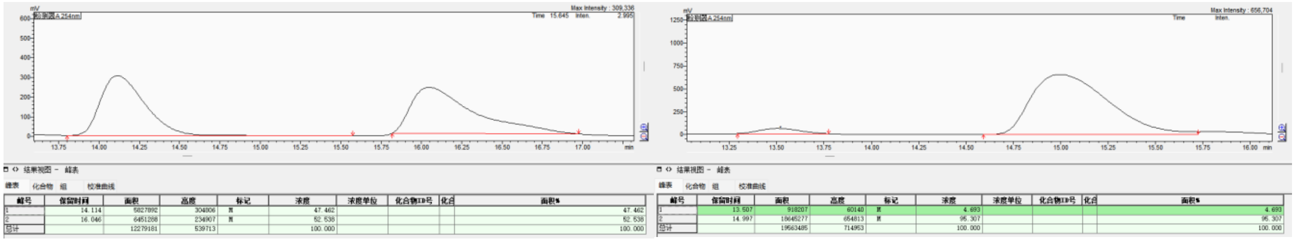

(*R*)-3-methyl-5-(4-propylphenyl)pent-4-ynenitrile (3d)

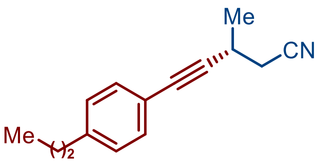

This compound was synthesized according to general procedure A. The residue was purified by column chromatography on silica gel (PE/EtOAc = 50:1) to afford the product **3d** (33.0 mg, 70% yield, 92% ee, rr > 20:1) as a yellow oil.

^{1}H NMR (400 MHz, Chloroform-*d*) δ 7.33 (d, *J* = 7.9 Hz, 2H), 7.11 (d, *J* = 7.9 Hz, 2H), 3.06 (h, *J* = 6.8 Hz, 1H), 2.67 – 2.53 (m, 4H), 1.62 (h, *J* = 7.4 Hz, 2H), 1.43 (d, *J* = 6.9 Hz, 3H), 0.92 (t, *J* = 7.4 Hz, 3H). **^{13}C NMR** (100 MHz, Chloroform-*d*) δ 143.3, 131.7, 128.6, 120.0, 117.8, 89.1, 83.1, 38.0, 25.5, 24.5, 24.3, 20.7, 13.9. **HRMS (ESI)** m/z calculated for $C_{15}H_{17}N$ $[M+Na]^+$ 234.1253; found: 234.1253. **Optical rotation**: $[\alpha]^{20}_{D}$ = 10.16 (c = 1.0 g/L, $CHCl_3$). The absolute configuration was assigned by analogy to that of **3e'**. **HPLC condition**: Chiral column OD-H, *n*-hexane/*i*-PrOH = 95:5, flow rate = 1.0 mL/min, wavelength = 254 nm, tR = 7.7 min for major isomer, tR = 6.9 min for minor isomer.

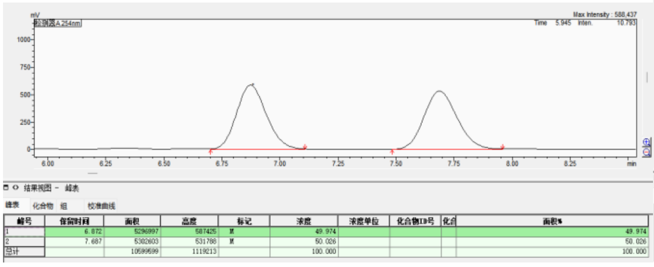

峰号	保留时间	面积	高度	标记	浓度	浓度单位	化合物ID号	化合	面积%
1	6.872	5296997	587425	M	49.974				49.974
2	7.687	5302603	531788	M	50.026				50.026
总计		10599599	1119213		100.000				100.000

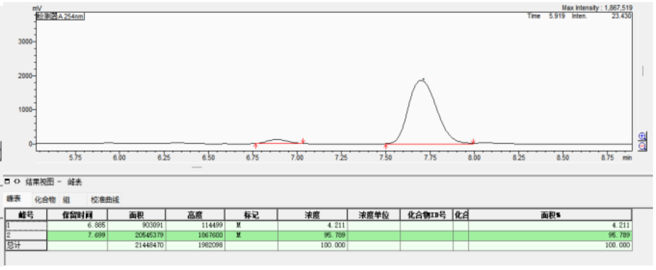

峰号	保留时间	面积	高度	标记	浓度	浓度单位	化合物ID号	化合	面积%
1	6.885	903091	114499	M	4.211				4.211
2	7.699	20545379	1867600	M	95.789				95.789
总计		21448470	1982098		100.000				100.000

(*R*)-5-(4-(tert-butyl)phenyl)-3-methylpent-4-ynenitrile (3e)

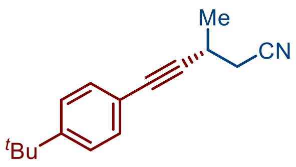

This compound was synthesized according to general procedure A. The residue was purified by column chromatography on silica gel (PE/EtOAc = 40:1) to afford the product **3e** (35.6 mg, 79% yield, 92% ee, rr > 20:1) as a yellow oil.

^{1}H NMR (400 MHz, Chloroform-*d*) δ 7.38–7.28 (m, 4H), 3.06 (h, *J* = 6.8 Hz, 1H), 2.68–2.53 (m, 2H), 1.43 (d, *J* = 6.9 Hz, 3H), 1.30 (s, 9H). **^{13}C NMR** (100 MHz, Chloroform-*d*) δ 151.7, 131.5, 125.4, 119.8, 117.8, 89.2, 83.0, 34.9, 31.3, 25.5, 24.3, 20.7. **HRMS (ESI)** m/z calculated for$C_{16}H_{19}N$ $[M+H]^+$ 226.1590; found: 226.1593.

(*R*)-5-(4-(tert-butyl)phenyl)-3-methylpent-4-ynamide (3e')

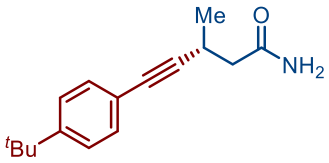

To a solution of the **3e** (45.0 mg, 0.2 mmol) in EtOH (2 mL) cooled 0 °C are added K_2CO_3 (3.0 equiv) and dropwise 30% H_2O_2 (0.5 mL). The reaction mixture was heated to rt for 2 h. After removal of the solvent, the

reaction mixture was concentrated. the crude mixture was purified by flash column chromatography on silica gel to provide **3e'** as a white solid (80%, 38.9 mg, 92% ee, rr > 20:1).

^{1}H NMR (400 MHz, Chloroform-*d*) δ 7.34 – 7.28 (m, 4H), 5.96 (s, 2H), 3.14 (h, *J* = 6.9 Hz, 1H), 2.54 – 2.36 (m, 2H), 1.32 (d, *J* = 6.9 Hz, 3H), 1.29 (s, 9H). **^{13}C NMR** (100 MHz, Chloroform-*d*) δ 173.7, 151.3, 131.4, 125.4, 120.3, 92.1, 81.9, 43.4, 34.8, 31.3, 23.8, 21.0. **HRMS (ESI)** m/z calculated forC$_{16}$H$_{22}$NO [M+H]$^+$ 244.1696; found: 244.1693.

Optical rotation: $[\alpha]^{20}_D$ = 7.23 (c = 1.0 g/L, CHCl$_3$). The absolute configuration was assigned by analogy to that of **3e'**. **HPLC condition**: Chiral column AD-H, *n*-hexane/*i*-PrOH = 97:3, flow rate = 0.6 mL/min, wavelength = 254 nm, tR = 34.0 min for major isomer, tR = 32.0 min for minor isomer.

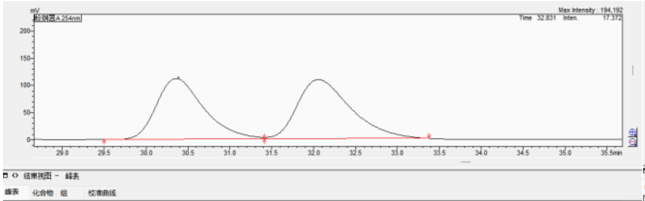

峰号	保留时间	面积	高度	标记	浓度	浓度单位	化合物ID号	化合	面积%
1	30.358	4404018	112152	M	47.686				47.686
2	32.057	4831463	109165	V M	52.314				52.314
总计		9235481	221317		100.000				100.000

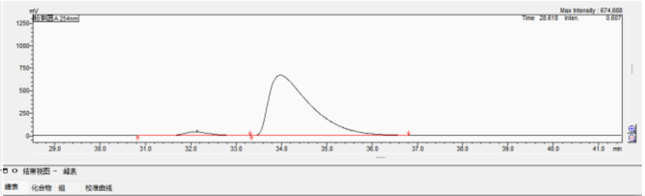

峰号	保留时间	面积	高度	标记	浓度	浓度单位	化合物ID号	化合	面积%
1	32.091	1755696	42481		3.936				3.936
2	33.976	42845451	672461	M	96.064				96.064
总计		44601147	714943		100.000				100.000

(*R*)-5-(4-methoxyphenyl)-3-methylpent-4-ynenitrile (3f)

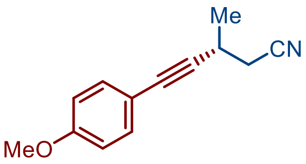

This compound was synthesized according to general procedure A. The residue was purified by column chromatography on silica gel (PE/EtOAc = 25:1) to afford the product **3f** (32.3 mg, 81% yield, 93% ee, rr > 20:1) as a yellow oil.

^{1}H NMR (400 MHz, Chloroform-*d*) δ 7.34 (d, *J* = 8.8 Hz, 2H), 6.82 (d, *J* = 8.7 Hz, 3H), 3.80 (s, 1H), 3.06 (h, *J* = 6.8 Hz, 2H), 2.68 – 2.54 (m, 3H), 1.43 (d, *J* = 6.9 Hz, 3H). **^{13}C NMR** (100 MHz, Chloroform-*d*) δ 159.7, 133.2, 117.9, 115.0, 114.0, 88.4, 82.8, 55.4, 25.5, 24.3, 20.7. **HRMS (ESI)** m/z calculated for C$_{13}$H$_{13}$NO [M+H]$^+$ 200.1070; found:200.1071. **Optical rotation**: $[\alpha]^{20}_D$ = -8.90 (c = 1.0 g/L, CHCl$_3$). The absolute configuration was assigned by analogy to that of **3e'**. **HPLC condition**: Chiral column OD-H, *n*-hexane/*i*-PrOH = 95:1, flow rate = 1.0 mL/min, wavelength = 254 nm, tR = 13.7 min for major isomer, tR = 12.4 min for minor isomer.

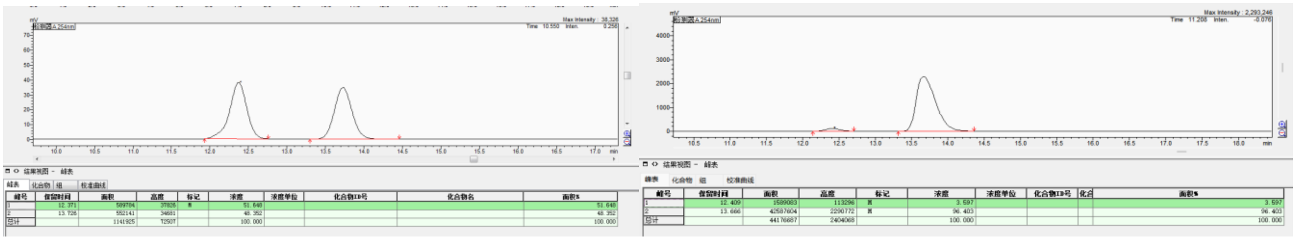

(*R*)-5-(3-fluorophenyl)-3-methylpent-4-ynenitrile (3g)

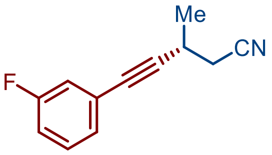

This compound was synthesized according to general procedure A. The residue was purified by column chromatography on silica gel (PE/EtOAc = 25:1) to afford the product **3g** (26.2 mg, 70% yield, 93% ee, rr > 20:1) as a yellow oil.

^{1}H NMR (400 MHz, Chloroform-*d*) δ 7.33 – 7.26 (m, 1H), 7.24 – 7.21 (m, 1H), 7.17 – 7.12 (m, 1H), 7.08 – 7.01 (m, 1H), 3.11 (h, *J* = 6.8 Hz, 1H), 2.72 – 2.57 (m, 2H), 1.47 (d, *J* = 6.9 Hz, 3H). **^{13}C NMR** (100 MHz, Chloroform-*d*) δ 162.4 (d, *J* = 246.6 Hz), 130.0 (d, *J* = 8.6 Hz), 127.7 (d, *J* = 3.0 Hz), 124.6, 118.7 (d, *J* = 22.9 Hz), 117.6, 115.8 (d, *J* = 21.1 Hz), 90.8, 81.9, 25.3, 24.2, 20.5. **^{19}F NMR** (376 MHz, Chloroform-*d*) δ -113.0. **Optical rotation**: $[\alpha]^{20}_D$ = 6.60 (c = 1.0 g/L, $CHCl_3$). The absolute configuration was assigned by analogy to that of **3e'**. **HRMS (ESI)** m/z calculated for $C_{12}H_{10}FN$ $[M+Na]^+$ 210.0689; found: 210.0696. **HPLC condition**: Chiral column OD-H, *n*-hexane/*i*-PrOH = 97:3, flow rate = 1.0 mL/min, wavelength = 254 nm, tR = 10.1 min for major isomer, tR = 9.1 min for minor isomer.

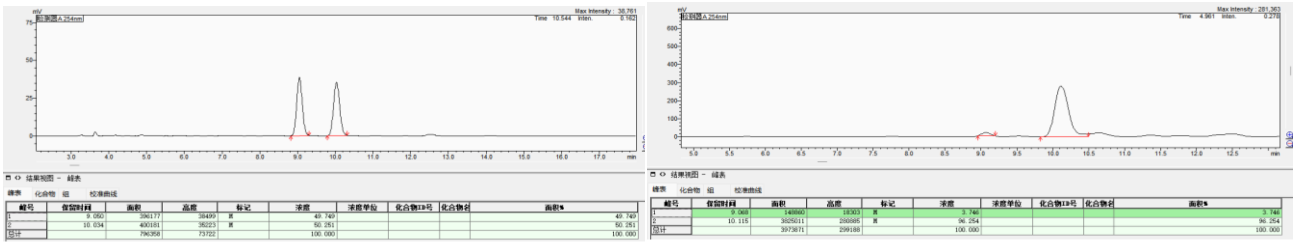

(*R*)-5-(4-chlorophenyl)-3-methylpent-4-ynenitrile (3h)

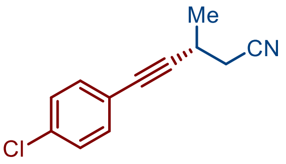

This compound was synthesized according to general procedure A. The residue was purified by column chromatography on silica gel (PE/EtOAc = 30:1) to afford the product **3h** (30.6 mg, 62% yield, 94% ee, rr > 20:1) as a yellow oil.

^{1}H NMR (400 MHz, Chloroform-*d*) δ 7.37 (d, *J* = 8.5 Hz, 2H), 7.31 (d, *J* = 8.5 Hz, 2H), 3.10 (h, *J* = 6.8 Hz, 1H), 2.70 – 2.57 (m, 2H), 1.46 (d, *J* = 6.9 Hz, 3H). **^{13}C NMR** (100 MHz, Chloroform-*d*) δ 134.4, 133.0, 128.7, 121.3, 117.7, 90.8, 81.9, 25.3, 24.2, 20.5. **HRMS (ESI)** m/z calculated for $C_{12}H_{10}ClN$ $[M+Na]^{+}$ 204.0575; found: 204.0575. **Optical rotation**: $[\alpha]^{20}_{D}$ = -5.60 (c = 1.0 g/L, $CHCl_3$). The absolute configuration was assigned by analogy to that of **3e'**. **HPLC condition**: Chiral column OD-H, *n*-hexane/*i*-PrOH = 98.8:0.2, flow rate = 1.0mL/min, wavelength = 254 nm, tR = 43.1 min for major isomer, tR = 40.5 min for minor isomer.

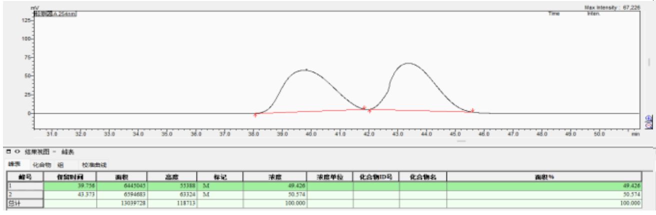
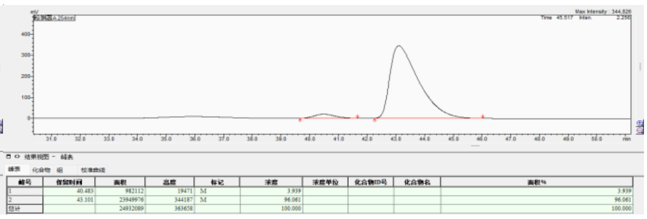

(*R*)-5-(3-bromophenyl)-3-methylpent-4-ynenitrile (3i)

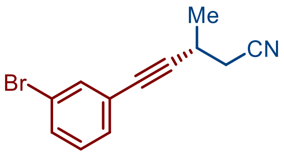

This compound was synthesized according to general procedure A. The residue was purified by column chromatography on silica gel (PE/EtOAc = 25:1) to afford the product **3i** (39.2 mg, 69% yield, 90% ee, rr > 20:1) as a yellow oil.

^{1}H NMR (400 MHz, Chloroform-*d*) δ 7.56 (s, 1H), 7.44 (d, *J* = 8.0 Hz, 1H), 7.34 (d, *J* = 7.8 Hz, 1H), 7.16 (t, *J* = 7.9 Hz, 1H), 3.07 (h, *J* = 6.8 Hz, 1H), 2.66 – 2.55 (m, 2H), 1.43 (d, *J* = 6.9 Hz, 3H). **^{13}C NMR** (100 MHz, Chloroform-*d*) δ 134.6, 131.6, 130.4, 129.8, 124.8, 122.2, 117.6, 91.2, 81.5, 25.3, 24.2, 20.5. **HRMS (ESI)** m/z calculated for$C_{12}H_{10}BrN$ $[M+Na]^{+}$ 269.9889; found: 269.9896. **Optical rotation**: $[\alpha]^{20}_{D}$ = -3.90 (c = 1.0 g/L, $CHCl_3$). The absolute configuration was assigned by analogy to that of **3e'**. **HPLC condition**: Chiral column OD-H, *n*-hexane/*i*-PrOH = 95:5, flow rate = 1.0 mL/min, wavelength = 254 nm, tR = 10.0 min for major isomer, tR = 8.5 min for minor isomer.

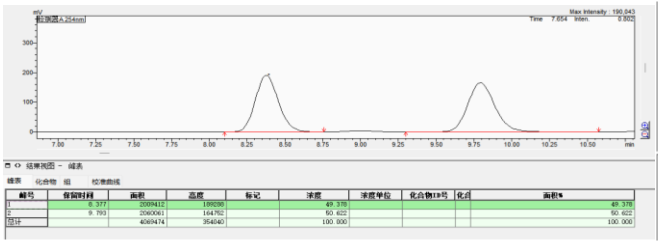
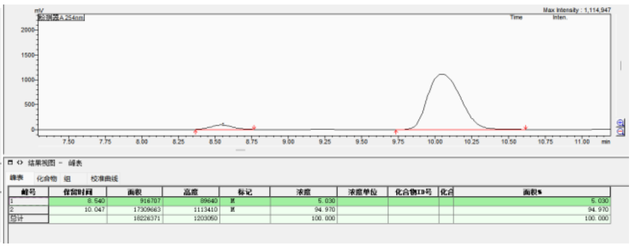

(*R*)-5-(4-bromophenyl)-3-methylpent-4-ynenitrile (3j)

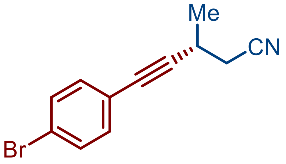

This compound was synthesized according to general procedure A. The residue was purified by column chromatography on silica gel (PE/EtOAc = 25:1) to afford the product **3j** (37.7 mg, 76% yield, 91% ee, rr > 20:1) as a yellow oil.

^{1}H NMR (400 MHz, Chloroform-*d*) δ 7.36 (d, J = 8.5 Hz, 2H), 7.20 (d, J = 8.5 Hz, 2H), 2.99 (h, J = 6.8 Hz, 1H), 2.59 – 2.47 (m, 2H), 1.36 (d, J = 6.9 Hz, 3H). **^{13}C NMR** (100 MHz, Chloroform-*d*) δ 133.3, 131.6, 122.6, 121.8, 117.7, 91.0, 82.0, 25.3, 24.3, 20.5. **HRMS (ESI)** m/z calculated for $C_{12}H_{10}BrN$ $[M+H]^+$ 248.0070; found: 248.0070. **Optical rotation**: $[\alpha]^{20}_D$ = 12.33 (c = 1.0 g/L, $CHCl_3$). The absolute configuration was assigned by analogy to that of **3e'**. **HPLC condition**: Chiral column AD-H, *n*-hexane/*i*-PrOH = 99.7:0.3, flow rate = 0.4 mL/min, wavelength = 254 nm, tR = 99.2 min for major isomer, tR = 96.8 min for minor isomer.

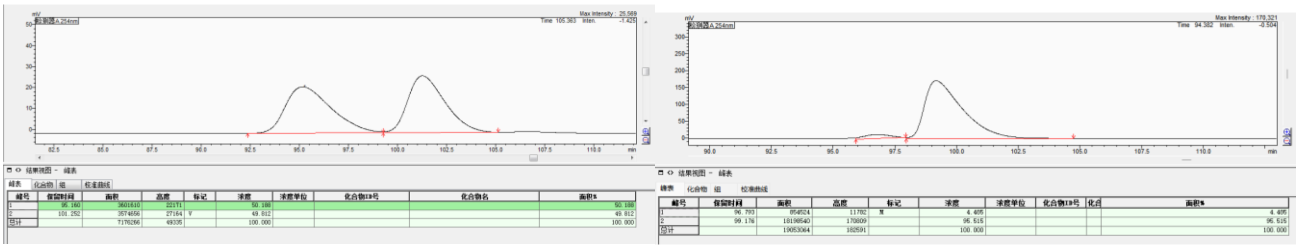

(*R*)-3-methyl-5-(4-(trifluoromethyl)phenyl)pent-4-ynenitrile (3k)

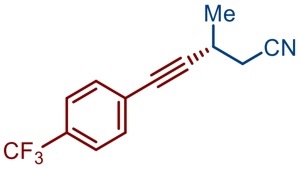

This compound was synthesized according to general procedure A. The residue was purified by column chromatography on silica gel (PE/EtOAc = 15:1) to afford the product **3k** (35.6 mg, 75% yield, 93% ee, rr > 20:1) as a yellow oil.

^{1}H NMR (400 MHz, Chloroform-*d*) δ 7.55 (d, J = 8.1 Hz, 2H), 7.51 (d, J = 8.2 Hz, 2H), 3.09 (h, J = 6.7 Hz, 1H), 2.66 – 2.59 (m, 2H), 1.45 (d, J = 7.0 Hz, 3H). **^{13}C NMR** (100 MHz, Chloroform-d) δ 132.1, 130.1 (q, $J_{C\text{-}F}$ = 32.7 Hz), 126.7, 125.3 (q, $J_{C\text{-}F}$ = 3.9 Hz), 123.9 (q, $J_{C\text{-}F}$ = 272.2 Hz). 117.6, 92.4, 81.8, 25.2, 24.3, 20.4. **^{19}F NMR** (376 MHz, Chloroform-*d*) δ -62.8. **HRMS (ESI)** m/z calculated for $C_{13}H_{10}F_3N$ $[M+H]^+$ 238.0838; found: 238.0832. **Optical rotation**: $[\alpha]^{20}_D$ = 13.23 (c = 1.0 g/L, $CHCl_3$). The absolute configuration was assigned by analogy to that of **3e'**. **HPLC condition**: Chiral

column OD-H, *n*-hexane/*i*-PrOH = 99.8:0.2, flow rate = 0.5 mL/min, wavelength = 254 nm, tR = 52.9 min for major isomer, tR = 51.1 min for minor isomer.

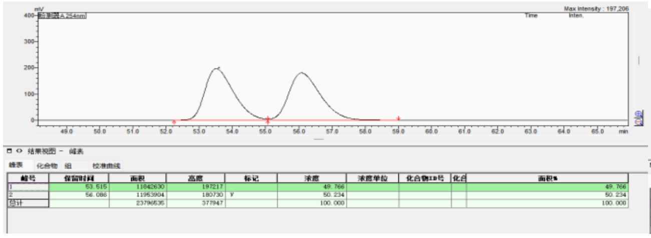

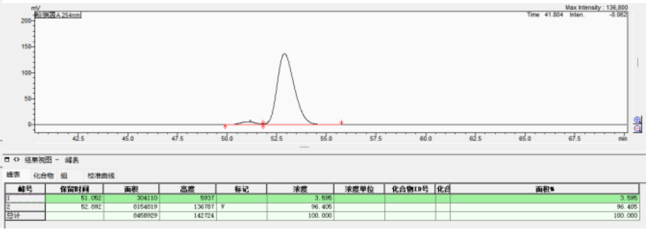

(*R*)-4-(4-cyano-3-methylbut-1-yn-1-yl)benzonitrile (3l)

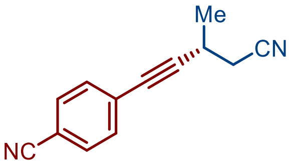

This compound was synthesized according to general procedure A. The residue was purified by column chromatography on silica gel (PE/EtOAc = 18:1) to afford the product **3l** (28.0 mg, 72% yield, 92% ee, rr > 20:1) as a yellow oil.

^{1}H NMR (400 MHz, Chloroform-*d*) δ 7.59 (d, *J* = 8.3 Hz, 2H), 7.49 (d, *J* = 8.3 Hz, 2H), 3.11 (h, *J* = 6.7 Hz, 1H), 2.66 – 2.55 (m, 2H), 1.45 (d, *J* = 6.9 Hz, 3H). **^{13}C NMR** (100 MHz, Chloroform-*d*) δ 132.4, 132.1, 127.8, 118.5, 117.5, 111.8, 94.4, 81.6, 25.2, 24.3, 20.3. **HRMS (ESI)** m/z calculated for$C_{13}H_{10}N_2$ $[M+Na]^+$ 217.0736; found: 217.0729. **Optical rotation**: $[\alpha]^{20}_D$ = -15.63 (c = 1.0 g/L, $CHCl_3$). The absolute configuration was assigned by analogy to that of **3e'**. **HPLC condition**: Chiral column AD-H, *n*-hexane/*i*-PrOH = 99.5:0.5., flow rate = 1.0 mL/min, wavelength = 254 nm, tR = 22.0 min for major isomer, tR = 19.4 min for minor isomer.

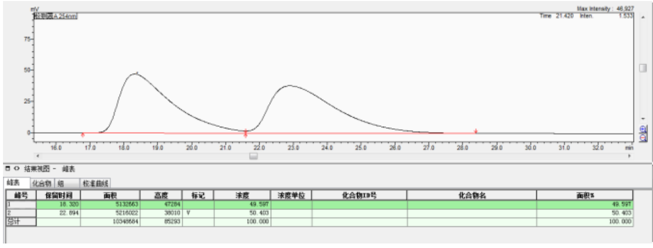

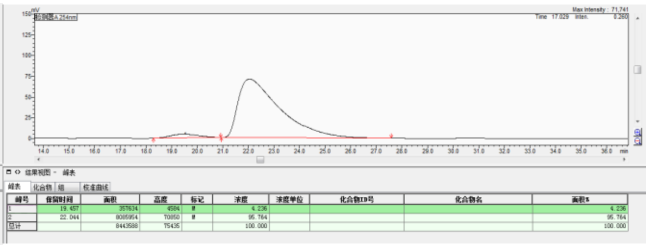

(*R*)-5-(4-formylphenyl)-3-methylpent-4-ynenitrile (3m)

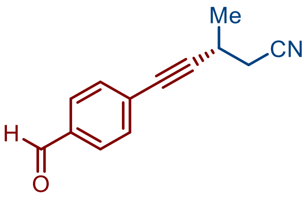

This compound was synthesized according to general procedure A. The residue was purified by column chromatography on silica gel (PE/EtOAc = 35:1) to afford the product **3m** (29.6 mg, 75% yield, 92% ee, rr > 20:1) as a yellow oil.

^{1}H NMR (400 MHz, Chloroform-*d*) δ 10.00 (s, 1H), 7.82 (d, *J* = 8.0 Hz, 2H), 7.56 (d, *J* = 8.1 Hz, 2H), 3.12 (h, *J* = 6.8 Hz, 1H), 2.69 – 2.59 (m, 2H), 1.46 (d, *J* = 6.9 Hz, 3H). **^{13}C NMR** (100 MHz, Chloroform-*d*) δ 191.6, 135.7, 132.4, 129.6, 129.1, 117.5, 94.0, 82.3, 25.3, 24.4, 20.4. **HRMS (ESI)** m/z calculated for $C_{20}H_{15}N$ $[M+Na]^+$ 220.0733; found: 220.0734. **Optical rotation**: $[\alpha]^{20}_D$ = -8.07 (c = 1.0 g/L, $CHCl_3$). The absolute configuration was assigned by analogy to that of **3e'**. **HPLC condition**: Chiral column AD-H, *n*-hexane/*i*-PrOH = 99:1, flow rate = 0.8 mL/min, wavelength = 254 nm, tR = 49.0 min for major isomer, tR = 45.8 min for minor isomer.

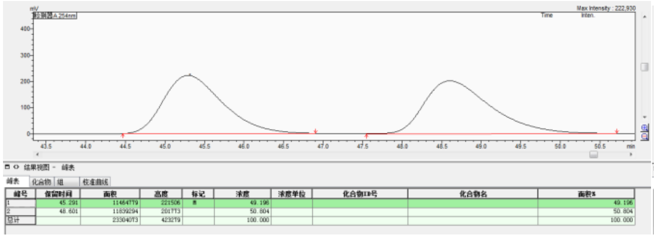

峰号	保留时间	面积	高度	标记	浓度	浓度单位	化合物ID号	化合物名	面积%
1	45.291	11464719	221506	M	49.196				49.196
2	48.601	11839294	201713		50.804				50.804
总计		23304013	423219		100.000				100.000

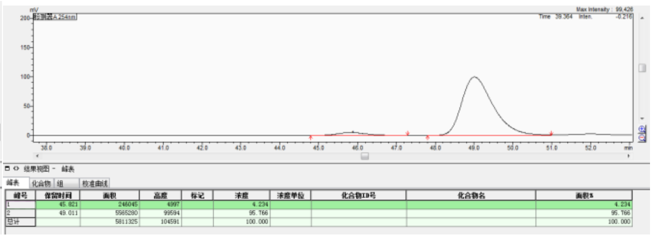

峰号	保留时间	面积	高度	标记	浓度	浓度单位	化合物ID号	化合物名	面积%
1	45.821	246045	4997		4.234				4.234
2	49.011	5565280	99594		95.766				95.766
总计		5811325	104591		100.000				100.000

(*R*)-5-(4-acetylphenyl)-3-methylpent-4-ynenitrile (3n)

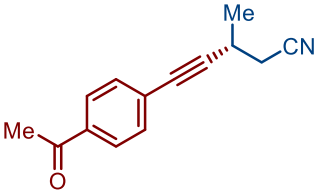

This compound was synthesized according to general procedure A. The residue was purified by column chromatography on silica gel (PE/EtOAc = 30:1) to afford the product **3n** (30.0 mg, 71% yield, 91% ee, rr > 20:1) as a yellow oil.

^{1}H NMR (400 MHz, Chloroform-*d*) δ 7.89 (d, *J* = 8.4 Hz, 2H), 7.49 (d, *J* = 8.4 Hz, 2H), 3.10 (h, *J* = 6.8 Hz, 1H), 2.67 – 2.61 (m, 2H), 2.59 (s, 3H), 1.45 (d, *J* = 6.9 Hz, 3H). **^{13}C NMR** (100 MHz, Chloroform-*d*) δ 197.5, 136.4, 132.0, 128.3, 127.7, 117.6, 93.2, 82.3, 26.8, 25.3, 24.4, 20.4. **HRMS (ESI)** m/z calculated for $C_{14}H_{13}NO$ $[M+H]^+$ 212.1070; found, 212.1070. **Optical rotation**: $[\alpha]^{20}_D$ = -16.23 (c = 1.0 g/L, $CHCl_3$). The absolute configuration was assigned by analogy to that of **3e'**. **HPLC condition**: Chiral column AD-H, *n*-hexane/*i*-PrOH = 95:5, flow rate = 1.0 mL/min, wavelength = 254 nm, tR = 16.1 min for major isomer, tR = 15.3 min for minor isomer.

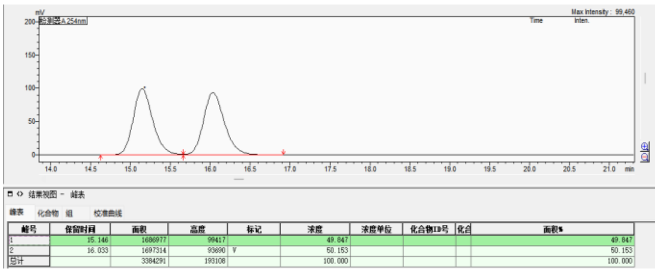

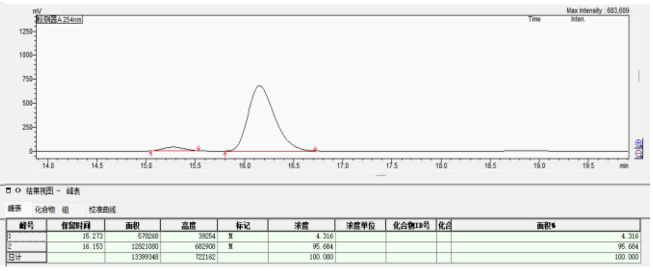

methyl (*R*)-4-(4-cyano-3-methylbut-1-yn-1-yl)benzoate (3o)

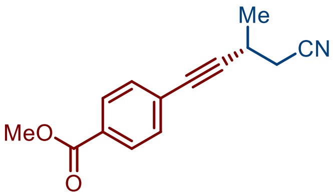

This compound was synthesized according to general procedure A. The residue was purified by column chromatography on silica gel (PE/EtOAc = 25:1) to afford the product **3o** (32.7 mg, 71% yield, 92% ee, rr > 20:1) as a yellow oil.

^{1}H NMR (400 MHz, Chloroform-*d*) δ 7.97 (d, *J* = 8.4 Hz, 2H), 7.47 (d, *J* = 8.4 Hz, 2H), 3.91 (s, 3H), 3.10 (h, *J* = 6.8 Hz, 1H), 2.70 – 2.57 (m, 2H), 1.45 (d, *J* = 6.9 Hz, 3H). **^{13}C NMR** (100 MHz, Chloroform-*d*) δ 166.6, 131.8, 129.7, 129.6, 127.5, 117.6, 92.9, 82.4, 52.4, 25.3, 24.3, 20.4. **HRMS (ESI)** m/z calculated for $C_{14}H_{13}NO_2$ $[M+Na]^+$ 250.0838; found: 250.0842. **Optical rotation**: $[\alpha]^{20}_{D}$ = -20.30 (c = 1.0 g/L, $CHCl_3$). The absolute configuration was assigned by analogy to that of **3e'**. **HPLC condition**: Chiral column AD-H, *n*-hexane/*i*-PrOH = 98:1.5, flow rate = 0.8 mL/min, wavelength = 254 nm, tR = 27.9 min for major isomer, tR = 26.3 min for minor isomer.

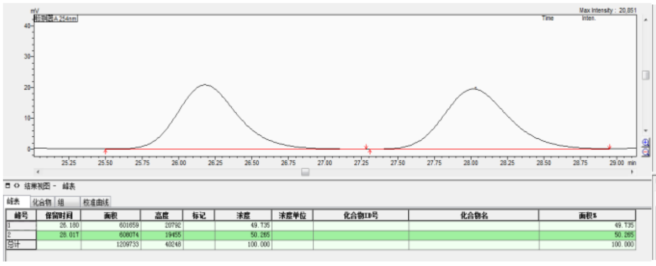

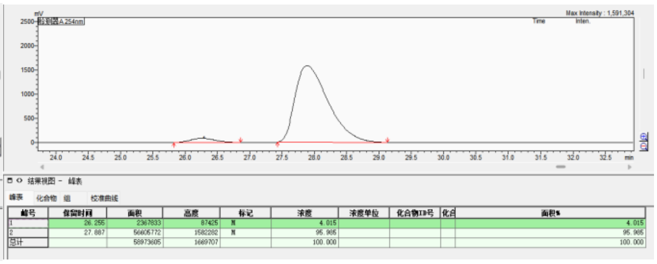

(*R*)-3-methyl-5-(naphthalen-1-yl)pent-4-ynenitrile (3p)

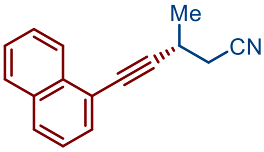

This compound was synthesized following general procedure A at 30°C. The residue was purified by column chromatography on silica gel (PE/EtOAc = 40:1) to afford the product **3p** (32.9 mg, 75% yield, 93% ee, rr > 20:1) as a brown oil.

^{1}H NMR (400 MHz, Chloroform-*d*) δ 8.33 (d, *J* = 9.4 Hz, 1H), 7.87 – 7.81 (m, 2H), 7.67 (dd, *J* = 7.2, 1.2 Hz, 1H), 7.62 – 7.57 (m, 1H), 7.55 – 7.50 (m, 1H), 7.45 – 7.40 (m, 1H), 3.23 (h, *J* = 6.8 Hz, 1H), 2.75 – 2.65 (m, 2H), 1.54 (d, *J* = 6.9 Hz, 3H). **^{13}C NMR** (100 MHz, Chloroform-*d*) δ 133.4, 133.2, 130.6, 128.9, 128.4, 126.9, 126.5, 126.1, 125.2, 120.4, 117.8, 94.7, 81.0, 25.5, 24.6, 20.8. **HRMS (ESI)** m/z calculated for $C_{16}H_{13}N$ $[M+H]^+$ 220.1121; found: 220.1119. **Optical rotation**: $[\alpha]^{20}_D$ = -25.57 (c = 1.0 g/L, $CHCl_3$). The absolute configuration was assigned by analogy to that of **3e'**. **HPLC condition**: Chiral column OD-H, *n*-hexane/*i*-PrOH = 95:5, flow rate = 1.0 mL/min, wavelength = 254 nm, tR = 15.3 min for major isomer, tR = 12.7 min for minor isomer.

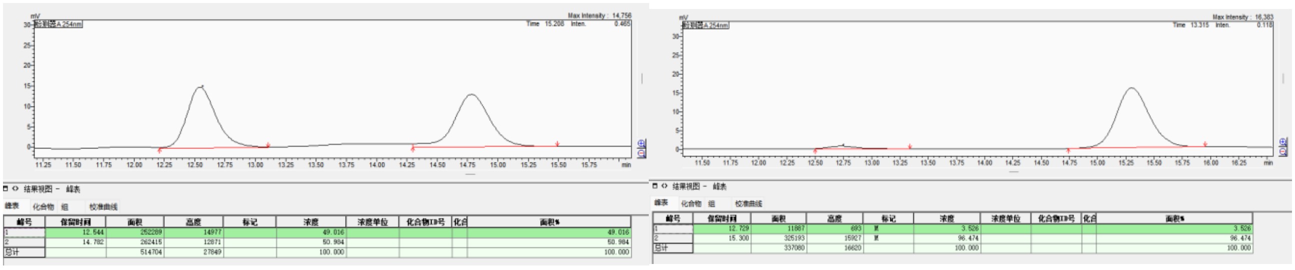

(*R*)-3-methyl-5-(phenanthren-9-yl)pent-4-ynenitrile (3q)

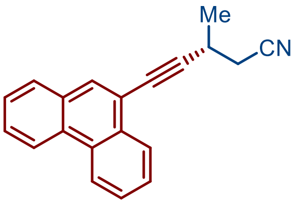

This compound was synthesized according to general procedure A. The residue was purified by column chromatography on silica gel (PE/EtOAc = 40:1) to afford the product **3q** (43.6 mg, 81% yield, 91% ee, rr > 20:1) as a yellow oil.

^{1}H NMR (400 MHz, Chloroform-*d*) δ 8.71 – 8.63 (m, 2H), 8.45 – 8.37 (m, 1H), 7.98 (s, 1H), 7.85 (dd, *J* = 7.9, 1.5 Hz, 1H), 7.72 – 7.63 (m, 3H), 7.63 – 7.57 (m, 1H), 3.27 (h, *J* = 6.8 Hz, 1H), 2.79 – 2.68 (m, 2H), 1.57 (d, *J* = 6.9 Hz, 3H). **^{13}C NMR** (100 MHz, Chloroform-d) δ 132.2, 131.20, 131.18, 130.4, 130.2, 128.6, 127.6, 127.22, 127.20, 127.1, 126.9, 122.9, 122.7, 119.1, 117.9, 94.3, 81.2, 25.6, 24.7, 20.8. **HRMS (ESI)** m/z calculated for $C_{20}H_{15}N$ $[M+H]^+$270.1277; found: 270.1277. **Optical rotation**: $[\alpha]^{20}_D$ = 14.31 (c = 1.0 g/L, $CHCl_3$). The absolute configuration was assigned by analogy to that of **3e'**. **HPLC condition**: Chiral column AD-H, *n*-hexane/*i*-PrOH = 95:5, flow rate = 1 mL/min, wavelength = 254 nm, tR = 12.2 min for major isomer, tR = 11.6 min for minor isomer.

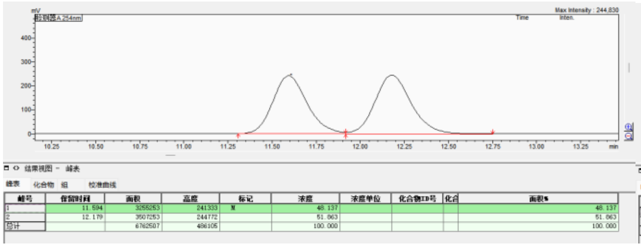

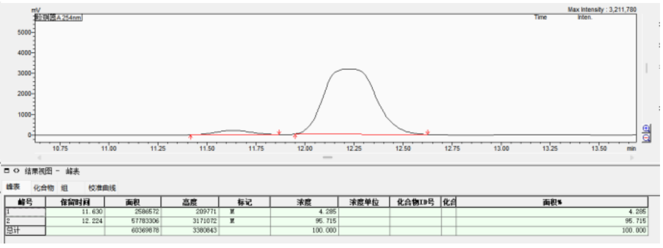

(*R*)-3-methyl-5-(thiophen-2-yl)pent-4-ynenitrile (3r)

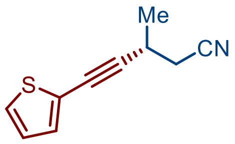

This compound was synthesized according to general procedure A. The residue was purified by column chromatography on silica gel (PE/EtOAc = 40:1) to afford the product **3r** (22.8 mg, 65% yield, 92% ee, rr > 20:1) as a colorless oil.

^{1}H NMR (400 MHz, Chloroform-*d*) δ 7.22 (d, *J* = 5.2 Hz, 1H), 7.17 (d, *J* = 3.6 Hz, 1H), 6.97 – 6.92 (m, 1H), 3.08 (h, *J* = 6.8 Hz, 1H), 2.70 – 2.50 (m, 2H), 1.42 (d, *J* = 6.9 Hz, 3H). **^{13}C NMR** (100 MHz, Chloroform-*d*) δ 132.1, 127.01, 126.97, 122.7, 117.6, 93.6, 76.1, 25.1, 24.4, 20.3. **HRMS (ESI)** m/z calculated for $C_{10}H_9NS$ $[M+Na]^+$ 198.0348; found:198.0352. **Optical rotation**: $[\alpha]^{20}_{D}$ = 11.00 (c = 1.0 g/L, $CHCl_3$). The absolute configuration was assigned by analogy to that of **3e'**. **HPLC condition:** Chiral column OD-H, *n*-hexane/*i*-PrOH = 95.5:0.5, flow rate = 0.8 mL/min, wavelength = 254 nm, tR = 54.3 min for major isomer, tR = 51.3 min for minor isomer.

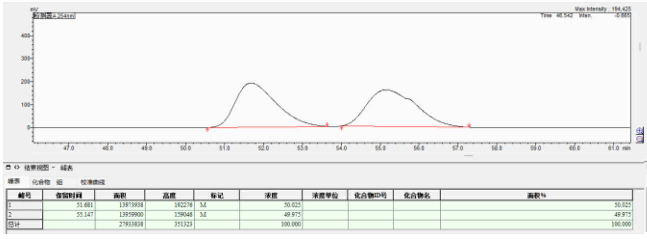

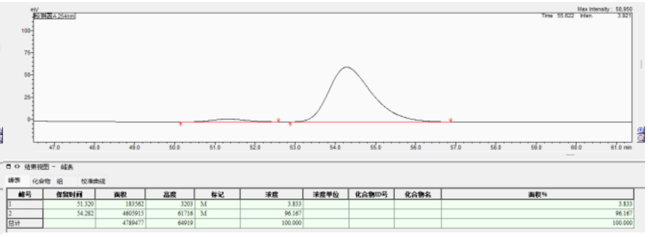

(*R*)-3-methyl-5-(pyridin-2-yl)pent-4-ynenitrile (3s)

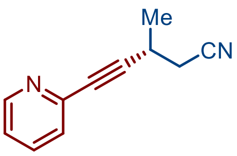

This compound was synthesized according to general procedure A. The residue was purified by column chromatography on silica gel (PE/EtOAc = 13:1) to afford the product **3s** (16.3 mg, 48% yield, 30% ee, rr > 20:1) as a colorless oil.

^{1}H NMR (400 MHz, Chloroform-*d*) δ 8.65 (s, 1H), 8.54 (d, *J* = 4.9 Hz, 1H), 7.77 (d, *J* = 8.0 Hz, 1H), 7.34 – 7.27 (m, 1H), 3.11 (h, *J* = 6.8 Hz, 1H), 2.69 – 2.58 (m, 2H), 1.46 (d, *J* = 6.9 Hz, 3H). **^{13}C NMR** (100 MHz, Chloroform-*d*) δ 151.4, 147.7, 139.9, 123.5, 120.5, 117.5, 94.1, 79.4, 25.2, 24.3, 20.4.

HRMS (ESI) m/z calculated for $C_{11}H_{10}N_2$ $[M+Na]^+$ 193.0736; found: 193.0738. **Optical rotation**: $[\alpha]^{20}_D$ = 4.33 (c = 1.0 g/L, $CHCl_3$). The absolute configuration was assigned by analogy to that of **3e'**. **HPLC condition**: Chiral column OD-H, *n*-hexane/*i*-PrOH = 98:2, flow rate = 0.8 mL/min, wavelength = 254 nm, tR = 40.9 min for major isomer, tR = 35.8 min for minor isomer.

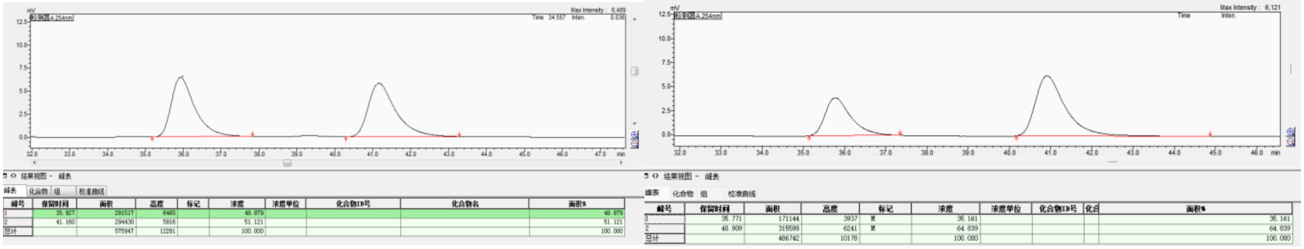

(*R*)-3-methyl-7-phenylhept-4-ynenitrile (3t)

This compound was synthesized according to general procedure A. The residue was purified by column chromatography on silica gel (PE/EtOAc = 20:1) to afford the product **3t** (17.4 mg, 44% yield, , 90% ee, rr > 20:1) as a yellow oil.

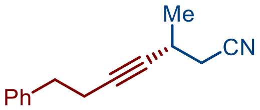

^{1}H NMR (400 MHz, Chloroform-*d*) δ 7.31 (t, *J* = 7.5 Hz, 2H), 7.25 – 7.19 (m, 3H), 2.85 – 2.74 (m, 3H), 2.51 – 2.39 (m, 4H), 1.29 (d, *J* = 6.8 Hz, 3H). **^{13}C NMR** (100 MHz, Chloroform-*d*) δ 140.7, 128.6, 128.4, 126.4, 118.0, 82.3, 81.5, 35.2, 25.6, 20.9, 20.8. **HRMS (ESI)** m/z calculated for $C_{14}H_{16}N$ $[M+H]^+$ 198.1277; found: 198.1278. **Optical rotation**: $[\alpha]^{20}_D$ = -2.53 (c = 1.0 g/L, $CHCl_3$). The absolute configuration was assigned by analogy to that of **3e'**. **HPLC condition**: Chiral column AD-H, *n*-hexane/*i*-PrOH = 96:4, flow rate = 1.0 mL/min, wavelength = 254 nm, tR = 11.3 min for major isomer, tR = 13.8 min for minor isomer.

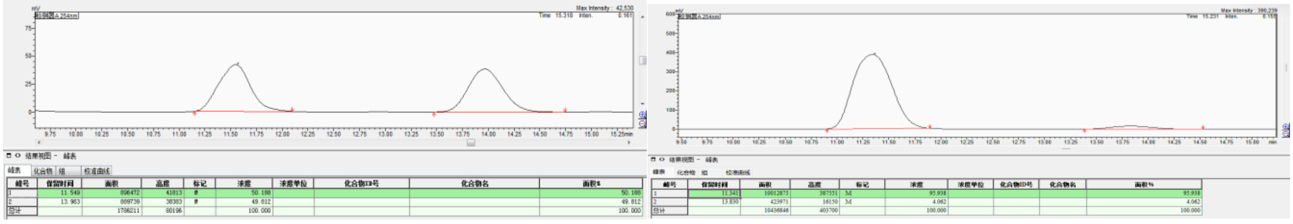

(*R*)-6-cyano-5-methylhex-3-yn-1-yl benzoate (3u)

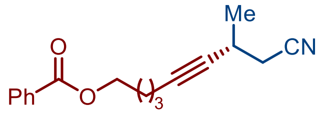

This compound was synthesized according to general procedure A. The residue was purified by column chromatography on silica gel (PE/EtOAc = 20:1) to afford the product **3u** (44.1 mg, 79% yield, 94% ee, rr > 20:1) as a colorless oil.

^{1}H NMR (400 MHz, Chloroform-*d*) δ 8.06 – 8.03 (m, 2H), 7.59 – 7.53 (m, 1H), 7.47 – 7.41 (m, 2H), 4.34 (t, *J* = 6.4 Hz, 2H), 2.87 – 2.75 (m, 1H), 2.53 – 2.41 (m, 2H), 2.26 (td, *J* = 7.0, 2.2 Hz, 2H), 1.94 – 1.84 (m, 2H), 1.71 – 1.60 (m, 2H), 1.30 (d, *J* = 6.9 Hz, 3H). **^{13}C NMR** (100 MHz, Chloroform-*d*) δ 166.7, 133.0, 130.5, 129.7, 128.5, 118.0, 82.4, 81.3, 64.6, 25.7, 25.4, 23.7, 20.9, 18.4. **HRMS (ESI)** m/z calculated for $C_{17}H_{19}NO_2$ $[M+H]^+$ 270.1489; found: 270.1492. **Optical rotation**: $[\alpha]^{20}_D$ = -1.07 (c = 1.0 g/L, $CHCl_3$). The absolute configuration was assigned by analogy to that of **3e'**. **HPLC condition**: Chiral column OD-H, *n*-hexane/*i*-PrOH = 98:2, flow rate = 0.5 mL/min, wavelength = 254 nm, tR = 38.1 min for major isomer, tR = 37.2 min for minor isomer.

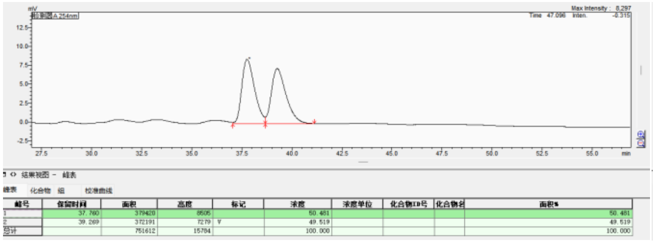

峰号	保留时间	面积	高度	标记	浓度	浓度单位	化合物ID号	化合物名	面积%
1	37.760	379420	8505		50.481				50.481
2	39.269	372191	7279	V	49.519				49.519
总计		751612	15784		100.000				100.000

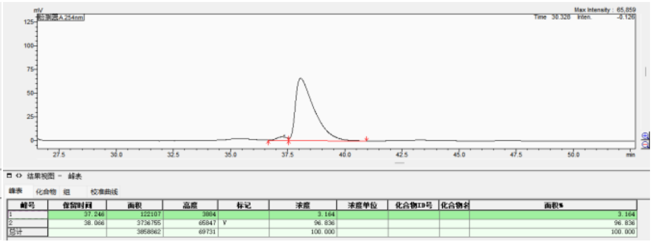

峰号	保留时间	面积	高度	标记	浓度	浓度单位	化合物ID号	化合物名	面积%
1	37.246	122107	3884		3.164				3.164
2	38.066	3736755	65847	V	96.836				96.836
总计		3858862	69731		100.000				100.000

(*R*)-8-(1,3-dioxoisoindolin-2-yl)-3-methyloct-4-ynenitrile (3v)

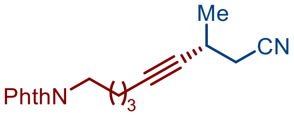

This compound was synthesized according to general procedure A. The residue was purified by column chromatography on silica gel (PE/EtOAc = 10:1) to afford the product **3v** (29.1 mg, 52% yield, 92% ee, rr > 20:1) as a yellow oil.

^{1}H NMR (400 MHz, Chloroform-*d*) δ 7.84 – 7.77 (m, 2H), 7.73 – 7.66 (m, 2H), 3.75 (t, *J* = 7.0 Hz, 2H), 2.73 – 2.63 (m, 1H), 2.48 – 2.35 (m, 2H), 2.21 (td, *J* = 7.0, 2.1 Hz, 2H), 1.86 (p, *J* = 7.0 Hz, 2H), 1.22 (d, *J* = 6.8 Hz, 3H). **^{13}C NMR** (100 MHz, Chloroform-*d*) δ 168.4, 134.0, 132.1, 123.2, 117.9, 81.5, 37.2, 27.5, 25.5, 23.6, 20.6, 16.4. **HRMS (ESI)** m/z calculated for $C_{17}H_{17}N_2O_2$ $[M+H]^+$ 281.1285; found: 281.1283. **Optical rotation**: $[\alpha]^{20}_D$ = 5.33 (c = 1.0 g/L, $CHCl_3$). The absolute configuration was assigned by analogy to that of **3e'**. **HPLC condition**: Chiral column AD-H, *n*-hexane/*i*-PrOH = 95:5,

flow rate = 1.0 mL/min, wavelength = 254 nm, tR = 28.1 min for major isomer, tR = 25.8 min for minor isomer.

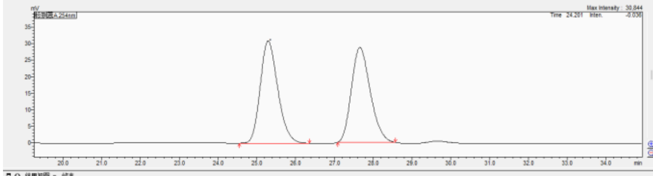

峰号	保留时间	面积	高度	标记	浓度	浓度单位	化合物ID号	化合物名	面积%
1	25.291	974006	30902	M	50.085				50.085
2	27.655	970693	28670	M	49.915				49.915
总计		1944698	59572		100.000				100.000

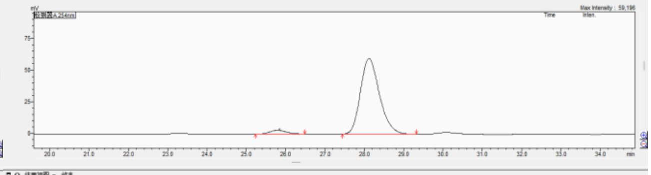

峰号	保留时间	面积	高度	标记	浓度	浓度单位	化合物ID号	化合物名	面积%
1	25.788	89039	3027	M	4.243				4.243
2	28.118	2009342	59765	M	95.757				95.757
总计		2098381	62792		100.000				100.000

(*R*)-4-methyl-6-phenylhex-5-ynenitrile (4a)

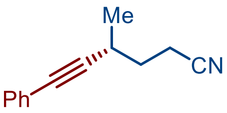

This compound was synthesized according to general procedure A. The residue was purified by column chromatography on silica gel (PE/EtOAc = 40:1) to afford the product **4a** (24.2 mg, 66% yield, 84% ee, rr = 90:10) as a yellow oil.

^{1}H NMR (400 MHz, Chloroform-*d*) δ 7.43 – 7.36 (m, 2H), 7.34 – 7.27 (m, 3H), 2.90 – 2.78 (m, 1H), 2.67 – 2.50 (m, 2H), 2.02 – 1.75 (m, 2H), 1.32 (d, *J* = 6.9 Hz, 3H). **^{13}C NMR** (100 MHz, Chloroform-*d*) δ 131.7, 128.4, 128.2, 123.3, 119.7, 91.4, 82.7, 32.6, 26.2, 20.9, 15.6. **HRMS (ESI)** m/z calculated for$C_{13}H_{13}N$ [M+Na^{+}] 206.0940; found: 206.0941. **Optical rotation**: $[\alpha]^{20}_{D}$ = 3.07 (c = 1.0 g/L, $CHCl_3$). The absolute configuration was assigned by analogy to that of **3e'**. **HPLC condition**: Chiral column OD-H, *n*-hexane/*i*-PrOH = 99:1, flow rate = 0.5 mL/min, wavelength = 254 nm, tR = 35.2 min for major isomer, tR = 38.2 min for minor isomer.

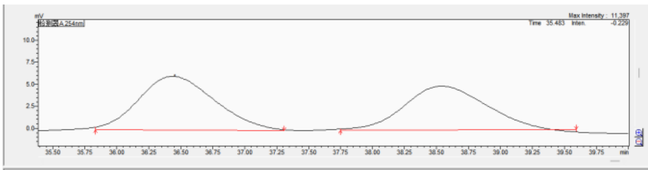

峰号	保留时间	面积	高度	标记	浓度	浓度单位	化合物ID号	化合	面积%
1	36.436	251499	6089	M	53.447				53.447
2	38.537	219061	4970	M	46.553				46.553
总计		470560	11059		100.000				100.000

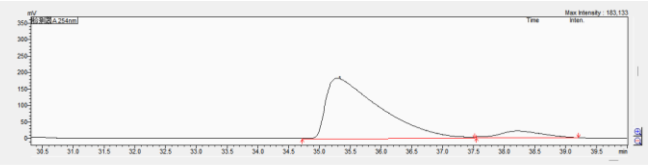

峰号	保留时间	面积	高度	标记	浓度	浓度单位	化合物ID号	化合	面积%
1	35.287	11176794	183489	M	92.060				92.060
2	38.212	963933	20177	M	7.940				7.940
总计		12140726	203666		100.000				100.000

(*R*)-2-(2-methyl-4-phenylbut-3-yn-1-yl)malononitrile (4b)

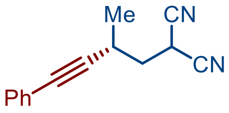

This compound was synthesized according to general procedure A. The residue was purified by column chromatography on silica gel (PE/EtOAc = 10:1) to afford the product **4b** (33.7 mg, 81% yield, 92% ee, rr = 91:9) as a green oil.

^{1}H NMR (400 MHz, Chloroform-*d*) δ 7.45 – 7.38 (m, 2H), 7.36 – 7.30 (m, 3H), 4.12 (dd, *J* = 10.7, 4.8 Hz, 1H), 3.09 – 2.95 (m, 1H), 2.34 – 2.23 (m, 1H), 2.22 – 2.09 (m, 1H), 1.41 (d, *J* = 6.9 Hz, 3H). **^{13}C NMR** (100 MHz, Chloroform-*d*) δ 131.8, 128.7, 128.5, 122.4, 112.8, 112.4, 88.8, 84.5, 37.7, 25.0, 21.4, 20.7. **HRMS (ESI)** m/z calculated for $C_{14}H_{12}N_2$ $[M+H]^+$ 209.1073; found: 209.1070. **Optical rotation**: $[\alpha]^{20}_D$ = 11.67 (c = 1.0 g/L, $CHCl_3$). The absolute configuration was assigned by analogy to that of **3e'**. **HPLC condition**: Chiral column OD-H, *n*-hexane/*i*-PrOH = 95:5, flow rate = 1.0 mL/min, wavelength = 254 nm, tR = 21.1 min for major isomer, tR = 14.5 min for minor isomer.

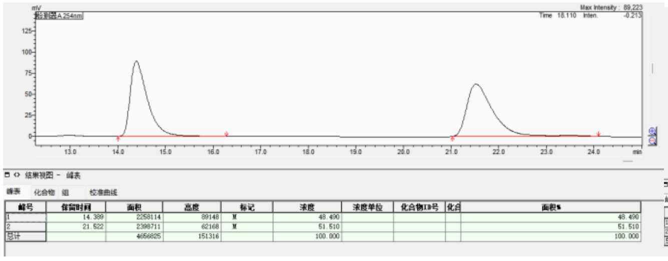
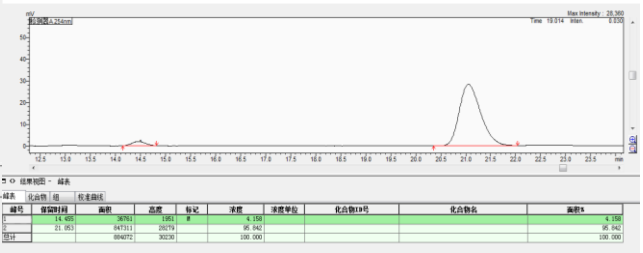

(*R*)-2-(4-methoxybenzyl)-2-(2-methyl-4-phenylbut-3-yn-1-yl)malononitrile (4c)

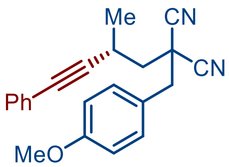

This compound was synthesized according to general procedure A. The residue was purified by column chromatography on silica gel (PE/EtOAc = 10:1) to afford the product **4c** (56.4 mg, 86% yield, 81% ee, rr = 92:8) as a colorless oil.

^{1}H NMR (400 MHz, Chloroform-*d*) δ 7.47–7.42 (m, 2H), 7.33 (d, *J* = 8.3 Hz, 2H), 7.31–7.24 (m, 3H), 6.93 (d, *J* = 8.2 Hz, 2H), 3.82 (s, 3H), 3.29 (q, *J* = 13.8 Hz, 2H), 3.21–3.08 (m, 1H), 2.23 (dd, *J* = 13.9, 10.0 Hz, 1H), 2.07 (dd, *J* = 13.9, 4.8 Hz, 1H), 1.42 (d, *J* = 6.8 Hz, 3H). **^{13}C NMR** (100 MHz, Chloroform-*d*) δ 160.1, 131.8, 131.7, 128.4, 128.3, 123.9, 123.0, 115.3, 114.5, 90.3, 84.1, 55.4, 43.8, 42.8, 38.4, 24.6, 22.0. **HRMS (ESI)** m/z calculated for $C_{22}H_{20}N_2O$ $[M+H]^+$ 329.1649; found: 329.1648. **Optical rotation**: $[\alpha]^{20}_D$ =20.30 (c = 1.0 g/L, $CHCl_3$). The absolute configuration was assigned by analogy to that of **3e'**. **HPLC condition**: Chiral column OD-H, n-hexane/i-PrOH = 95:5, flow rate = 1.0 mL/min, wavelength = 25 4 nm, tR = 21.5 min for major isomer, tR = 34.0 min for minor isomer.

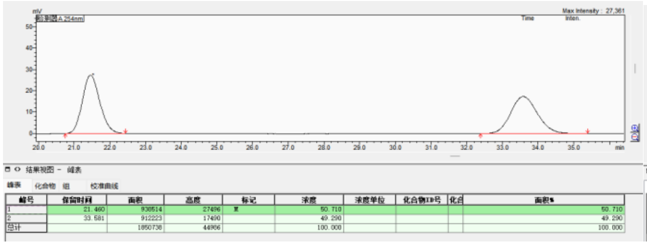
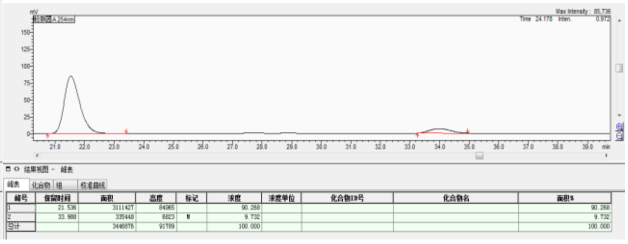

(*R*)-5-methyl-7-phenylhept-6-ynenitrile (4d)

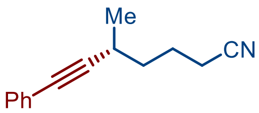

This compound was synthesized according to general procedure B. The residue was purified by column chromatography on silica gel (PE/EtOAc = 50:1) to afford the product **4d** (28.0 mg, 71% yield, 95% ee, rr = 85:15) as a yellow oil. **^{1}H NMR** (400 MHz, Chloroform-*d*) δ 7.41–7.35 (m, 2H), 7.31–7.27 (m, 3H), 2.77–2.64 (m, 1H), 2.42 (t, *J* = 7.1 Hz, 2H), 2.03–1.91 (m, 1H), 1.89–1.79 (m, 1H), 1.73–1.60 (m, 3H), 1.29 (d, *J* = 6.9 Hz, 3H). **^{13}C NMR** (100 MHz, Chloroform-*d*) δ 131.7, 128.4, 127.9, 123.7, 119.8, 93.1, 81.8, 35.8, 26.2, 23.6, 21.3, 17.2. **HRMS (ESI)** m/z calculated for $C_{14}H_{15}N$ [M+H]$^{+}$ 198.1277. found: 198.1285. **Optical rotation**: $[\alpha]^{20}_{D}$ = 10.93 (c = 1.0 g/L, $CHCl_3$). The absolute configuration was assigned by analogy to that of **3e'**. **HPLC condition**: Chiral column IE, *n*-hexane/*i*-PrOH = 97:3, flow rate = 0.6 mL/min, wavelength = 254 nm, tR = 12.1 min for major isomer, tR = 13.5 min for minor isomer.

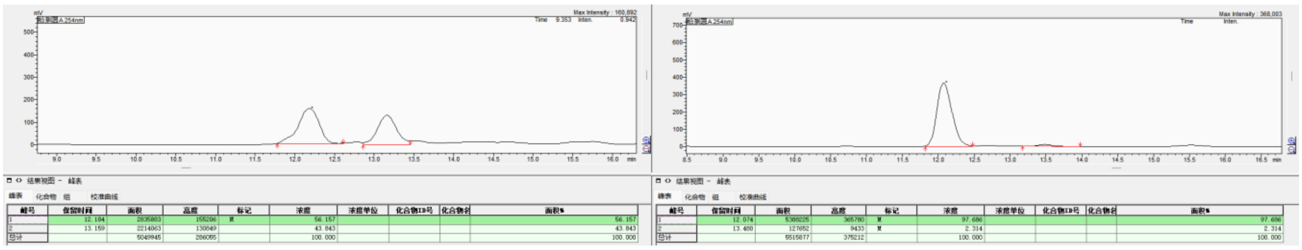

(*R*)-5-methyl-3,3,7-triphenylhept-6-ynenitrile (4e)

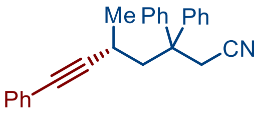

This compound was synthesized according to general procedure B. The residue was purified by column chromatography on silica gel (PE/EtOAc = 20:1) to afford the product **4e** (41.9 mg, 60% yield, 94% ee, rr = 92:8) as a colorless oil. **^{1}H NMR** (600 MHz, Chloroform-*d*) δ 7.40 – 7.33 (m, 4H), 7.30 – 7.22 (m, 8H), 7.19 – 7.15 (m, 3H), 2.77 (dd, *J* = 13.7, 8.3 Hz, 1H), 2.73 – 2.65 (m, 1H), 2.44 (dd, *J* = 13.7, 4.6 Hz, 1H), 1.25 (d, *J* = 6.8 Hz, 3H). **^{13}C NMR** (150 MHz, Chloroform-*d*) δ 140.4, 140.2, 131.7, 129.1, 129.0, 128.2, 128.16, 128.14, 127.8, 127.3, 127.1, 123.7, 122.3, 92.7, 82.4, 50.7, 45.8, 24.1, 22.6. **HRMS (ESI)** m/z calculated for $C_{16}H_{23}Na$ [M+Na]$^{+}$ 372.1723. found: 372.1726. **Optical rotation**: $[\alpha]^{20}_{D}$ = 8.57 (c = 1.0 g/L, $CHCl_3$). The absolute configuration was assigned by analogy to that of **3e'**. **HPLC condition**: Chiral column OD-H, *n*-hexane/*i*-PrOH = 98:2, flow rate = 1.0 mL/min, wavelength = 254 nm, tR = 7.3 min for major isomer, tR = 6.6 min for minor isomer.

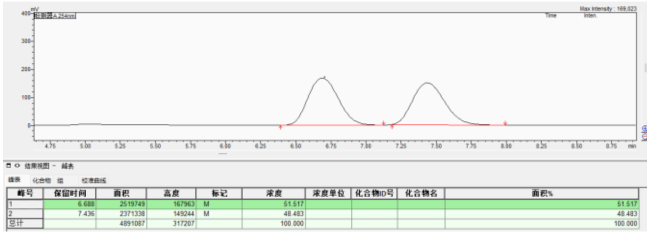
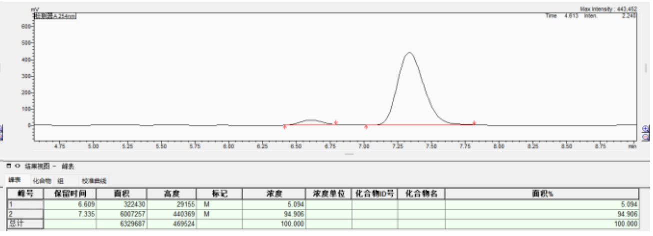

(*R*)-2-benzyl-2-(3-methyl-5-phenylpent-4-yn-1-yl)malononitrile (4f)

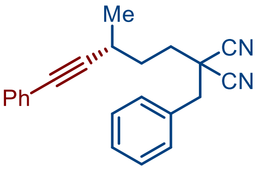

This compound was synthesized according to general procedure B. The residue was purified by column chromatography on silica gel (PE/EtOAc = 20:1) to afford the product **4f** (50.6 mg, 81% yield, 91% ee, rr = 95:5) as a yellow oil. **^{1}H NMR** (400 MHz, Chloroform-*d*) δ 7.38 (d, *J* = 9.2 Hz, 8H), 7.33–7.28 (m, 2H), 3.24 (s, 2H), 2.77 (h, *J* = 6.7 Hz, 1H), 2.32 (td, *J* = 12.8, 4.7 Hz, 1H), 2.09 (td, *J* = 12.9, 4.5 Hz, 1H), 2.01–1.84 (m, 2H), 1.34 (d, *J* = 6.9 Hz, 3H). **^{13}C NMR** (100 MHz, Chloroform-*d*) δ 132.1, 131.7, 130.4, 129.1, 128.9, 128.4, 128.1, 123.3, 115.4, 115.3, 92.1, 82.5, 43.5, 39.3, 35.7, 32.8, 26.3, 21.2. **HRMS (ESI)** m/z calculated for $C_{22}H_{20}N_2$ $[M+H]^+$ 313.1699. found: 313.1700. **Optical rotation**: $[\alpha]^{20}_D$ = 16.00 (c = 1.0 g/L, $CHCl_3$). The absolute configuration was assigned by analogy to that of **3e'**. **HPLC condition**: Chiral column OD-H, *n*-hexane/*i*-PrOH = 98:2, flow rate = 0.8 mL/min, wavelength = 254 nm, tR = 32.7 min for major isomer, tR = 48.4 min for minor isomer.

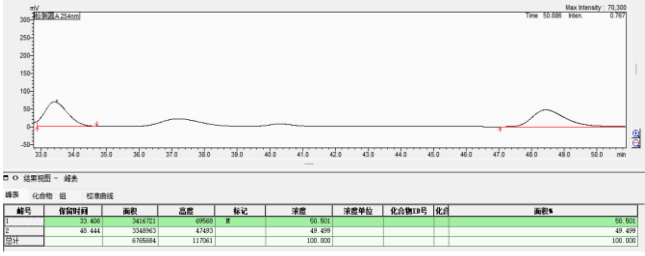
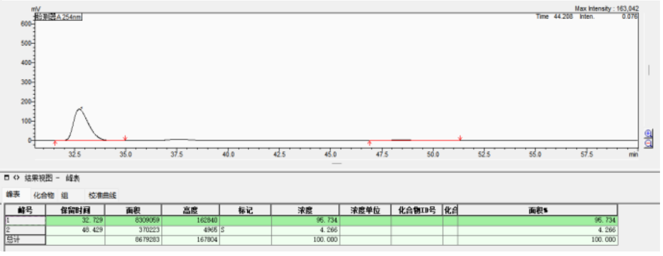

(*R*)-2-(3-methyl-5-phenylpent-4-yn-1-yl)-2-(4-methylbenzyl)malononitrile (4g)

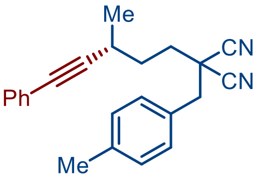

This compound was synthesized according to general procedure B. The residue was purified by column chromatography on silica gel (PE/EtOAc = 18:1) to afford the product **4g** (40.4 mg, 62% yield, 87% ee, rr = 93:7) as a yellow oil. **^{1}H NMR** (400 MHz, Chloroform-*d*) δ 7.43–7.38 (m, 2H), 7.36–7.32 (m, 3H), 7.30 (d, *J* = 1.6 Hz, 2H), 7.23 (d, *J* = 8.0 Hz, 2H), 3.25 (s, 2H), 2.86–2.76 (m, 1H), 2.39 (s, 3H), 2.36–

2.30 (m, 1H), 2.11 (td, J = 13.3, 12.8, 4.4 Hz, 1H), 2.02–1.89 (m, 2H), 1.38 (d, J = 6.9 Hz, 3H). **^{13}C NMR** (100 MHz, Chloroform-*d*) δ 138.7, 131.7, 130.1, 129.7, 128.9, 128.3, 128.0, 123.3, 115.4, 115.3, 92.0, 82.4, 43.0, 39.3, 35.5, 32.7, 26.2, 21.2, 21.1. **HRMS (ESI)** m/z calculated for $C_{23}H_{22}N_2$ $[M+H]^+$ 327.1856; found: 327.1856. **Optical rotation**: $[\alpha]^{20}_D$ = 15.53 (c = 1.0 g/L, $CHCl_3$). The absolute configuration was assigned by analogy to that of **3e'**. **HPLC condition**: Chiral column AD-H, *n*-hexane/*i*-PrOH = 99:1, flow rate = 0.3 mL/min, wavelength = 254 nm, tR = 34.4 min for major isomer, tR = 36.7 min for minor isomer.

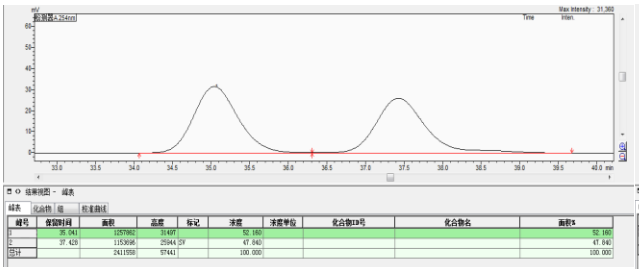

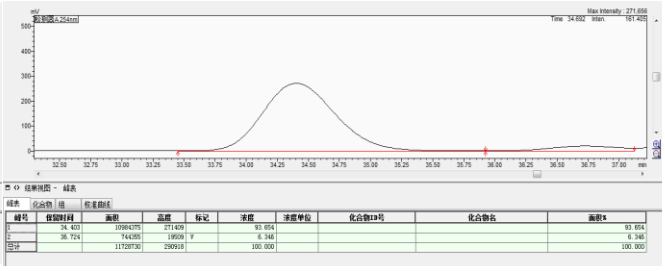

(*R*)-2-(4-chlorobenzyl)-2-(3-methyl-5-phenylpent-4-yn-1-yl)malononitrile (4h)

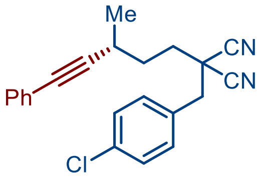

This compound was synthesized according to general procedure B. The residue was purified by column chromatography on silica gel (PE/EtOAc = 25:1) to afford the product **4h** (54.7 mg, 79% yield, 92% ee, rr = 92:8) as a yellow oil. **^{1}H NMR** (400 MHz, Chloroform-*d*) δ 7.39–7.29 (m, 9H), 3.21 (s, 2H), 2.83–2.72 (m, 1H), 2.31 (td, J = 12.9, 4.7 Hz, 1H), 2.08 (td, J = 13.0, 4.5 Hz, 1H), 2.01–1.84 (m, 2H), 1.34 (d, J = 6.9 Hz, 3H). **^{13}C NMR** (100 MHz, Chloroform-*d*) δ 135.2, 131.71, 131.67, 130.5, 129.4, 128.4, 128.2, 123.3, 115.2, 115.0, 91.9, 82.6, 42.8, 39.2, 35.7, 32.7, 26.3, 21.2. **HRMS (ESI)** m/z calculated for $C_{22}H_{19}ClN_2$ $[M+H]^+$ 347.1310; found: 347.1312. **Optical rotation**: $[\alpha]^{20}_D$ =19.97 (c = 1.0 g/L, $CHCl_3$). The absolute configuration was assigned by analogy to that of **3e'**. **HPLC condition**: Chiral column AD-H, *n*-hexane/*i*-PrOH = 98:2, flow rate = 1.0 mL/min, wavelength = 254 nm, tR = 10.2 min for major isomer, tR = 10.9 min for minor isomer.

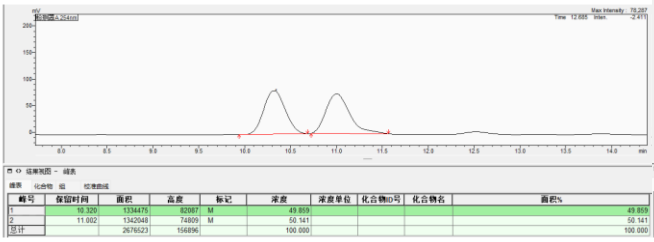

峰号	保留时间	面积	高度	标记	浓度	浓度单位	化合物ID号	化合物名	面积%
1	10.320	1334475	82087	M	49.859				49.859
2	11.002	1342048	74809	M	50.141				50.141
总计		2676523	156896		100.000				100.000

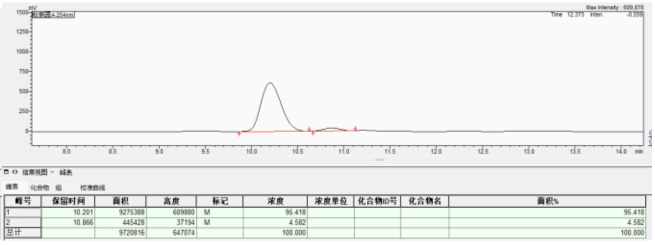

峰号	保留时间	面积	高度	标记	浓度	浓度单位	化合物ID号	化合物名	面积%
1	10.201	9275388	609880	M	95.418				95.418
2	10.866	445428	37194	M	4.582				4.582
总计		9720816	647074		100.000				100.000

(*R*)-2-(3-bromobenzyl)-2-(3-methyl-5-phenylpent-4-yn-1-yl)malononitrile (4i)

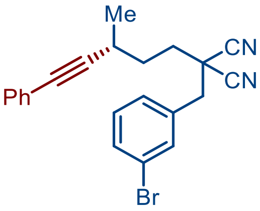

This compound was synthesized according to general procedure B. The residue was purified by column chromatography on silica gel (PE/EtOAc = 15:1) to afford the product **4i** (66.3 mg, 62% yield, 82% ee, rr = 97:3) as a green oil. **^{1}H NMR** (400 MHz, Chloroform-*d*) δ 7.60–7.57 (m, 2H), 7.42–7.30 (m, 7H), 3.24 (d, *J* = 1.4 Hz, 2H), 2.86–2.78 (m, 1H), 2.37 (td, *J* = 13.1, 4.6 Hz, 1H), 2.13 (td, *J* = 12.9, 4.4 Hz, 1H), 2.04–1.91 (m, 2H), 1.39 (d, *J* = 6.9 Hz, 3H). **^{13}C NMR** (100 MHz, Chloroform-*d*) δ 134.2, 133.3, 132.2, 131.7, 130.7, 129.0, 128.4, 128.1, 123.3, 123.0, 115.1, 114.9, 91.9, 82.6, 42.9, 39.2, 35.9, 32.8, 26.3, 21.2. **HRMS (ESI)** m/z calculated for $C_{22}H_{19}BrN_2$ $[M+H]^+$ 391.0805; found: 391.0806. **Optical rotation**: $[\alpha]^{20}_{D}$ = -21.97 (c = 1.0 g/L, $CHCl_3$). The absolute configuration was assigned by analogy to that of **3e'**. **HPLC condition**: Chiral column OD-H, *n*-hexane/*i*-PrOH = 95:5, flow rate = 1.0 mL/min, wavelength = 254 nm, tR = 20.5 min for major isomer, tR = 21.8 min for minor isomer.

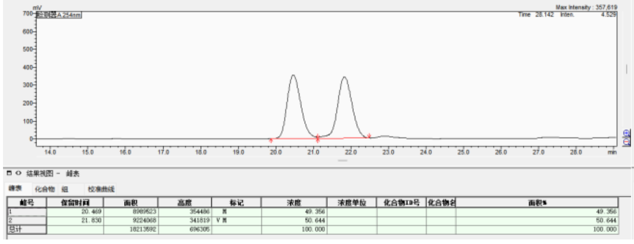

峰号	保留时间	面积	高度	标记	浓度	浓度单位	化合物ID号	化合物名	面积%
1	20.469	8989523	354486	M	49.356				49.356
2	21.830	9224068	341819	V M	50.644				50.644
总计		18213592	696305		100.000				100.000

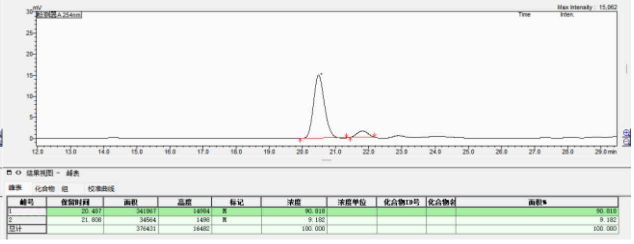

峰号	保留时间	面积	高度	标记	浓度	浓度单位	化合物ID号	化合物名	面积%
1	20.487	341867	14984	M	90.818				90.818
2	21.808	34564	1498	M	9.182				9.182
总计		376431	16482		100.000				100.000

(*R*)-2-(3-chloro-4-fluorobenzyl)-2-(3-methyl-5-phenylpent-4-yn-1-yl)malononitrile (4j)

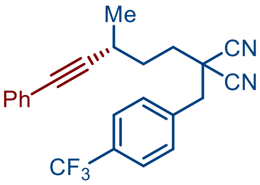

This compound was synthesized according to general procedure B. The residue was purified by column chromatography on silica gel (PE/EtOAc = 20:1) to afford the product **4j** (64.6 mg, 85% yield, 91% ee, rr = 94:6) as a yellow oil.

^{1}H NMR (400 MHz, Chloroform-*d*) δ 7.69 (d, *J* = 8.0 Hz, 2H), 7.57 (d, *J* = 7.9 Hz, 2H), 7.42–7.38 (m, 2H), 7.36–7.33 (m, 3H), 3.33 (s, 2H), 2.88–2.78 (m, 1H), 2.39 (td, *J* = 12.9, 4.6 Hz, 1H), 2.15 (td, *J* = 12.9, 4.4 Hz, 1H), 2.07–1.86 (m, 2H), 1.39 (d, *J* = 6.9 Hz, 3H). **^{13}C NMR** (100 MHz, Chloroform-*d*) δ 136.0, 134.3 (q, $J_{C\text{-}F}$ = 77.5 Hz), 131.7, 130.81, 130.84, 128.4, 128.2, 126.1 (q, $J_{C\text{-}F}$ = 3.8 Hz), 124.3 (q, $J_{C\text{-}F}$ = 240.9 Hz), 115.0, 114.9, 91.9, 82.6, 43.0, 39.1, 35.9, 32.8, 26.3, 21.2. **^{19}F NMR** (376 MHz, Chloroform-*d*) δ -62.8. **HRMS (ESI)** m/z calculated for $C_{23}H_{19}F_3N_2$ $[M+H]^+$ 381.1573; found: 381.1574. **Optical rotation**: $[\alpha]^{20}_D$ = 10.17 (c = 1.0 g/L, $CHCl_3$). The absolute configuration was assigned by analogy to that of **3e'**. **HPLC condition**: Chiral column AD-H, *n*-hexane/*i*-PrOH = 97:3, flow rate = 0.6 mL/min, wavelength = 254 nm, tR = 13.5 min for major isomer, tR = 14.1 min for minor isomer.

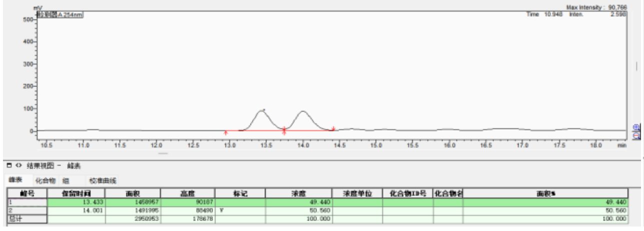

峰号	保留时间	面积	高度	标记	浓度	浓度单位	化合物ID号	化合物名	面积%
1	13.433	1458957	90187		49.440				49.440
2	14.001	1491995	88490	V	50.560				50.560
总计		2950953	178678		100.000				100.000

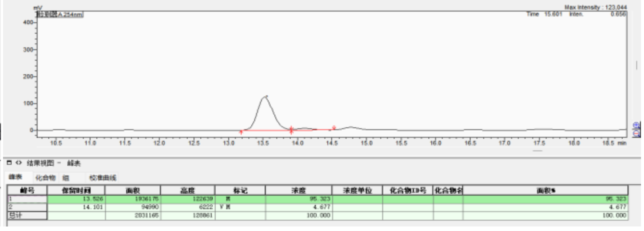

峰号	保留时间	面积	高度	标记	浓度	浓度单位	化合物ID号	化合物名	面积%
1	13.526	1936175	122639	M	95.323				95.323
2	14.101	94990	6222	V [illegible]	4.677				4.677
总计		2031165	128861		100.000				100.000

(*R*)-2-(3-chloro-4-fluorobenzyl)-2-(3-methyl-5-phenylpent-4-yn-1-yl)malononitrile (4k)

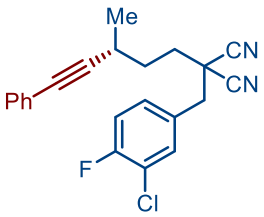

This compound was synthesized according to general procedure B. The residue was purified by column chromatography on silica gel (PE/EtOAc = 25:1) to afford the product **4k** (60.4 mg, 83% yield, 92% ee, rr = 95:5) as a bright colorless oil.

^{1}H NMR (400 MHz, Chloroform-*d*) δ 7.45 (dd, *J* = 6.8, 2.3 Hz, 1H), 7.40–7.36 (m, 2H), 7.33–7.27 (m, 4H), 7.16 (t, *J* = 8.6 Hz, 1H), 3.19 (d, *J* = 1.1 Hz, 2H), 2.83–2.76 (m, 1H), 2.33 (ddd, *J* = 13.5, 12.2, 4.6 Hz, 1H), 2.10 (ddd, *J* = 13.6, 12.3, 4.5 Hz, 1H), 2.01–1.85 (m, 2H), 1.35 (d, *J* = 6.9 Hz, 3H). **^{13}C NMR** (100 MHz, Chloroform-*d*) δ 158.6 (d, $J_{C\text{-}F}$= 251.2 Hz), 132.5, 131.7, 130.2 (d, $J_{C\text{-}F}$ = 7.5 Hz), 129.2 (d, $J_{C\text{-}F}$ = 4.0 Hz), 128.4, 128.2, 123.2, 121.9 (d, $J_{C\text{-}F}$ = 18.2 Hz), 117.3 (d, $J_{C\text{-}F}$ = 21.3 Hz), 114.9 (d, $J_{C\text{-}F}$ = 16.4 Hz), 91.9, 82.6, 42.4, 39.3, 35.9, 32.8, 26.3, 21.2. **^{19}F NMR** (376 MHz, Chloroform-*d*) δ -114.6. **HRMS (ESI)** m/z calculated for $C_{22}H_{18}FClN_2$ $[M+H]^+$ 365.1216; found: 365.1215. **Optical rotation**: $[\alpha]^{20}_D$ =-16.77 (c = 1.0 g/L, $CHCl_3$). The absolute configuration was

assigned by analogy to that of **3e’**. **HPLC condition**: Chiral column AD-H, *n*-hexane/*i*-PrOH = 97:3, flow rate = 1.0 mL/min, wavelength = 254 nm, tR = 8.9 min for major isomer, tR = 9.4 min for minor isomer.

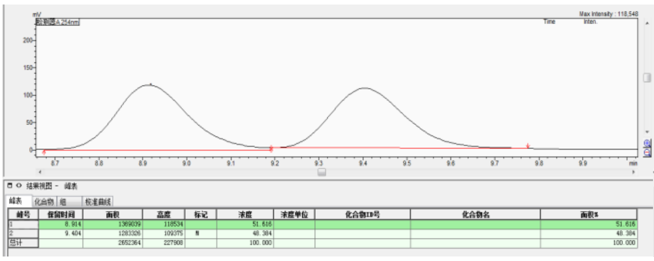

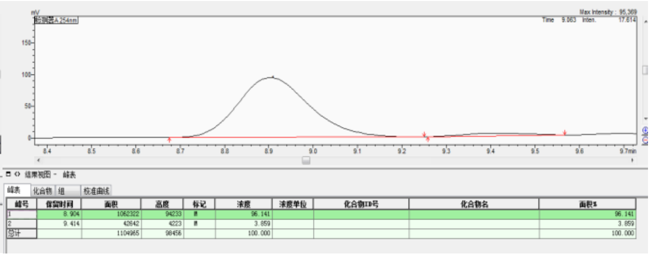

(*R*)-2-(3-methyl-5-phenylpent-4-yn-1-yl)-2-(naphthalen-2-ylmethyl)malononitrile (4l)

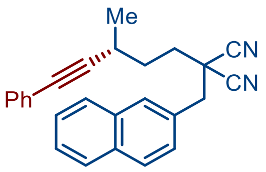

This compound was synthesized according to general procedure B. The residue was purified by column chromatography on silica gel (PE/EtOAc = 20:1) to afford the product **4l** (57.9 mg, 80% yield, 90% ee, rr = 95:5) as a yellow oil. **^{1}H NMR** (400 MHz, Chloroform-*d*) δ 7.89–7.83 (m, 5H), 7.56–7.48 (m, 3H), 7.33–7.27 (m, 4H), 3.42 (s, 2H), 2.84–2.74 (m, 1H), 2.37 (ddd, *J* = 13.6, 12.2, 4.7 Hz, 1H), 2.13 (td, *J* = 12.9, 4.5 Hz, 1H), 2.04–1.88 (m, 2H), 1.34 (d, *J* = 6.9 Hz, 3H). **^{13}C NMR** (100 MHz, Chloroform-*d*) δ 133.33, 133.28, 131.7, 129.8, 129.5, 128.9, 128.4, 128.2, 128.1, 127.9, 127.6, 126.8, 126.7, 123.3, 115.5, 115.3, 92.0, 82.5, 43.6, 39.3, 35.8, 32.8, 26.3, 21.2. **HRMS (ESI)** m/z calculated for $C_{26}H_{22}N_2$ $[M+H]^+$ 363.1856; found: 363.1855. **Optical rotation**: $[\alpha]^{20}_{D}$ = 20.73 (c = 1.0 g/L, $CHCl_3$). The absolute configuration was assigned by analogy to that of **3e’**. **HPLC condition**: Chiral column AD-H, *n*-hexane/*i*-PrOH = 98:2, flow rate = 1.0 mL/min, wavelength = 254 nm, tR = 12.0 min for major isomer, tR = 12.8 min for minor isomer.

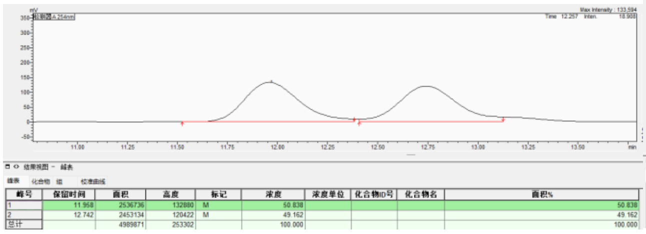

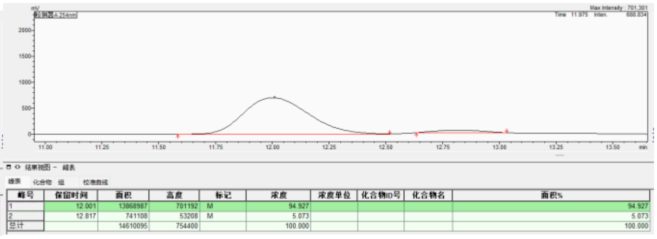

(*R*)-2-(3-methyl-5-phenylpent-4-yn-1-yl)-2-(thiophen-2-ylmethyl)malononitrile (4m)

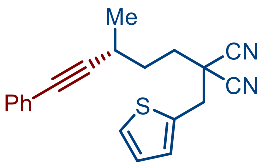

This compound was synthesized according to general procedure B. The residue was purified by column chromatography on silica gel (PE/EtOAc = 25:1) to afford the product **4m** (47.7mg, 75% yield, 87% ee, rr = 93:7) as a colorless oil. **^{1}H NMR** (400 MHz, Chloroform-*d*) δ 7.42–7.33 (m, 2H), 7.34–7.27 (m, 4H), 7.17 (d, *J* = 2.4 Hz, 1H), 7.03 (dd, *J* = 5.2, 3.5 Hz, 1H), 3.50 (s, 2H), 2.84–2.71 (m, 1H), 2.34 (ddd, *J* = 13.5, 12.1, 4.7 Hz, 1H), 2.10 (ddd, *J* = 13.7, 12.4, 4.5 Hz, 1H), 2.00–1.86 (m, 2H), 1.34 (d, *J* = 6.9 Hz, 3H). **^{13}C NMR** (100 MHz, Chloroform-*d*) δ 132.8, 131.8, 129.3, 128.4, 128.1, 127.7, 126.8, 123.3, 115.3, 115.2, 92.0, 82.5, 39.4, 37.7, 35.3, 32.7, 26.3, 21.2. **HRMS (ESI)** m/z calculated for $C_{20}H_{18}N_2S$ $[M+H]^+$ 319.1264; found: 319.1266. **Optical rotation**: $[\alpha]^{20}_{D}$ = 9.00 (c = 1.0 g/L, $CHCl_3$). The absolute configuration was assigned by analogy to that of **3e'**. **HPLC condition**: Chiral column AD-H, *n*-hexane/*i*-PrOH = 95:5, flow rate = 1.0 mL/min, wavelength = 254 nm, tR = 19.0 min for major isomer, tR = 20.2 min for minor isomer.

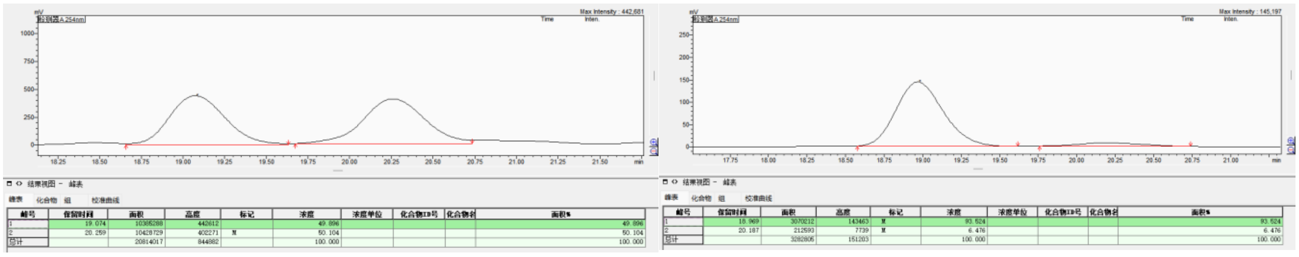

(*R*)-2-(cyclohexylmethyl)-2-(3-methyl-5-phenylpent-4-yn-1-yl)malononitrile (4n)

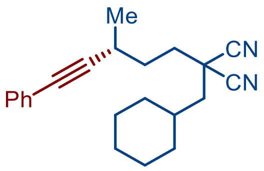

This compound was synthesized according to general procedure B. The residue was purified by column chromatography on silica gel (PE/EtOAc = 15:1) to afford the product **4n** (45.5 mg, 73% yield, 91% ee, rr = 95:5) as a yellow oil. **^{1}H NMR** (400 MHz, Chloroform-*d*) δ 7.42–7.37 (m, 2H), 7.32–7.28 (m, 3H), 2.82–2.70 (m, 1H), 2.33–2.22 (m, 1H), 2.05 (td, *J* = 13.2, 12.7, 4.4 Hz, 1H), 1.98–1.83 (m, 7H), 1.81–1.65 (m, 8H), 1.34 (d, *J* = 6.9 Hz, 3H). **^{13}C NMR** (100 MHz, Chloroform-*d*) δ 131.7, 128.4, 128.1, 123.4, 116.2, 116.0, 92.1, 82.4, 44.8, 37.4, 35.8, 35.7, 33.5, 32.6, 26.3, 26.0, 25.9, 21.2. **HRMS (ESI)** m/z calculated for $C_{22}H_{26}N_2$ $[M+H]^+$ 319.2169; found: 319.2166. **Optical rotation**: $[\alpha]^{20}_{D}$ =22.23 (c = 1.0 g/L, $CHCl_3$). The absolute configuration was assigned by analogy to that of **3e'**. **HPLC condition**:

Chiral column AD-H, *n*-hexane/*i*-PrOH = 95:5, flow rate = 1.0 mL/min, wavelength = 254 nm, tR = 10.3 min for major isomer, tR = 10.8 min for minor isomer.

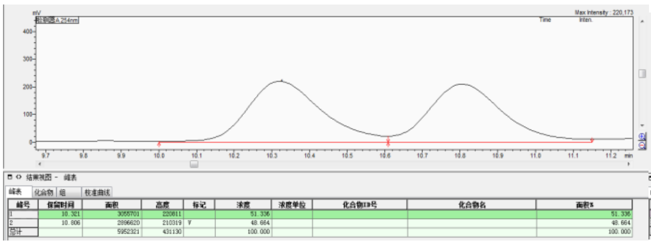

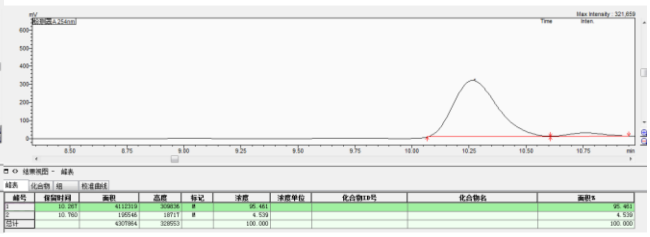

(*R*)-2-(3-chloropropyl)-2-(3-methyl-5-phenylpent-4-yn-1-yl)malononitrile (4o)

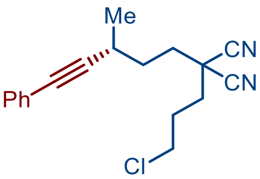

This compound was synthesized according to general procedure B. The residue was purified by column chromatography on silica gel (PE/EtOAc = 25:1) to afford the product **4o** (53.1 mg, 85% yield, 82% ee, rr = 91:9) as a yellow oil.

^{1}H NMR (400 MHz, Chloroform-*d*) δ 7.42–7.37 (m, 2H), 7.32–7.28 (m, 3H), 3.67–3.63 (m, 2H), 2.84–2.73 (m, 1H), 2.31 (ddd, *J* = 13.6, 12.1, 4.8 Hz, 1H), 2.24–2.14 (m, 4H), 2.10 (ddd, *J* = 13.6, 12.3, 4.6 Hz, 2H), 1.97–1.82 (m, 2H), 1.35 (d, *J* = 6.9 Hz, 3H). **^{13}C NMR** (100 MHz, Chloroform-*d*) δ 131.7, 128.4, 128.1, 123.3, 115.4, 115.3, 91.9, 82.5, 43.3, 37.2, 36.1, 35.5, 32.7, 28.5, 26.3, 21.2. **HRMS (ESI)** m/z calculated for $C_{17}H_{19}ClN_2$ $[M+H]^+$ 299.1310; found: 299.1313. **Optical rotation**: $[\alpha]^{20}_D$ = 27.63(c = 1.0 g/L, $CHCl_3$). The absolute configuration was assigned by analogy to that of **3e'**. **HPLC condition**: Chiral column AD-H, *n*-hexane/*i*-PrOH = 95:5, flow rate = 1.0 mL/min, wavelength = 254 nm, tR = 15.7 min for major isomer, tR = 16.5 min for minor isomer.

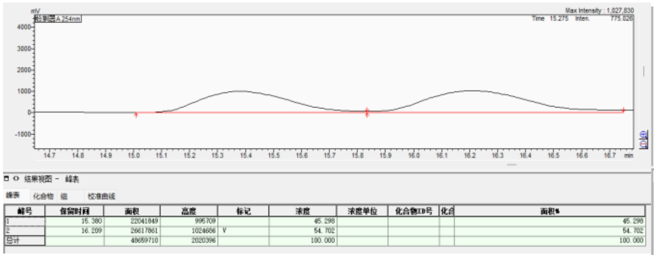

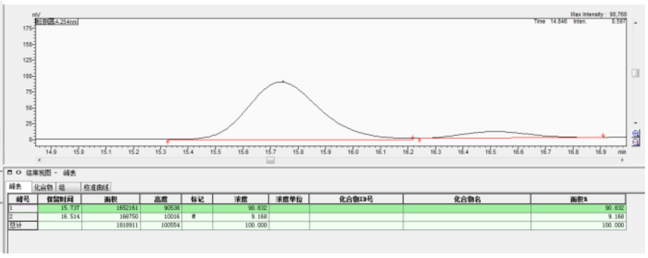

(*R*)-3-phenethyl-5-phenylpent-4-ynenitrile (4p)

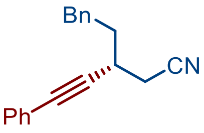

This compound was synthesized according to general procedure A. The residue was purified by column chromatography on silica gel (PE/EtOAc = 20:1) to afford the product **4p** (28.5 mg, 55% yield, 52% ee, rr = 90:10) as a green oil.

^{1}H NMR (400 MHz, Chloroform-*d*) δ 7.49–7.42 (m, 2H), 7.36–7.27 (m, 5H), 7.28–7.18 (m, 3H), 2.94 (dp, *J* = 13.7, 6.8 Hz, 2H), 2.87–2.78 (m, 1H), 2.63 (d, *J* = 6.5 Hz, 2H), 2.01 (q, *J* = 7.6 Hz, 2H). **^{13}C NMR** (100 MHz, Chloroform-*d*) δ 140.8, 131.9, 128.7, 128.6, 128.51, 128.46, 126.4, 122.8, 117.7, 88.4, 84.6, 36.1, 33.4, 28.9, 24.0. **HRMS (ESI)** m/z calculated for $C_{19}H_{17}N$ $[M+H]^+$ 260.1434; found 260.1440. **Optical rotation**: $[\alpha]^{20}_{D}$ = 17.60 (c = 1.0 g/L, $CHCl_3$). The absolute configuration was assigned by analogy to that of **3e'**. **HPLC condition**: Chiral column AD-H, *n*-hexane/*i*-PrOH = 99.5:0.5, flow rate = 0.8 mL/min, wavelength = 254 nm, tR = 32.4 min for major isomer, tR = 27.7 min for minor isomer.

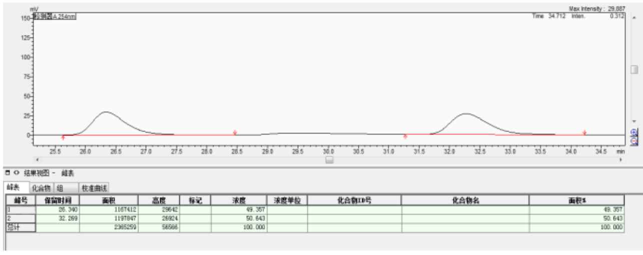

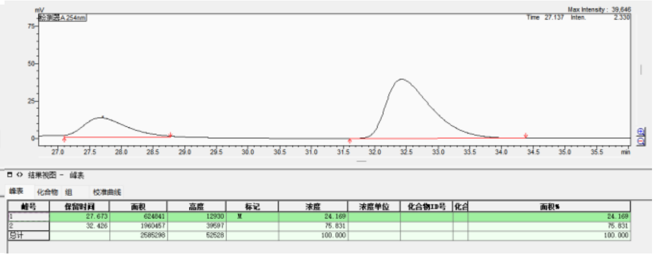

(*R*)-3-ethyl-5-phenylpent-4-ynenitrile (4q)

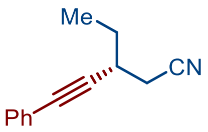

This compound was synthesized according to general procedure A. The residue was purified by column chromatography on silica gel (PE/EtOAc = 25:1) to afford the product **4q** (20.6 mg, 57% yield, 93% ee, rr = 95:5) as a colorless oil.

^{1}H NMR (600 MHz, Chloroform-*d*) δ 7.44 – 7.41 (m, 2H), 7.34 – 7.27 (m, 3H),, 2.94 – 2.85 (m, 1H), 2.67 – 2.59 (m, 2H), 1.83 – 1.74 (m, 1H), 1.74 – 1.66 (m, 1H), 1.12 (t, *J* = 7.3 Hz, 3H). **^{13}C NMR** (150 MHz, Chloroform-*d*) δ 131.9, 128.39, 128.37, 122.9, 117.9, 88.7, 84.0, 31.1, 27.6, 23.6, 11.6. **HRMS (ESI)** m/z calculated for $C_{13}H_{14}N$ $[M+H]^+$ 184.1121; found 184.1122. **Optical rotation**: $[\alpha]^{20}_{D}$ = 10.35 (c = 1.0 g/L, $CHCl_3$). The absolute configuration was assigned by analogy to that of **3e'**. **HPLC condition**: Chiral column OD-H, *n*-hexane/*i*-PrOH = 98:2, flow rate = 1.0 mL/min, wavelength = 254 nm, tR = 13.4 min for major isomer, tR = 10.6 min for minor isomer.

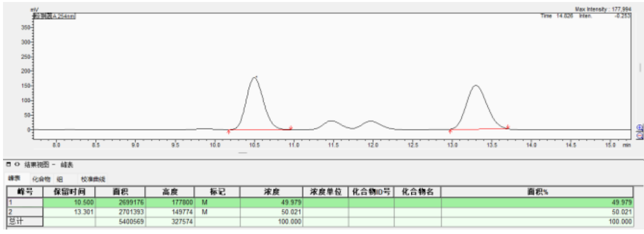

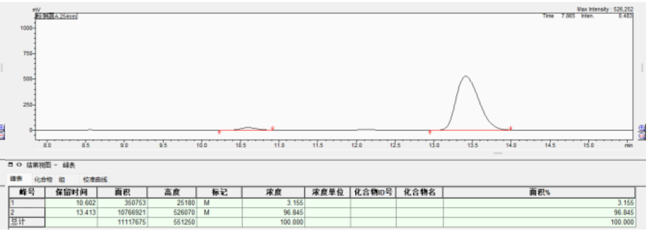

(*R*)-6-cyano-5-methylhex-3-yn-1-yl 2-(3-cyano-4-isobutoxyphenyl)-4-methylthiazole-5-carboxylate (5a)

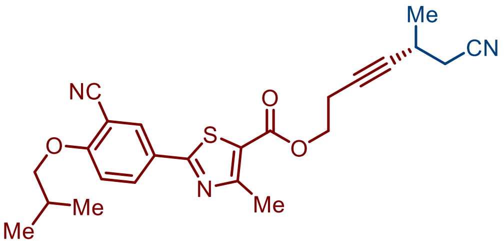

This compound was synthesized according to general procedure A. The residue was purified by column chromatography on silica gel (PE/EtOAc = 12:1) to afford the product **5a** (67.0 mg, 77% yield, 93% ee, rr > 20:1) as a green oil.

^{1}H NMR (400 MHz, Chloroform-*d*) δ 8.18 (d, *J* = 2.3 Hz, 1H), 8.09 (dd, *J* = 8.9, 2.3 Hz, 1H), 7.01 (d, *J* = 8.7 Hz, 1H), 4.36 (t, *J* = 6.7 Hz, 2H), 3.89 (d, *J* = 6.5 Hz, 2H), 2.88–2.79 (m, 1H), 2.76 (s, 3H), 2.63 (td, *J* = 6.8, 2.1 Hz, 2H), 2.49 (dd, *J* = 6.6, 1.5 Hz, 2H), 2.26–2.14 (m, 1H), 1.31 (d, *J* = 7.0 Hz, 3H), 1.08 (d, *J* = 6.7 Hz, 6H). **^{13}C NMR** (100 MHz, Chloroform-*d*) δ 167.7, 162.7, 161.9, 161.6, 132.7, 132.2, 126.0, 121.6, 117.8, 115.5, 112.7, 103.1, 82.7, 78.4, 75.8, 63.2, 28.3, 25.5, 23.7, 20.7, 19.4, 19.2, 17.7. **HRMS (ESI)** m/z calculated for $C_{24}H_{25}N_3O_3S$ $[M+H]^+$ 436.1695; found: 436.1692. **Optical rotation**: $[\alpha]^{20}_D$ =31.19 (c = 1.0 g/L, $CHCl_3$). The absolute configuration was assigned by analogy to that of **3e'**. **HPLC condition**: Chiral column AD-H, *n*-hexane/*i*-PrOH = 90:10, flow rate = 1.0 mL/min, wavelength = 254 nm, tR = 35.0 min for major isomer, tR = 39.1 min for minor isomer.

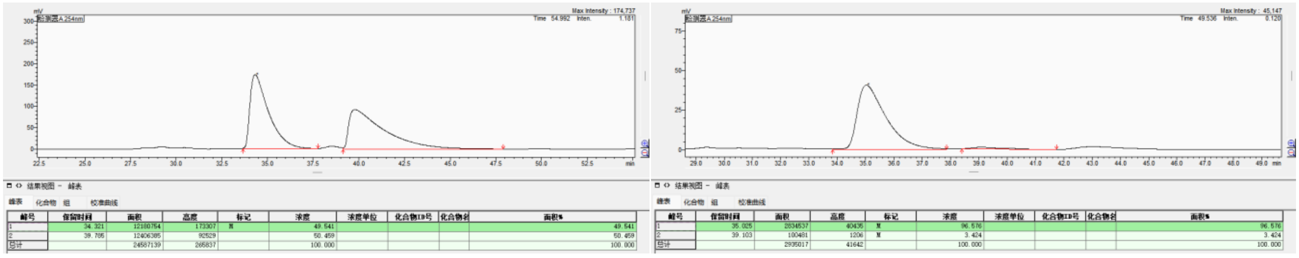

6-cyano-5-methylhex-3-yn-1-yl 4-(N,N-diisopropylsulfamoyl)benzoate (5b)

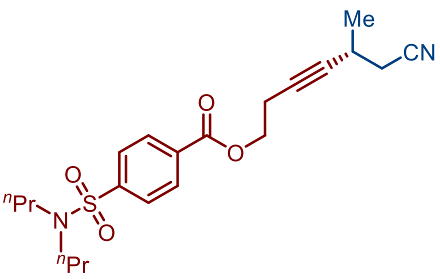

This compound was synthesized according to general procedure A. The residue was purified by column chromatography on silica gel (PE/EtOAc = 18:1) to afford the product **5b** (60.6 mg, 75% yield, 94% ee, rr > 20:1) as a white solid.

^{1}H NMR (400 MHz, Chloroform-*d*) δ 8.17 (d, *J* = 8.4 Hz, 2H), 7.87 (d, *J* = 8.4 Hz, 2H), 4.42 (t, *J* = 6.8 Hz, 2H), 3.18–2.97 (m, 4H), 2.86–2.76 (m, 1H), 2.66 (td, *J* =

6.8, 2.1 Hz, 2H), 2.47 (d, J = 6.5 Hz, 1H), 1.54 (h, J = 7.5 Hz, 4H), 1.29 (d, J = 6.9 Hz, 3H), 0.86 (t, J = 7.4 Hz, 6H). **^{13}C NMR** (100 MHz, Chloroform-*d*) δ 165.1, 144.5, 133.4, 130.4, 127.1, 117.8, 82.7, 78.4, 63.5, 50.0, 25.5, 23.6, 22.0, 20.7, 19.4, 11.3. **HRMS (ESI)** m/z calculated for $C_{21}H_{28}N_2O_4S$ $[M+H]^+$ 405.1843; found: 405.1846. **Optical rotation**: $[\alpha]^{20}_D$ = 71.70 (c = 1.0 g/L, $CHCl_3$). The absolute configuration was assigned by analogy to that of **3e'**. **HPLC condition**: Chiral column IF, *n*-hexane/*i*-PrOH = 90:10, flow rate = 1.0 mL/min, wavelength = 254 nm, tR = 35.4 min for major isomer, tR = 41.8 min for minor isomer.

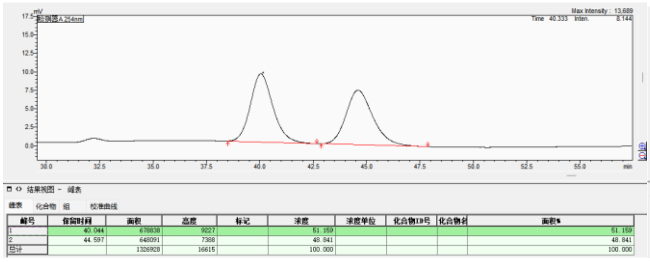

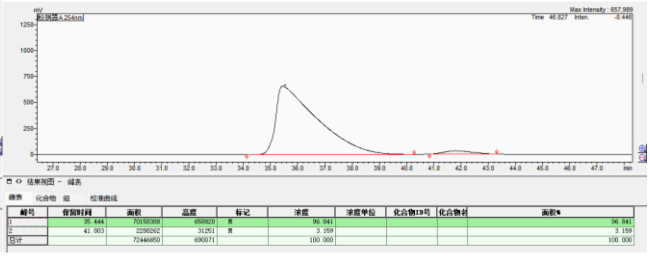

6-cyano-5-methylhex-3-yn-1-yl 11-oxo-6,11-dihydrodibenzo[b,e]oxepine-3-carboxylate (5c)

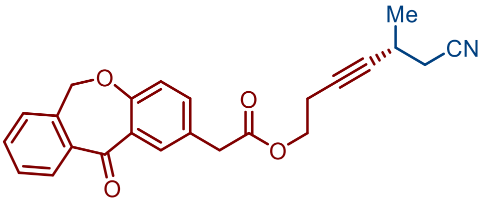

This compound was synthesized according to general procedure A. The residue was purified by column chromatography on silica gel (PE/EtOAc = 17:1) to afford the product **5c** (57.3 mg, 74% yield, 92% ee, rr > 20:1) as a colorless oil.

^{1}H NMR (400 MHz, Chloroform-*d*) δ 8.11 (s, 1H), 7.87 (d, J = 7.6 Hz, 1H), 7.57 – 7.52 (m, 1H), 7.49 – 7.41 (m, 2H), 7.35 (d, J = 7.1 Hz, 1H), 7.05 – 7.00 (m, 1H), 5.17 (s, 2H), 4.17 (t, J = 6.8 Hz, 2H), 3.66 (s, 2H), 2.84 – 2.73 (m, 1H), 2.54 – 2.48 (m, 2H), 2.47 – 2.42 (m, 2H), 1.27 (d, J = 7.0 Hz, 3H). **^{13}C NMR** (100 MHz, Chloroform-*d*) δ 190.9, 171.3, 160.5, 140.5, 136.5, 135.6, 132.9, 132.5, 129.5, 129.3, 127.9, 127.7, 125.2, 121.1, 117.8, 82.4, 78.5, 73.7, 62.9, 40.1, 25.4, 23.6, 20.6, 19.2. **HRMS (ESI)** m/z calculated for $C_{24}H_{21}NO_4$ $[M+H]^+$ 388.1544; found: 388.1545. **Optical rotation**: $[\alpha]^{20}_D$ = -66.67 (c = 1.0 g/L, $CHCl_3$). The absolute configuration was assigned by analogy to that of **3e'**. **HPLC condition**: Chiral column AD-H, *n*-hexane/*i*-PrOH = 97:3, flow rate = 0.6 mL/min, wavelength = 254 nm, tR = 234.9 min for major isomer, tR = 224.3 min for minor isomer.

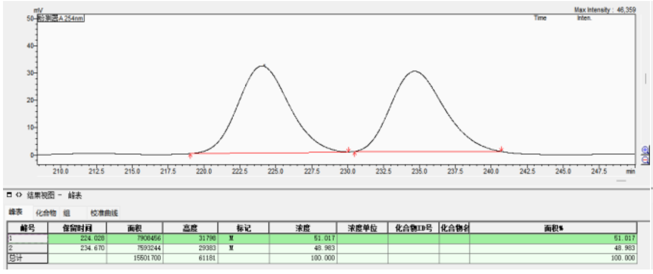

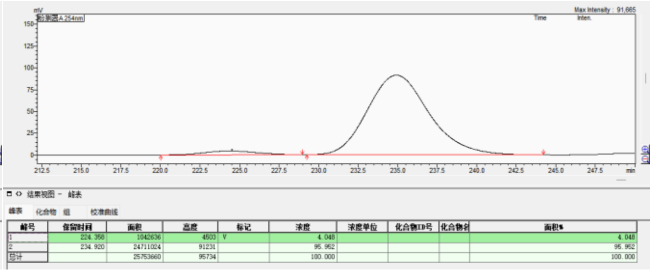

(*R*)-1-(4-cyano-3-methylbut-1-yn-1-yl)cyclohexyl 2-(1-(4-chlorobenzoyl)-5-methoxy-2-methyl-1H-indol-3-yl)acetate (5d)

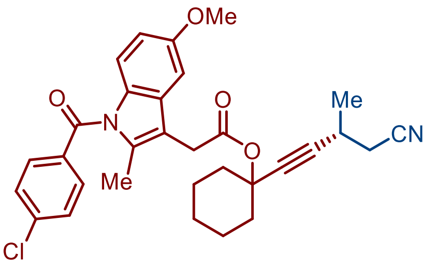

This compound was synthesized according to general procedure A. The residue was purified by column chromatography on silica gel (PE/EtOAc = 16:1) to afford the product **5d** (79.5 mg, 75% yield, 94% ee, rr > 20:1) as a colorless oil.

^{1}H NMR (400 MHz, Chloroform-*d*) δ 7.64 (d, *J* = 8.4 Hz, 2H), 7.46 (d, *J* = 8.4 Hz, 2H), 6.98 (d, *J* = 2.5 Hz, 1H), 6.89 (d, *J* = 9.0 Hz, 1H), 6.66 (dd, *J* = 9.0, 2.5 Hz, 1H), 3.83 (s, 3H), 3.62 (s, 2H), 2.83 (h, *J* = 6.8 Hz, 1H), 2.47 – 2.40 (m, 2H), 2.37 (s, 3H), 2.07 – 2.01 (m, 2H), 1.85 – 1.76 (m, 2H), 1.59 – 1.44 (m, 5H), 1.26 (d, *J* = 6.9 Hz, 4H). **^{13}C NMR** (100 MHz, Chloroform-*d*) δ 168.9, 168.3, 156.0, 139.2, 135.8, 134.0, 131.2, 130.9, 130.8, 129.1, 117.6, 114.9, 113.0, 111.5, 101.6, 87.0, 82.6, 76.2, 55.7, 37.1, 31.4, 25.2, 25.1, 23.6, 22.5, 20.2, 13.5. **HRMS (ESI)** m/z calculated for $C_{31}H_{31}ClN_2O_4$ $[M+H]^+$ 531.2045; found: 531.2048. **Optical rotation**: $[\alpha]^{20}_{D}$ = 75.83 (c = 1.0 g/L, $CHCl_3$). The absolute configuration was assigned by analogy to that of **3e'**. **HPLC condition:** Chiral column AD-H, *n*-hexane/*i*-PrOH = 97:3, flow rate = 1.0 mL/min, wavelength = 254 nm, tR = 52.3 min for major isomer, tR = 48.7 min for minor isomer.

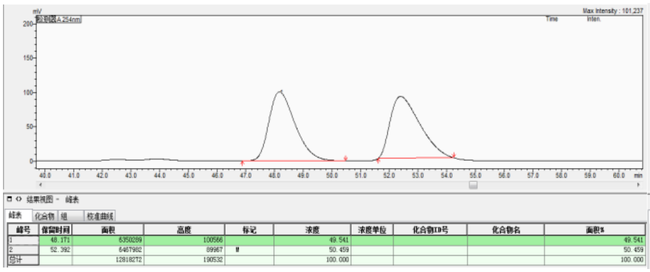

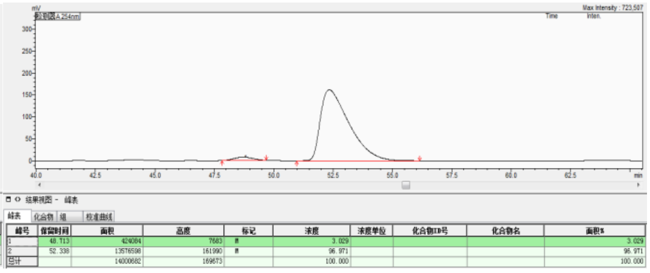

Unsuccessful substrates[a]

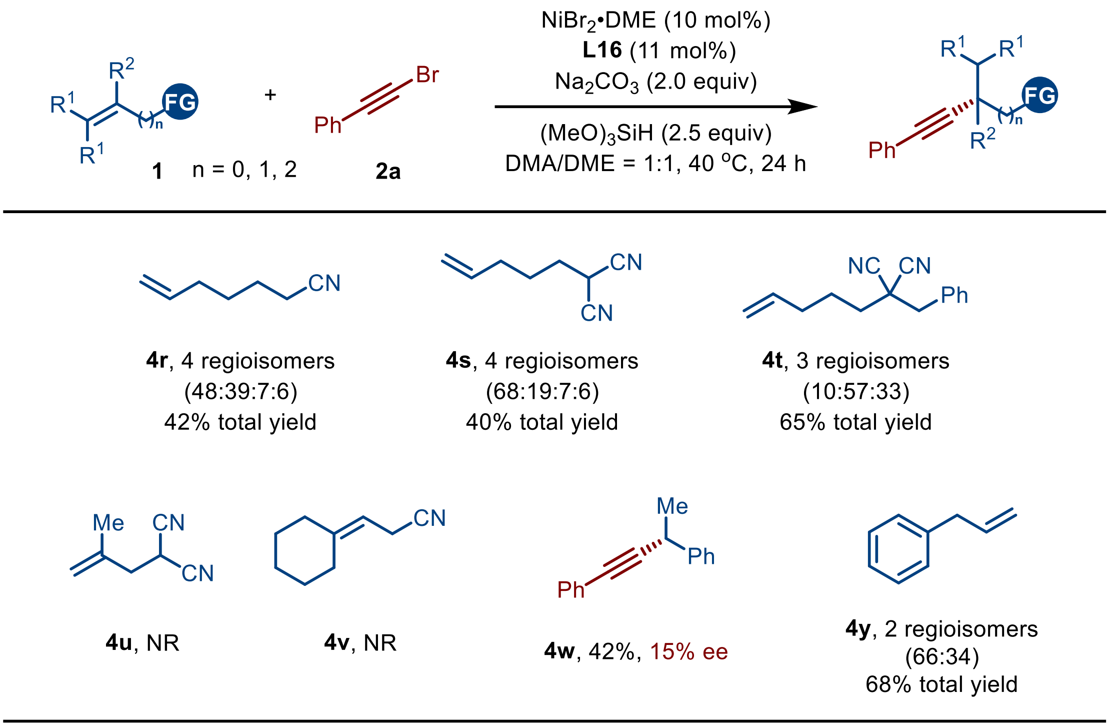

[a]Reactions were conducted on a 0.2 mmol scale in DMA/DME (1:1, 1 mL), isolated yield; rr represents the ratio of the desired product to the sum of all other isomers, as determined by GC-MS analysis of the crude reaction mixture.

In an argon-filled glovebox, $NiBr_2$•DME (6.2 mg, 0.02 mmol, 10.0 mol%), Ligand 16 (13.7 mg, 0.02 mmol, 11 mol%), relevant alkene substrate (0.2 mmol, 1.0 equiv), Na_2CO_3 (42.4 mg, 0.4 mmol, 2.0 equiv), DMA/DME (1:1, 1.0 mL) were added to a 4 mL reaction tube. Then relevant acetylene bromide (0.3 mmol, 1.5 equiv), $(MeO)_3SiH$ (64 μL, 0.5 mmol, 2.5 equiv) were added to the mixture. The reaction mixture was stirred at 40 °C for 24 h.

(*R*)-but-1-yne-1,3-diyldibenzene (4w)

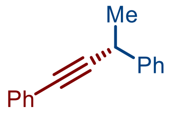

This compound was synthesized according to general procedure B. The residue was purified by column chromatography on silica gel (PE/EtOAc = 80:1) to afford the product **4w** (17.3 mg, 42% yield, 15% ee, rr > 20:1) as a colorless oil.

^{1}H NMR (600 MHz, Chloroform-*d*) δ 7.5 – 7.4 (m, 4H), 7.4 (t, *J* = 7.6 Hz, 2H), 7.3 – 7.3 (m, 4H), 4.0 (q, *J* = 7.1 Hz, 1H), 1.6 (d, *J* = 7.1 Hz, 3H). **^{13}C NMR** (150 MHz, Chloroform-*d*) δ 143.4, 131.7, 128.7, 128.3, 127.9, 127.0, 126.8, 123.8, 92.7, 82.5, 32.6, 24.6. **HRMS (ESI)** m/z calculated for $C_{16}H_{15}$

$[M+H]^+$ 207.1168; found: 207.1166. **Optical rotation**: $[\alpha]^{20}_D$ = -16.3 (c = 1.0 g/L, $CHCl_3$). The absolute configuration was assigned by analogy to that of **3e'**. **HPLC condition**: Chiral column OD-H, *n*-hexane/*i*-PrOH = 99.5:0.5, flow rate = 1.0 mL/min, wavelength = 254 nm, tR = 4.9 min for major isomer, tR = 5.2 min for minor isomer.

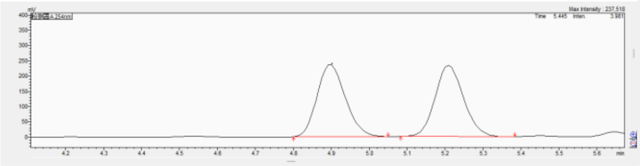

峰号	保留时间	面积	高度	标记	浓度	浓度单位	化合物ID号	化合物名	面积%
1	4.896	1227419	236370	M	50.187				50.187
2	5.208	1218293	233435	M	49.813				49.813
总计		2445711	469805		100.000				100.000

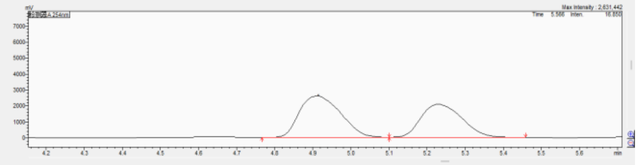

峰号	保留时间	面积	高度	标记	浓度	浓度单位	化合物ID号	化合物名	面积%
1	4.909	19522910	2601274	M	55.623				55.623
2	5.229	15575657	2068275	M	44.377				44.377
总计		35098567	4669549		100.000				100.000

5. Gram-scale reaction and product derivatizations

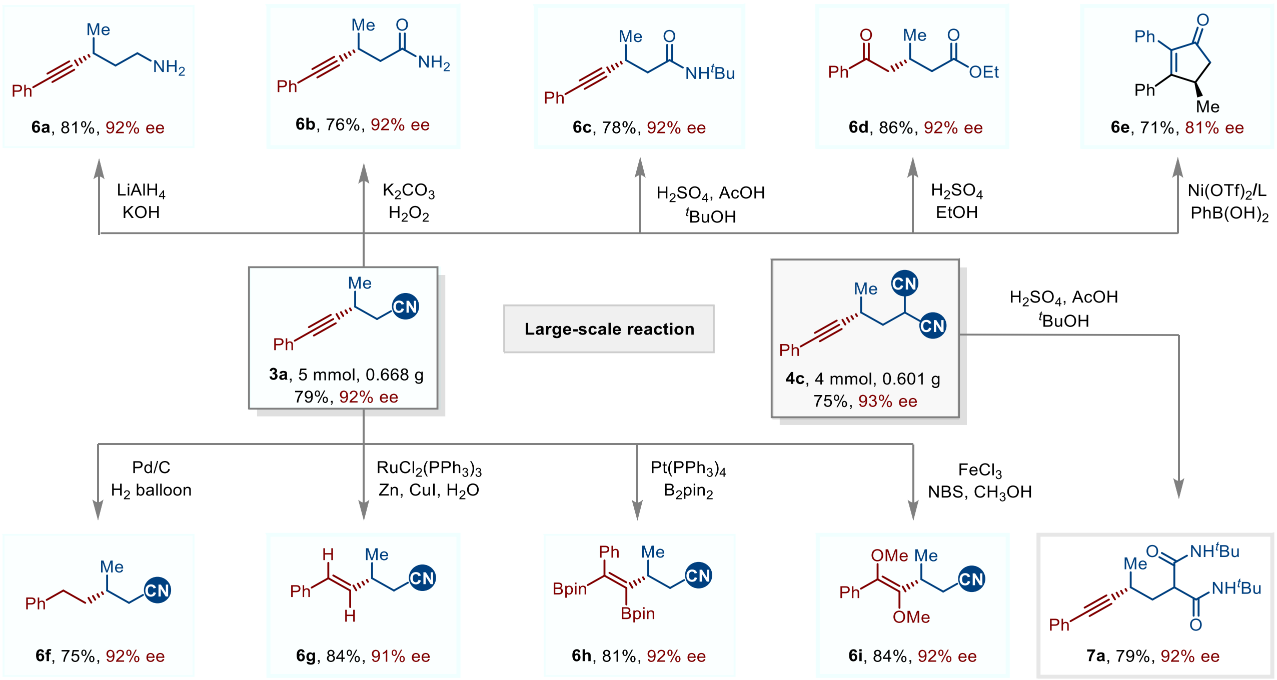

(*R*)-3-methyl-5-phenylpent-4-ynenitrile (3a)

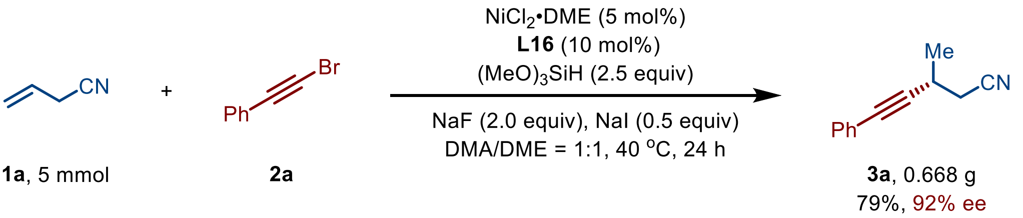

In an argon-filled glovebox, a 100 mL round-bottom pressure vessel equipped with a magnetic stir bar was charged with $NiCl_2$•DME (0.25 mmol, 5 mol%), Ligand 16 (0.5 mmol, 10 mol%), 3-butenenitrile **1a** (5.0 mmol, 1.0 equiv), NaF (10.0 mmol, 2.0 equiv), NaI (2.5 mmol, 0.5 equiv), DMA/DME (1:1, 25 mL), bromoacetylene **2a** (7.5 mmol, 1.5 equiv), and $(MeO)_3SiH$ (12.5 mmol, 2.5 equiv). The reaction mixture was stirred at 40 °C for 24 h. After completion, the reaction mixture was concentrated in vacuo and the obtained residue was purified by column chromatography on silica gel (eluent: petroleum ether/ethyl acetate) to afford the desired chiral product **3a** (9% yield, 0.668 g, 7 93% ee, rr >20:1).

HPLC condition: Chiral column OD-H, *n*-hexane/*i*-PrOH = 95:5, flow rate = 1.0 mL/min, wavelength = 254 nm, tR = 10.1 min for major isomer, tR = 8.7 min for minor isomer.

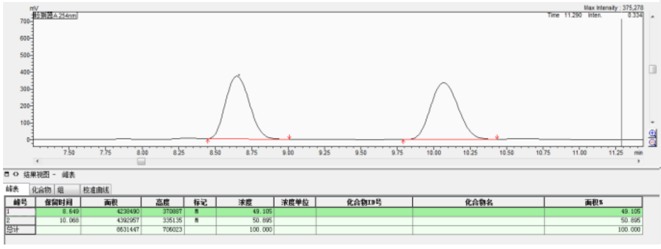

峰号	保留时间	面积	高度	标记	浓度	浓度单位	化合物ID号	化合物名	面积%
1	8.649	4238490	370887	M	49.105				49.105
2	10.068	4392957	335135	M	50.895				50.895
总计		8631447	706023		100.000				100.000

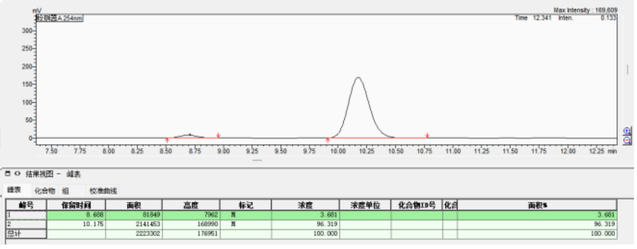

峰号	保留时间	面积	高度	标记	浓度	浓度单位	化合物ID号	化合	面积%
1	8.688	81849	7962	M	3.681				3.681
2	10.175	2141453	168990	M	96.319				96.319
总计		2223302	176951		100.000				100.000

(*R*)-2-(2-methyl-4-phenylbut-3-yn-1-yl)malononitrile (4c)

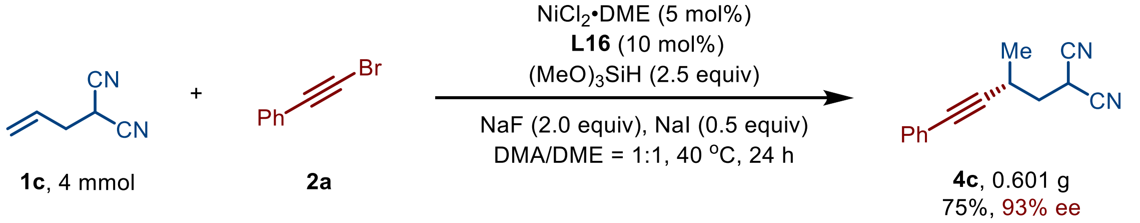

In an argon-filled glovebox, a 100 mL round-bottom pressure vessel equipped with a magnetic stir bar was charged with $NiCl_2$•DME (0.2 mmol, 5 mol%), Ligand 16 (0.4 mmol, 10 mol%), 2-allylmalononitrile **1c** (4.0 mmol, 1.0 equiv), NaF (8.0 mmol, 2.0 equiv), NaI (2.0 mmol, 0.5 equiv), DMA/DME (1:1, 20 mL), acetylene bromide **2a** (6.0 mmol, 1.5 equiv) and $(MeO)_3SiH$ (10.0 mmol, 2.5 equiv). The reaction mixture was stirred at 40 °C for 24 h. After completion, the reaction mixture was concentrated in vacuo and the obtained residue was purified by column chromatography on silica gel using a petroleum ether/ethyl acetate eluent system, yielding the desired chiral products **4c** (75%, 0.601 g, 93% ee, rr = 90:10).

HPLC condition: Chiral column OD-H, *n*-hexane/*i*-PrOH = 95:5, flow rate = 1.0 mL/min, wavelength =254 nm, tR = 21.1 min for major isomer, tR = 14.5 min for minor isomer.

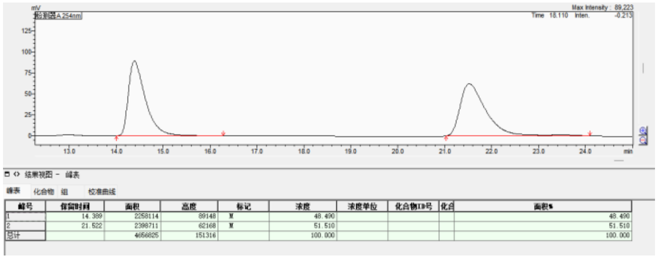

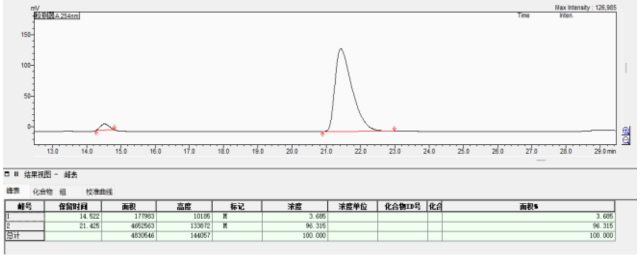

(*R*)-N-(3-methyl-5-phenylpent-4-yn-1-yl)benzamide (6a')

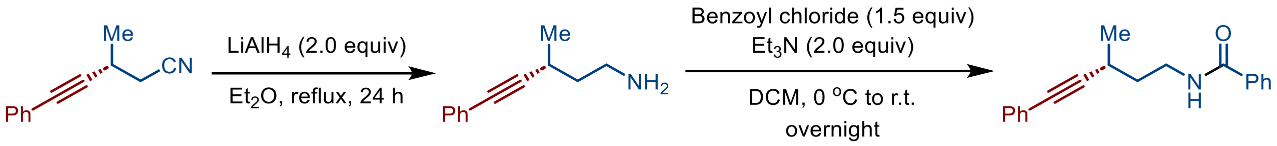

3a **6a**, 81%, 92% ee **6a'**, 80%, 92% ee

The title compound was isolated as a colourless oil (28.0 mg, 81% yield, 92% ee) after chromatography on silica with ethyl acetate/petroleum ether (1:8).

To a stirred suspension of $LiAlH_4$ (15.2 mg, 0.4 mmol, 2.0 equiv) in dry Et_2O (2.0 mL) was added **3a** (33.8 mg, 0.2 mmol) dropwise at room temperature. After heating at reflux overnight, the reaction mixture was cooled to 0 °C and carefully quenched via sequential addition of H_2O (1 mL), 15% aqueous NaOH (1 mL) and H_2O (2 mL). The mixture was subsequently stirred at room temperature for 2 h and anhydrous $MgSO_4$ was added. The suspension was filtered and extracted 3 times with diethyl ether (5 mL). The filtrate was concentrated *in vacuo* and the residue was distilled to afford the title compound **6a**.

^{1}H NMR (600 MHz, Chloroform-*d*) δ 7.34 – 7.29 (m, 2H), 7.23 – 7.18 (m, 3H), 2.91 – 2.81 (m, 2H), 2.69 (h, *J* = 7.1 Hz, 1H), 2.01 (s, 2H), 1.61 (q, *J* = 7.2 Hz, 2H), 1.21 (d, *J* = 6.9 Hz, 3H). **^{13}C NMR** (150 MHz, Chloroform-*d*) δ 131.7, 128.3, 127.7, 123.9, 93.9, 81.3, 40.34, 40.26, 24.4, 21.4.

6a (0.1 mmol, 17.3 mg) was dissolved in CH_2Cl_2 (1.0 mL), triethylamine (0.2 mmol, 2.0 equiv) was added and the mixture was stirred for 10 min, benzoyl chloride (0.15 mmol, 1.5 equiv) dissolve in CH_2Cl_2 (0.5 mL) was added dropewise and the mixture was stirred at room temperature under Ar atmosphere. Then, the aqueous layer was extracted with CH_2Cl_2 (3 × 5 mL), dried over sodium sulfate

and the solvent was removed under reduced pressure. The residue was purified by using column chromatography to provide the desired product **6a’** (44.3 mg, 80% yield, 92% ee).

^{1}H NMR (400 MHz, Chloroform-*d*) δ 7.80–7.75 (m, 2H), 7.46 (t, *J* = 7.4 Hz, 1H), 7.41 (dd, *J* = 6.6, 3.0 Hz, 2H), 7.36–7.30 (m, 5H), 6.86 (t, *J* = 5.7 Hz, 1H), 3.78–3.61 (m, 2H), 2.82 (dqd, *J* = 9.3, 6.9, 4.8 Hz, 1H), 1.95 (dtd, *J* = 13.6, 6.8, 4.8 Hz, 1H), 1.82 (ddt, *J* = 13.4, 9.3, 6.6 Hz, 1H), 1.35 (d, *J* = 6.9 Hz, 3H). **^{13}C NMR** (100 MHz, Chloroform-*d*) δ 167.6, 134.6, 131.7, 131.4, 128.5, 128.3, 127.9, 127.0, 123.5, 93.8, 82.0, 38.8, 36.2, 25.0, 21.3. **HRMS (ESI)** m/z calculated for $C_{12}H_{15}N$ $[M+H]^+$ 174.1277; found: 174.1280. **Optical rotation**: $[\alpha]^{20}_D$ =12.97 (c = 1.0 g/L, $CHCl_3$). The absolute configuration was assigned by analogy to that of **3e’**. **HPLC condition**: Chiral column ID, *n*-hexane/*i*-PrOH = 95:5, flow rate = 1.0 mL/min, wavelength = 254 nm, tR = 58.5 min for major isomer, tR = 47.2 min for minor isomer.

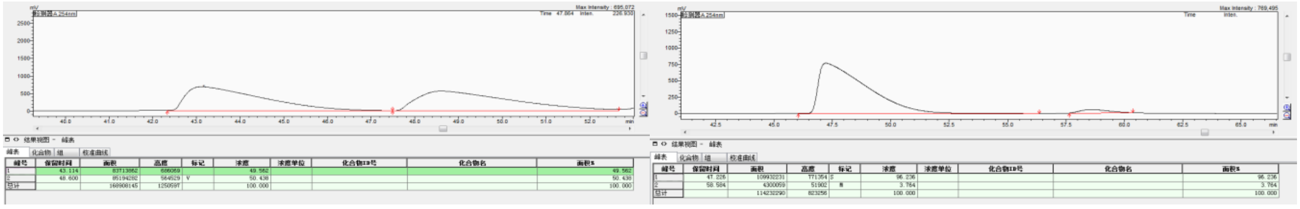

(*R*)-3-methyl-5-phenylpent-4-ynamide (6b)

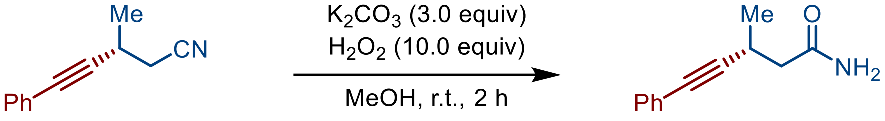

3a **6b**, 76%, 92% ee

The title compound was isolated as a yellow oil (28.4 mg, 76% yield, 92% ee) after chromatography on silica with with ethyl acetate/petroleum ether (1:2). The title compound was prepared according the literature.[7]

To a solution of the **3a** (33.8 mg, 0.2 mmol) in EtOH (2 mL) cooled 0 °C are added K_2CO_3 (3.0 equiv) and dropwise 30% H_2O_2 (0.5 mL). The reaction mixture was heated to rt for 2 h. After removal of the solvent, the reaction mixture was concentrated. the crude mixture was purified by flash column chromatography on silica gel to provide **6b** as a yellow oil.

^{1}H NMR (400 MHz, Chloroform-*d*) δ 7.44–7.39 (m, 2H), 7.34–7.30 (m, 3H), 5.81 (d, *J* = 52.8 Hz, 2H), 3.20 (h, *J* = 7.0 Hz, 1H), 2.55 (dd, *J* = 14.5, 7.8 Hz, 1H), 2.45 (dd, *J* = 14.4, 6.3 Hz, 1H), 1.37 (d,

J = 6.9 Hz, 3H). **^{13}C NMR** (100 MHz, Chloroform-*d*) δ 173.4, 131.7, 128.4, 128.1, 123.3, 92.8, 81.9, 43.3, 23.8, 21.0. **HRMS (ESI)** m/z calculated for $C_{12}H_{13}NO$ $[M+H]^+$ 188.1070; found: 188.1070. **Optical rotation**: $[\alpha]^{20}_D$ = 4.17 (c = 1.0 g/L, $CHCl_3$). The absolute configuration was assigned by analogy to that of **3e'**. **HPLC condition**: Chiral column AD-H, *n*-hexane/*i*-PrOH = 95:5, flow rate = 1 mL/min, wavelength = 254 nm, tR = 14.7 min for major isomer, tR = 13.4 min for minor isomer.

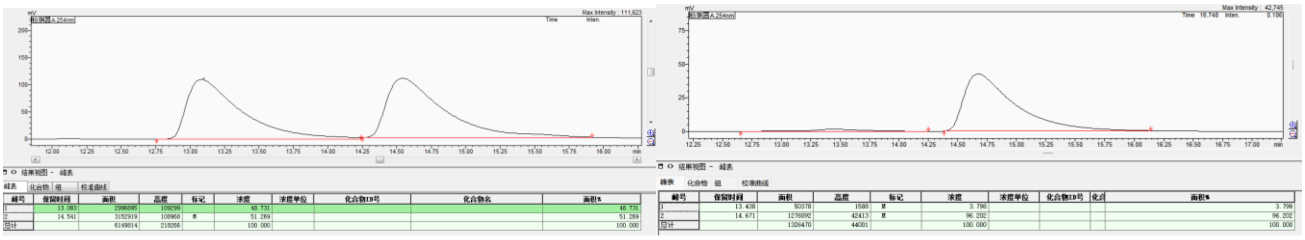

(*R*)-*N*-(tert-butyl)-3-methyl-5-phenylpent-4-ynamide (6c)

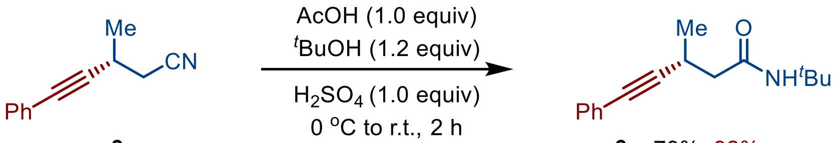

3a **6c**, 78%, 92% ee

The title compound was isolated as a colourless oil (37.9 mg, 78% yield, 92% ee) after chromatography on silica with ethyl acetate/petroleum ether (1:20).

To a degassed solution of **3a** (33.8 mg, 0.2 mmol), AcOH (0.2 mmol, 1.0 equiv) and ^{t}BuOH (0.24 mmol, 1.2 equiv) in 0 °C was slowly added H_2SO_4 (0.2 mmol, 1.0 equiv). The reaction mixture was heated to rt for 2 h. After this time, the reaction was diluted with EA and washed with saturated aqueous NaCl. The organic phase was dried over $MgSO_4$ and concentrated under reduced pressure. After the solvents were removed under reduced pressure, the residue was purified by silica gel column chromatography to provide **6c** as a yellow oil.

^{1}H NMR (400 MHz, Chloroform-*d*) δ 7.39–7.35 (m, 2H), 7.30–7.26 (m, 3H), 5.66 (s, 1H), 3.14 (h, *J* = 7.0 Hz, 1H), 2.37–2.24 (m, 2H), 1.35 (s, 9H), 1.30 (d, *J* = 7.2 Hz, 3H). **^{13}C NMR** (100 MHz, Chloroform-*d*) δ 170.3, 131.6, 128.4, 123.5, 93.2, 81.8, 51.4, 45.1, 28.9, 24.2, 20.9. **HRMS (ESI)** m/z calculated for $C_{16}H_{21}NO$ $[M+H]^+$ 244.1696; found: 244.1696. **Optical rotation**: $[\alpha]^{20}_D$ = 12.93 (c = 1.0 g/L, $CHCl_3$). The absolute configuration was assigned by analogy to that of **3e'**. **HPLC condition**:

Chiral column OD-H, *n*-hexane/*i*-PrOH = 95:5, flow rate = 1.0 mL/min, wavelength = 254 nm, tR = 8.1 min for major isomer, tR = 7.1 min for minor isomer.

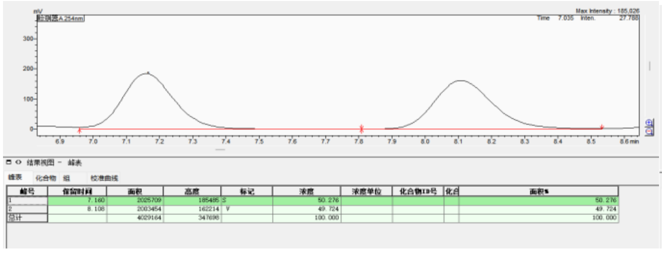

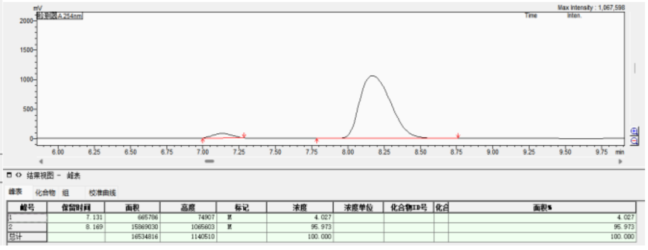

ethyl (S)-3-methyl-5-oxo-5-phenylpentanoate (6d)

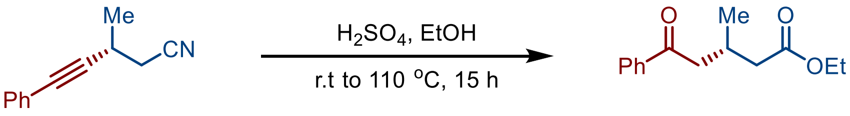

3a **6d**, 86%, 92% ee

The title compound was isolated as a colourless oil (40.3 mg, 86% yield, 92% ee) after chromatography on silica with ethyl acetate/petroleum ether (1:50).

To a stirring solution of **3a** (33.8 mg, 0.2 mmol) in EtOH (1.0 mL) was added sulfuric acid (0.38 mL) at room temperature. The resulting mixture was stirred at 110 °C for 15 h. After cooling to room temperature, the reaction mixture was quenched with aqueous $NaHCO_3$ (3 mL) and extracted with CH_2Cl_2. The organic portion was washed with brine, dried over Na_2SO_4, and filtered. After the solvent was removed under reduced pressure, the residue was purified by silica gel chromatography to afford the desired product **6d** as a colorless oil.

^{1}H NMR (400 MHz, Chloroform-*d*) δ 7.99–7.93 (m, 2H), 7.59–7.53 (m, 1H), 7.46 (t, *J* = 7.5 Hz, 2H), 4.13 (q, *J* = 7.1 Hz, 2H), 3.11 (dd, *J* = 16.2, 5.8 Hz, 1H), 2.83 (dd, *J* = 16.2, 7.7 Hz, 1H), 2.74–2.61 (m, 1H), 2.42 (dd, *J* = 15.2, 6.7 Hz, 1H), 2.30 (dd, *J* = 15.2, 7.0 Hz, 1H), 1.24 (t, *J* = 7.1 Hz, 3H), 1.05 (d, *J* = 6.7 Hz, 3H). **^{13}C NMR** (100 MHz, Chloroform-*d*) δ 199.4, 172.7, 137.1, 133.2, 128.7, 128.3, 60.4, 45.0, 41.3, 27.0, 20.2, 14.4. **HRMS (ESI)** m/z calculated for $C_{14}H_{18}O_3$ $[M+H]^+$ 220.1458; found: 220.1460. **Optical rotation**: $[\alpha]^{20}_D$ = 10.60 (c = 1.0 g/L, $CHCl_3$). The absolute configuration was assigned by analogy to that of **3e'**. **HPLC condition**: Chiral column AD-H, *n*-hexane/*i*-PrOH = 95:5, flow rate = 1.0 mL/min, wavelength = 254 nm, tR = 6.9 min for major isomer, tR = 7.3 min for minor isomer.

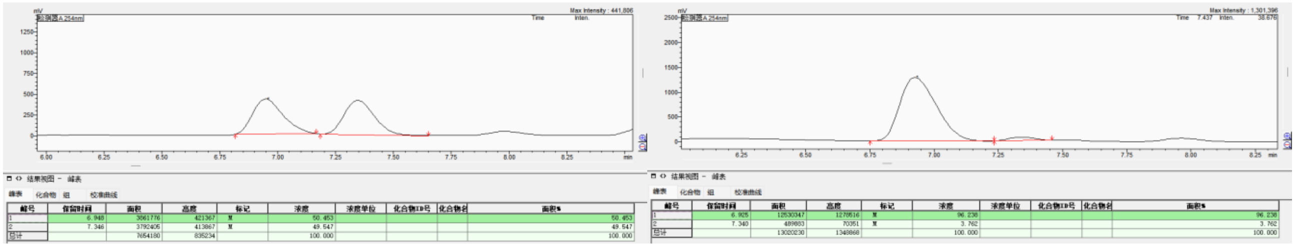

(*R*)-4-methyl-2,3-diphenylcyclopent-2-en-1-one (6e)

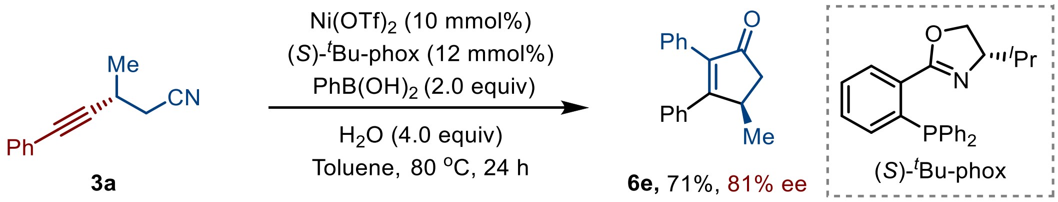

The title compound was isolated as a colourless oil (35.2 mg, 71% yield, 81% ee) after chromatography on silica with with ethyl acetate/petroleum ether (25:1). The title compound was prepared according the literature.[2]

$Ni(OTf)_2$ (7.2 mg, 0.02 mmol, 10 mol%), (*S*)-^{t}Bu-phox (9.3 mg, 0.024 mmol, 12 mol%) and 2.0 mL of toluene were added to resealable Schlenk tube under argon. The resultant solution was stirred at room temperature for 30 min, then **3a** (33.8 mg, 0.2 mmol) and phenylboronic acid (48.8 mg, 0.4 mmol, 2.0 equiv), toluene (2.0 mL), and H_2O (15 uL, 0.8 mmol, 4.0 equiv) were added. The tube was sealed, and stirred at 80 °C for 24 h. After the mixture was cooled to room temperature, 1M HCl (1.0 mL) and EA (1.0 mL) were added to the tube and the mixture was stirred for additional 30 min. The mixture was then extracted with ethyl acetate (10 mL x 3). The combined organic phase was washed with brine, dried over Na_2SO_4, and filtered. After the solvents were removed under reduced pressure, the residue was purified by silica gel column chromatography.

^{1}H NMR (600 MHz, Chloroform-*d*) δ 7.28 – 7.16 (m, 6H), 7.15 – 7.12 (m, 2H), 7.10 – 7.07 (m, 2H), 3.42 (pd, *J* = 7.1, 2.1 Hz, 1H), 2.90 (dd, *J* = 18.8, 6.7 Hz, 1H), 2.26 (dd, *J* = 18.8, 2.1 Hz, 1H), 1.08 (d, *J* = 7.2 Hz, 3H). **^{13}C NMR** (150 MHz, Chloroform-*d*) δ = 206.8, 174.1, 139.5, 134.9, 131.9, 129.6, 129.4, 128.6, 128.4, 128.3, 127.8, 43.8, 35.6, 20.0. **HRMS (ESI)** m/z calculated for $C_{18}H_{16}O$ $[M+H]^+$ 249.1274; found: 249.1274. **Optical rotation**: $[\alpha]^{20}_D$ = 16.67 (c = 1.0 g/L, $CHCl_3$). The absolute configuration was assigned by analogy to that of **3e'**. **HPLC condition**: Chiral column AD-H, *n*-

hexane/*i*-PrOH = 95:5, flow rate = 1.0 mL/min, wavelength = 254 nm, tR = 7.4 min for major isomer, tR = 10.8 min for minor isomer.

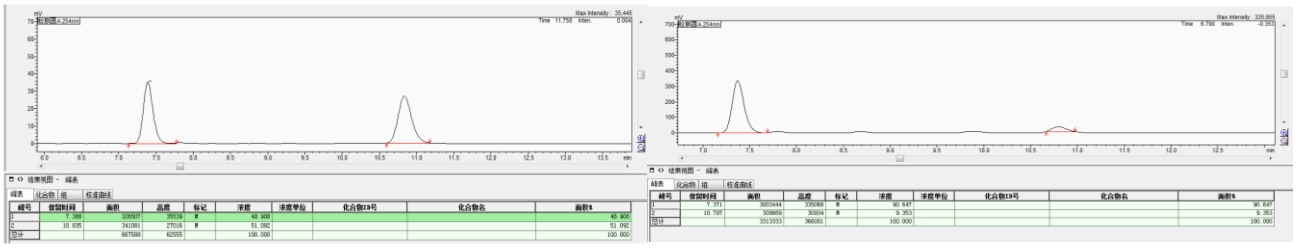

(*S*)-3-methyl-5-phenylpentanenitrile (6f)

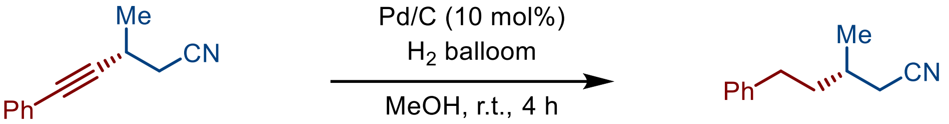

3a **6f**, 75%, 92% ee

The title compound was isolated as a brown oil (25.9 mg, 75% yield, 92% ee) after chromatography on silica with ethyl acetate/petroleum ether (1:5).

To a 10 mL round-bottomed flask charged with a stirred bar was added **3a** (33.8 mg, 0.2 mmol), Pd/C (21.1 mg, 0.02 mmol, 10%) and MeOH (4.0 mL), The mixture was carefully evacuated and backfilled with hydrogen three times then stirred at room temperature for 3 h under a H_2 balloon atmosphere. Filtered to remove insoluble materials, the solvent was removed under reduced pressure and the residue was directly purified by flash column chromatography to afford the desired product **6f**.

^{1}H NMR (600 MHz, Chloroform-*d*) δ 7.30 (t, *J* = 7.6 Hz, 2H), 7.23 – 7.16 (m, 3H), 2.72 – 2.58 (m, 2H), 2.38 – 2.25 (m, 2H), 1.94 – 1.84 (m, *J* = 6.6 Hz, 1H), 1.81 – 1.73 (m, 1H), 1.68 – 1.61 (m, 1H), 1.13 (d, *J* = 6.7 Hz, 3H). **^{13}C NMR** (150 MHz, Chloroform-*d*) δ 141.5, 128.6, 128.4, 126.2, 118.8, 37.6, 33.2, 30.0, 24.6, 19.5. **HRMS (ESI)** m/z calculated for $C_{12}H_{15}N$ $[M+H]^+$ 174.1277; found: 174.1278. **Optical rotation**: $[\alpha]^{20}_D$ = 10.57 (c = 1.0 g/L, $CHCl_3$). The absolute configuration was assigned by analogy to that of **3e'**. **HPLC condition**: Chiral column ID, *n*-hexane/*i*-PrOH = 95:5, flow rate = 1.0 mL/min, wavelength = 254 nm, tR = 12.2 min for major isomer, tR = 9.5 min for minor isomer.

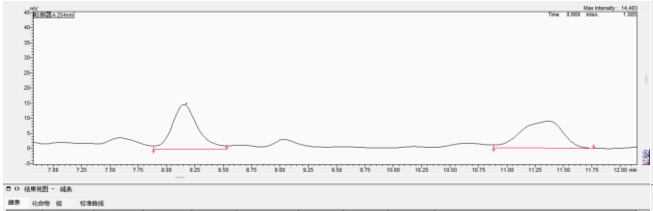

峰号	保留时间	面积	高度	标记	浓度	浓度单位	化合物ID号	化合物名	面积%
1	8.153	231858	14709		50.458				50.458
2	11.368	227647	8931	M	49.542				49.542
总计		459505	23640		100.000				100.000

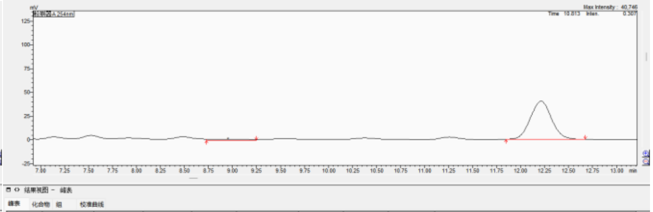

峰号	保留时间	面积	高度	标记	浓度	浓度单位	化合物ID号	化合物名	面积%
1	8.929	26877	1059	M	4.190				4.190
2	12.214	614617	40207	M	95.810				95.810
总计		641494	41265		100.000				100.000

(*R*,*E*)-3-methyl-5-phenylpent-4-enenitrile (6g)

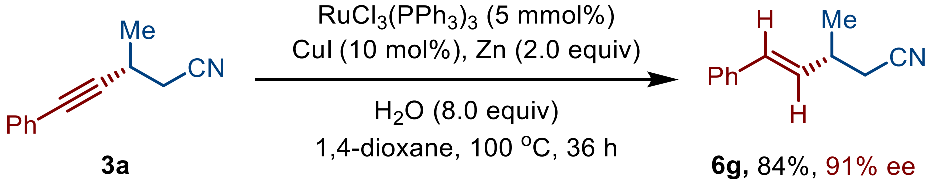

3a **6g**, 84%, 91% ee

The title compound was isolated as a colourless oil (28.7 mg, 84% yield, 91% ee) after chromatography on silica with ethyl acetate/petroleum ether (15:1).

To a solution of **3a** (33.8 mg, 0.2 mmol) in a Schlenk tube equipped with a stirred bar was added H_2O (28.8 µL, 1.6 mmol, 8.0 equiv) and CuI (10 mol%, 0.02 mmol), $RuCl_3(PPh_3)_3$ (5 mol%, 0.01 mmol) and Zn-powder (26 mg, 0.4 mmo, 2.0 equiv) in dry 1,4-dioxane (1.0 mL) under a N_2 atmosphere. The tube was sealed and placed into room temperature stirred for 36 h. Cooled down to room temperature, the solvent was removed and the residue was directly purified by flash column chromatography on silica gel.

^{1}H NMR (400 MHz, Chloroform-*d*) δ 7.39–7.29 (m, 4H), 7.23 (d, *J* = 7.0 Hz, 1H), 6.49 (d, *J* = 15.9 Hz, 1H), 6.13 (dd, *J* = 15.9, 7.5 Hz, 1H), 2.82–2.70 (m, 1H), 2.53–2.37 (m, 2H), 1.29 (d, *J* = 6.8 Hz, 3H). **^{13}C NMR** (100 MHz, Chloroform-*d*) δ 136.7, 131.6, 130.8, 128.7, 127.8, 126.4, 118.5, 34.2, 25.1, 19.9. **HRMS (ESI)** m/z calculated for $C_{12}H_{13}N$ $[M+H]^+$ 172.1121; found: 172.1123. **Optical rotation**: $[\alpha]^{20}_D$ =19.67 (c = 1.0 g/L, $CHCl_3$). The absolute configuration was assigned by analogy to that of **3e’**.

HPLC condition: Chiral column OD-H, *n*-hexane/*i*-PrOH = 97:3, flow rate = 0.6 mL/min, wavelength = 254 nm, tR = 42.1 min for major isomer, tR = 28.3 min for minor isomer.

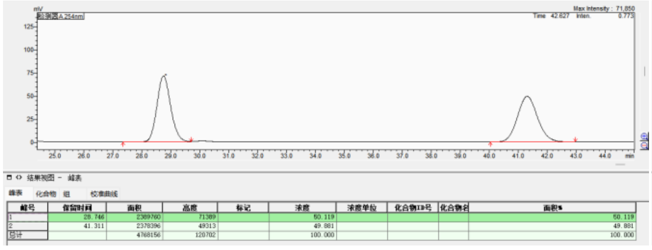

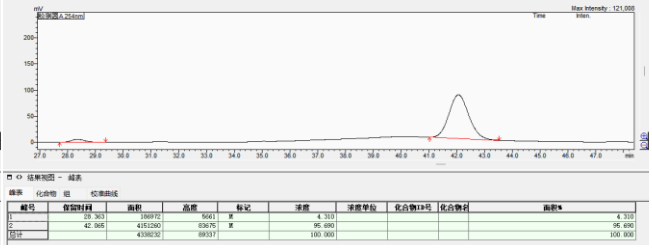

(*R*,*Z*)-3-methyl-5-phenyl-4,5-bis(4,4,5,5-tetramethyl-1,3,2-dioxaborolan-2-yl)pent-4-enenitrile (6h)

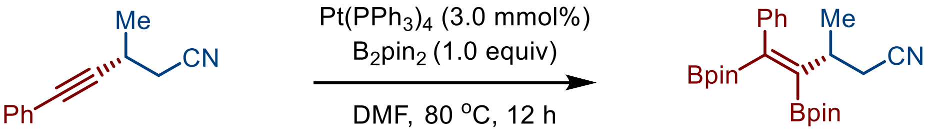

3a **6h,** 81%, 92% ee

The title compound was isolated as a yellow oil (68.6 mg, 81% yield, 92% ee) after chromatography on silica with ethyl acetate/petroleum ether (1:10).

To a degassed solution of **3a** (33.8 mg, 0.2 mmol) and *bis*(pinacolato)diboron (50.8 mg, 0.2 mmol) in DMF (2.0 mL) was added Pt(PPh_3)$_4$ (7.5 mg, 3.0 mol%). The reaction mixture was heated to 80 °C for 12 h. The reaction mixture was then extracted with AcOEt, washed with water, dried over $MgSO_4$ and concentrated in vacuo. The residue was purified by flash chromatography on silica gel with ethyl acetate/petroleum ether to afford compound **6h**.

^{1}H NMR (400 MHz, Chloroform-*d*) δ 7.32 (t, *J* = 7.5 Hz, 2H), 7.25–7.20 (m, 1H), 7.12–7.05 (m, 2H), 2.91–2.80 (m, 1H), 2.60 (dd, *J* = 16.6, 9.6 Hz, 1H), 2.40 (dd, *J* = 16.7, 6.1 Hz, 1H), 1.32 (s, 12H), 1.22 (s, 12H), 1.14 (d, *J* = 6.7 Hz, 3H). **^{13}C NMR** (150 MHz, Chloroform-*d*) δ 141.0, 128.4, 127.4, 126.6, 119.7, 84.2, 84.0, 33.5, 25.1, 25.0, 24.9, 24.7, 23.3, 19.7. **HRMS (ESI)** m/z calculated for $C_{26}H_{39}B_2NO_2$ $[M+H]^+$ 417.3167; found: 417.3169. **Optical rotation**: $[\alpha]^{20}_D$ = 16.13 (c = 1.0 g/L, $CHCl_3$). The absolute configuration was assigned by analogy to that of **3e'**. **HPLC condition**: Chiral column IE, *n*-hexane/*i*-PrOH = 99:1, flow rate = 1.0 mL/min, wavelength = 254 nm, tR = 13.6 min for major isomer, tR = 11.5 min for minor isomer.

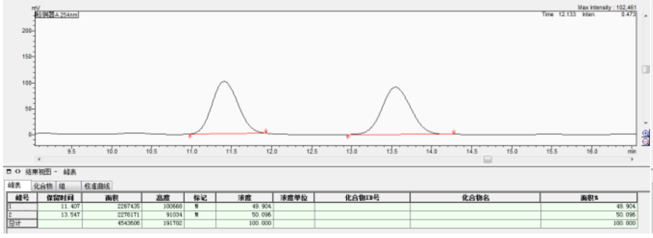

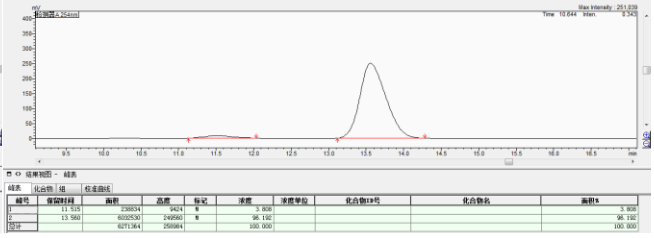

(*R*,*E*)-4,5-dimethoxy-3-methyl-5-phenylpent-4-enenitrile (6i)

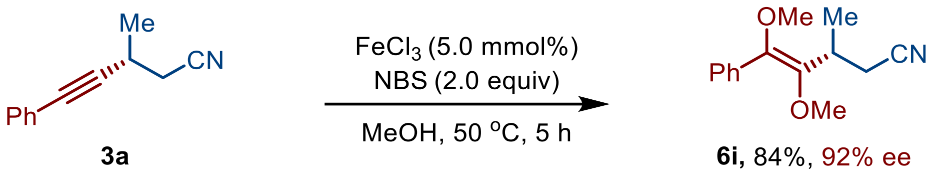

The title compound was isolated as a white solid (38.8 mg, 84% yield, 92% ee) after chromatography on silica with ethyl acetate/petroleum ether (1:30).

3a (33.8 mg, 0.2 mmol), NBS (71.2 mg, 0.4 mmol, 2.0 equiv), $FeCl_3$ (1.6 mg, 5 mol%) and MeOH (2 mL) were added to an oven-dried test tube equipped with a magnetic stirring bar at room temperature. Then the reaction solution was stirring for 5 h at 50 °C. The solvent was removed after the reaction was completed, and the crude mixture was purified by flash column chromatography on silica gel.

^{1}H NMR (400 MHz, Chloroform-*d*) δ 7.67–7.62 (m, 2H), 7.44–7.39 (m, 3H), 3.60 (s, 3H), 3.53 (s, 3H), 3.39–3.32 (m, 1H), 2.46 (dd, *J* = 17.0, 10.2 Hz, 1H), 2.00 (dtd, *J* = 12.7, 6.3, 2.9 Hz, 1H), 1.47 (dd, *J* = 6.3, 1.0 Hz, 3H). **^{13}C NMR** (100 MHz, Chloroform-*d*) δ 135.0, 129.9, 129.5, 128.0, 118.9, 105.5, 86.4, 53.4, 53.4, 43.4, 24.3, 20.1. **HRMS (ESI)** m/z calculated for $C_{14}H_{17}NO_2$ $[M+H]^+$ 232.1332; found: 232.1333. **Optical rotation**: $[\alpha]^{20}_D$ = 14.60 (c = 1.0 g/L, $CHCl_3$). The absolute configuration was assigned by analogy to that of **3e'**. **HPLC condition**: Chiral column AD-H, *n*-hexane/*i*-PrOH = 98:2, flow rate = 0.5 mL/min, wavelength = 254 nm, tR = 19.6 min for major isomer, tR = 20.8 min for minor isomer.

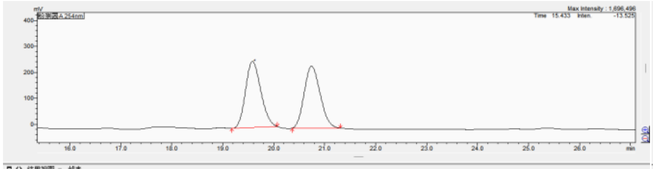

峰号	保留时间	面积	高度	标记	浓度	浓度单位	化合物ID号	化合物名	面积%
1	19.587	5340238	255012	M	50.673				50.673
2	20.743	5198337	238766	M	49.327				49.327
总计		10538575	493778		100.000				100.000

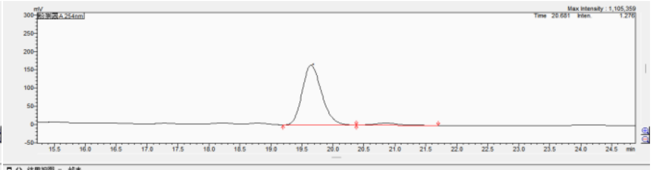

峰号	保留时间	面积	高度	标记	浓度	浓度单位	化合物ID号	化合物名	面积%
1	19.648	3568808	164151		96.277				96.277
2	20.856	138022	5891		3.723				3.723
总计		3706830	170041		100.000				100.000

(*R*)-N1,N3-di-tert-butyl-2-(2-methyl-4-phenylbut-3-yn-1-yl)malonamide (7a)

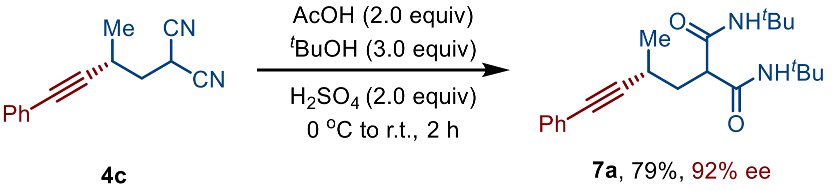

The title compound was isolated as a colourless oil (56.3 mg, 79% yield, 92% ee) after chromatography on silica with with ethyl acetate/petroleum ether (1:7). The title compound was prepared according the literature.[9]

To a degassed solution of **4c** (41.6 mg, 0.2 mmol), AcOH (0.4 mmol, 2.0 equiv) and ^{t}BuOH (0.6 mmol, 1.5 equiv) in 0 °C was slowly added H_2SO_4 (0.4 mmol, 2.0 equiv). The reaction mixture was heated to rt for 2 h. After this time, the reaction was diluted with ethyl acetate and washed with saturated aqueous NaCl. The organic phase was dried over $MgSO_4$ and concentrated under reduced pressure. After the solvents were removed under reduced pressure, the residue was purified by silica gel column chromatography.

^{1}H NMR (400 MHz, Chloroform-*d*) δ 7.46–7.39 (m, 2H), 7.35–7.27 (m, 3H), 6.87 (s, 1H), 6.16 (s, 1H), 3.17 (dd, *J* = 10.0, 5.5 Hz, 1H), 2.69 (dt, *J* = 10.2, 6.4 Hz, 1H), 2.09–1.97 (m, 2H), 1.38 (s, 9H), 1.36 (s, 9H), 1.32 (d, *J* = 6.8 Hz, 3H). **^{13}C NMR** (100 MHz, Chloroform-*d*) δ 170.7, 169.7, 131.7, 128.4, 128.0, 123.5, 92.6, 82.4, 55.4, 51.7, 51.2, 39.4, 28.7, 25.2, 21.3. **HRMS (ESI)** m/z calculated for $C_{22}H_{32}N_2O_2$ $[M+H]^+$ 357.2537; found: 357.2537. **Optical rotation**: $[\alpha]^{20}_D$ = 9.27 (c = 1.0 g/L, $CHCl_3$). The absolute configuration was assigned by analogy to that of **3e'**. **HPLC condition**: Chiral column OD-H, *n*-hexane/*i*-PrOH = 95:5, flow rate = 1.0 mL/min, wavelength = 254 nm, tR = 4.3 min for major isomer, tR = 4.8 min for minor isomer.

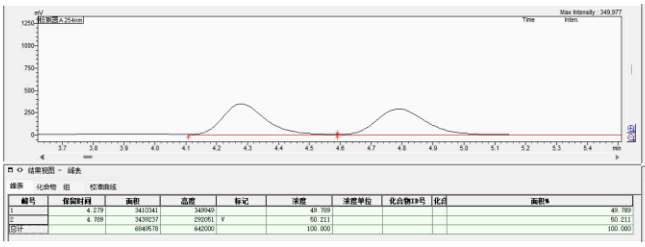

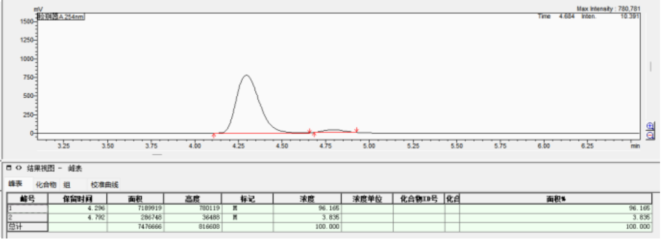

6. Mechanistic studies

1. Deuterium labeling experiment

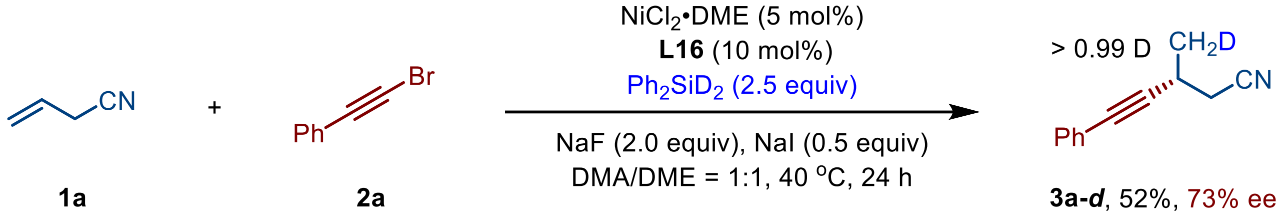

In an Ar-filled glovebox, a 4 mL reaction tube equipped with a magnetic stir bar was added $NiCl_2$•DME (0.01 mmol, 5 mol%), Ligand 16 (0.02 mmol, 10 mol%), 3-butenenitrile **1a** (0.2 mmol, 1.0 equiv), NaF (0.4 mmol, 2.0 equiv), NaI (0.1 mmol, 0.5 equiv), DMA/DME (1:1, 1.0 mL), a 1-bromo-2-phenylacetylene **2a** (0.3 mmol, 1.5 equiv) and Ph_2SiD_2 (0.5 mmol, 2.5 equiv). The reaction mixture was stirred at 40 °C for 24 h. After completion, the reaction mixture was concentrated in vacuo and the obtained residue was purified by column chromatography on silica gel using a petroleum ether/ethyl acetate eluent system, yielding the desired chiral products **3a-*d***.

(*R*)-3-(methyl-d)-5-phenylpent-4-ynenitrile (3a-*d*)

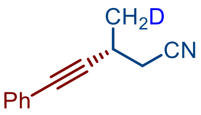

This compound was synthesized according to general procedure. The residue was purified by column chromatography on silica gel (PE/EtOAc = 40:1) to afford the product **3a-*d*** (18.1 mg, 52% yield, 73% ee, rr >20:1) as a yellow oil.

^{1}H NMR (600 MHz, Chloroform-*d*) δ 7.44 – 7.39 (m, 2H), 7.33 – 7.27 (m, 3H), 3.07 (p, *J* = 6.8 Hz, 1H), 2.67 – 2.56 (m, 2H), 1.44 – 1.41 (m, 2H). **^{13}C NMR** (150 MHz, Chloroform-*d*) δ 131.8, 128.4, 122.8, 117.8, 89.8, 83.0, 25.4, 24.2, 20.3 (d, *J* = 20.0 Hz). The absolute configuration was assigned by analogy to that of **3e'**. **HPLC condition**: Chiral column OD-H, *n*-hexane/*i*-PrOH = 95:5, flow rate = 1.0 mL/min, wavelength = 254 nm, tR = 10.4 min for major isomer, tR = 8.8 min for minor isomer.

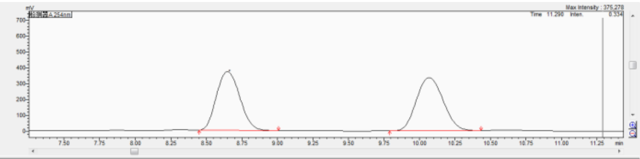

峰号	保留时间	面积	高度	标记	浓度	浓度单位	化合物ID号	化合物名	面积%
1	8.649	4238490	370887	[illegible]	49.105				49.105
2	10.068	4392957	335135	[illegible]	50.895				50.895
总计		8631447	706023		100.000				100.000

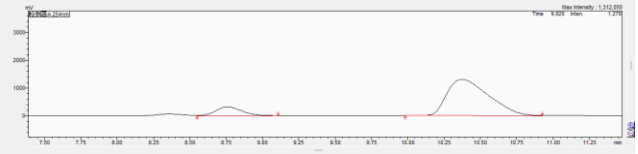

峰号	保留时间	面积	高度	标记	浓度	浓度单位	化合物ID号	化合物名	面积%
1	8.761	4008527	316135	M	13.345				13.345
2	10.372	26028489	1306037	M	86.655				86.655
总计		30037016	1622172		100.000				100.000

2. Reaction in the absence of cyano group

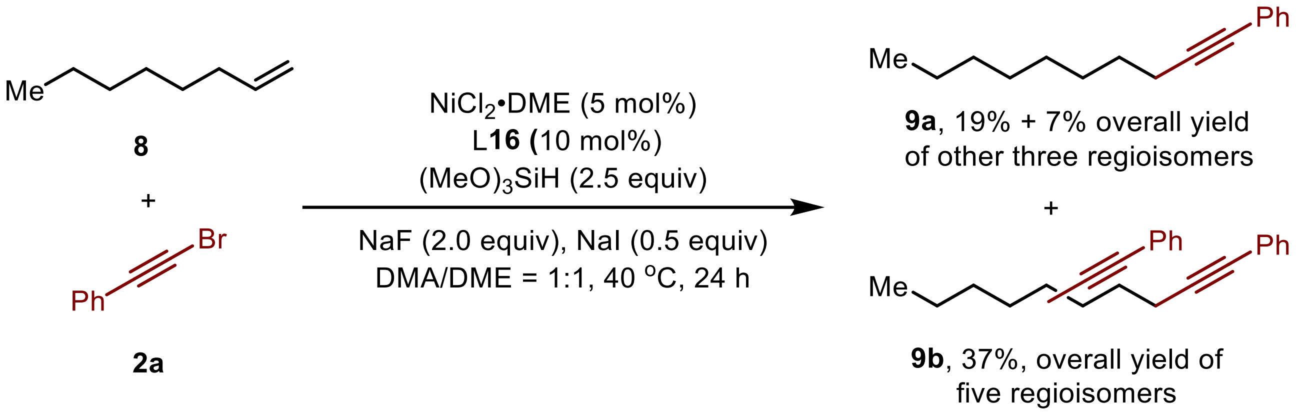

In an Ar-filled glovebox, a 4 mL reaction tube equipped with a magnetic stir bar was added $NiCl_2$•DME (0.01 mmol, 5 mol%), Ligand 16 (0.02 mmol, 10 mol%), 1-Octene **8** (0.2 mmol, 1.0 equiv), NaF (0.4 mmol, 2.0 equiv), NaI (0.1 mmol, 0.5 equiv), DMA/DME (1:1, 1.0 mL), a bromo-2-phenylacetylene **2a** (0.3 mmol, 1.5 equiv) and $(MeO)_3SiH$ (0.5 mmol, 2.5 equiv). The reaction mixture was stirred at 40 °C for 24 h. After completion of the reaction, the reaction mixture was analyzed by GC-MS (Fig. S5). Only product **9a** was successfully isolated via column chromatography. The remaining products and unreacted starting materials could not be efficiently separated, preventing acquisition of their clean NMR spectra.

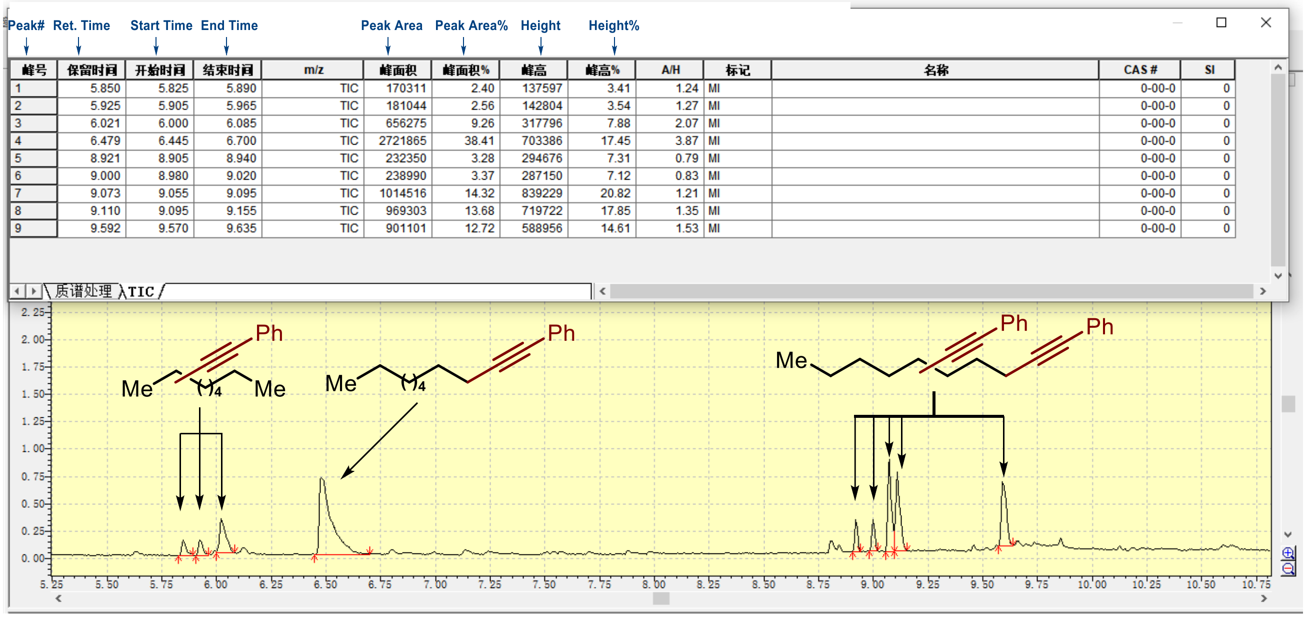
Peak# Ret. Time Start Time End Time Peak Area Peak Area% Height Height%

峰号	保留时间	开始时间	结束时间	m/z	峰面积	峰面积%	峰高	峰高%	A/H	标记	名称	CAS #	SI
1	5.850	5.825	5.890	TIC	170311	2.40	137597	3.41	1.24	MI		0-00-0	0
2	5.925	5.905	5.965	TIC	181044	2.56	142804	3.54	1.27	MI		0-00-0	0
3	6.021	6.000	6.085	TIC	656275	9.26	317796	7.88	2.07	MI		0-00-0	0
4	6.479	6.445	6.700	TIC	2721865	38.41	703386	17.45	3.87	MI		0-00-0	0
5	8.921	8.905	8.940	TIC	232350	3.28	294676	7.31	0.79	MI		0-00-0	0
6	9.000	8.980	9.020	TIC	238990	3.37	287150	7.12	0.83	MI		0-00-0	0
7	9.073	9.055	9.095	TIC	1014516	14.32	839229	20.82	1.21	MI		0-00-0	0
8	9.110	9.095	9.155	TIC	969303	13.68	719722	17.85	1.35	MI		0-00-0	0
9	9.592	9.570	9.635	TIC	901101	12.72	588956	14.61	1.53	MI		0-00-0	0

Fig. S5. GC-MS spectra of 1-Octene 8 with bromo-2-phenylacetylene 2a

dec-1-yn-1-ylbenzene (9a)

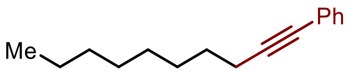

This compound was synthesized according to general procedure A. The residue was purified by column chromatography on silica gel (PE/EtOAc = 50:1) to afford the product **9a** (8.2 mg, 19% yield) as a yellow oil.

^{1}H NMR (400 MHz, Chloroform-*d*) δ 7.45 – 7.41 (m, 2H), 7.35 – 7.28 (m, 3H), 2.44 (t, J = 7.1 Hz, 2H), 1.68 – 1.61 (m, 2H), 1.53 – 1.42 (m, 2H), 1.40 – 1.27 (m, 8H), 0.93 (t, J = 6.7 Hz, 3H). ^{13}C NMR (100 MHz, Chloroform-*d*) δ 131.7, 128.3, 127.6, 124.2, 90.6, 80.7, 32.0, 29.4, 29.3, 29.1, 28.9, 22.8, 19.6, 14.3. **HRMS (ESI)** m/z calculated for $C_{16}H_{23}$ $[M+H]^+$ 215.1794; found: 215.1791.

3. Non-linear effect study

We investigated the dependence of product enantiomeric excess on catalyst ee. To perform this study we prepared ligand (*S*,*S*)-**L16**, and mixed with ligand racemic **L16** to afford 100%, 80%, 60%, 40%, 20% and 0% ee of the catalyst in standard, determined by chiral HPLC analysis, a linear correlation was observed by plotting the ee of **3a** against the ee of the ligand.

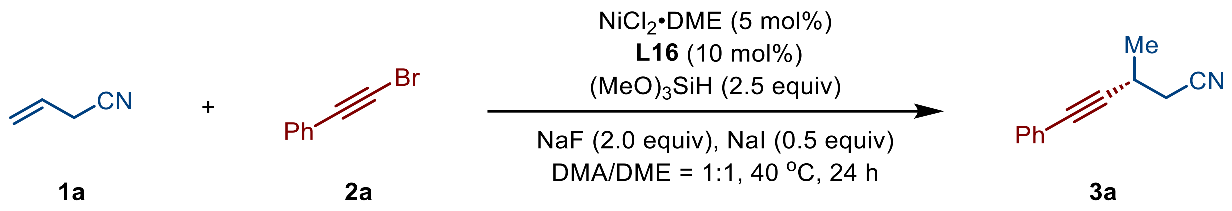

ee of ligand (%)	ee of product (%)
0	0
20	16
40	32
60	52
80	72
100	92

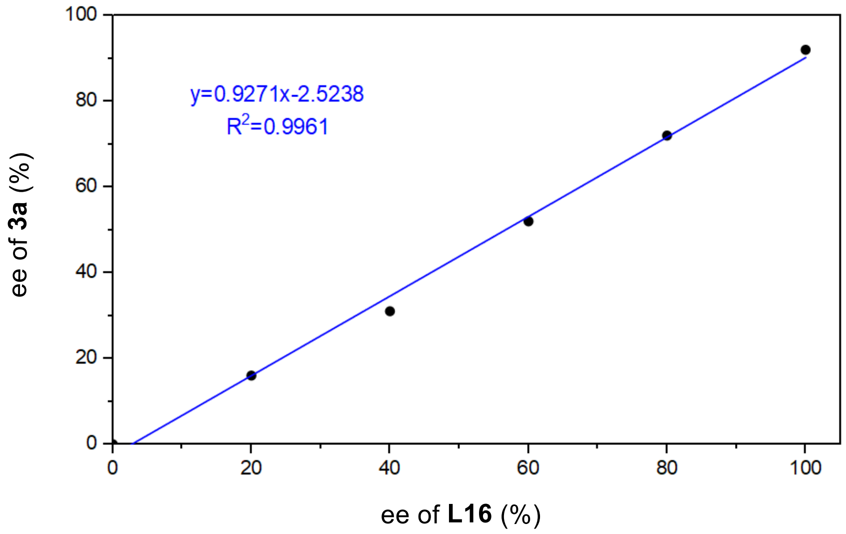

Fig. S6. Non-linear effect study

7. X-ray crystallographic data

Crystals were grown from a mixture of ethyl acetate and *n*-hexane.

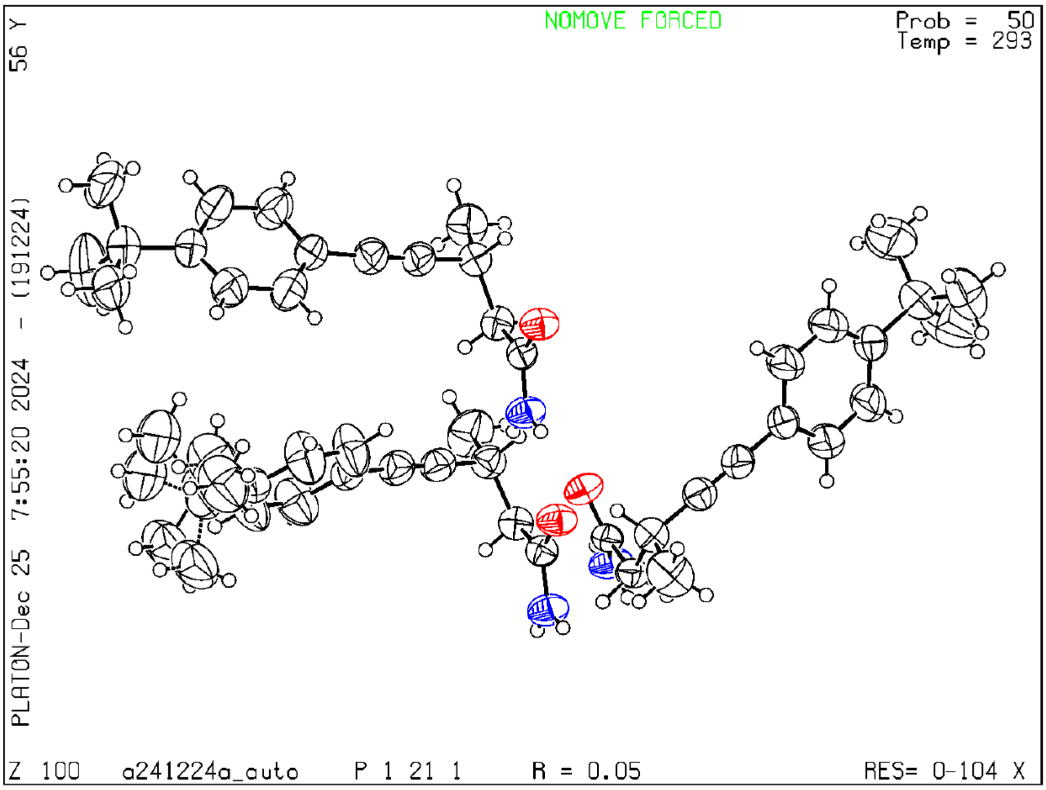

Fig. S7. X-ray structure of compound 3e' (The thermal ellipsoid was drawn at the 50% probability level, CCDC 2417811.).

Table S11. Details for X-ray data collection and structure refinement for compound 3e'.

CCDC number	2417811
Empirical formula	$C_{16}H_{21}NO$
Formula weight	243.34
Temperature [K]	293(2)
Crystal system	monoclinic
Space group (number)	$P2_1$ (4)
a [Å]	6.6656(3)
b [Å]	15.6186(5)
c [Å]	22.0288(9)
α [°]	90
β [°]	95.980(4)
γ [°]	90
Volume [Å^3]	2280.88(15)
Z	6
ρ_{calc} [gcm^{-3}]	1.063
μ [mm^{-1}]	0.507
F(000)	792
Crystal size [mm^3]	0.14×0.23×0.25
Crystal colour	clear light colourless
Crystal shape	block
Radiation	Cu K_α (λ=1.54184 Å)
2θ range [°]	6.95 to 134.08 (0.84 Å)
Index ranges	$-7 \le h \le 7$, $-18 \le k \le 18$, $-25 \le l \le 26$
Reflections collected	16392
Independent reflections	8046 [R_{int} = 0.0322, R_{sigma} = 0.0346]
Completeness to θ = 67.040°	99.8 %
Data / Restraints / Parameters	8046 / 39 / 515
Absorption correction T_{min}/T_{max} (method)	0.6131 / 1.0000 (multi-scan)
Goodness-of-fit on F^2	1.018
Final R indexes [$I \geq 2\sigma(I)$]	R_1 = 0.0547, wR_2 = 0.1492
Final R indexes [all data]	R_1 = 0.0657, wR_2 = 0.1625
Largest peak/hole [eÅ^{-3}]	0.24/−0.21
Flack X parameter	0.5

8. DFT calculations

1. Computational method

All the density functional theory (DFT) calculations were performed with the Gaussian 09 software package *(96)*. The geometry optimizations were carried out using (u)B3LYP-D3(BJ) functional with a mixed basis set of SDD for Ni and Br and 6-31G(d) for all other atoms *(97-99)*. Vibrational frequencies were calculated at the same level of theory to confirm if each optimized structure is a local minimum on the respective potential energy surface or a transition state structure with only one imaginary frequency. Selected transition-state structures were confirmed to connect the correct reactants and products by intrinsic reaction coordinate (IRC) calculations *(100-101)*. Thorough conformation search of transition states was performed with the Conformer-Rotamer Ensemble Sampling Tool (CREST) that developed by Grimme and co-workers *(102)*. To obtain better accuracy, solution-phase single-point energies for the optimized geometries were recalculated using (u)TPSS-D3(BJ) *(103)* functional with a larger basis set of SDD for Ni and Br and 6-311+G(d,p) for all other atoms. Solvation effects were taken into account by performing single-point calculations with the SMD model *(104)*. Since the solvent parameters for the mixed solvent (DMA : DME = 1:1) are not available under Gaussian, the full SMD parameters of the solvent are defined (Solvent = Generic, Eps = 22.5, Eps Inf = 1.98). The final free energies reported in the article are the large basis set single-point energies corrected by gas-phase Gibbs free energy correction (at 298.15 K). The 3D diagrams of molecules were generated using CYLview *(105)*. Hirshfeld surface analysis *(106-108)* map, Electrostatic potential-colored molecular van der Waals surface penetration map and Non-covalent interactions *(109-111)* were performed with Multiwfn *(112-113)*, and were visualized with visual molecular dynamics (VMD) *(114)*.

2. Possibility of the reaction initiated by the C-Br bond cleavage

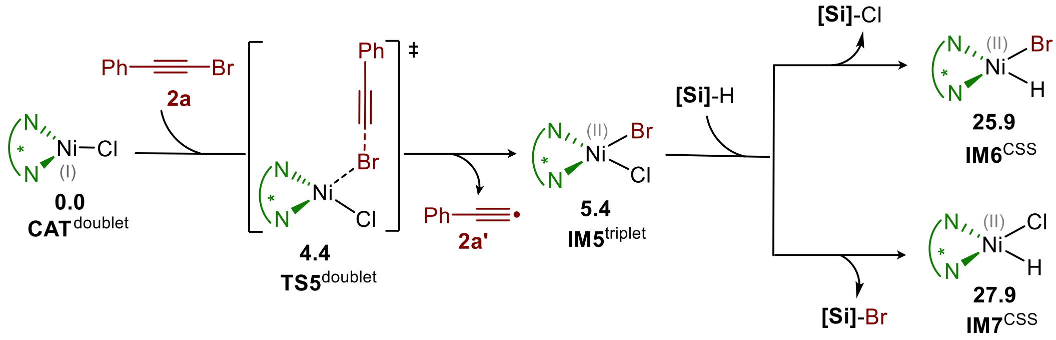

Fig. S8. Possibility of the reaction initiated by the C-Br bond cleavage.

3. Chain-walking process

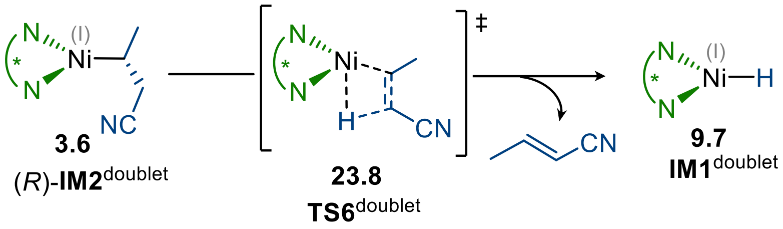

Fig. S9. The chain-walking process from (*R*)-IM2^{doublet}.

4. Other possibility for migratory insertion

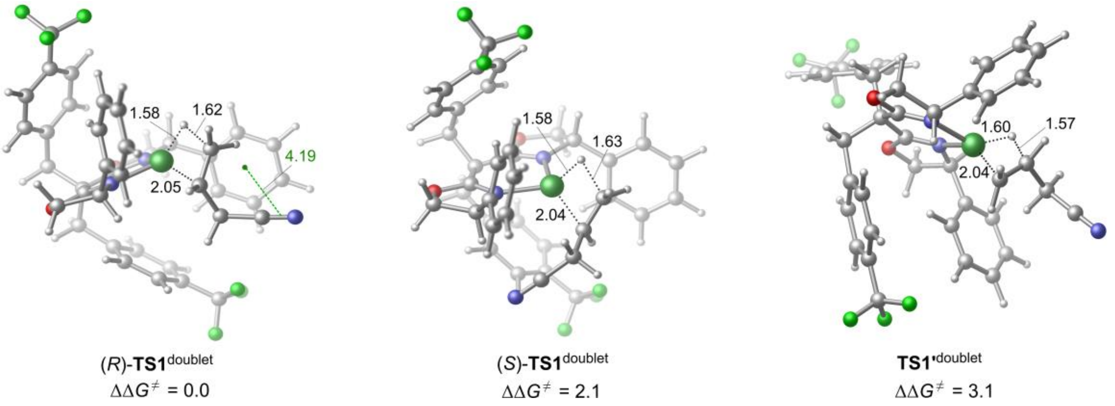

Fig. S10. Selectivity-determining migratory insertion transition states. The Gibbs free energies and bond distances are given in kcal/mol and Å, respectively.

5. Analysis of the interaction

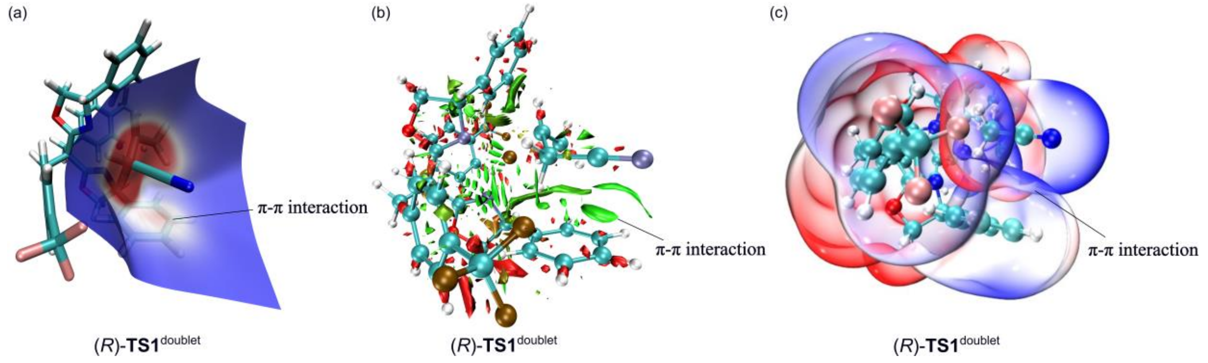

Fig. S11. Computed (a) Hirshfeld surface analysis map; (b) Isosurface map of IRI-π = 1.0 and (c) electrostatic potential surface of (*R*)-TS1$^{\mathbf{doublet}}$.

6. Other conformations of (*S*)-TS1$^{\mathbf{doublet}}$

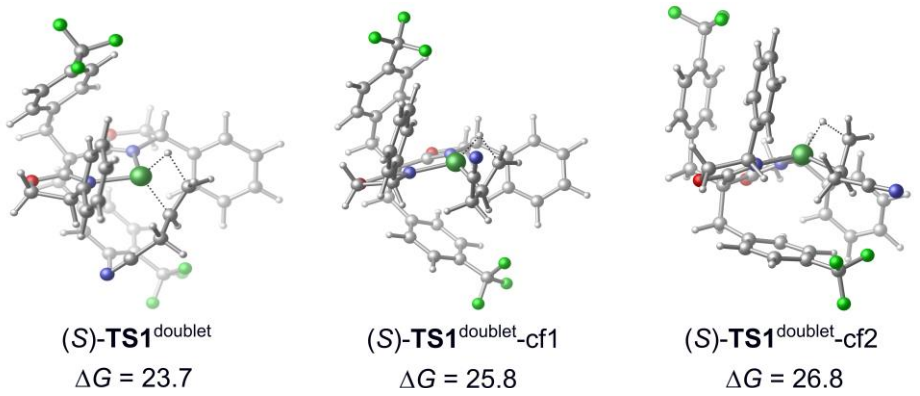

Fig. S12. Other conformations of (*S*)-TS1$^{\mathbf{doublet}}$. The Gibbs free energies are given in kcal/mol.

7. Table S12. Calculated Energies and Energy Corrections

Stationary point	Single-point energy-(u)TPSS-D3(BJ)-SMD/6-311+G(d,p)&SDD (a.u.)	Thermal correction to Gibbs free energy at 298.15 K (a.u.)
**1a**	-210.2551386	0.05104
**2a**	-321.3179692	0.065802
**2a'**	-307.8539114	0.066007
**[Si]-H**	-898.0908757	0.101242
**[Si]-Cl**	-1357.790637	0.094965
**[Si]-Br**	-910.9349566	0.092721

**3a**	-518.8567571	0.153872
**CAT**^{doublet}	-2841.941882	0.488063
(*R*)-**TS1**^{doublet}	-2592.486492	0.569051
**IM1**^{doublet}	-2382.219731	0.497118
(*R*)-**IM2**^{doublet}	-2592.518986	0.57279
(*R*)-**TS2**^{doublet}	-2913.857636	0.665843
(*R*)-**IM3**^{triplet}	-2605.979441	0.574416
(*R*)-**TS3**^{doublet}	-2913.85284	0.66563
(*R*)-**IM4**^{doublet}	-2913.945355	0.673
(*R*)-**TS4**^{doublet}	-2913.933861	0.670361
**CAT'**^{doublet}	-2395.081523	0.484653
(*S*)-**TS1**^{doublet}	-2592.485458	0.571399
**TS1'**^{doublet}	-2592.47798	0.565451
**TS5**^{doublet}	-3163.27797	0.579032
**IM5**^{triplet}	-2855.396815	0.487342
**IM6**^{CSS}	-2395.665376	0.494631
**IM7**^{CSS}	-2842.51779	0.496719
**TS6**^{doublet}	-2592.485041	0.571138

8. Cartesian coordinates

1a

C -2.36047000 0.95458700 -0.83795800

H -2.98995700 1.83001900 -0.96669000

H -2.72161500 0.02452000 -1.27123400

C -1.20502700 1.02063400 -0.18086000

H -0.85784400 1.96390700 0.23599800

C -0.30053200 -0.17139100 0.04450700

H -0.20258100 -0.38220900 1.11874300

H -0.72537700 -1.06822300 -0.42092800

C	1.04804900	0.04922500	-0.49415500
N	2.10928200	0.24648100	-0.91972000

2a

C	-1.33699900	1.90348200	0.00024100
C	-0.12675400	1.90361000	0.00001700
C	-2.76413800	1.90367100	0.00017400
C	-3.47612000	3.11676200	0.04236400
C	-3.47641000	0.69076100	-0.04208800
C	-4.86813600	3.11196700	0.04211400
H	-2.92621000	4.05175100	0.07510300
C	-4.86843000	0.69589800	-0.04197400
H	-2.92672800	-0.24436700	-0.07478200
C	-5.56821400	1.90401600	0.00003700
H	-5.40852700	4.05359700	0.07502000
H	-5.40904200	-0.24560400	-0.07493200
H	-6.65434900	1.90415300	-0.00001900
Br	1.70277600	1.90397200	-0.00024900

2a'

C	-2.07994600	0.00014900	0.08411100
C	-3.34580000	-0.00008500	-0.09854800
C	-0.67956500	0.00006600	0.03452500
C	0.04530000	-1.22410700	0.01730100
C	0.04543000	1.22416500	0.01728700
C	1.43140000	-1.21757900	-0.01112800
H	-0.51095700	-2.15569900	0.02768600
C	1.43153200	1.21749600	-0.01113000
H	-0.51073100	2.15581400	0.02766300
C	2.12654500	-0.00007800	-0.02572500

H	1.97962200	-2.15504600	-0.02320900
H	1.97984700	2.15490700	-0.02321200
H	3.21284800	-0.00014300	-0.04908700

[Si]-H

Si	-2.01832200	-0.45926800	-0.82698700
H	-0.89036800	0.15956300	-0.01144700
O	-2.09033200	0.78270400	-1.96958100
O	-1.01158000	-1.86675900	-1.23828200
O	-3.07981000	-0.63757100	0.47451400
C	-1.34951000	1.97536100	-1.87735800
H	-0.77019500	2.12458200	-2.79921500
H	-2.02792100	2.83199500	-1.76105400
H	-0.65077600	1.97311200	-1.02799300
C	-2.82791000	-0.13817700	1.76543500
H	-3.56909700	0.63132800	2.02246800
H	-2.91346500	-0.95036300	2.50075100
H	-1.82597300	0.30568800	1.85764000
C	0.31379900	-1.93481700	-0.74075600
H	0.33402600	-1.94957600	0.35569100
H	0.77743800	-2.85859200	-1.10969800
H	0.91677400	-1.08361800	-1.07884200
Na	-2.37310400	-2.99363300	-2.44795300
F	-3.24173800	-1.26869000	-1.79262700

[Si]-Cl

Si	-1.82747300	-0.39785200	-0.95912700
O	-1.81977100	0.94383800	-1.92281600
O	-0.90372100	-1.82415600	-1.19522300
O	-2.99720800	-0.79152600	0.23309800

C	-1.14015500	2.18032300	-1.81968900
H	-1.42918200	2.77419600	-2.69315000
H	-1.41764300	2.72259800	-0.90987100
H	-0.05342200	2.04833100	-1.81860100
C	-3.14888300	-0.26816800	1.54435600
H	-4.12509400	-0.59980000	1.91612000
H	-2.36107100	-0.62232500	2.21707300
H	-3.12636100	0.82514200	1.54105800
C	0.47098900	-2.05334800	-0.92278100
H	0.66999600	-2.09693800	0.15267800
H	0.73918500	-3.00990600	-1.38558900
H	1.09824100	-1.26721500	-1.35270100
Na	-2.95542800	-2.84478300	-0.84205100
F	-2.94294100	-1.12014100	-2.07168800
Cl	-0.38182800	0.45947700	0.54451000

[Si]-Br

Si	-1.93702000	-0.55832000	-0.72316000
O	-1.94031900	0.56140900	-2.00603700
O	-0.92630500	-1.83646100	-1.21463500
O	-2.88548100	-0.50746000	0.61417300
C	-1.58120700	1.93765800	-2.02757300
H	-0.49621900	2.06633300	-2.07308100
H	-2.04635700	2.37872300	-2.91599100
H	-1.95024900	2.45536300	-1.13856300
C	-2.73638900	0.00623800	1.92646200
H	-3.67609000	-0.19712200	2.44946300
H	-1.91255800	-0.47966200	2.45689600
H	-2.55064400	1.08393200	1.91800500

C	0.26773400	-2.35016800	-0.63744400
H	0.16610700	-2.46390500	0.44499300
H	0.44172700	-3.33569200	-1.08323000
H	1.12015000	-1.69511300	-0.83551400
Na	-1.88802700	-1.37530900	-3.28459000
F	-3.18683700	-1.37436100	-1.58211200
Br	0.00248600	0.64680000	0.42785300

3a

C	-0.75427500	-0.39789500	-0.01510100
H	-1.58786900	-0.84349300	0.53995600
H	-0.68483600	-0.91648700	-0.97870900
C	0.48248400	-0.62014500	0.73564200
N	1.46603400	-0.78062300	1.32978100
C	-1.16867100	2.36147200	2.10086700
C	-1.11959200	1.82200000	1.01722700
C	-1.18567700	2.97562400	3.38885400
C	-2.10429400	3.99692200	3.69203700
C	-0.27275600	2.55680500	4.37534600
C	-2.10678000	4.58481000	4.95424800
H	-2.80774400	4.31925100	2.93089600
C	-0.28323300	3.14994900	5.63467400
H	0.43448800	1.76881800	4.13683100
C	-1.19820300	4.16409200	5.92815000
H	-2.81938800	5.37330400	5.17897100
H	0.42511500	2.82059700	6.38939900
H	-1.20304600	4.62483200	6.91180300
C	-2.33803800	1.26833800	-1.07958400
H	-3.19369000	0.85421100	-0.53581100

H	-2.53869900	2.32546900	-1.27390900
H	-2.24955600	0.74913600	-2.04041100
C	-1.04653300	1.11008700	-0.25774600
H	-0.20877700	1.51533800	-0.84214200

CAT^{doublet}

O	0.40767600	1.56273400	-0.43648600
C	0.10736700	0.64926700	0.51966600
N	-1.02226700	0.78396400	1.10876300
O	1.56456500	-2.58269700	1.67301200
C	0.64424000	-1.60258200	1.50016800
N	-0.53595300	-1.83576900	1.93735000
C	1.18274600	-0.37759400	0.79763300
C	1.82702000	-0.84125100	-0.55223200
H	2.68025400	-1.47847900	-0.30627300
H	2.20340900	0.04623600	-1.06328000
C	0.85126200	-1.59445500	-1.43089400
C	0.71851500	-2.98319700	-1.31697200
C	0.02539800	-0.91558500	-2.33894400
C	-0.23425100	-3.67858400	-2.05755400
H	1.35628800	-3.52559700	-0.62792700
C	-0.92518000	-1.60348600	-3.08644000
H	0.13782700	0.15608300	-2.46138100
C	-1.06390900	-2.98637500	-2.93646900
H	-0.34666600	-4.74826500	-1.93211400
H	-1.55891400	-1.07038700	-3.78835900
C	2.26340200	0.23839100	1.76798000
H	3.04666900	-0.51722300	1.87801700
H	1.78272300	0.35709600	2.74465500

C	2.85047100	1.55698100	1.33774700
C	3.91902300	1.62138300	0.43445800
C	2.32555600	2.75004300	1.84606600
C	4.43138400	2.84908900	0.02423900
H	4.35959200	0.70397500	0.05472200
C	2.83488300	3.98161200	1.44474300
H	1.50337700	2.71758400	2.55434200
C	3.88520500	4.03243900	0.52697900
H	5.26171100	2.89092200	-0.67246100
H	2.42377200	4.89934000	1.85078900
C	-1.65411800	3.08732500	1.70321400
C	-0.73837300	4.14370700	1.74135200
C	-2.57866500	2.94828500	2.74756500
C	-0.74229400	5.04736400	2.80612800
H	-0.00421400	4.26905800	0.95317600
C	-2.57661400	3.84644200	3.81356200
H	-3.28263700	2.12032000	2.74111400
C	-1.65970300	4.89938700	3.84631400
H	-0.02989100	5.86807000	2.81761200
H	-3.29492800	3.72026500	4.61811600
H	-1.66268300	5.60199000	4.67478200
C	-1.38401500	-4.09017600	1.36071100
C	-2.41944300	-3.50871900	0.61864400
C	-1.11639100	-5.45300800	1.18662500
C	-3.14574300	-4.26494100	-0.29891400
H	-2.65565100	-2.45856300	0.75540600
C	-1.84299000	-6.21120500	0.26593100
H	-0.34574300	-5.94444600	1.77448900

C	-2.85443400	-5.61649600	-0.48813900
H	-3.92628300	-3.78939100	-0.88276500
H	-1.61512300	-7.26611300	0.14121000
H	-3.40831100	-6.19797600	-1.21848100
C	-1.65621100	2.04067500	0.60226600
H	-2.68712300	1.80050000	0.33353200
C	-0.58827900	-3.25899500	2.36504500
H	-1.07061100	-3.30093400	3.34606600
C	-2.12515500	-3.69430800	-3.72811700
C	4.39714700	5.35695600	0.03603800
F	-2.08963300	-5.03287500	-3.56311500
F	-3.36740000	-3.27869000	-3.37710000
F	-2.00233400	-3.44605700	-5.05298500
F	5.72320800	5.31980400	-0.21999200
F	4.18024100	6.34560900	0.93038500
F	3.78719700	5.72635200	-1.11545900
Ni	-1.99054900	-0.52610800	2.21613600
C	-0.80979900	2.33002800	-0.64937600
H	-0.52994900	3.37077700	-0.79753200
H	-1.28446000	1.94241400	-1.55583700
C	0.91186700	-3.61098300	2.46877900
H	1.18887900	-4.57315800	2.04281100
H	1.29232800	-3.52822600	3.49102300
Cl	-3.93841300	-0.53117300	3.18383800

(*R*)-**TS1**^{doublet}

O	0.56230800	1.91496400	2.82557400
C	0.17353400	1.43662100	1.60676300
N	0.23862100	0.17262200	1.42946300

O	-1.16670300	2.88695400	-1.56143800
C	-0.72828200	1.94235200	-0.67599200
N	-0.72118400	0.72910900	-1.08101100
C	-0.30063100	2.50673000	0.65483900
C	-1.52379000	3.25739700	1.29499300
H	-1.73130400	4.13686700	0.68016700
H	-1.21371900	3.60135700	2.28544700
C	-2.74544400	2.37687000	1.38277700
C	-2.85367400	1.40214300	2.38283100
C	-3.75393900	2.46106800	0.41473300
C	-3.92244300	0.51081800	2.39733200
H	-2.08997200	1.33648900	3.15145300
C	-4.83297300	1.58084200	0.42824000
H	-3.68116400	3.21398800	-0.36433700
C	-4.90914000	0.59376100	1.41314800
H	-3.98552300	-0.25529500	3.16183800
H	-5.59913700	1.64092200	-0.33721200
C	0.85912300	3.54040800	0.42931200
H	1.01055600	4.07904700	1.36793600
H	0.51455200	4.25820200	-0.32052100
C	2.14152200	2.87322600	-0.00232200
C	3.19363100	2.70417800	0.90342500
C	2.27541600	2.34350100	-1.29401800
C	4.33886200	1.99276400	0.54630300
H	3.10605900	3.11644600	1.90451300
C	3.40475700	1.61715800	-1.65128400
H	1.48440700	2.49417700	-2.02110500
C	4.43136400	1.42373600	-0.72304300

H	5.14223800	1.85294400	1.26089100
H	3.48596400	1.18725800	-2.64381100
C	1.83322800	-1.43478600	2.41144200
C	1.63910800	-2.80037500	2.62843900
C	3.03713100	-0.99781500	1.85025400
C	2.62861900	-3.71856500	2.27136700
H	0.69688100	-3.14809200	3.04274200
C	4.02865700	-1.91053900	1.49405300
H	3.18203200	0.05701800	1.64781300
C	3.82071000	-3.27706800	1.69528500
H	2.45968300	-4.78035500	2.42436800
H	4.94619800	-1.55760700	1.03467300
H	4.57503100	-3.99298100	1.38545900
C	-2.20095500	-0.37397400	-2.74371200
C	-3.18217100	-0.65869400	-1.78752500
C	-2.18913400	-1.08124000	-3.94807000
C	-4.14543300	-1.63447300	-2.03816700
H	-3.17510100	-0.13215700	-0.84062800
C	-3.15023400	-2.06172200	-4.19933500
H	-1.41686100	-0.87519100	-4.68561800
C	-4.13060700	-2.33781600	-3.24493800
H	-4.90507100	-1.83996100	-1.29145600
H	-3.12766500	-2.61273700	-5.13512700
H	-4.87746200	-3.10258600	-3.43829500
C	0.72453500	-0.44809300	2.68934600
H	-0.12761100	-0.98003000	3.12377600
C	-1.18317300	0.70905100	-2.48698700
H	-0.30043600	0.52777800	-3.10973600

C	-6.08015400	-0.34661900	1.43787300
C	5.63042300	0.60984400	-1.11877900
F	-6.49156500	-0.66320300	0.18480500
F	-5.78934600	-1.50130600	2.07108900
F	-7.14731200	0.19598000	2.06758800
F	6.46385400	1.29762400	-1.93017500
F	5.26774600	-0.50870200	-1.78379900
F	6.35481400	0.22185900	-0.04150700
Ni	-0.14710600	-0.95464000	-0.18892500
C	1.11714300	0.78015300	3.54617400
H	2.19955500	0.91833300	3.61021500
H	0.68692000	0.77643700	4.54857200
C	-1.72186600	2.14698600	-2.68284200
H	-2.81248900	2.18525700	-2.60611200
H	-1.39383300	2.63097800	-3.60331200
H	-0.67161600	-2.24979000	0.55415300
C	0.15050500	-2.92062900	-0.66979300
H	-0.72512500	-3.48413600	-0.98492100
H	0.79369000	-3.48769400	0.00226800
C	0.77205200	-2.06407600	-1.64535100
H	0.31397900	-2.05128200	-2.63226200
C	2.28595400	-1.88905900	-1.64734800
H	2.64603000	-1.46331700	-0.70581100
C	3.03550900	-3.14561900	-1.84338100
N	3.59839900	-4.15660200	-1.95446500
H	2.59115800	-1.18719200	-2.43127100

IM1^{doublet}

O	-0.60026400	2.64933700	-1.68002800

C	-0.33552300	1.58183500	-0.87848000
N	-0.50597100	0.42135800	-1.37977300
O	0.65934400	1.08794300	2.68160600
C	0.38925400	0.76068700	1.38717600
N	0.47702300	-0.47671700	1.06835100
C	0.06567300	1.95427200	0.52545200
C	1.31957100	2.89490100	0.49978800
H	1.37005500	3.40514500	1.46488800
H	1.14342700	3.65237200	-0.26922800
C	2.61151200	2.15328200	0.25271500
C	2.89750400	1.58267600	-0.99497400
C	3.52606500	1.97280100	1.29755900
C	4.06323300	0.84816700	-1.19238800
H	2.20154700	1.70082100	-1.81868300
C	4.70154300	1.25042000	1.10595800
H	3.30960200	2.39905400	2.27290500
C	4.96857600	0.68404000	-0.14099500
H	4.25919700	0.38767100	-2.15434600
H	5.39951400	1.11088800	1.92370200
C	-1.14107500	2.73365800	1.16282000
H	-1.27251900	3.65567000	0.59083500
H	-0.85061800	3.00445500	2.18103200
C	-2.41241200	1.92030700	1.16444100
C	-2.66636200	0.99969900	2.19121800
C	-3.32270000	2.01704900	0.10766100
C	-3.79586200	0.18961700	2.15393700
H	-1.97230900	0.92260400	3.02292600
C	-4.45528000	1.20746200	0.06117000

H	-3.13425500	2.71748000	-0.69979100
C	-4.68916300	0.29006700	1.08296600
H	-3.98865200	-0.51829200	2.95392700
H	-5.13517400	1.27065400	-0.77880400
C	-2.53786800	0.08294800	-2.73427700
C	-2.86119400	-1.06527400	-1.99778100
C	-3.54118900	0.70568200	-3.48358800
C	-4.16390600	-1.55705300	-1.98957000
H	-2.08143300	-1.57159500	-1.43575600
C	-4.84907100	0.21256800	-3.47426200
H	-3.32040700	1.57457800	-4.09674200
C	-5.16696800	-0.91553300	-2.71944500
H	-4.40017100	-2.43288300	-1.39499500
H	-5.61518900	0.71397300	-4.05939200
H	-6.18560300	-1.29060600	-2.69584600
C	1.92424500	-2.24438100	2.08088400
C	3.00355200	-1.94175700	1.24334000
C	1.89584400	-3.46964700	2.75124700
C	4.04324800	-2.85529900	1.07964600
H	3.01536500	-1.00292400	0.70357100
C	2.93960700	-4.38265000	2.59382600
H	1.05068400	-3.71472000	3.39046200
C	4.01380100	-4.07588200	1.75744200
H	4.86580200	-2.61861100	0.41393600
H	2.90740500	-5.33503600	3.11519100
H	4.82168000	-4.78995100	1.62579300
C	-1.09717400	0.58461400	-2.73022200
H	-0.50170100	-0.02286600	-3.41806100

C	0.81961300	-1.23998600	2.29553400
H	-0.09065600	-1.76319900	2.61033100
C	6.24343800	-0.07760200	-0.36619400
C	-5.87914000	-0.62494500	1.05006900
F	7.20797400	0.70254600	-0.90428500
F	6.73895700	-0.58353200	0.78505200
F	6.06121500	-1.11576200	-1.21620500
F	-6.73815400	-0.32526300	0.05373400
F	-6.57321300	-0.57475500	2.21199600
F	-5.50930700	-1.91903000	0.88582100
Ni	0.34251700	-1.30973900	-0.72503700
C	-0.89568600	2.09519900	-2.99362000
H	-1.76827300	2.62183800	-3.37497000
H	-0.03424800	2.29538600	-3.63769700
C	1.18583400	-0.11848700	3.29683100
H	2.26862800	0.00044700	3.39479000
H	0.72739200	-0.22628000	4.28027100
H	0.47064900	-2.27241200	-1.95076100

(*R*)-**IM2**^{doublet}

O	-0.77160100	-0.51878400	-3.37493400
C	-0.32982700	-0.09414400	-2.15382800
N	-0.22987200	-0.98360400	-1.23550900
O	0.94354800	3.11253900	-0.73409600
C	0.54142100	1.81188200	-0.76869700
N	0.66829400	1.13142800	0.30288000
C	-0.03329800	1.38394800	-2.09539400
C	0.99363000	1.74685100	-3.22419900
H	1.02527300	2.83576900	-3.30449000

H	0.60171500	1.34975000	-4.16434500
C	2.37435800	1.20687400	-2.93815800
C	2.66321300	-0.15542100	-3.10824800
C	3.37337200	2.04663900	-2.43442400
C	3.90646100	-0.66763800	-2.75460900
H	1.90816000	-0.81513400	-3.52437600
C	4.62564000	1.54249900	-2.08482500
H	3.16223700	3.10302000	-2.30202500
C	4.88793400	0.18197200	-2.23435300
H	4.11906400	-1.72441600	-2.88101000
H	5.38992300	2.19941700	-1.68627600
C	-1.36733600	2.17684100	-2.34706200
H	-1.67943900	1.97831600	-3.37576500
H	-1.13371400	3.24189700	-2.26230900
C	-2.44632600	1.77314900	-1.37509700
C	-3.38730900	0.79932400	-1.72677300
C	-2.46590200	2.29074700	-0.07332500
C	-4.29938700	0.31756400	-0.79077100
H	-3.39401700	0.40561000	-2.73907400
C	-3.36772900	1.81017500	0.87037900
H	-1.75845000	3.06614300	0.20533200
C	-4.27365200	0.80823000	0.51517900
H	-5.01676900	-0.44725100	-1.06681100
H	-3.36520800	2.20173200	1.88138700
C	-1.41781500	-3.16986800	-0.96068900
C	-1.20094100	-4.54924800	-0.91737800
C	-2.44809600	-2.61188200	-0.19382100
C	-2.00927100	-5.36655600	-0.12525900

H	-0.39108200	-4.98561700	-1.49774300
C	-3.25098400	-3.42583300	0.60381800
H	-2.60152200	-1.53975100	-0.19850300
C	-3.03483500	-4.80513100	0.63617600
H	-1.82916600	-6.43722600	-0.09494800
H	-4.02901200	-2.97496500	1.21029100
H	-3.65553300	-5.43825200	1.26354300
C	2.44135500	1.34941400	2.02397400
C	3.43645800	0.72900500	1.26041900
C	2.53795500	1.34834900	3.41684000
C	4.52497400	0.12229300	1.88297300
H	3.34461600	0.69908500	0.18010000
C	3.62927600	0.74240300	4.04207600
H	1.74550100	1.78475400	4.01899800
C	4.62383000	0.13218100	3.27719800
H	5.28337100	-0.36669000	1.28017900
H	3.68824900	0.73407200	5.12625600
H	5.46853400	-0.34605900	3.76499000
C	-0.55798200	-2.29996500	-1.84265200
H	0.39754300	-2.80793000	-2.02349900
C	1.26700100	2.00437300	1.34050400
H	0.48942900	2.20966600	2.08267800
C	6.21190400	-0.40234200	-1.83242000
C	-5.15951800	0.20138700	1.56409600
F	7.06971500	0.53181600	-1.37618700
F	6.06261100	-1.32601500	-0.84911500
F	6.80925200	-1.03825500	-2.86472800
F	-5.53449900	1.09932700	2.49600000

F	-4.51679900	-0.80123800	2.22459000
F	-6.27708700	-0.34069200	1.03581100
Ni	-0.03543000	-0.74284900	0.70438900
C	-1.21458200	-1.88945500	-3.17796200
H	-2.30683300	-1.88751000	-3.10735100
H	-0.89514000	-2.47644600	-4.03914600
C	1.62215800	3.28522300	0.54298200
H	2.69441400	3.35832600	0.34286700
H	1.26014400	4.20957200	0.99426800
H	-1.53601800	-2.68916100	2.80934900
C	-0.56102600	-2.35318500	3.18745200
H	0.17861600	-3.09482700	2.86162300
H	-0.60628400	-2.40276200	4.29013400
C	-0.19881300	-0.95546700	2.66436400
H	0.79982300	-0.71120500	3.05375900
C	-1.18096200	0.09098500	3.25531400
H	-2.21392900	-0.26853800	3.17538400
C	-0.89845600	0.42667200	4.66154300
N	-0.58065800	0.70793000	5.74494100
H	-1.15433200	1.03750300	2.70000500

(*R*)-**TS2**^{doublet}

O	-0.45371800	-1.54697500	-2.81991900
C	-0.07755800	-0.82692100	-1.74590900
N	-0.20010500	-1.38877100	-0.60423300
O	1.38138500	2.54262600	-1.15880000
C	0.91152200	1.31532000	-0.84929100
N	0.97716500	0.96605700	0.38168100
C	0.42517300	0.55637600	-2.06224900

C	1.62380900	0.46277700	-3.07562800
H	1.68928500	1.42600600	-3.58746500
H	1.35542600	-0.29149200	-3.82081000
C	2.95248800	0.14290800	-2.43097200
C	3.97331300	1.10271900	-2.42261200
C	3.18174400	-1.08730300	-1.80397700
C	5.19841600	0.83708900	-1.81495800
H	3.80340800	2.06735800	-2.89237100
C	4.40192400	-1.36254900	-1.19276100
H	2.41184500	-1.84818300	-1.75967500
C	5.41083000	-0.39801600	-1.19767200
H	5.98031500	1.58844100	-1.80608100
H	4.53683700	-2.31816400	-0.69885500
C	-0.74386000	1.34830200	-2.75051800
H	-0.87346500	0.92517600	-3.74993700
H	-0.41107200	2.38342900	-2.86271600
C	-2.03566600	1.26659200	-1.97661100
C	-3.04173700	0.37705400	-2.37274200
C	-2.21977700	2.01970200	-0.80910600
C	-4.19557900	0.21520800	-1.60691300
H	-2.91212900	-0.20472300	-3.28056700
C	-3.36913300	1.86699800	-0.04182100
H	-1.46113500	2.72390700	-0.48375200
C	-4.34911600	0.95066600	-0.43188100
H	-4.96246900	-0.48845900	-1.91010200
H	-3.48864200	2.44647600	0.86580800
C	-1.68673500	-3.22009600	0.17444200
C	-2.80727500	-2.42110400	0.43148600

C	-1.52751900	-4.42564300	0.86285200
C	-3.76641400	-2.83042200	1.35681000
H	-2.91953100	-1.47311900	-0.08385100
C	-2.49055500	-4.84019800	1.78430700
H	-0.63779000	-5.02458300	0.68965300
C	-3.61076500	-4.04501900	2.03023800
H	-4.62374400	-2.19685400	1.55640400
H	-2.35692600	-5.77570700	2.31939500
H	-4.35551700	-4.36337600	2.75395500
C	2.61507500	1.65049600	2.13356200
C	3.72567200	0.92028500	1.70020200
C	2.47095400	1.94761000	3.49166400
C	4.68643800	0.49231600	2.61330100
H	3.82475800	0.65735200	0.65350600
C	3.43352300	1.52218600	4.40806700
H	1.60031000	2.50439200	3.82835600
C	4.54115500	0.79456700	3.96912100
H	5.53363600	-0.08912100	2.26694400
H	3.31494600	1.75389300	5.46266400
H	5.28696000	0.45644700	4.68273400
C	-0.63946700	-2.79019100	-0.81815500
H	0.24964300	-3.41075700	-0.68566100
C	1.56463100	2.10173700	1.15369700
H	0.73932300	2.56916500	1.69249800
C	6.73460300	-0.70000600	-0.55443800
C	-5.52172200	0.72971300	0.47761300
F	6.59224500	-1.47449600	0.54627900
F	7.56385400	-1.36008100	-1.39288100

F	7.37525300	0.42854200	-0.17093700
F	-6.44341300	-0.09769500	-0.05495600
F	-6.13914800	1.88352900	0.80091700
F	-5.11764200	0.16836200	1.65277200
Ni	0.39450200	-0.78595500	1.25899700
C	-1.07522000	-2.76728900	-2.29835200
H	-2.15709400	-2.65257600	-2.41274000
H	-0.70811300	-3.60561600	-2.88546100
C	2.06914200	3.03027400	0.03173900
H	3.14249500	2.92917500	-0.14930600
H	1.79955700	4.07708400	0.16828600
H	0.28512600	-2.48959800	3.86668500
C	-0.64056400	-1.92345100	3.73780600
H	-1.10862800	-1.81026600	4.73105100
H	-1.31784800	-2.53787200	3.13329100
C	-0.36831300	-0.56158900	3.08692500
H	0.36119600	-0.01033900	3.69238400
C	-1.69560900	0.23569700	3.07004300
H	-2.41992700	-0.20520500	2.37184400
H	-2.18188900	0.20848200	4.06047600
C	-1.52818300	1.64270200	2.71588900
N	-1.37066800	2.75986400	2.43443500
C	2.34268600	-4.41245900	-1.02819300
C	2.82728800	-4.05554500	0.05038600
C	1.77778600	-4.84326100	-2.26200100
C	0.81184700	-5.87339500	-2.28833700
C	2.09763800	-4.19769500	-3.47634400
C	0.17645300	-6.22379400	-3.47658100

H	0.56646600	-6.38174200	-1.36120600
C	1.45638000	-4.55259100	-4.66041400
H	2.84749100	-3.41362400	-3.47210900
C	0.48926300	-5.56167400	-4.66815800
H	-0.56726100	-7.01606400	-3.47420800
H	1.71281200	-4.03928600	-5.58327400
H	-0.00949300	-5.83511000	-5.59340000
Br	2.26735900	-2.37918200	1.68120500

(*R*)-**IM3**^{triplet}

O	-1.21562400	-0.20231800	-3.09071900
C	-0.71484100	0.16402200	-1.88985800
N	-0.69753200	-0.72784600	-0.97291800
O	0.69834600	3.30637600	-0.46697500
C	0.31633500	2.01092000	-0.51645200
N	0.55303200	1.30018400	0.51991200
C	-0.26508000	1.60323400	-1.84717800
C	0.84061200	1.85019700	-2.94018900
H	0.86004500	2.92557900	-3.13366700
H	0.49990100	1.35614800	-3.85431900
C	2.22209400	1.38313500	-2.55040100
C	3.21949100	2.32318200	-2.25681500
C	2.52686100	0.02206000	-2.43805600
C	4.49402000	1.91594600	-1.87174500
H	2.99080200	3.38259900	-2.32872600
C	3.79537600	-0.39517400	-2.04248600
H	1.77024700	-0.73025800	-2.63250000
C	4.78071800	0.55315400	-1.76304800
H	5.25790700	2.64988400	-1.64024600

H	3.99552600	-1.45201700	-1.91563600
C	-1.49484600	2.51033700	-2.21545400
H	-1.62009500	2.45253300	-3.29953400
H	-1.21829300	3.53890300	-1.96787200
C	-2.78159900	2.11344100	-1.53522200
C	-2.97308800	2.33824700	-0.16688200
C	-3.79173800	1.47087500	-2.26270700
C	-4.13778500	1.91244400	0.46560700
H	-2.21164800	2.83328800	0.42515900
C	-4.96642200	1.05405300	-1.64033500
H	-3.65041500	1.28787800	-3.32372900
C	-5.13344000	1.26585900	-0.27041600
H	-4.25499800	2.07103000	1.53117200
H	-5.73823800	0.54661600	-2.20820700
C	-2.26125100	-2.63352600	-0.65342600
C	-3.37902600	-1.90188600	-0.23607100
C	-2.11458800	-3.96020800	-0.24387100
C	-4.34433100	-2.49162400	0.57802000
H	-3.48538800	-0.86467000	-0.53432400
C	-3.08417300	-4.55535300	0.56588600
H	-1.22844800	-4.51577400	-0.53728000
C	-4.19781400	-3.82277000	0.97768100
H	-5.20128000	-1.91218300	0.90441400
H	-2.95959700	-5.58562500	0.88580800
H	-4.94779200	-4.28389800	1.61378800
C	2.34277100	1.49695800	2.22288000
C	3.30262100	0.81842800	1.46437200
C	2.45245700	1.51575500	3.61654000

C	4.36252200	0.16530700	2.09063100
H	3.20736100	0.77390100	0.38640900
C	3.51858400	0.87112300	4.24453600
H	1.69511900	2.02501100	4.20726100
C	4.47290400	0.19460900	3.48195300
H	5.08731800	-0.37428100	1.49168600
H	3.59633900	0.88677900	5.32785400
H	5.29535700	-0.31867700	3.97163600
C	-1.22243500	-1.99686600	-1.54122200
H	-0.36431100	-2.66863800	-1.63134800
C	1.17659700	2.17338400	1.55083300
H	0.40108300	2.38947800	2.28801600
C	6.15836400	0.10413600	-1.36515500
C	-6.38769600	0.81135900	0.42001900
F	6.79985200	1.04069700	-0.63112800
F	6.12317000	-1.03094100	-0.62875600
F	6.93309200	-0.15326500	-2.44311300
F	-6.99808900	-0.19016200	-0.25441700
F	-7.28801600	1.80838300	0.55328400
F	-6.12513600	0.34891300	1.66678200
Ni	0.41721100	-0.73484200	0.74586600
C	-1.74875500	-1.54822600	-2.92537600
H	-2.83871300	-1.47598700	-2.95605000
H	-1.38715500	-2.15453200	-3.75618300
C	1.51133100	3.44201900	0.73929200
H	2.55814800	3.47493000	0.42474300
H	1.22633900	4.37174600	1.23037600
H	0.39256400	-3.00654600	2.76613600

C	-0.58680400	-2.52553400	2.83780700
H	-0.97931200	-2.70110600	3.85528600
H	-1.25348000	-3.04397900	2.14035000
C	-0.48901100	-1.03352900	2.51617500
H	0.20798800	-0.56245800	3.22803800
C	-1.87860300	-0.39081600	2.71034200
H	-2.55434400	-0.66822300	1.89242700
H	-2.35913200	-0.75237600	3.63627000
C	-1.84507900	1.06592400	2.79894300
N	-1.79536300	2.22507900	2.88049600
Br	1.91519800	-2.59313200	0.11142700

(*R*)-**TS3**^{doublet}

O	-0.91768300	-3.58397300	-0.53190500
C	-0.96821700	-2.38288400	0.08803200
N	-0.35791900	-1.41236600	-0.48285800
O	-2.94594300	-1.02215600	2.92844000
C	-2.04961500	-1.03289300	1.91943100
N	-1.59371100	0.09844800	1.53942300
C	-1.74993500	-2.40873900	1.37873100
C	-3.12682500	-3.11984700	1.12573700
H	-3.55910400	-3.34618300	2.10266500
H	-2.91476400	-4.06757900	0.62531300
C	-4.09300600	-2.28180600	0.31753900
C	-5.22053200	-1.72067300	0.92871600
C	-3.86331600	-2.00986700	-1.03948300
C	-6.09461700	-0.90378300	0.21314000
H	-5.41013800	-1.92017200	1.97872500
C	-4.72082300	-1.18171600	-1.75686000

H	-3.01280200	-2.45496000	-1.54407700
C	-5.83818600	-0.62482800	-1.12901700
H	-6.96128600	-0.47052500	0.69922900
H	-4.52032800	-0.96149800	-2.80002100
C	-0.92843500	-3.24037400	2.43704800
H	-1.16054100	-4.29530400	2.26715200
H	-1.31303800	-2.97712300	3.42725500
C	0.56631800	-3.03394300	2.34445000
C	1.36881800	-4.04913700	1.80411000
C	1.16992100	-1.82791000	2.71975900
C	2.73999600	-3.86829400	1.65244200
H	0.91178700	-4.98656400	1.49952000
C	2.54201600	-1.62889700	2.55258900
H	0.57757000	-1.01532500	3.12757100
C	3.32602100	-2.65404700	2.02311700
H	3.35337500	-4.65933100	1.23317900
H	2.98301900	-0.66752800	2.78900400
C	1.49489500	-1.39672800	-2.18395600
C	1.71702000	-0.88814200	-3.46727400
C	2.54957800	-1.44512900	-1.26691900
C	2.98295800	-0.42495400	-3.82841100
H	0.89532900	-0.83581900	-4.17718000
C	3.81246000	-0.98528300	-1.62954100
H	2.38055400	-1.82511500	-0.26592700
C	4.03132700	-0.46959600	-2.90839100
H	3.14363600	-0.01232200	-4.81987300
H	4.61885700	-1.01024600	-0.90942000
H	5.01038600	-0.08484000	-3.17305600

C	-2.79851500	2.24117700	1.44506700
C	-3.71000600	1.90292700	0.43906400
C	-2.36286700	3.56218000	1.56582000
C	-4.18559300	2.87586800	-0.43680500
H	-4.02602100	0.87200400	0.32204000
C	-2.83960000	4.53921700	0.69011000
H	-1.62770400	3.81899300	2.32317300
C	-3.74869800	4.19716400	-0.31168000
H	-4.87861000	2.59968100	-1.22428000
H	-2.48742000	5.56221800	0.78137000
H	-4.10819800	4.95523400	-1.00113100
C	0.13476700	-1.90747800	-1.80078600
H	-0.59872100	-1.57123600	-2.54256900
C	-2.23708600	1.16922600	2.34603500
H	-1.46206700	1.60029800	2.98427100
C	-6.77228400	0.24490400	-1.92293600
C	4.81067600	-2.49054400	1.84099900
F	-7.58109700	-0.48710400	-2.71926700
F	-7.56083800	0.99789600	-1.12930500
F	-6.08817500	1.08867500	-2.73420300
F	5.19532300	-2.91033400	0.60678800
F	5.50080400	-3.24175800	2.73071300
F	5.21827600	-1.21907300	1.97809000
Ni	-0.01424700	0.45304200	0.32368100
C	0.05012900	-3.43181600	-1.61356200
H	0.99783700	-3.85721700	-1.27236700
H	-0.32555400	-3.97495000	-2.47987100
C	-3.27441600	0.37786100	3.17819000

H	-4.29925900	0.53738800	2.83519800
H	-3.20344900	0.54912400	4.25230500
H	-2.14821800	0.97900400	-1.85613800
C	-1.20182800	1.37057800	-2.24675400
H	-1.44748200	2.19836500	-2.93005100
H	-0.73574600	0.58833500	-2.85889700
C	-0.28185700	1.81279300	-1.11543300
H	-0.73714600	2.65100100	-0.57583000
C	1.11872900	2.24067800	-1.60179000
H	1.74484100	2.54905800	-0.75741700
H	1.64329300	1.40731400	-2.08285700
C	1.07737800	3.34529700	-2.57079300
N	1.01159700	4.21391300	-3.34051600
Br	1.15403700	1.82909500	2.12213200
C	3.48721800	1.37913200	1.22881500
C	4.20169800	1.69716600	0.27905900
C	5.13810300	1.88624300	-0.78032200
C	6.35247700	1.17058600	-0.79069600
C	4.84716800	2.74096900	-1.86099800
C	7.23802500	1.29605700	-1.85784500
H	6.57255500	0.50892000	0.04162200
C	5.73913800	2.86278600	-2.92381300
H	3.91524000	3.29624000	-1.86656300
C	6.93505100	2.14024500	-2.93079200
H	8.16818700	0.73393200	-1.85336200
H	5.49495300	3.52234900	-3.75192700
H	7.62752600	2.23692700	-3.76233100

(*R*)-**IM4**^{doublet}

O	1.68581300	3.34711100	0.95876200
C	1.11924000	2.30283900	0.31224200
N	1.09369100	1.18672000	0.93664200
O	-0.54031000	1.84669300	-2.98460500
C	-0.15736000	1.54917500	-1.72304000
N	-0.57153900	0.44466600	-1.23046700
C	0.63133600	2.65576500	-1.06918700
C	-0.31391000	3.91490600	-1.00109200
H	-0.38176400	4.30623200	-2.01895700
H	0.20162800	4.66191300	-0.39219900
C	-1.69873200	3.64816400	-0.46643600
C	-1.91177100	3.40316800	0.89454500
C	-2.79629200	3.62435900	-1.33663700
C	-3.18927100	3.13964800	1.37799200
H	-1.07611900	3.40456900	1.58610500
C	-4.08038400	3.37413300	-0.85947300
H	-2.64098100	3.80659000	-2.39627300
C	-4.27538400	3.13190000	0.50099500
H	-3.33335000	2.91420100	2.42798100
H	-4.92398300	3.34962400	-1.53961700
C	1.86537600	3.08042500	-1.94562800
H	2.13770400	4.09130700	-1.63136000
H	1.51404900	3.14023400	-2.97870300
C	3.07134500	2.18192000	-1.83965300
C	3.17079700	1.00833700	-2.59222700
C	4.12053900	2.51386500	-0.97000100
C	4.27586600	0.16640800	-2.46427800
H	2.37763700	0.75018100	-3.28749400

C	5.23190500	1.68693500	-0.84677000
H	4.06311100	3.43223200	-0.39342900
C	5.30416300	0.50295000	-1.58607500
H	4.33837900	-0.75060600	-3.03753300
H	6.04329900	1.95455200	-0.17703300
C	2.59864000	0.32052900	2.74088300
C	2.22975800	-0.53476600	3.78415400
C	3.82958800	0.13279000	2.10052400
C	3.08956400	-1.55922600	4.18808700
H	1.26008000	-0.41243500	4.25759800
C	4.68631300	-0.89047400	2.49921300
H	4.11010600	0.76650200	1.26627000
C	4.31719400	-1.73661600	3.54833700
H	2.79278700	-2.22293600	4.99478700
H	5.61776200	-1.04600900	1.96791400
H	4.97930300	-2.54206300	3.85144800
C	-2.76620700	-0.68446300	-1.76157100
C	-3.38931000	-1.75431200	-2.41261800
C	-3.42326500	-0.03313900	-0.71814400
C	-4.66142600	-2.16951900	-2.02620900
H	-2.86301800	-2.28987800	-3.19926400
C	-4.69271200	-0.45544700	-0.32340900
H	-2.92635000	0.76291500	-0.17968600
C	-5.31539400	-1.51802000	-0.97915500
H	-5.12175300	-3.02261000	-2.51513100
H	-5.18310000	0.03611100	0.50882900
H	-6.29907100	-1.85189400	-0.66148600
C	1.64972500	1.40811500	2.30653900

H	0.78130800	1.40642200	2.96927100
C	-1.40852400	-0.24113100	-2.24809700
H	-0.85605200	-1.12383600	-2.57636500
C	-5.65587600	2.88693000	1.04052200
C	6.53014800	-0.35819400	-1.44785500
F	-6.17812300	4.00194500	1.60203700
F	-6.51437500	2.48741300	0.07759000
F	-5.65435000	1.93353000	2.00148300
F	6.78770700	-0.63744200	-0.14798000
F	7.62501500	0.28798400	-1.92013800
F	6.42528100	-1.51656000	-2.11440100
Ni	0.11348500	-0.51335000	0.49939400
C	2.25881600	2.81782600	2.18818700
H	3.34495500	2.80574000	2.06983100
H	1.97915300	3.48751000	3.00138700
C	-1.49368700	0.81757900	-3.37895100
H	-2.47926000	1.28515000	-3.42716500
H	-1.20012900	0.44856300	-4.36240700
H	0.81522100	-2.69257200	-1.83240200
C	1.56244600	-1.89822400	-1.77040400
H	1.27515200	-1.09032900	-2.44642400
H	2.52153400	-2.29717800	-2.13064700
C	1.71690600	-1.40541300	-0.34471400
H	2.38845600	-0.54566800	-0.30852600
C	2.23514700	-2.50825900	0.59152000
H	2.22309400	-2.19501900	1.63871900
H	1.59732500	-3.39357500	0.51304300
C	3.61272700	-2.86166900	0.22813900

N	4.69830100	-3.09593500	-0.11278300
C	-0.69386700	-2.15506700	0.24165900
C	-1.35177900	-3.14546200	-0.03785100
C	-2.26820500	-4.18861800	-0.35844800
C	-2.05553100	-5.02803900	-1.46790600
C	-3.44970000	-4.34437700	0.39196300
C	-3.00339600	-5.98426000	-1.82508900
H	-1.14504500	-4.91163700	-2.04864100
C	-4.39143400	-5.30321500	0.03207400
H	-3.61988300	-3.68421600	1.23571300
C	-4.17577400	-6.12408600	-1.07872300
H	-2.82759200	-6.62207800	-2.68733000
H	-5.30249200	-5.40695400	0.61522800
H	-4.91475300	-6.86982500	-1.35856900
Br	-1.48028600	0.03952300	2.35615100

(*R*)-**TS4**^{doublet}

O	1.71972800	3.51187600	0.99157000
C	1.13755000	2.47160200	0.34645200
N	1.07475800	1.36190500	0.97484000
O	-0.47972000	2.04696500	-2.97806300
C	-0.08500400	1.70946100	-1.72916100
N	-0.42378000	0.55324500	-1.30093900
C	0.64426300	2.83139900	-1.03165100
C	-0.36093600	4.03753100	-0.92274300
H	-0.45661000	4.46531400	-1.92349700
H	0.10907400	4.78904900	-0.28296100
C	-1.72466800	3.65394800	-0.40010700
C	-1.91268700	3.28391400	0.93697000

C	-2.82550100	3.62911300	-1.26619800
C	-3.16768000	2.90226700	1.40181300
H	-1.07550700	3.27481000	1.62625600
C	-4.08784100	3.26240800	-0.80674300
H	-2.69007000	3.90211700	-2.30901300
C	-4.25838400	2.90146900	0.53046800
H	-3.28588800	2.57768800	2.42866000
H	-4.93349700	3.24083200	-1.48471300
C	1.86948900	3.29874300	-1.89190900
H	2.21845800	4.24483200	-1.47082300
H	1.49988400	3.49567000	-2.90126900
C	2.98920800	2.28981600	-1.91074700
C	2.91755500	1.15691100	-2.73057800
C	4.09099300	2.43542300	-1.05909800
C	3.90143000	0.17389200	-2.67333500
H	2.08253400	1.04309100	-3.41571200
C	5.07938900	1.45575800	-0.99726100
H	4.16269600	3.31636900	-0.42799400
C	4.97055300	0.30968000	-1.78646700
H	3.83052600	-0.70701100	-3.30000900
H	5.92024500	1.56697100	-0.32090700
C	2.55960700	0.48019500	2.77833700
C	2.25827500	-0.30310500	3.89535000
C	3.73336900	0.22779300	2.05789800
C	3.13368700	-1.31601900	4.29639900
H	1.32746900	-0.13794700	4.42881800
C	4.60309100	-0.78579900	2.45094200
H	3.95422800	0.80903700	1.16936700

C	4.30489100	-1.55752900	3.57759200
H	2.89020600	-1.92367100	5.16308100
H	5.48528800	-0.99795100	1.85784000
H	4.97440400	-2.35874400	3.87485800
C	-2.55810400	-0.65063900	-1.89683500
C	-3.16836500	-1.66019100	-2.64820800
C	-3.20802500	-0.13796600	-0.77388700
C	-4.41800600	-2.15521800	-2.28115900
H	-2.64960800	-2.08445400	-3.50508400
C	-4.45179000	-0.64405600	-0.39706800
H	-2.72839700	0.61637900	-0.16353800
C	-5.06026900	-1.64789100	-1.15133600
H	-4.86761000	-2.96220100	-2.85123500
H	-4.93169700	-0.26357800	0.49696900
H	-6.02221700	-2.04988200	-0.84727800
C	1.61736800	1.57555100	2.34718400
H	0.74647100	1.57686900	3.00754800
C	-1.21964100	-0.12273500	-2.35760000
H	-0.62301900	-0.96342800	-2.72327300
C	-5.62204300	2.54321100	1.04922700
C	6.02934100	-0.75646300	-1.71263300
F	-6.22897400	3.60507500	1.62904600
F	-6.44000000	2.10915200	0.06568300
F	-5.56307700	1.56951000	1.98675900
F	6.54783500	-0.85230200	-0.46730500
F	7.06197300	-0.48733800	-2.54547100
F	5.54446900	-1.96414900	-2.05859000
Ni	0.06674200	-0.36167700	0.50550600

C	2.25095500	2.97910900	2.23870400
H	3.33992100	2.94687300	2.15034700
H	1.96239500	3.65821900	3.04127600
C	-1.35602700	0.98038700	-3.43943200
H	-2.36933000	1.38425700	-3.48590900
H	-1.02261300	0.67897600	-4.43340600
H	1.20319800	-3.12021500	-1.74953700
C	1.75283200	-2.19549200	-1.55979400
H	1.39964600	-1.43104900	-2.25592200
H	2.81736300	-2.36644500	-1.76243100
C	1.58178400	-1.73604900	-0.12269400
H	2.17061300	-0.82536800	0.02880700
C	2.02797500	-2.77250800	0.91984500
H	1.91103900	-2.37605100	1.93310300
H	1.40896700	-3.67149500	0.84548400
C	3.43143800	-3.14878200	0.71240700
N	4.53624500	-3.43736900	0.50082900
C	-0.43900600	-2.06924200	0.06488500
C	-1.10539500	-3.05977700	-0.21574800
C	-2.09548800	-4.04833300	-0.47357900
C	-2.07837300	-4.80965300	-1.65749300
C	-3.15873600	-4.22633300	0.43383300
C	-3.10374100	-5.71019700	-1.93395200
H	-1.25982300	-4.67512400	-2.35852900
C	-4.17890900	-5.12898500	0.15160900
H	-3.17654100	-3.62591900	1.33733700
C	-4.15959100	-5.87052600	-1.03328800
H	-3.08027200	-6.28845800	-2.85385300

H	-4.99865900	-5.24985300	0.85456100
H	-4.96008500	-6.57210500	-1.25116400
Br	-1.35990600	-0.14379300	2.56096900

CAT'doublet

O	1.89390100	-2.54728000	-1.56157200
C	0.90726900	-1.63827100	-1.40207500
N	1.11110300	-0.70303300	-0.55627000
O	-2.70464000	-1.75387500	-2.03165100
C	-1.52370000	-1.21402300	-1.65862300
N	-1.57502700	-0.21284700	-0.85548800
C	-0.27478800	-1.71974900	-2.35762000
C	0.00824700	-0.67295300	-3.53527200
H	-0.47407800	0.26600500	-3.25507300
H	-0.48833800	-1.04414300	-4.43357000
C	1.47650700	-0.40716800	-3.75772300
C	2.05881000	0.72309000	-3.16546600
C	2.30232000	-1.31356700	-4.43395900
C	3.43763900	0.91144500	-3.19264600
H	1.43656400	1.43661400	-2.63252500
C	3.68212600	-1.13230300	-4.46809500
H	1.87491700	-2.19069800	-4.90918300
C	4.25170100	-0.02964000	-3.82704500
H	3.87467400	1.77591100	-2.70443600
H	4.31731000	-1.85015300	-4.97558300
C	-0.40051400	-3.19339400	-2.86739300
H	-0.64843400	-3.81944100	-2.00368400
H	0.60400200	-3.48914600	-3.17596400
C	-1.36236800	-3.50134500	-3.99684900

C	-2.62189000	-4.05905800	-3.73841700
C	-0.98806500	-3.31702100	-5.33476100
C	-3.49102200	-4.38710200	-4.77292600
H	-2.92611800	-4.22728800	-2.71146100
C	-1.85386300	-3.63398300	-6.38004400
H	-0.00183400	-2.93237800	-5.57337100
C	-3.11200600	-4.16460000	-6.09916500
H	-4.46557200	-4.81057100	-4.55418400
H	-1.55041300	-3.47339800	-7.40867100
C	2.27689700	-1.17691100	1.54359400
C	1.86193700	-2.43232800	2.00308500
C	2.46336700	-0.13795300	2.45979200
C	1.65344300	-2.65171300	3.36395800
H	1.69062800	-3.24136000	1.29651500
C	2.25264700	-0.35622300	3.82202800
H	2.74336400	0.84782400	2.09986300
C	1.85166400	-1.61300500	4.27684600
H	1.33606700	-3.63090100	3.71171300
H	2.39765400	0.45876300	4.52502600
H	1.69028000	-1.78340400	5.33755200
C	-3.23135100	1.47624400	-1.37850900
C	-3.36656700	1.53161400	-2.77176100
C	-3.17410900	2.66589800	-0.64786200
C	-3.45777000	2.76176100	-3.42073100
H	-3.38792200	0.61459500	-3.35570900
C	-3.25943800	3.89781300	-1.29701400
H	-3.03498000	2.62926300	0.42911100
C	-3.40380500	3.94725900	-2.68353700

H	-3.56534500	2.79567900	-4.50111000
H	-3.20021700	4.81514800	-0.71974200
H	-3.46767000	4.90561200	-3.19032900
C	2.44213700	-0.90471300	0.06485800
H	3.00429400	0.02104600	-0.07682900
C	-3.00619700	0.15687000	-0.67307800
H	-3.20565200	0.26559500	0.39509500
C	5.74348500	0.06959400	-3.70473600
C	-4.03269100	-4.56829700	-7.21566700
F	6.38368700	-0.51204600	-4.74189700
F	6.16975900	1.34499100	-3.62072500
F	6.16925800	-0.56369800	-2.57804400
F	-3.89022500	-5.87696800	-7.52894900
F	-3.79753300	-3.86456400	-8.34441200
F	-5.33039900	-4.38578900	-6.88336000
Ni	-0.05266900	0.87108400	-0.26582000
Br	1.00422900	2.89632600	0.25947600
C	3.03124600	-2.05451200	-0.78785700
H	3.42143800	-2.88779500	-0.20401200
H	3.78257100	-1.70877900	-1.50083800
C	-3.73419200	-1.06786400	-1.26364900
H	-4.54921600	-0.82193800	-1.94401700
H	-4.08584700	-1.75892200	-0.49274000

(*S*)-**TS1**^{doublet}

O	-0.73298700	3.00580400	1.65872400
C	-0.30502500	1.77029000	1.26121700
N	-0.38452800	1.50043100	0.01522400
O	1.32567400	-1.15062300	2.89893900

C	0.72654100	-0.42039400	1.91341000
N	0.63045400	-0.96798600	0.76133300
C	0.23384000	0.92399500	2.38536500
C	1.43371700	1.68713700	3.05356200
H	1.71101400	1.14391100	3.95980900
H	1.07444900	2.67754700	3.34461400
C	2.60823900	1.78575400	2.10976700
C	3.65120200	0.85209200	2.17044300
C	2.62268700	2.74150900	1.08626800
C	4.66010300	0.84511000	1.21199700
H	3.65477800	0.11184600	2.96392500
C	3.62511700	2.73847900	0.11964000
H	1.83330600	3.48530700	1.03887600
C	4.63668200	1.77891200	0.17312400
H	5.44944300	0.10184800	1.25300000
H	3.61491400	3.46744100	-0.68264300
C	-0.90721100	0.69801900	3.44244500
H	-1.13054800	1.66296400	3.90481000
H	-0.51047400	0.03327500	4.21504000
C	-2.14058700	0.11964100	2.79307700
C	-3.21146000	0.94925600	2.44223600
C	-2.18657900	-1.23160600	2.42348800
C	-4.28466100	0.45475700	1.70263900
H	-3.19353300	1.99583600	2.73289700
C	-3.24237600	-1.73139600	1.67010100
H	-1.37903000	-1.89875300	2.70619000
C	-4.28584600	-0.88006600	1.29670800
H	-5.10336900	1.10725300	1.42107000

H	-3.23221600	-2.76835700	1.35358700
C	-1.94450800	2.40449100	-1.70684800
C	-1.90833000	3.01341200	-2.96259400
C	-2.97547300	1.50728200	-1.40718600
C	-2.89412000	2.73330900	-3.91094200
H	-1.09723200	3.69520800	-3.20645800
C	-3.95804700	1.22156600	-2.35322500
H	-2.98315600	1.00191600	-0.44884400
C	-3.91961200	1.83651900	-3.60701300
H	-2.85379500	3.20663200	-4.88785300
H	-4.73628200	0.50459900	-2.11561000
H	-4.68095000	1.60931000	-4.34780900
C	2.12851400	-2.65844100	-0.28760300
C	2.99484400	-1.68783600	-0.80495700
C	2.14670400	-3.95149000	-0.81925600
C	3.87033300	-2.00881500	-1.84112700
H	2.96676600	-0.67834600	-0.41102000
C	3.02100100	-4.27246400	-1.85951700
H	1.46220300	-4.70011400	-0.42836400
C	3.88371000	-3.30138100	-2.37110400
H	4.54031900	-1.24810100	-2.22795200
H	3.02426000	-5.27794500	-2.27096600
H	4.56328300	-3.54997500	-3.18148800
C	-0.88347800	2.71042400	-0.68110000
H	-0.01948000	3.16249300	-1.18159400
C	1.20647800	-2.33179400	0.85924800
H	0.36753600	-3.03088800	0.86324200
C	5.72884500	1.75871500	-0.85714300

C	-5.36009900	-1.40663900	0.39191900
F	6.87691300	2.29531200	-0.38313600
F	6.02704900	0.49076300	-1.23652400
F	5.39621500	2.44581600	-1.96863000
F	-4.88793800	-1.59583600	-0.87122000
F	-6.40827100	-0.55965400	0.28817800
F	-5.83554000	-2.59896800	0.80445100
Ni	-0.11534700	-0.26506200	-0.95535600
C	-1.37083600	3.59202500	0.49262900
H	-2.45422200	3.52199900	0.62846900
H	-1.06761600	4.63757600	0.42934100
C	1.90013800	-2.31084400	2.24018300
H	2.97963000	-2.15203100	2.15667200
H	1.69439700	-3.18690400	2.85563000
H	0.48545300	0.33391400	-2.29129500
C	-0.67362200	-0.65727300	-2.87399000
H	-1.24925300	0.14278100	-3.33385000
H	0.04898400	-1.09502600	-3.56339300
C	-1.34675000	-1.54933200	-1.96098100
H	-2.37733000	-1.31072000	-1.70893100
C	-1.08285700	-3.04281000	-2.07901700
H	-1.68032400	-3.53015500	-2.87043500
H	-0.03000500	-3.23098700	-2.32290800
C	-1.37114400	-3.75268000	-0.82785200
N	-1.59738900	-4.27998200	0.18384900

TS1'^{doublet}

O	-10.17495600	-5.19900800	-5.43436900
C	-11.19891700	-4.33096300	-5.24847100

N	-11.03899100	-3.41112600	-4.37258800
O	-14.74915300	-4.16542300	-6.39397600
C	-13.61875300	-3.83694100	-5.69992600
N	-13.72969300	-2.92506700	-4.80823200
C	-12.39192500	-4.59954600	-6.13419100
C	-12.09632300	-4.11790700	-7.61135500
H	-12.11632300	-3.02305700	-7.59617400
H	-12.94115300	-4.45130000	-8.22073200
C	-10.79202500	-4.58508000	-8.20130300
C	-9.64074300	-3.79598400	-8.07060300
C	-10.69416500	-5.80770600	-8.87374400
C	-8.41704100	-4.23099400	-8.56598700
H	-9.70803700	-2.83389200	-7.56877200
C	-9.47096200	-6.25417100	-9.37077300
H	-11.58301500	-6.41627100	-9.01506300
C	-8.32962900	-5.46865000	-9.21088800
H	-7.53278100	-3.61006800	-8.46635000
H	-9.40450300	-7.20318300	-9.89091100
C	-12.69715200	-6.13470900	-6.09482100
H	-11.87862100	-6.65436400	-6.59508000
H	-13.61119400	-6.30344600	-6.67072900
C	-12.84474600	-6.64510500	-4.67860900
C	-11.81473000	-7.37781200	-4.07719600
C	-13.97976300	-6.33396800	-3.91553500
C	-11.89885400	-7.77203300	-2.74335700
H	-10.92912700	-7.62023300	-4.65501600
C	-14.06720800	-6.71445300	-2.58000300
H	-14.80011100	-5.78891100	-4.37029000

C	-13.02126300	-7.42897800	-1.98997600
H	-11.08748000	-8.32417800	-2.28302900
H	-14.93950100	-6.44798200	-1.99259000
C	-9.80499600	-3.80522500	-2.26233000
C	-10.88047900	-4.51260700	-1.71188100
C	-8.79963300	-3.32110500	-1.42055300
C	-10.95188300	-4.72226700	-0.33467500
H	-11.67435400	-4.87317000	-2.35491500
C	-8.86496000	-3.53809600	-0.04300700
H	-7.97125400	-2.75635200	-1.84202100
C	-9.94465200	-4.23540700	0.50184500
H	-11.79854400	-5.25530000	0.08339300
H	-8.08448600	-3.14768400	0.60350300
H	-10.00714200	-4.39021200	1.57482900
C	-15.34759800	-1.03098500	-4.65639200
C	-14.46807100	-0.15381200	-5.30003800
C	-16.43076400	-0.51264100	-3.94157600
C	-14.66976800	1.22389900	-5.22325700
H	-13.60554300	-0.55059100	-5.82592100
C	-16.63747700	0.86509400	-3.86953600
H	-17.10523700	-1.18974000	-3.42255800
C	-15.75529400	1.73660200	-4.51012100
H	-13.97162400	1.89747100	-5.71204000
H	-17.47760700	1.25632900	-3.30326300
H	-15.90755100	2.81010100	-4.44605700
C	-9.70514300	-3.58893900	-3.75593400
H	-9.12609600	-2.68103000	-3.94684600
C	-15.15445500	-2.52408100	-4.74593300

H	-15.57519800	-2.99180900	-3.84944300
C	-6.99202700	-5.96164100	-9.68514300
C	-13.13583300	-7.84632600	-0.55189100
F	-7.10846200	-6.90535900	-10.64345300
F	-6.23876400	-4.95848900	-10.18806600
F	-6.28099800	-6.51166100	-8.67197700
F	-13.94820000	-8.91627200	-0.40051100
F	-13.65967200	-6.85282300	0.20958400
F	-11.93993500	-8.17205100	-0.01888900
Ni	-12.40388600	-2.19820600	-3.50457400
C	-9.11644700	-4.80518500	-4.52693500
H	-8.90040100	-5.65505500	-3.87683000
H	-8.23887700	-4.56050400	-5.12962900
C	-15.73606700	-3.16349200	-6.02785700
H	-15.80718900	-2.44187100	-6.84785400
H	-16.69385800	-3.66538700	-5.88780100
H	-11.51277500	-0.91726700	-3.16106100
C	-13.50042100	-1.87800400	-1.80936900
H	-14.53513200	-1.57774700	-1.94728600
H	-13.33846900	-2.68371400	-1.09532400
C	-12.48585700	-0.86693100	-1.92939400
H	-12.83676000	0.12871400	-2.19782900
C	-11.37326400	-0.82846100	-0.87689000
H	-10.98323300	-1.83587900	-0.71443500
H	-10.53145400	-0.20804800	-1.20930500
C	-11.86734400	-0.31243800	0.40527800
N	-12.25618300	0.11046500	1.41444700

TS5^{doublet}

O	0.25198600	-2.63022600	1.49891500
C	0.43098800	-1.31676900	1.24182100
N	0.90029000	-1.02035100	0.09024200
O	-0.35013600	1.88798800	2.98822600
C	0.08280000	1.06749600	2.00966000
N	0.46341700	1.62091800	0.92239500
C	0.07306500	-0.39674500	2.38532500
C	-1.33538600	-0.76216800	2.93770900
H	-1.44107400	-0.26275800	3.90569100
H	-1.33134000	-1.83924400	3.13521600
C	-2.52280100	-0.40197100	2.06459500
C	-3.79672700	-0.39930400	2.65638000
C	-2.42178700	-0.09465900	0.70661500
C	-4.93079400	-0.10094200	1.91264300
H	-3.89719300	-0.64028600	3.71184600
C	-3.55268800	0.23126500	-0.04043400
H	-1.47624200	-0.09504100	0.18501600
C	-4.80630800	0.22394900	0.55681500
H	-5.91061000	-0.11665100	2.37836800
H	-3.43685000	0.47108200	-1.08915600
C	1.13463700	-0.62279400	3.52822000
H	0.89798600	-1.57684300	4.00646100
H	0.98360500	0.16795600	4.26900700
C	2.55505800	-0.64403500	3.01693300
C	3.25425500	-1.85509700	2.94832400
C	3.17343500	0.51578400	2.52979700
C	4.53432300	-1.91187200	2.40109200
H	2.78757100	-2.76283900	3.32064500

C	4.43656500	0.46277000	1.94747300
H	2.66182500	1.47016900	2.57965500
C	5.11733700	-0.75452600	1.88200300
H	5.06527400	-2.85589100	2.34710700
H	4.87504100	1.35834800	1.52237700
C	2.19148900	-2.30626100	-1.59730900
C	3.46879200	-2.15297200	-1.04681200
C	2.04417100	-2.43068800	-2.98182800
C	4.59249900	-2.12906900	-1.87055500
H	3.58140000	-2.03088900	0.02445600
C	3.16963500	-2.41312800	-3.80633900
H	1.04821300	-2.51107300	-3.40849300
C	4.44198200	-2.26102800	-3.25293400
H	5.57678300	-1.99853300	-1.43429700
H	3.04922900	-2.50134200	-4.88197100
H	5.31560100	-2.23495500	-3.89783300
C	-0.86550200	3.47869000	0.02659600
C	-0.49867200	4.01375000	-1.21208700
C	-2.21629300	3.23736600	0.30057200
C	-1.47552800	4.31212100	-2.16195100
H	0.55268200	4.16154300	-1.44089900
C	-3.19330800	3.53316600	-0.64907500
H	-2.51346600	2.78975500	1.24443600
C	-2.82204700	4.07269300	-1.88233100
H	-1.18159100	4.71903000	-3.12475500
H	-4.23550000	3.31964600	-0.43360000
H	-3.58093600	4.29826000	-2.62589700
C	0.96921500	-2.26914800	-0.72017100

H	0.07346400	-2.27104300	-1.34976100
C	0.21218900	3.08641300	1.01414700
H	1.14669800	3.58951400	0.75922000
C	-6.01926500	0.62940000	-0.22528100
C	6.48992200	-0.81012700	1.27177800
F	-6.33546100	1.93586000	0.00255200
F	-5.84553300	0.50283500	-1.55543600
F	-7.10956500	-0.09220100	0.11783800
F	6.62309200	0.06879600	0.25996600
F	6.76306200	-2.04353800	0.77667600
F	7.45427400	-0.52972600	2.17643700
Ni	1.42182300	0.77908000	-0.62473000
C	0.85998800	-3.34565800	0.37283300
H	1.83449400	-3.71346000	0.70664700
H	0.20380800	-4.17617700	0.11601000
C	-0.14542900	3.25287200	2.50913500
H	-1.06556200	3.80829800	2.68529200
H	0.66935100	3.68296800	3.09711800
Br	-0.26071100	0.54690500	-2.50278400
Cl	2.88437700	2.28742200	-1.26000600
C	-2.39156700	-1.81590500	-2.27997400
C	-1.53925900	-1.19086800	-2.91875600
C	-3.42671600	-2.48994900	-1.57469500
C	-4.76639200	-2.39275900	-2.00187000
C	-3.15349700	-3.15762200	-0.36268800
C	-5.79561000	-2.92124900	-1.22873700
H	-4.98350000	-1.86773600	-2.92551200
C	-4.18719900	-3.68205200	0.40541000

H	-2.12936100	-3.21131500	-0.00759800
C	-5.51269800	-3.56339200	-0.02087000
H	-6.82425800	-2.81564700	-1.56066200
H	-3.96084900	-4.17437700	1.34735400
H	-6.31927600	-3.96371300	0.58644400

IM5^{triplet}

O	-0.93249700	3.19683100	0.10722900
C	-0.50081900	1.94620700	0.36889700
N	-0.75184700	1.05873800	-0.51383700
O	1.29219700	0.24709700	3.16061400
C	0.71838600	0.40978900	1.95094900
N	0.72392400	-0.60303400	1.17458100
C	0.18720200	1.79967100	1.70311400
C	1.40381000	2.78859500	1.79630500
H	1.60701000	2.94772400	2.85842200
H	1.07451100	3.74479400	1.37865500
C	2.65974400	2.29390700	1.11548600
C	2.68930000	2.00558700	-0.25340600
C	3.81825200	2.07419200	1.87387200
C	3.84317500	1.50996500	-0.85551900
H	1.80402700	2.12963600	-0.86630300
C	4.98266800	1.59972400	1.27725200
H	3.80413000	2.27635300	2.94123800
C	4.99274100	1.31507300	-0.09135500
H	3.82855800	1.24719500	-1.90596400
H	5.87429700	1.43244200	1.87270800
C	-0.85284000	2.18871900	2.81338400
H	-0.88549000	3.28051600	2.85388000

H	-0.44967300	1.83856200	3.76860400
C	-2.24922900	1.65751700	2.57796300
C	-2.50517000	0.28909300	2.43726100
C	-3.31814600	2.55711700	2.45693500
C	-3.79398300	-0.17537700	2.18359600
H	-1.70182200	-0.43650600	2.48317100
C	-4.61238100	2.10137400	2.22652200
H	-3.13065900	3.62375800	2.54378200
C	-4.84934200	0.73021200	2.09026200
H	-3.95960300	-1.23378300	2.02605500
H	-5.43331600	2.80531900	2.13513400
C	-2.63060800	0.88156500	-2.09907700
C	-3.77989700	0.80277600	-1.30572300
C	-2.54149200	0.11487200	-3.26474300
C	-4.83595500	-0.02398700	-1.68117400
H	-3.84575100	1.36460600	-0.37964900
C	-3.59723200	-0.71701600	-3.63833700
H	-1.63373200	0.14858100	-3.86024700
C	-4.74444400	-0.78539800	-2.84823400
H	-5.72161300	-0.08220800	-1.05927600
H	-3.51696300	-1.31618100	-4.54040100
H	-5.56461300	-1.43712200	-3.13520200
C	2.34434500	-2.44642300	0.98351000
C	3.57822100	-1.86235100	0.68090500
C	1.97705600	-3.64500000	0.36454200
C	4.44499900	-2.47940100	-0.21811200
H	3.85728800	-0.91070600	1.12119700
C	2.84141800	-4.25938300	-0.54143700

H	1.00150100	-4.07645100	0.57050600
C	4.07627300	-3.67898400	-0.83045000
H	5.39848100	-2.01842600	-0.44871900
H	2.54468800	-5.18455700	-1.02624300
H	4.74829500	-4.15411500	-1.53916600
C	-1.43989100	1.70095700	-1.66453200
H	-0.70790500	1.72832900	-2.47762300
C	1.34791600	-1.75219000	1.87993200
H	0.53210300	-2.44450200	2.10959600
C	6.25834100	0.80970800	-0.72243500
C	-6.25267900	0.26096500	1.83475300
F	6.86831200	-0.11057100	0.07049700
F	6.04027500	0.22607300	-1.91645200
F	7.15535600	1.80305100	-0.91539700
F	-6.84000100	0.99229300	0.85064500
F	-7.03528200	0.39673600	2.92892000
F	-6.30457200	-1.03087500	1.45710500
Ni	-0.18767400	-0.86254300	-0.59410700
C	-1.72709100	3.11650300	-1.11654900
H	-2.77189800	3.26355900	-0.83594800
H	-1.39883800	3.91934700	-1.77646400
C	1.89528700	-1.08396700	3.16154400
H	2.97867800	-0.95090200	3.14927500
H	1.59016800	-1.58286300	4.08133800
Br	1.29061500	-0.66269800	-2.49164100
Cl	-1.71660300	-2.34080400	0.11238300

IM6^{CSS}

O	2.27249800	-1.32447100	-1.52684500

C	1.80795600	-0.08490400	-1.78766700
N	2.67424400	0.86439800	-1.77960500
O	-1.55564700	1.48202900	-2.03411200
C	-0.20932800	1.38109500	-1.98337500
N	0.45547600	2.47568400	-2.05233200
C	0.32758200	-0.02483900	-2.09999600
C	0.11566500	-0.37495300	-3.64545100
H	0.08050800	0.57182400	-4.18998300
H	-0.86656100	-0.84043100	-3.74349800
C	1.21008300	-1.22190700	-4.23743900
C	2.32465900	-0.58775900	-4.80543900
C	1.19237300	-2.61843200	-4.16439900
C	3.41720600	-1.32893300	-5.24437900
H	2.34084900	0.49561600	-4.88262600
C	2.28403900	-3.36798400	-4.59513000
H	0.33095900	-3.12686800	-3.74191400
C	3.40415100	-2.72054300	-5.11972400
H	4.27995200	-0.83097200	-5.67418800
H	2.27316000	-4.44939800	-4.51706800
C	-0.37844400	-1.05234600	-1.14419700
H	-0.15360800	-0.74062600	-0.11793800
H	0.13453500	-2.00375200	-1.29545000
C	-1.86721600	-1.26981400	-1.28140300
C	-2.77012500	-0.59369200	-0.44988900
C	-2.37727700	-2.19953500	-2.19508900
C	-4.13886900	-0.81083000	-0.55004100
H	-2.39276100	0.12005400	0.27613800
C	-3.74902100	-2.42025500	-2.31033600

H	-1.69758100	-2.77333200	-2.81814500
C	-4.63230700	-1.71995100	-1.49061300
H	-4.82708100	-0.27313900	0.09349300
H	-4.13019800	-3.13563400	-3.03050300
C	4.36135500	0.73107500	0.02085100
C	3.60318700	0.31519300	1.12220400
C	5.44898500	1.58236700	0.22290700
C	3.93012700	0.74459500	2.40658700
H	2.75079400	-0.34460500	0.97698900
C	5.77972500	2.01217500	1.50828200
H	6.02304000	1.92810700	-0.63128500
C	5.02078700	1.59526500	2.60180000
H	3.33426200	0.41803600	3.25415000
H	6.62277700	2.68124900	1.65183300
H	5.27402300	1.93427500	3.60204400
C	-0.44206300	3.81406900	-3.89459700
C	-1.10967900	2.98742600	-4.81233400
C	0.35679000	4.85533100	-4.37907900
C	-0.97642900	3.19644900	-6.18496500
H	-1.74123600	2.17624300	-4.46188300
C	0.48720800	5.06469600	-5.75275100
H	0.89682200	5.47975700	-3.67616400
C	-0.17524800	4.23652900	-6.65944200
H	-1.50299800	2.54996600	-6.88161600
H	1.11226200	5.87735200	-6.11144700
H	-0.07192100	4.40133300	-7.72823700
C	3.99150700	0.27870300	-1.37413300
H	4.73960100	0.62120300	-2.08861700

C	-0.52946800	3.55478300	-2.39924000
H	-0.27214700	4.44970400	-1.83474500
C	4.64544100	-3.49771200	-5.44832500
C	-6.10930400	-1.98755500	-1.56174800
F	4.38774200	-4.79893900	-5.69259300
F	5.28911300	-2.99611000	-6.52277700
F	5.52204300	-3.45443500	-4.41119600
F	-6.49571800	-2.89391100	-0.63446800
F	-6.47662400	-2.47393900	-2.76715100
F	-6.83026400	-0.86673600	-1.33611200
Ni	2.43110400	2.73838500	-1.85341700
C	3.72453400	-1.23520800	-1.50465300
H	4.07837600	-1.81963500	-0.65620500
H	4.09221500	-1.66176500	-2.44070500
C	-1.84207000	2.91219400	-1.94534500
H	-2.69932400	3.11875500	-2.58383100
H	-2.08089400	3.13483800	-0.90096100
Br	2.40876600	5.05348000	-1.41002400
H	3.87230800	2.90534000	-1.81802000

IM7^{CSS}

O	2.16329400	-1.51205600	-0.97020600
C	1.84525900	-0.35620200	-1.57787800
N	2.80395200	0.46295400	-1.81658900
O	-1.00947600	1.61547500	-1.09284000
C	0.08161700	1.29461100	-1.81566900
N	0.76989100	2.26081800	-2.29575100
C	0.41355600	-0.16869300	-2.04674600
C	0.42184800	-0.42348000	-3.62092000

H	0.60853200	0.53765700	-4.10088200
H	-0.57930800	-0.74875700	-3.90987300
C	1.48945800	-1.40033600	-4.05321100
C	2.67901900	-0.90442200	-4.60727100
C	1.38913300	-2.77529900	-3.80534600
C	3.76154400	-1.74629600	-4.84550400
H	2.77470000	0.15926100	-4.80422800
C	2.46663800	-3.62522200	-4.03961400
H	0.47663300	-3.19009400	-3.39127800
C	3.66256800	-3.10508900	-4.53875200
H	4.68432700	-1.34477000	-5.25009500
H	2.38645100	-4.68489800	-3.82279600
C	-0.53256300	-1.14282000	-1.27769600
H	-0.52501400	-0.85609300	-0.22191200
H	-0.07007100	-2.13046500	-1.33078900
C	-1.96608000	-1.24381400	-1.76392900
C	-2.99463600	-0.50692700	-1.16091000
C	-2.31120900	-2.12959200	-2.79553100
C	-4.31261900	-0.62315300	-1.59391400
H	-2.75757300	0.16884900	-0.34920500
C	-3.62550600	-2.24929300	-3.24009800
H	-1.54686400	-2.74457900	-3.25890000
C	-4.63013000	-1.48847200	-2.64203900
H	-5.09483600	-0.03964900	-1.12052200
H	-3.86939400	-2.93142600	-4.04725200
C	4.52410700	0.71602400	-0.08337200
C	3.83137900	0.68071000	1.13312000
C	5.62020500	1.57018000	-0.22158400

C	4.23625800	1.48157100	2.19927900
H	2.96783300	0.02912300	1.24783700
C	6.02687000	2.37359100	0.84457900
H	6.14036700	1.62081300	-1.17323800
C	5.33741500	2.32958400	2.05655400
H	3.69420600	1.44480100	3.14005000
H	6.87802800	3.03714000	0.72438600
H	5.65342500	2.95500100	2.88647000
C	-0.71031800	3.91184000	-3.37121100
C	-1.69644000	3.08507500	-3.92708700
C	-0.38608900	5.11018000	-4.01007000
C	-2.34941000	3.45500200	-5.10143900
H	-1.96497200	2.14925600	-3.44262400
C	-1.04156400	5.48295800	-5.18396500
H	0.40362900	5.73243100	-3.60367100
C	-2.02299300	4.65729600	-5.73276300
H	-3.11206600	2.80575600	-5.52237700
H	-0.77460400	6.41406200	-5.67518700
H	-2.52910900	4.94575800	-6.64967000
C	4.05705600	-0.11528900	-1.25667600
H	4.80728200	-0.10016600	-2.04701500
C	-0.00877900	3.51183000	-2.08687700
H	0.67168700	4.30039400	-1.76798100
C	4.87739900	-3.98141200	-4.61866900
C	-6.05952500	-1.65805100	-3.07383400
F	4.56397200	-5.27285200	-4.85503900
F	5.73791000	-3.58362300	-5.57666300
F	5.55863200	-3.95347400	-3.44095000

F	-6.67382800	-2.64264100	-2.37779300
F	-6.15330800	-1.98418900	-4.38162000
F	-6.77985700	-0.53159200	-2.88058500
Ni	2.73914700	2.11807700	-2.75268000
C	3.62311500	-1.56062100	-0.92218400
H	3.91053800	-1.89352100	0.07448000
H	3.94594100	-2.28368900	-1.67494000
C	-0.98067900	3.07486900	-0.97072600
H	-2.00035400	3.43484800	-1.10029500
H	-0.61271200	3.30666300	0.03321000
H	4.16178400	1.98102000	-3.03068300
Cl	2.84671500	4.01787000	-3.78878400

TS6^{doublet}

O	-1.17612000	1.35467900	-2.85005000
C	-0.57991000	1.02714500	-1.67222600
N	-0.56854900	-0.20725800	-1.34946700
O	0.93587000	2.93293500	1.15164100
C	0.52765100	1.84811300	0.43467900
N	0.69250300	0.69881700	0.97557100
C	-0.03533100	2.21297900	-0.91749500
C	1.10480100	2.89300600	-1.75686400
H	1.28465100	3.88322100	-1.33065400
H	0.72199600	3.02824000	-2.77248900
C	2.38177900	2.08925800	-1.75493600
C	2.49548600	0.91742300	-2.51491400
C	3.45161900	2.46420700	-0.93437300
C	3.63690000	0.12780200	-2.43761600
H	1.67885300	0.61135400	-3.16045700

C	4.60506000	1.68526300	-0.85941600
H	3.37462800	3.37098200	-0.34130600
C	4.69278400	0.51091500	-1.60571200
H	3.70415100	-0.79071800	-3.01091800
H	5.42356500	1.97673500	-0.21158900
C	-1.20298300	3.24603400	-0.73392900
H	-1.45918700	3.62910000	-1.72448900
H	-0.80928300	4.07669700	-0.14269800
C	-2.42213900	2.64752900	-0.07418700
C	-2.45913100	2.44851500	1.31339400
C	-3.51815200	2.23746400	-0.84116200
C	-3.55965500	1.84767100	1.91585200
H	-1.62175700	2.77315400	1.92367100
C	-4.62631400	1.64099700	-0.24446000
H	-3.49403800	2.37218200	-1.91776300
C	-4.64571000	1.44329800	1.13488700
H	-3.57643900	1.68742700	2.98889700
H	-5.46051400	1.30643400	-0.84975000
C	-2.67859500	-1.45071100	-1.74423800
C	-3.59484500	-2.09706000	-2.58242000
C	-2.98298800	-1.30584400	-0.38981200
C	-4.80245200	-2.57562700	-2.07851100
H	-3.35632300	-2.24324700	-3.63374100
C	-4.18552200	-1.79816900	0.11901900
H	-2.27859700	-0.81502200	0.27255300
C	-5.10245800	-2.42519600	-0.72263000
H	-5.50320500	-3.07500400	-2.74161300
H	-4.40919100	-1.67367400	1.17291000

H	-6.04250900	-2.79667900	-0.32539500
C	2.46381900	0.01208900	2.60669200
C	2.77396100	-0.30895000	3.93092000
C	3.28187000	-0.45777300	1.57491100
C	3.89051800	-1.09173700	4.22484000
H	2.12873600	0.03840600	4.73478600
C	4.39314700	-1.24897000	1.86633200
H	3.02707700	-0.23446500	0.54625700
C	4.70066500	-1.56506700	3.19137100
H	4.11798900	-1.34272200	5.25688900
H	5.01178200	-1.62073100	1.05700400
H	5.56475500	-2.18354300	3.41680500
C	-1.37308400	-0.95019000	-2.34236100
H	-0.79398500	-1.81179000	-2.68567900
C	1.28159300	0.90422200	2.31903100
H	0.49036200	0.69796600	3.04869600
C	5.91395600	-0.35984400	-1.54014100
C	-5.83872300	0.82528900	1.80581500
F	6.60866800	-0.33906600	-2.69931000
F	6.75884100	0.01148700	-0.55585700
F	5.58088100	-1.65680600	-1.31554200
F	-5.46521900	-0.05621200	2.76711100
F	-6.63186300	0.16397100	0.93831300
F	-6.60591100	1.75710800	2.41823200
Ni	0.34233100	-1.10782800	0.22881900
C	-1.56149300	0.09540700	-3.47130000
H	-2.59185100	0.19224600	-3.81334600
H	-0.89551000	-0.06841700	-4.32384300

C	1.64953500	2.40868400	2.30268700
H	2.71889100	2.56136700	2.13296500
H	1.32519000	2.96253100	3.18427200
H	1.84685000	-2.82683900	2.66171400
C	0.76798400	-2.99392200	2.75442300
H	0.42040500	-2.35124300	3.57278500
H	0.62555800	-4.03802100	3.08301900
C	0.04768200	-2.68040300	1.45838200
H	-1.02929300	-2.85160200	1.48596200
C	0.69024700	-3.10396700	0.20362100
H	1.32605600	-1.99837100	-0.63350000
H	1.67111000	-3.57147200	0.31290200
C	-0.10431700	-3.77526100	-0.80304500
N	-0.75011100	-4.26483300	-1.63942500

9. Proposed mechanism

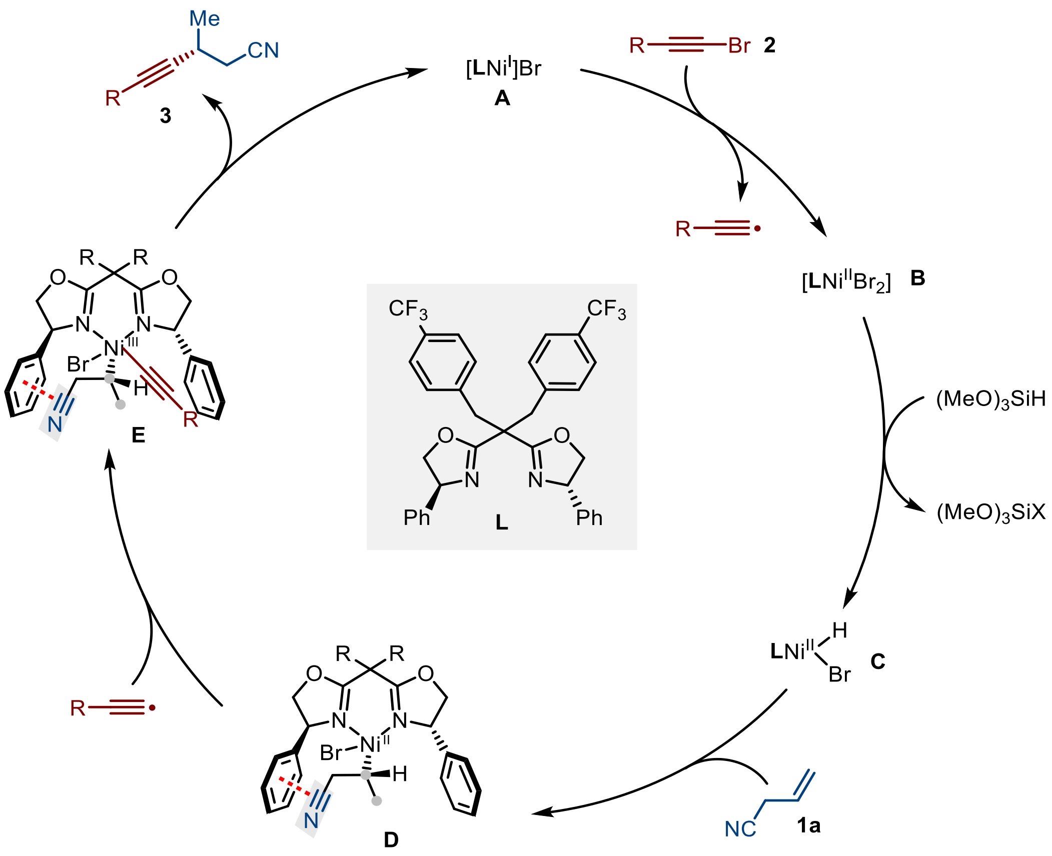

Fig. S13 Proposed mechanism

10. NMR spectra

^{1}H NMR (400 MHz, $CDCl_3$) spectrum of 3a

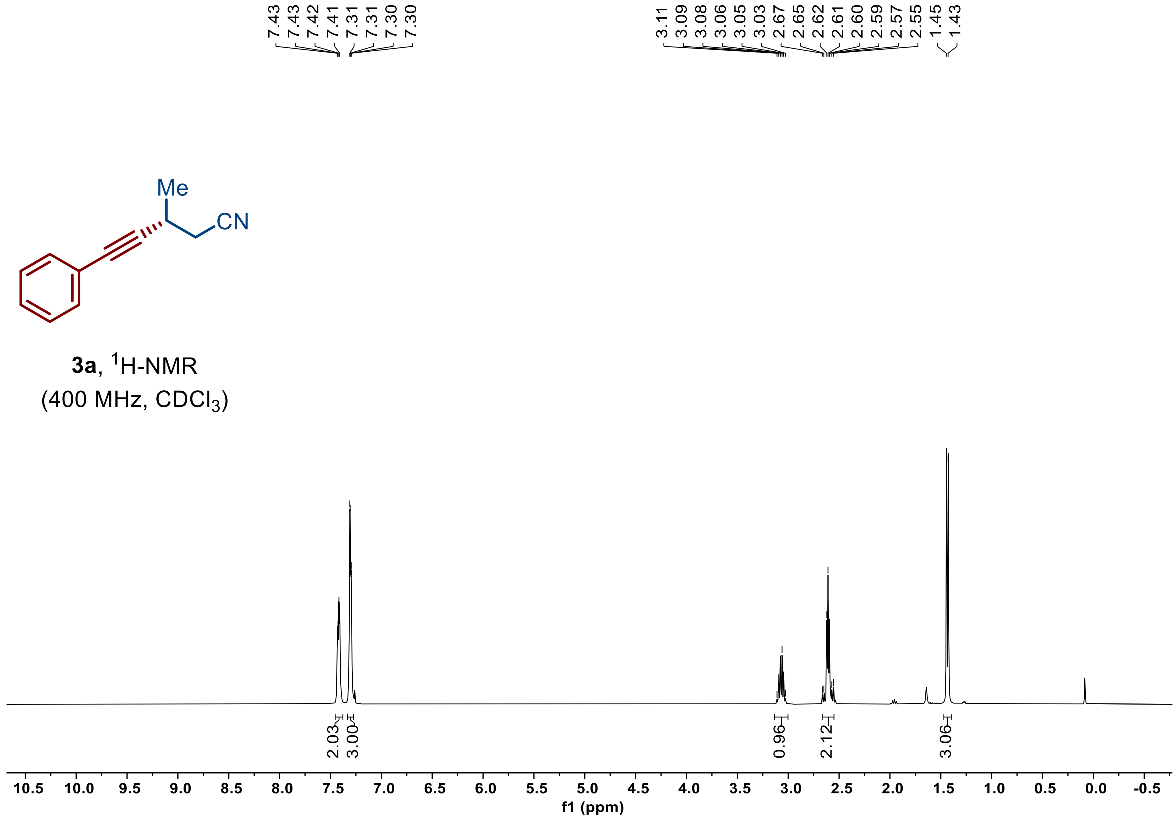

^{13}C NMR (100 MHz, $CDCl_3$) spectrum of 3a

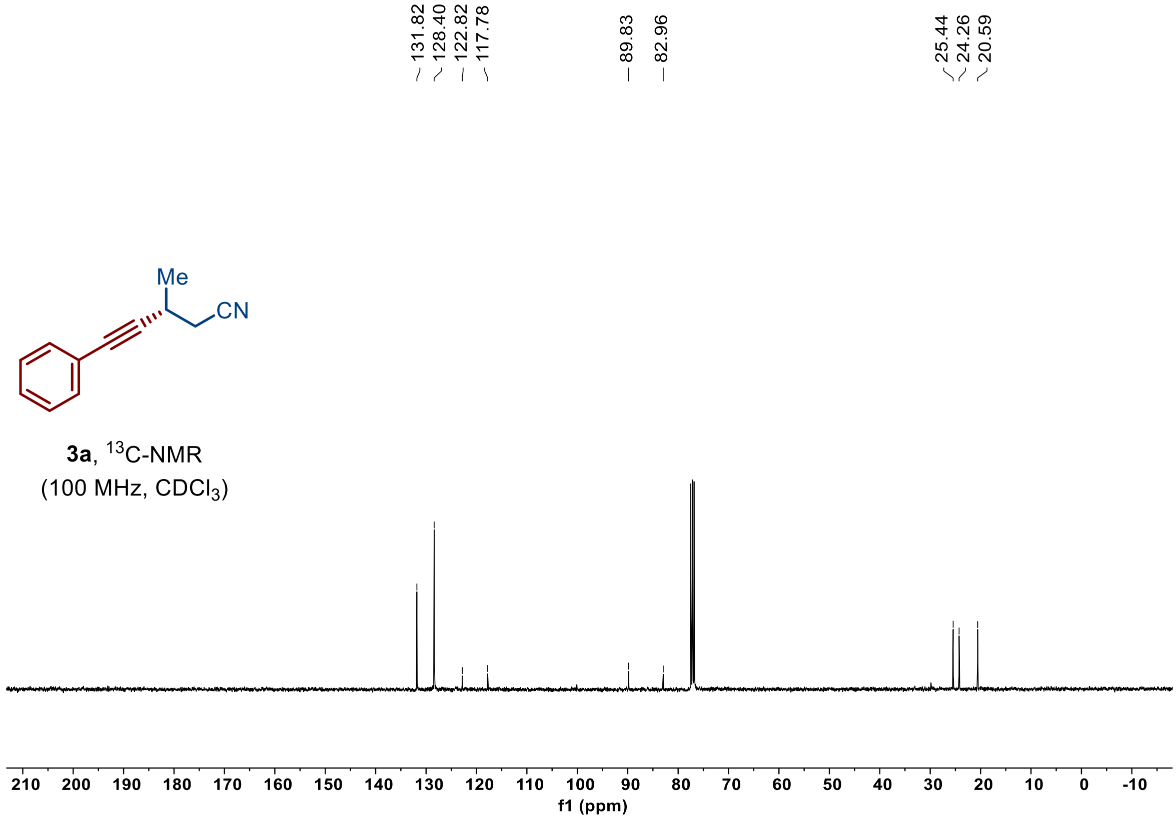

^{1}H NMR (400 MHz, $CDCl_3$) spectrum of 3b

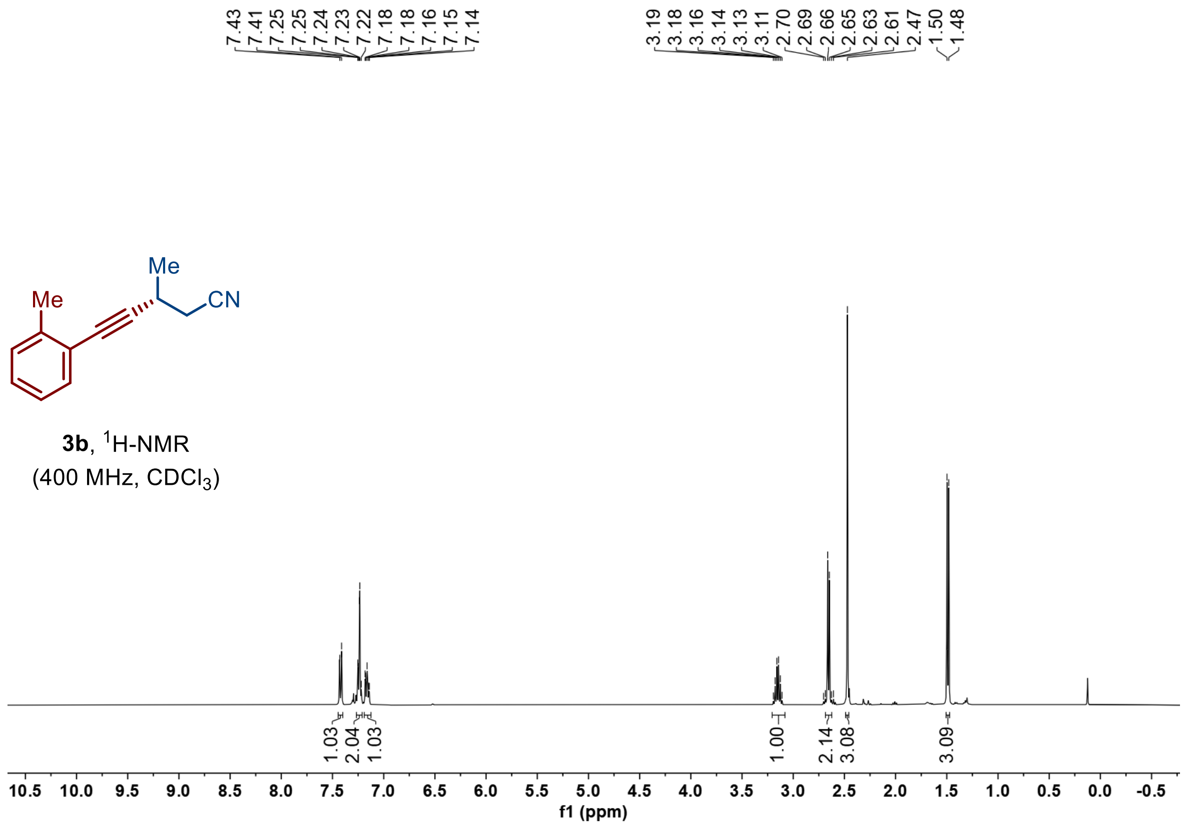

^{13}C NMR (100 MHz, $CDCl_3$) spectrum of 3b

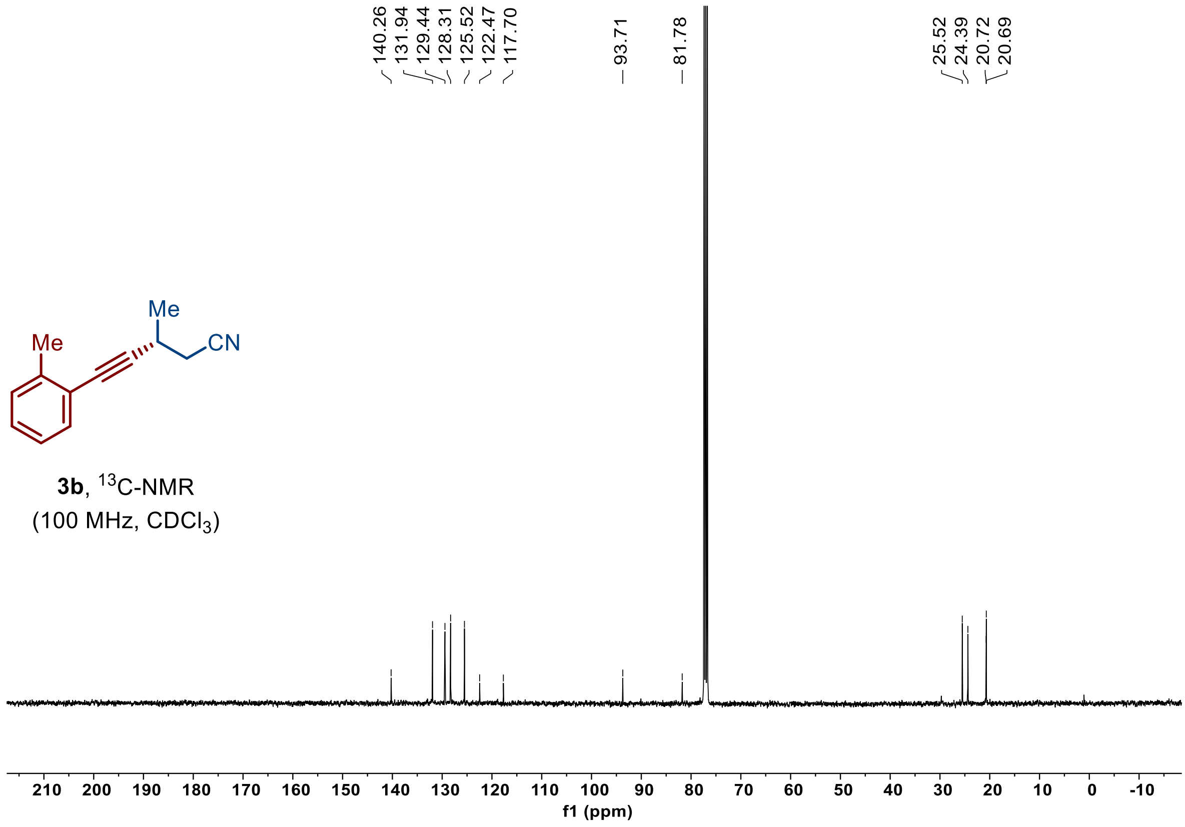

^{1}H NMR (400 MHz, CDCl$_3$) spectrum of 3c

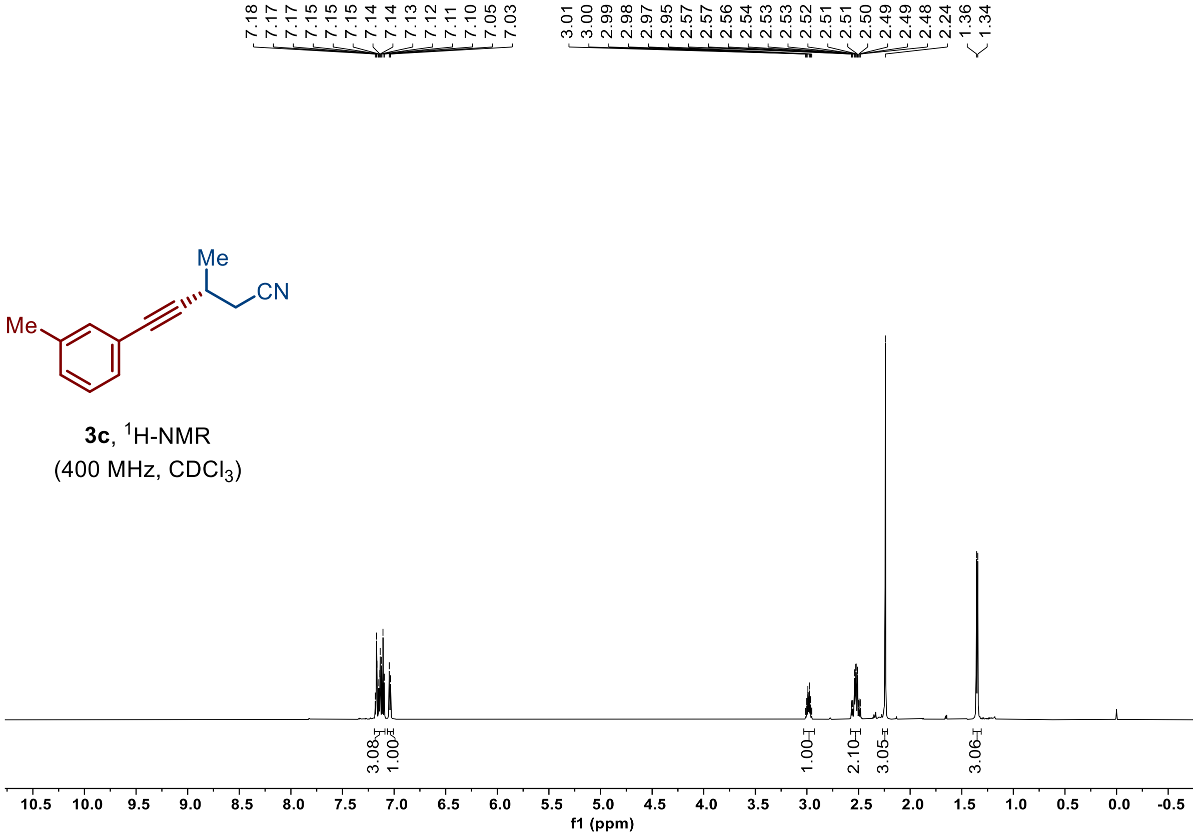

^{13}C NMR (100 MHz, CDCl$_3$) spectrum of 3c

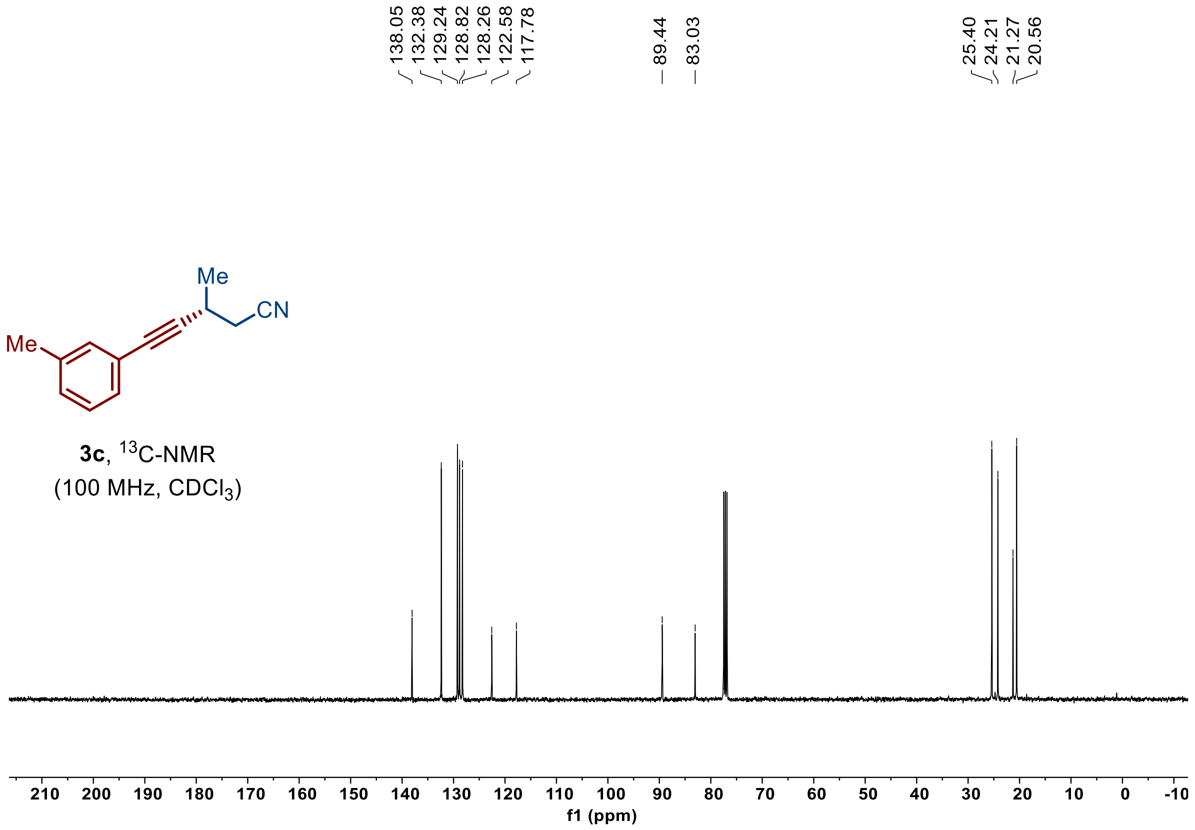

^{1}H NMR (400 MHz, $CDCl_3$) spectrum of 3d

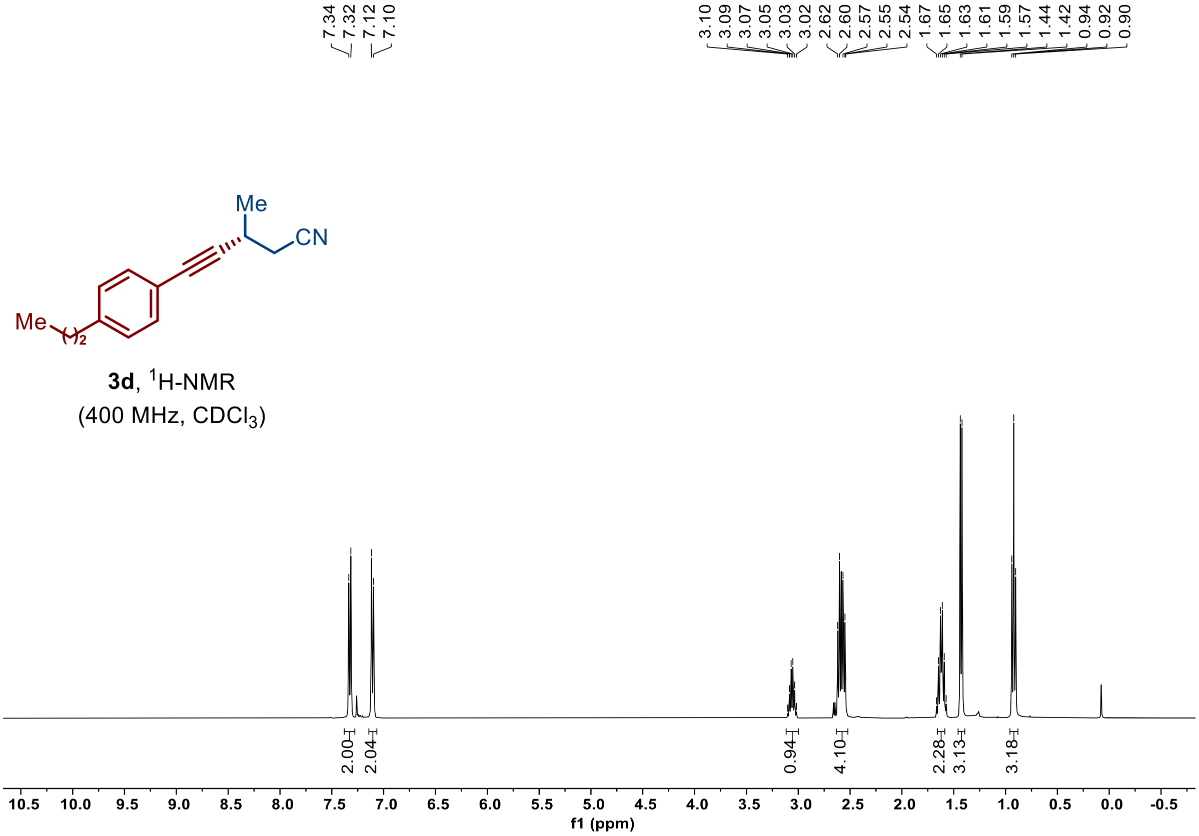

^{13}C NMR (100 MHz, $CDCl_3$) spectrum of 3d

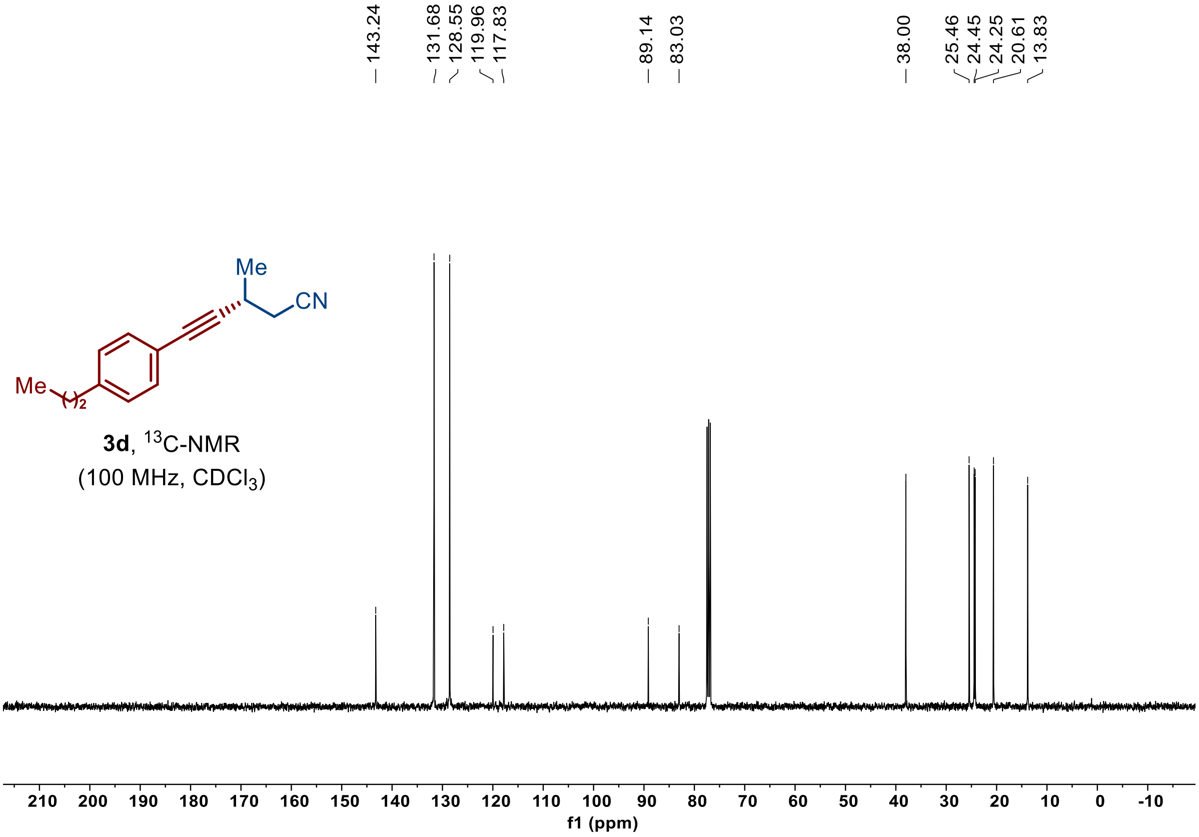

^{1}H NMR (400 MHz, $CDCl_3$) spectrum of 3e

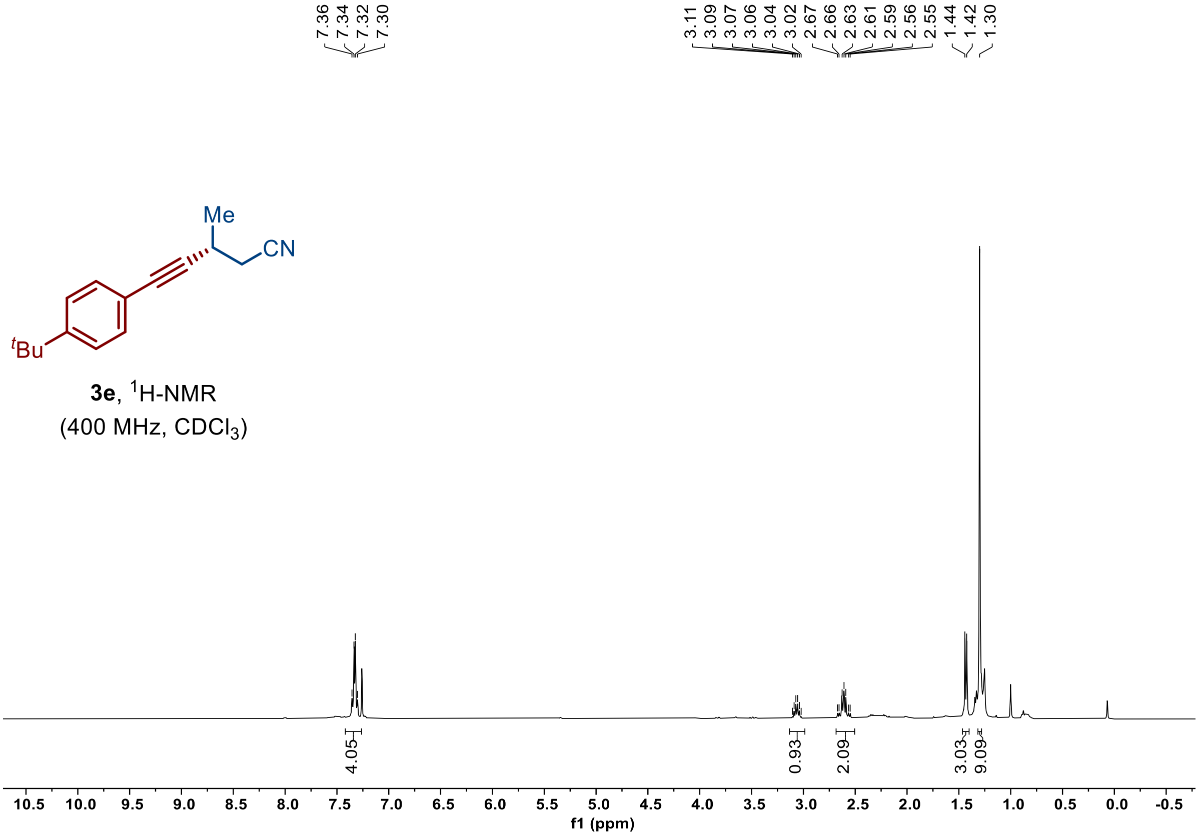

^{13}C NMR (100 MHz, $CDCl_3$) spectrum of 3e

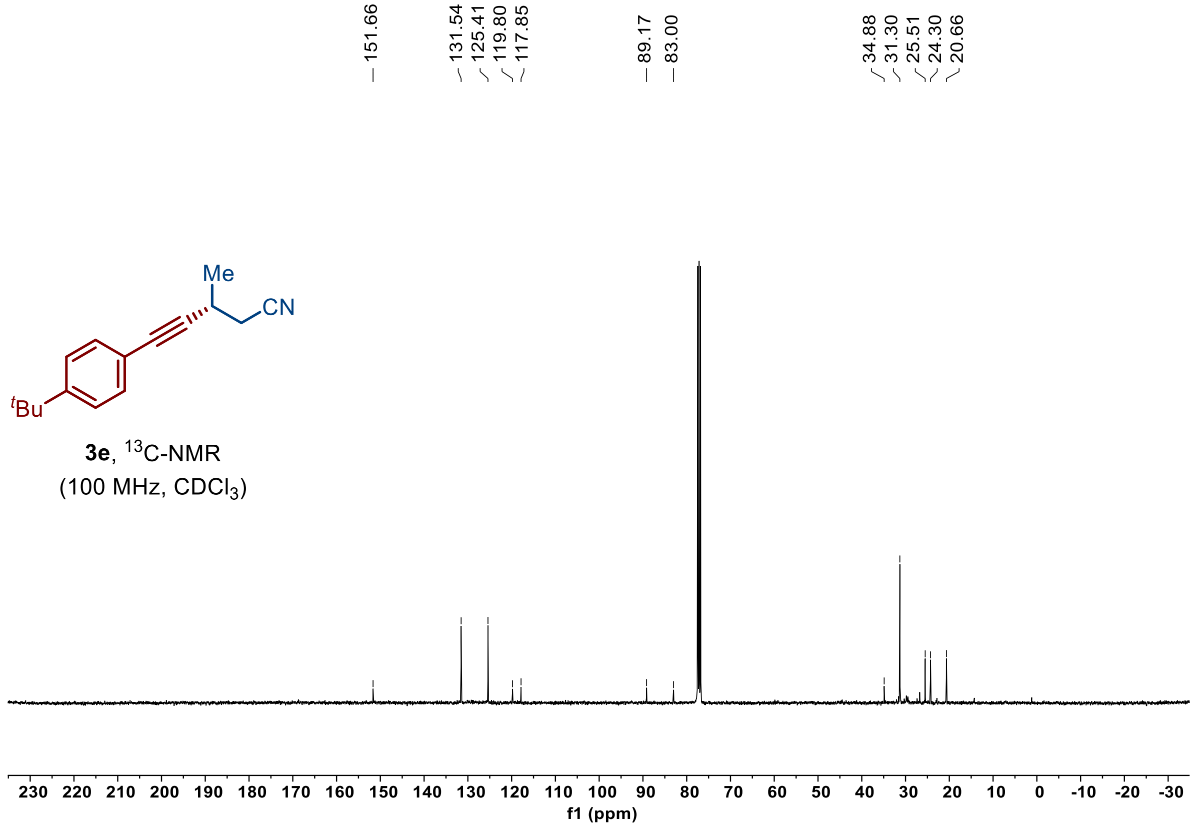

^{1}H NMR (400 MHz, CDCl$_3$) spectrum of 3e'

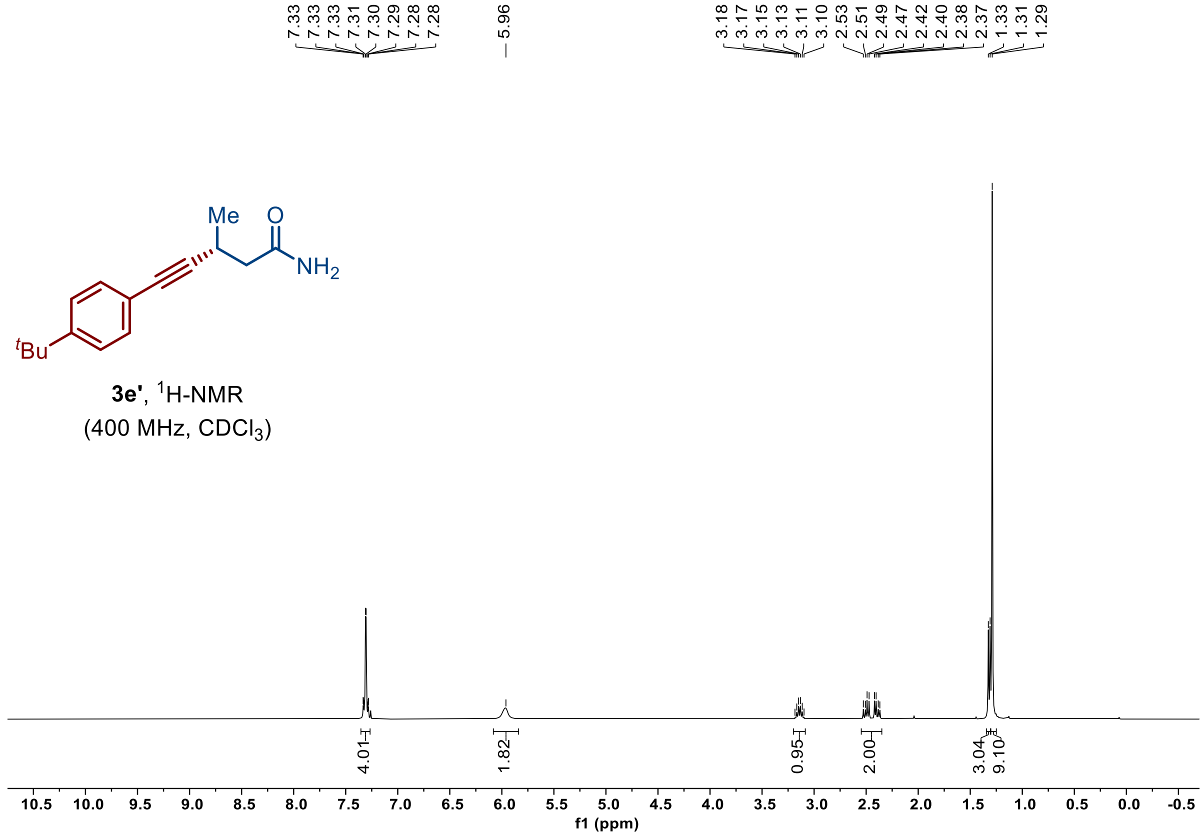

^{13}C NMR (100 MHz, CDCl$_3$) spectrum of 3e'

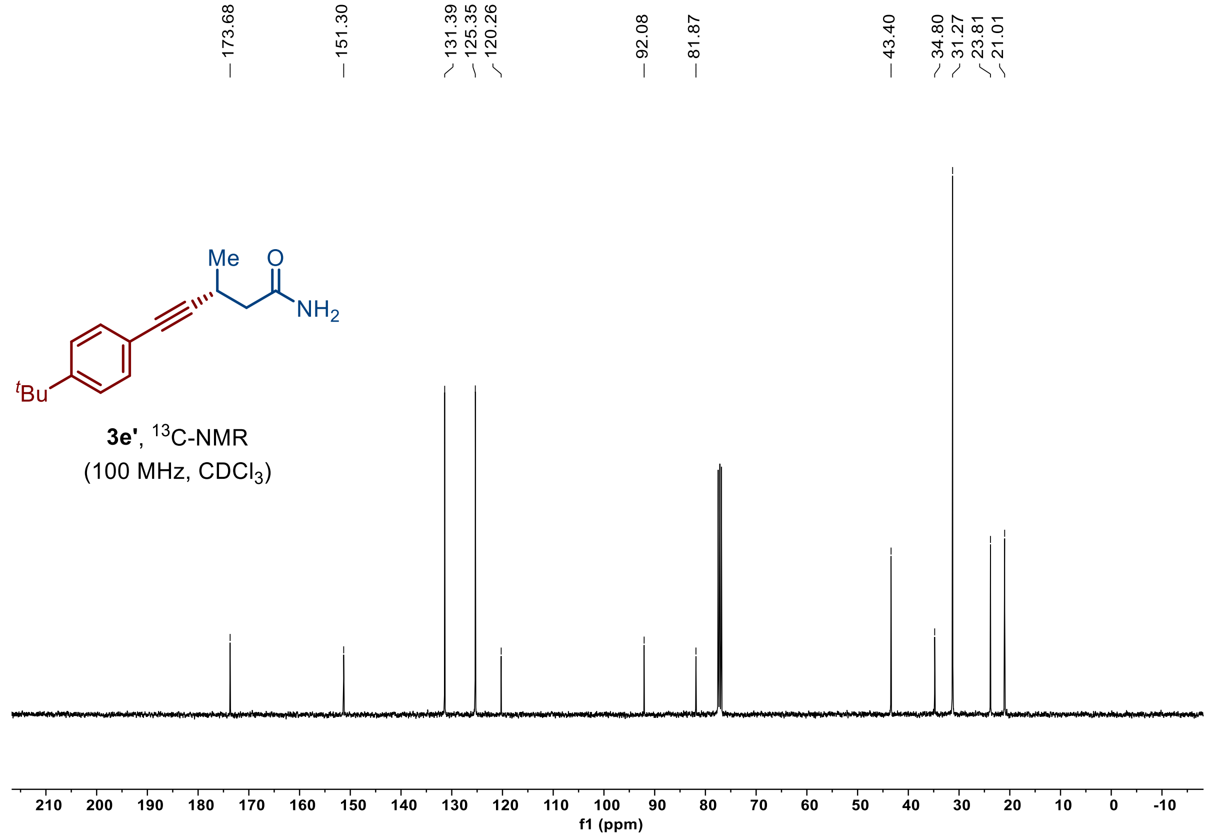

^{1}H NMR (400 MHz, CDCl₃) spectrum of 3f

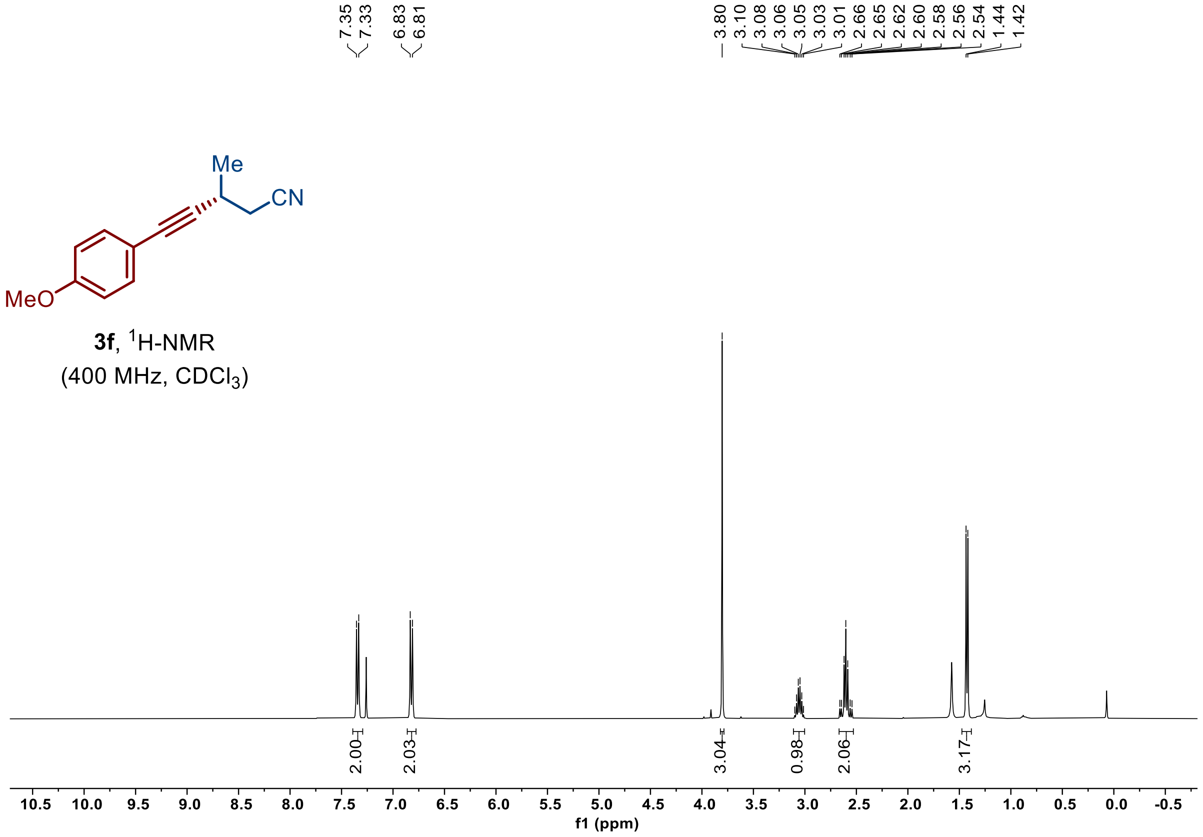

^{13}C NMR (100 MHz, CDCl₃) spectrum of 3f

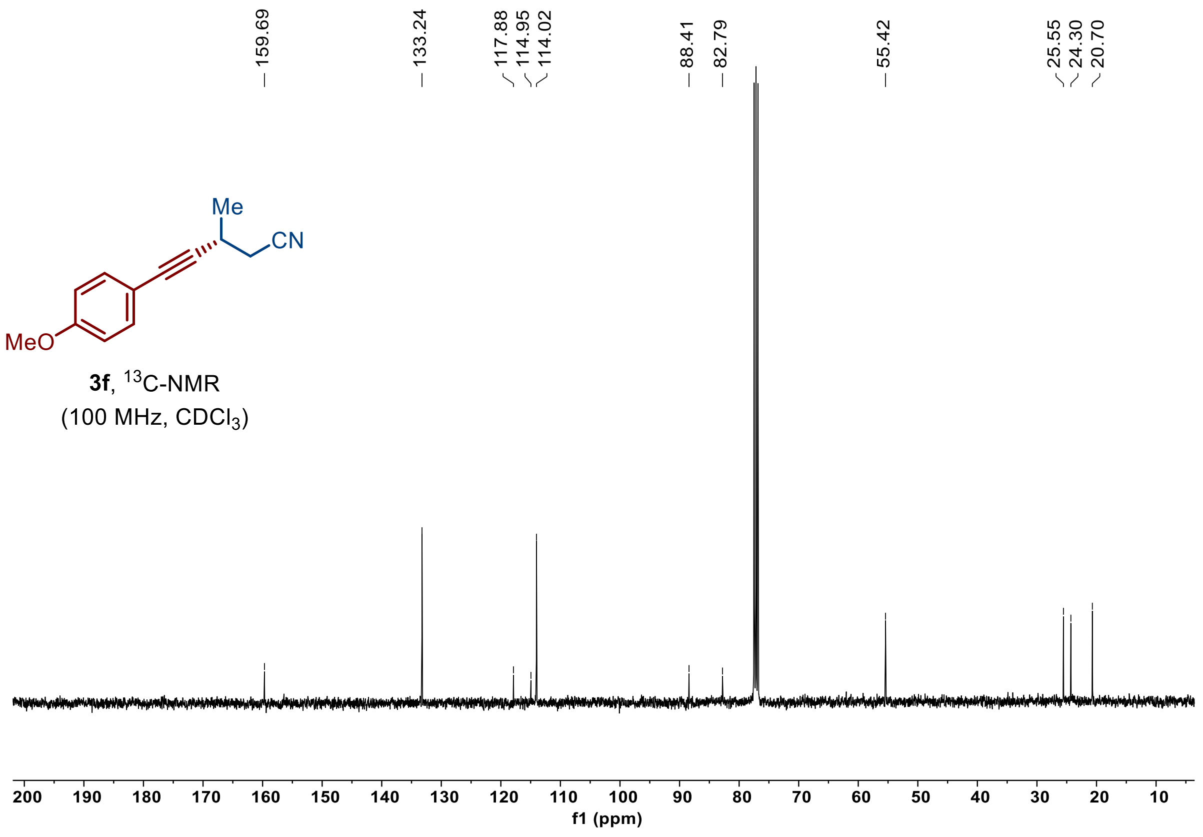

^{1}H NMR (400 MHz, CDCl$_3$) spectrum of 3g

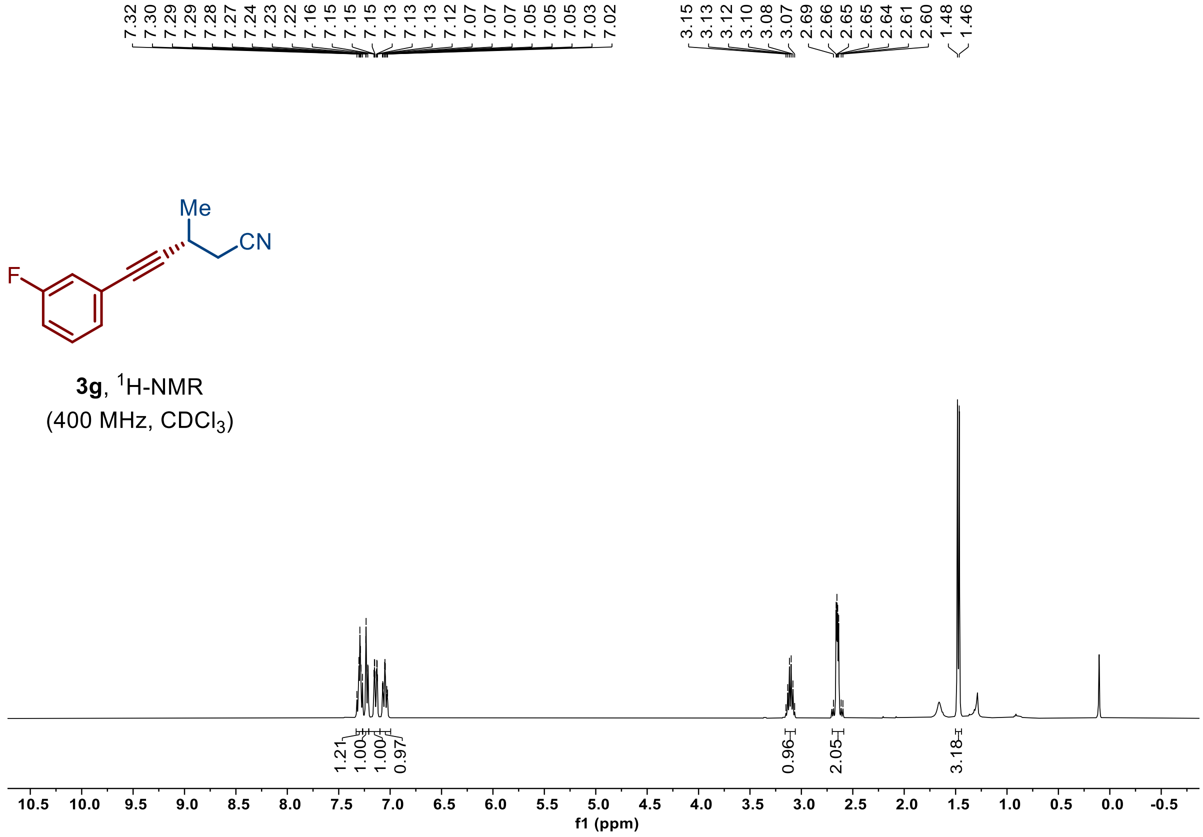

^{13}C NMR (100 MHz, CDCl$_3$) spectrum of 3g

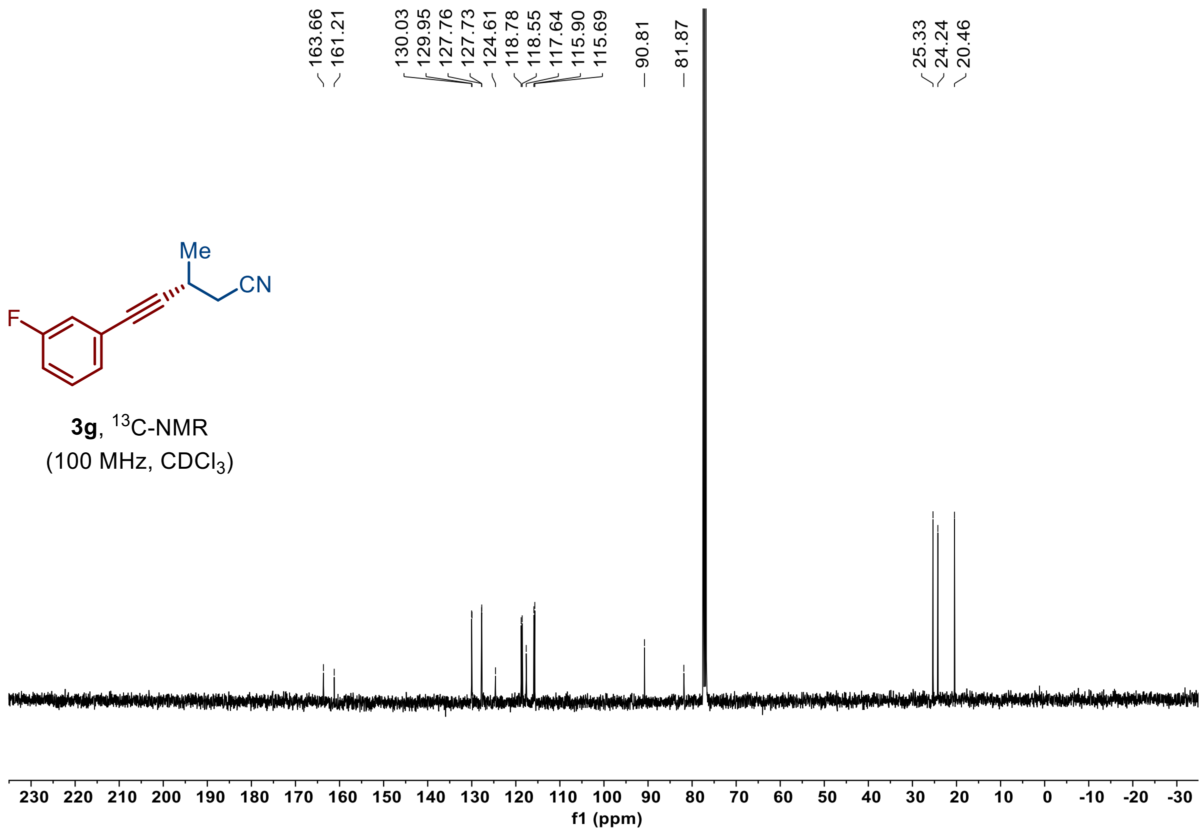

^{19}F NMR (376 MHz, CDCl3) spectrum of 3g

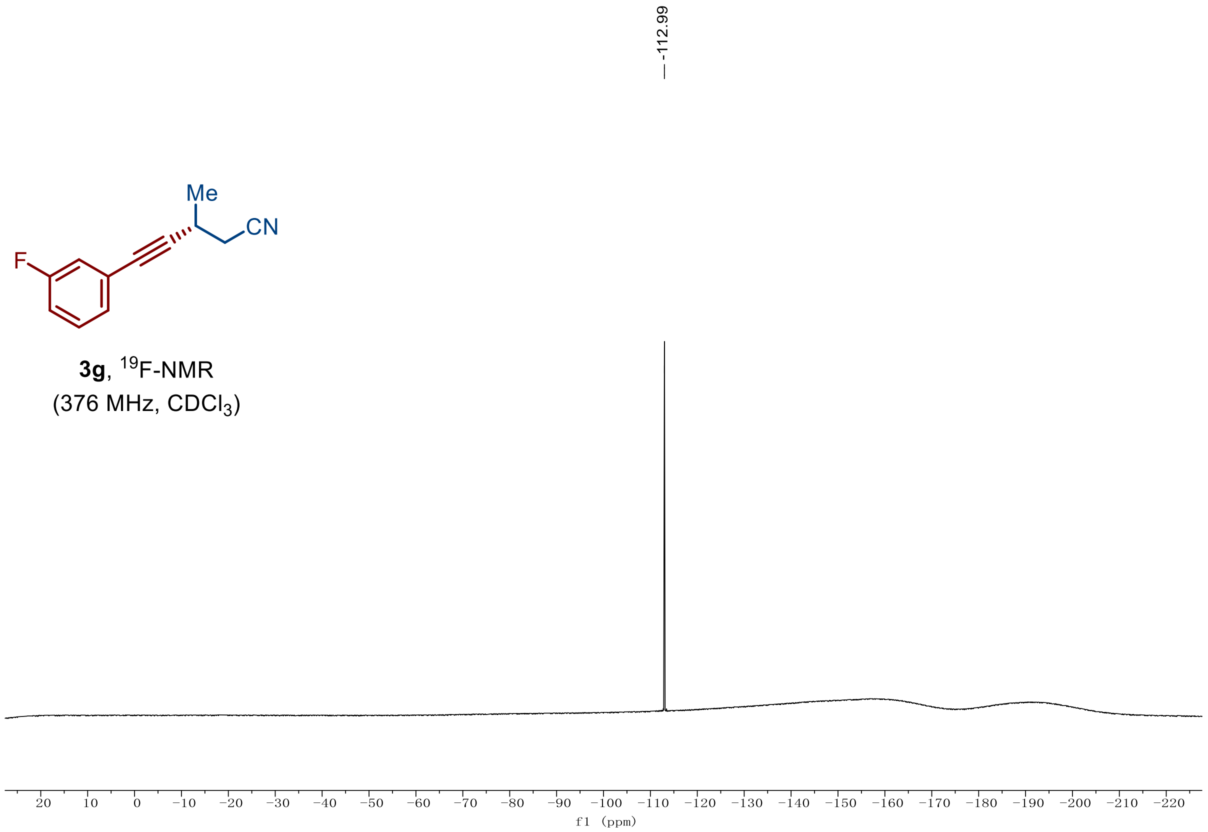

^{1}H NMR (400 MHz, $CDCl_3$) spectrum of 3h

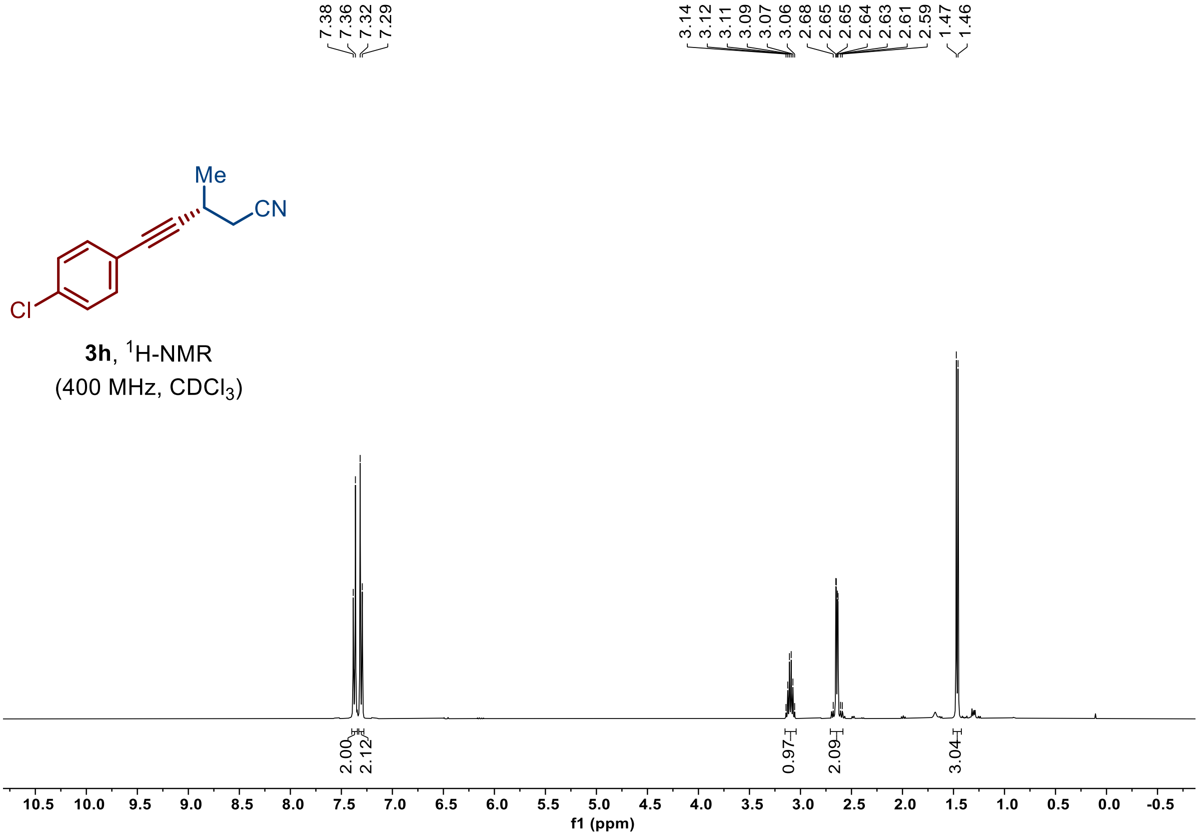

^{13}C NMR (100 MHz, $CDCl_3$) spectrum of 3h

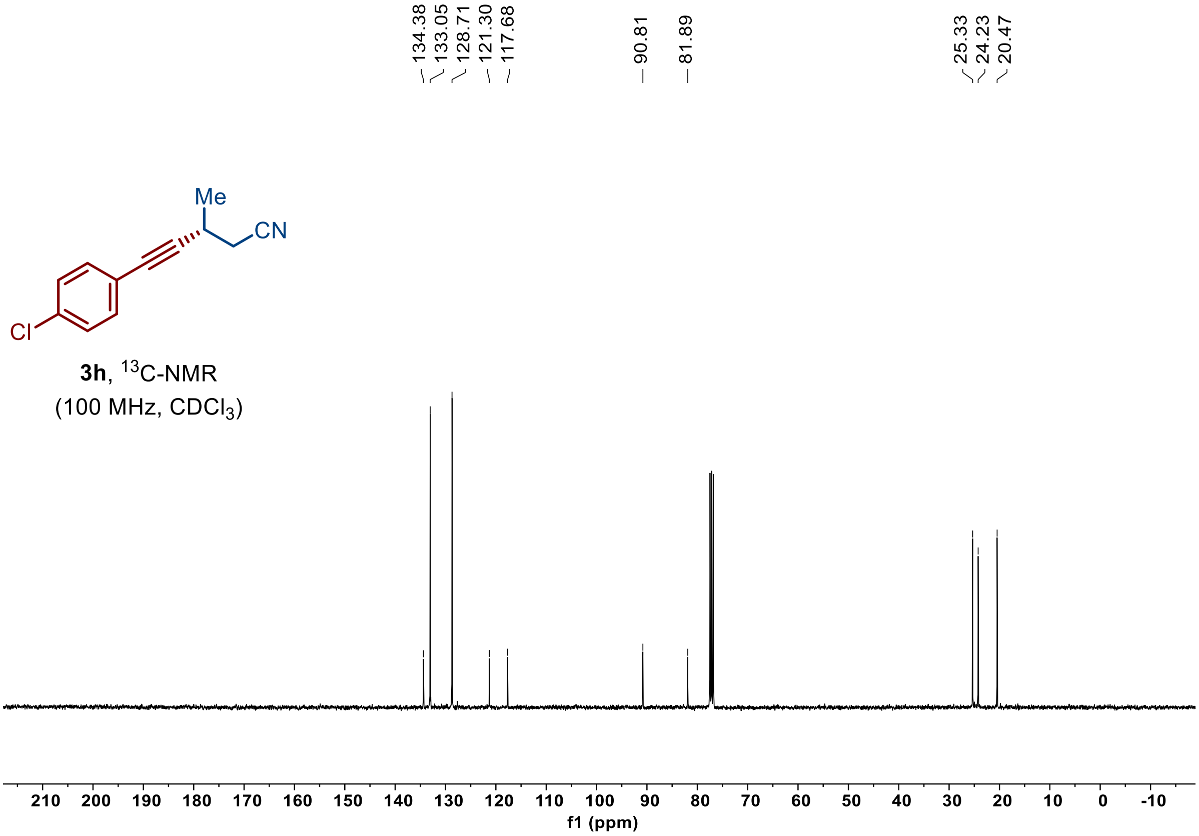

^{1}H NMR (400 MHz, $CDCl_3$) spectrum of 3i

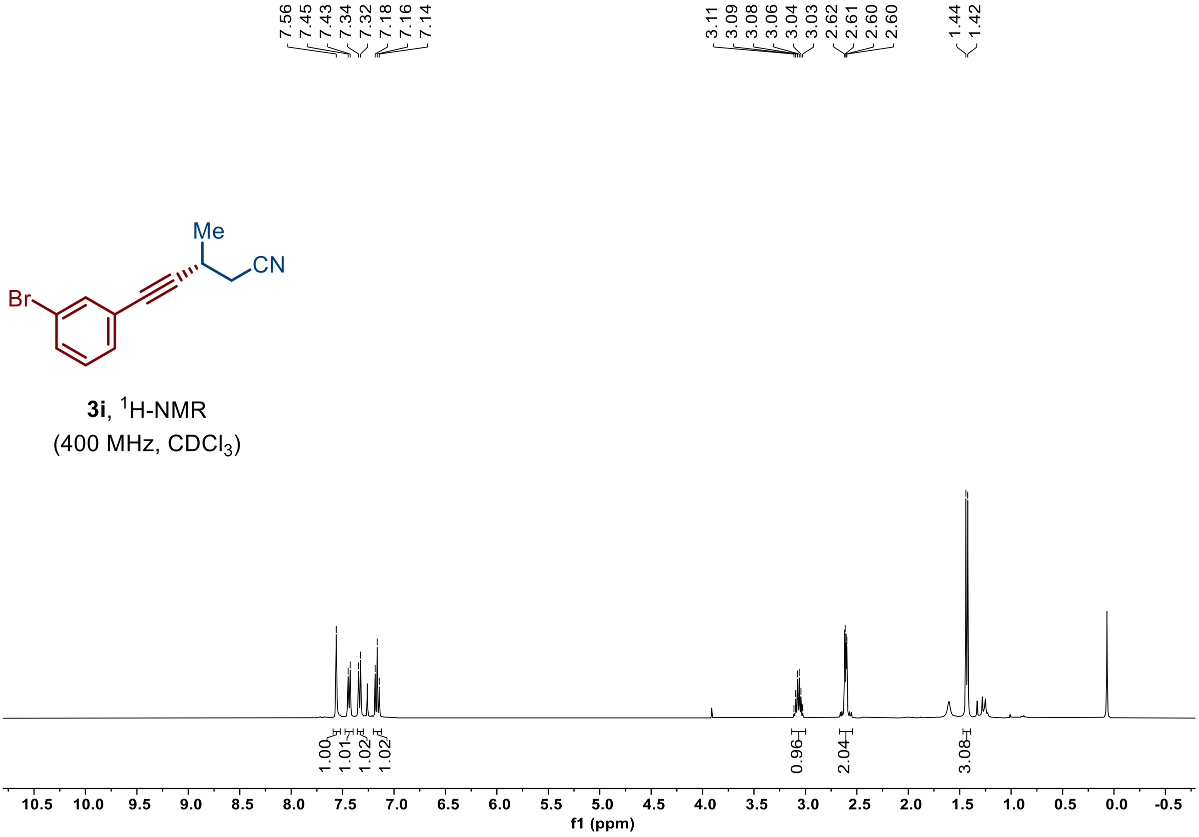

^{13}C NMR (100 MHz, $CDCl_3$) spectrum of 3i

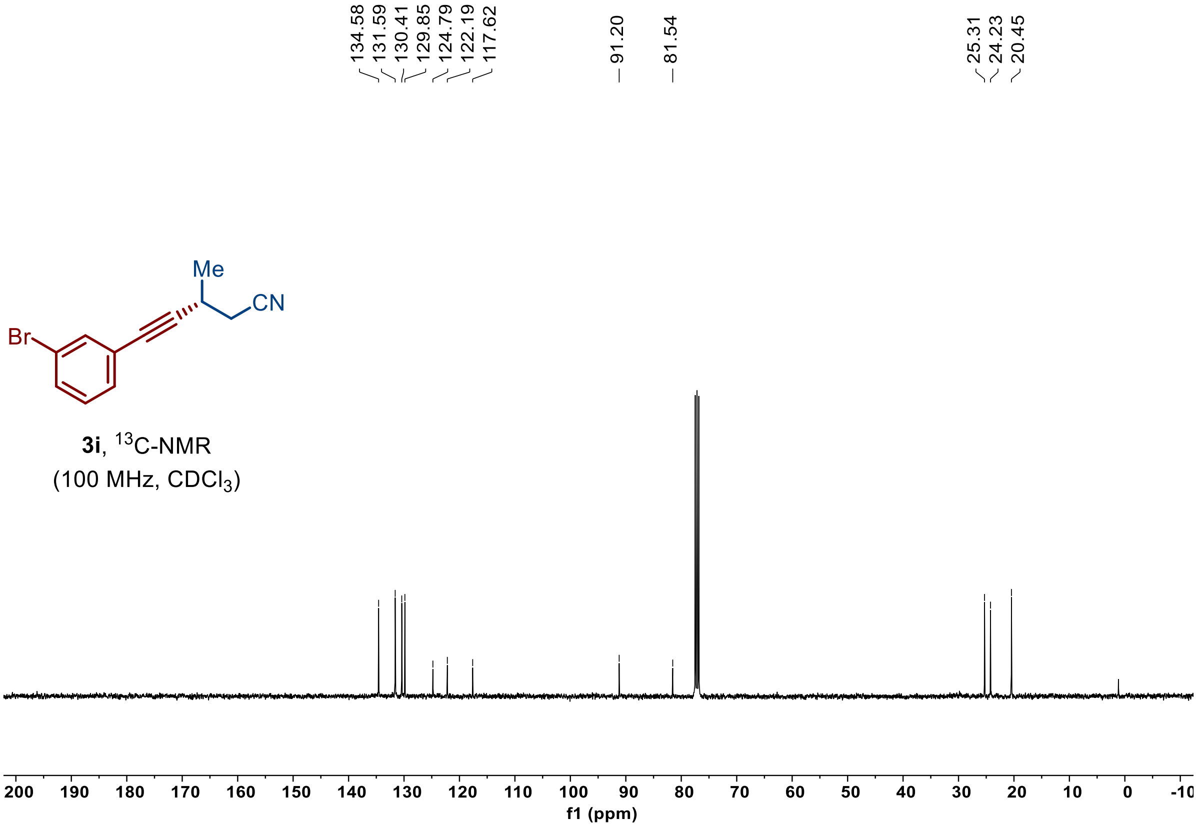

^{1}H NMR (400 MHz, $CDCl_3$) spectrum of 3j

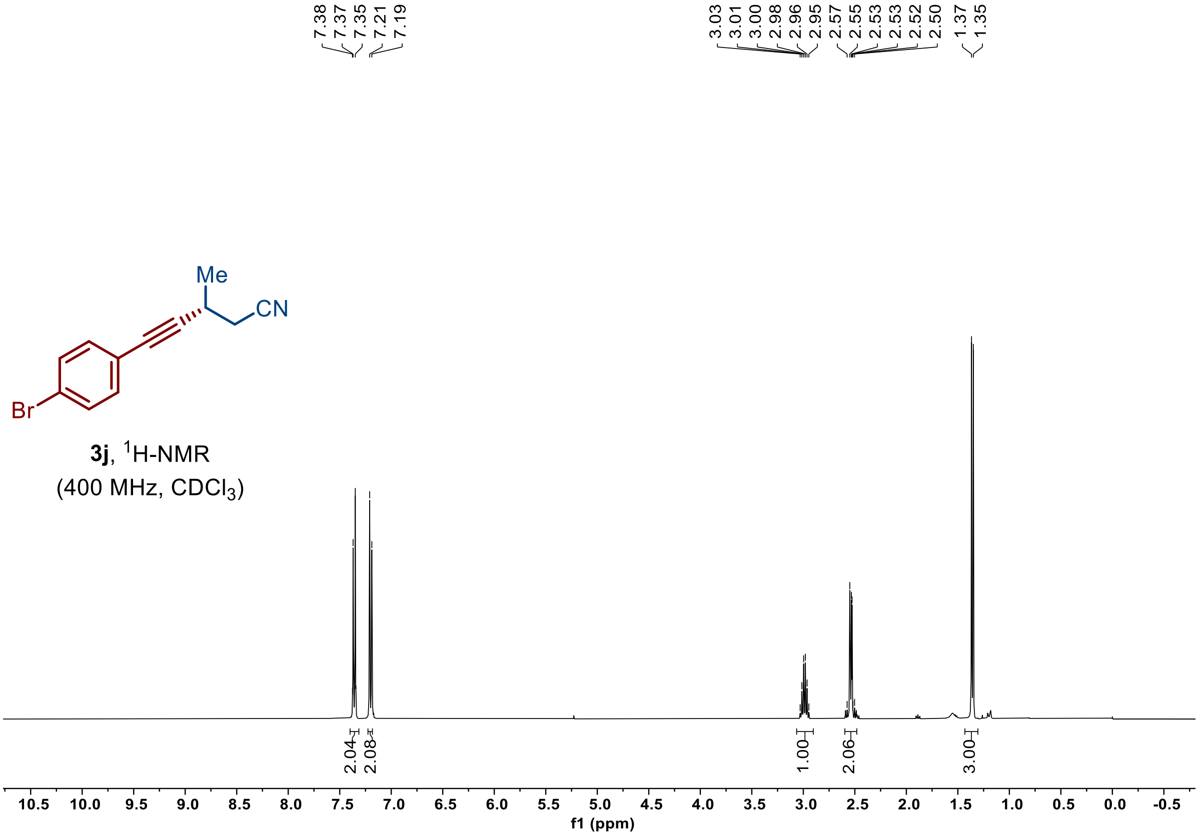

^{13}C NMR (100 MHz, $CDCl_3$) spectrum of 3j

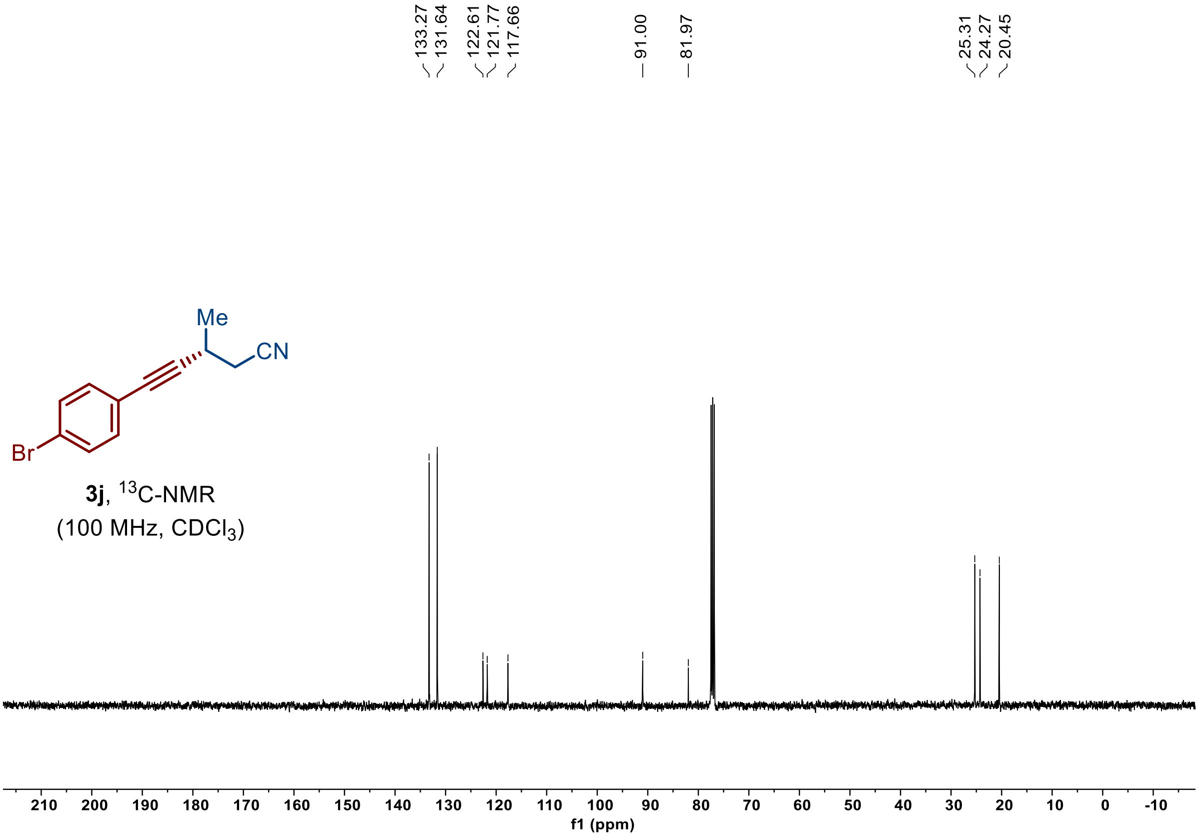

^{1}H NMR (400 MHz, $CDCl_3$) spectrum of 3k

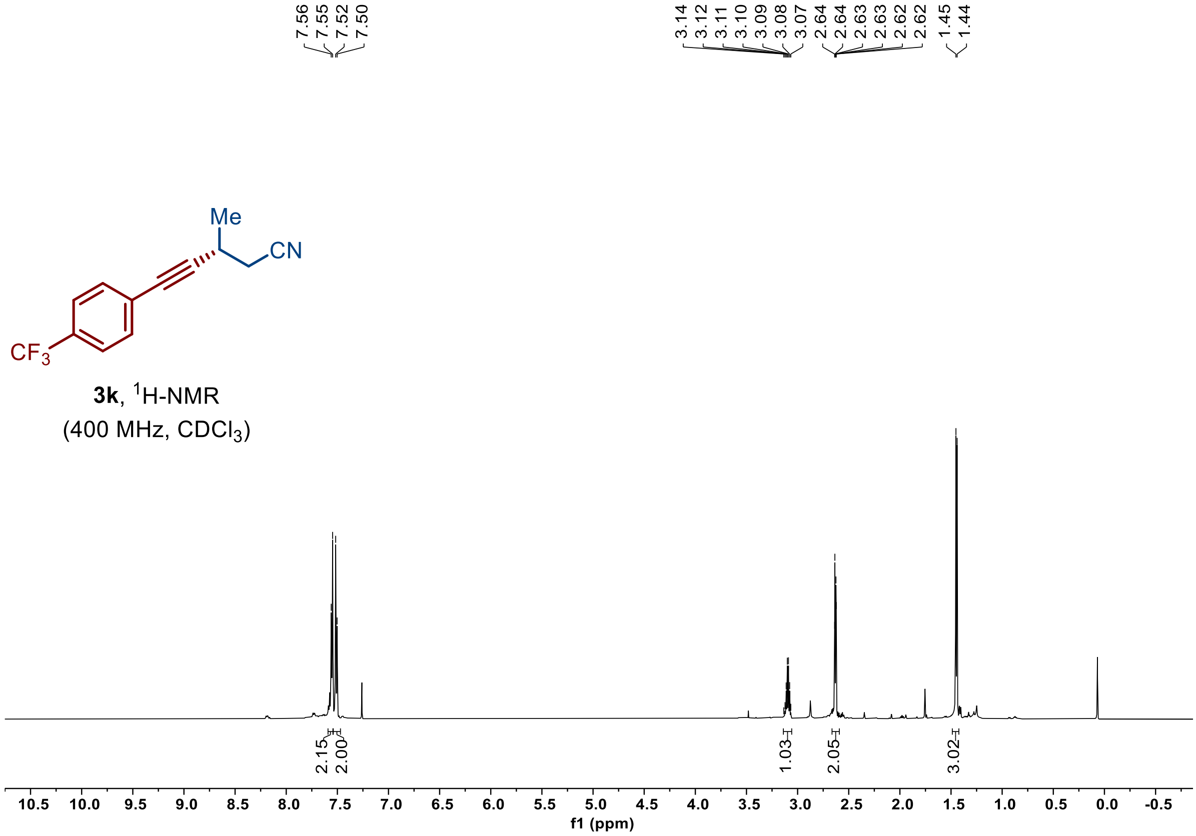

^{13}C NMR (100 MHz, $CDCl_3$) spectrum of 3k

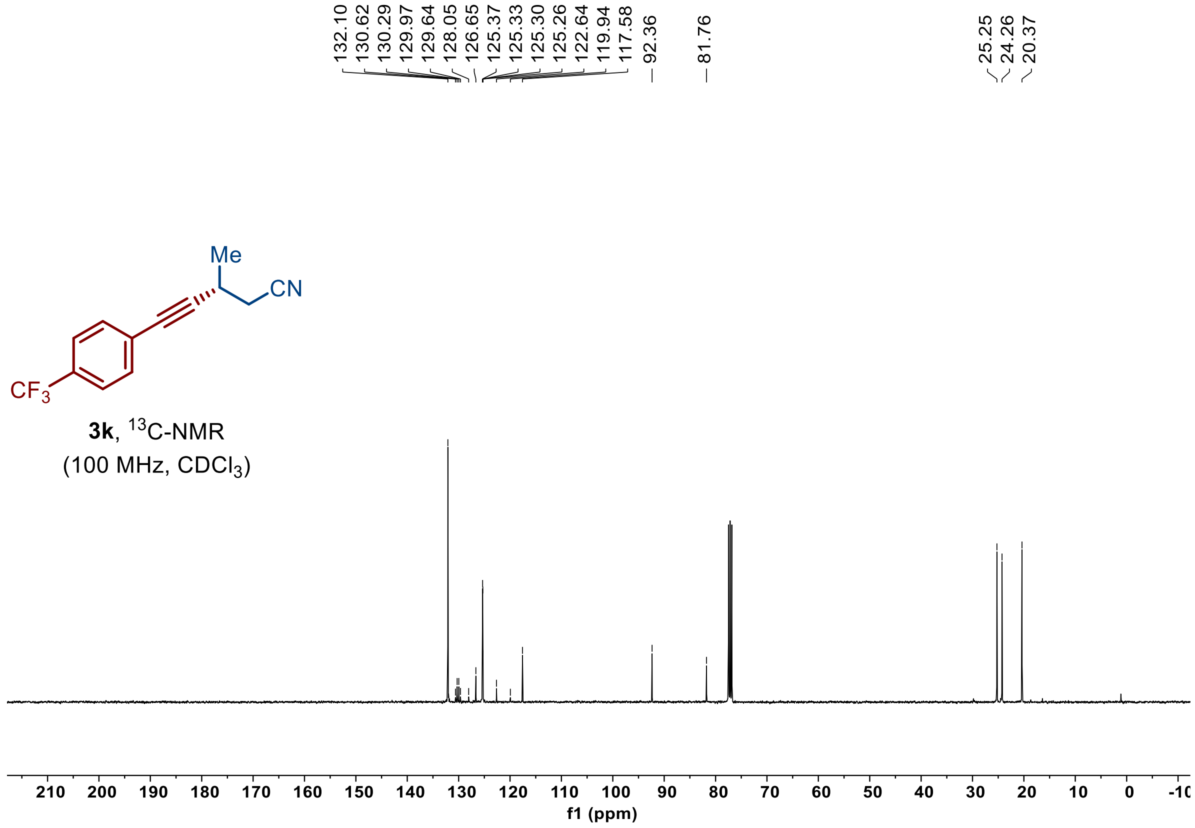

^{19}F NMR (376 MHz, CDCl3) spectrum of 3k

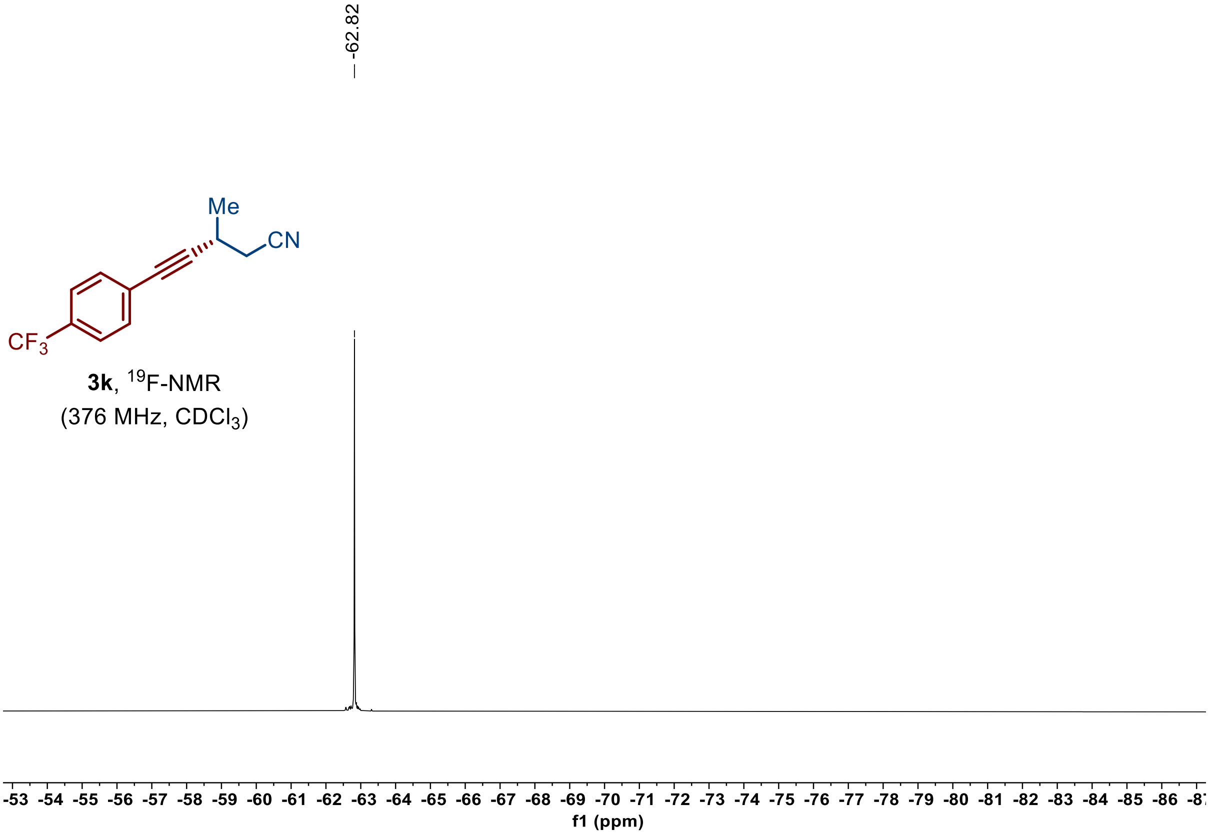

^{1}H NMR (400 MHz, $CDCl_3$) spectrum of 3l

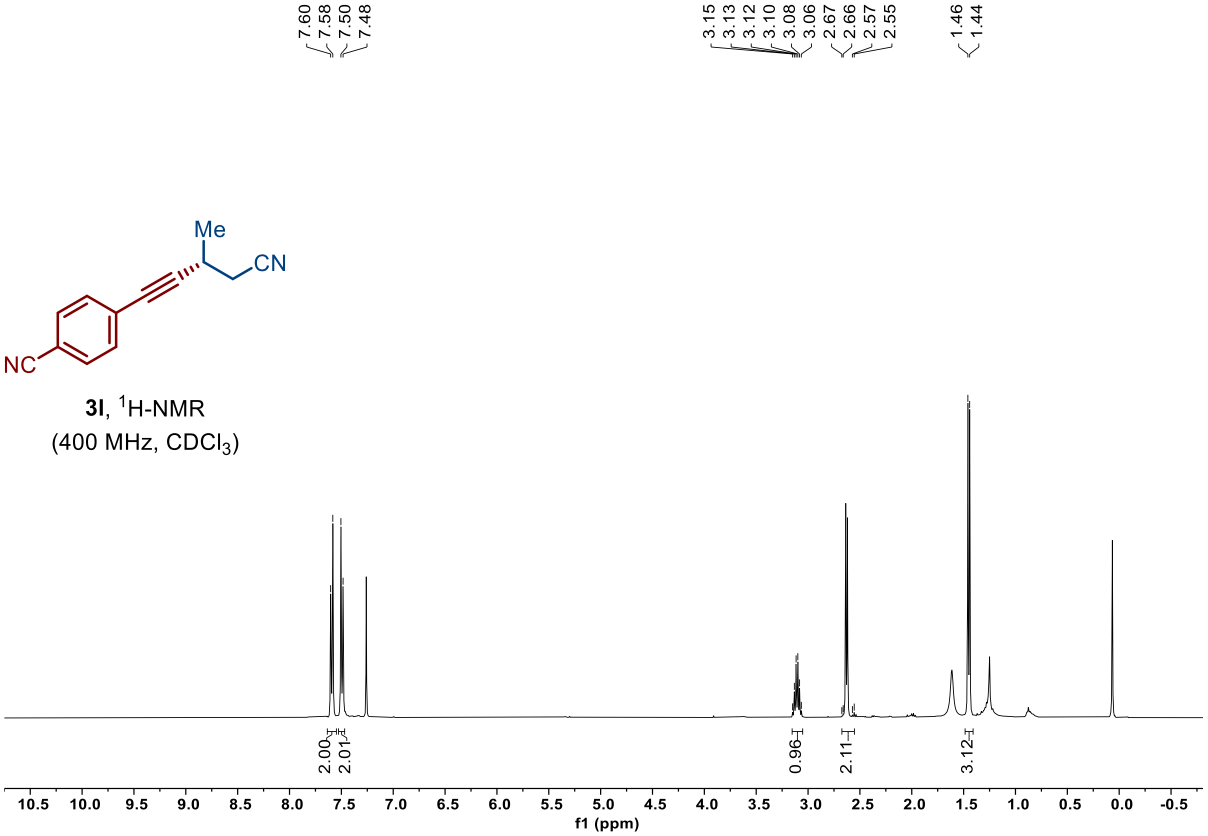

^{13}C NMR (100 MHz, $CDCl_3$) spectrum of 3l

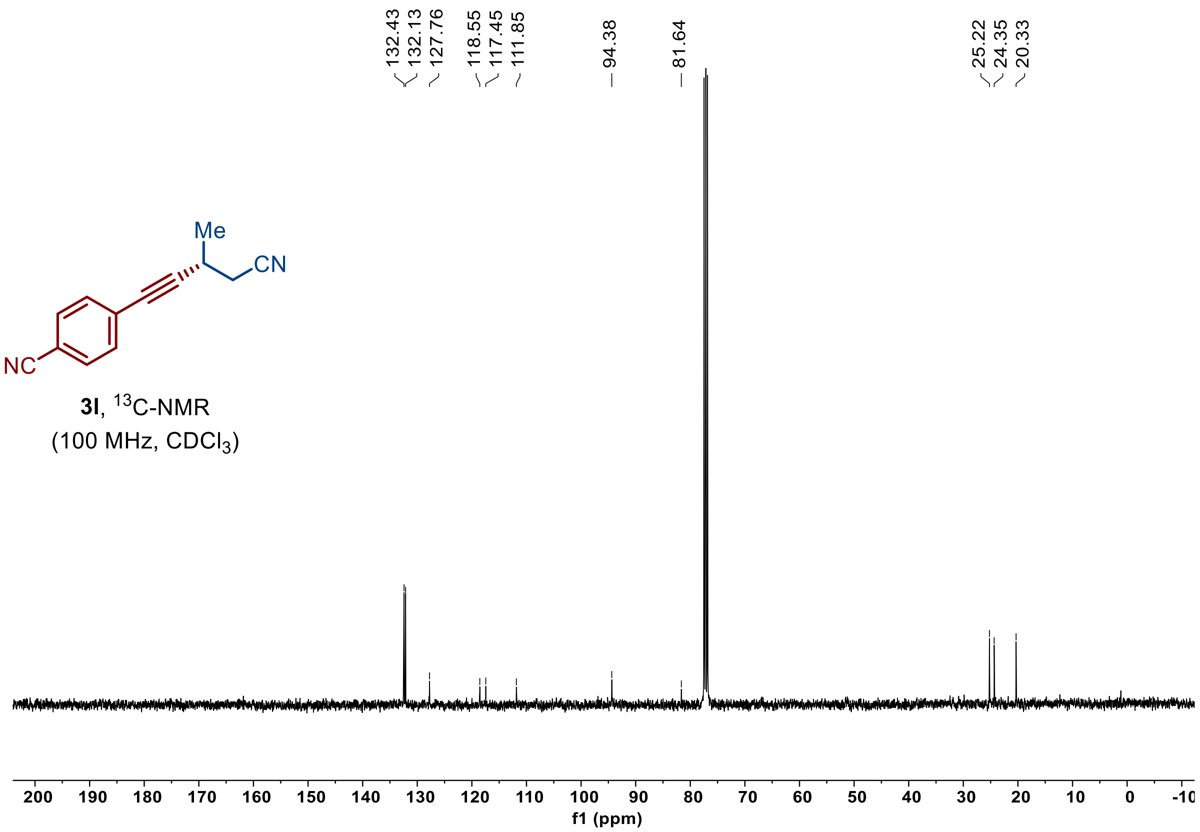

^{1}H NMR (400 MHz, $CDCl_3$) spectrum of 3m

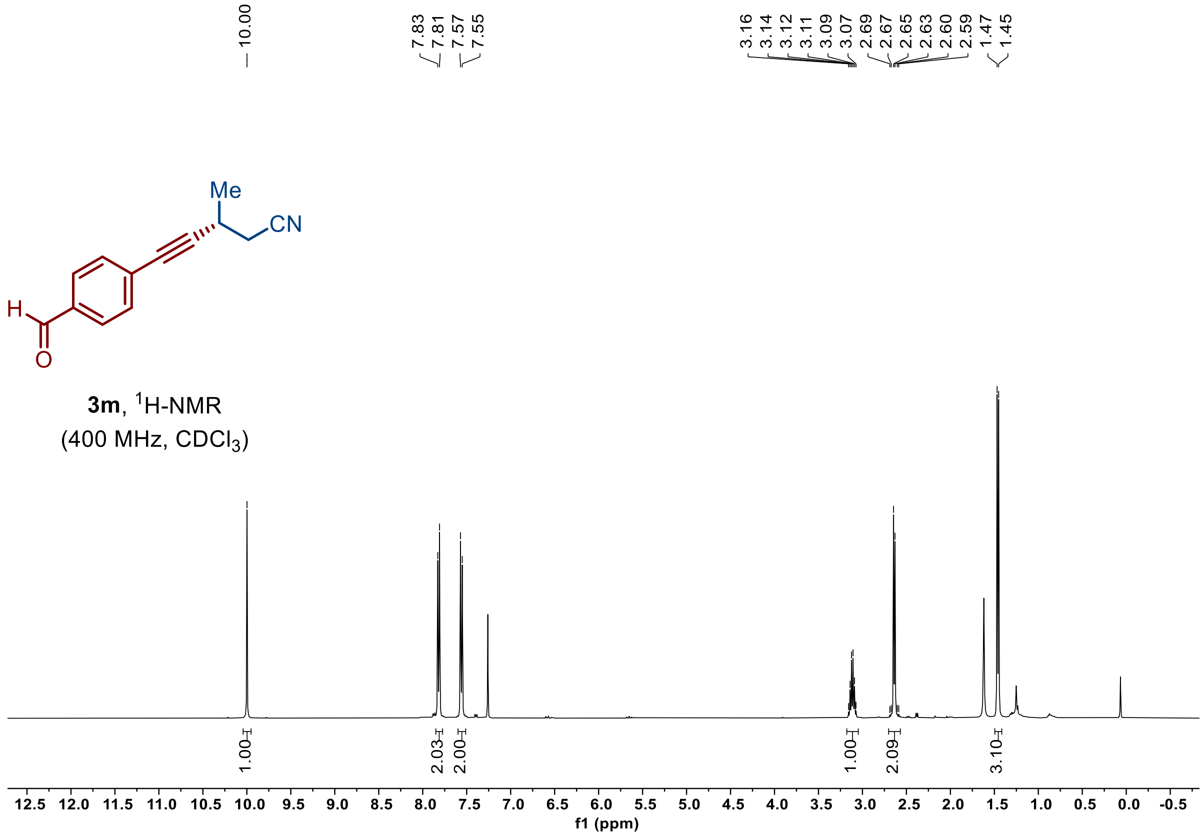

^{13}C NMR (100 MHz, $CDCl_3$) spectrum of 3m

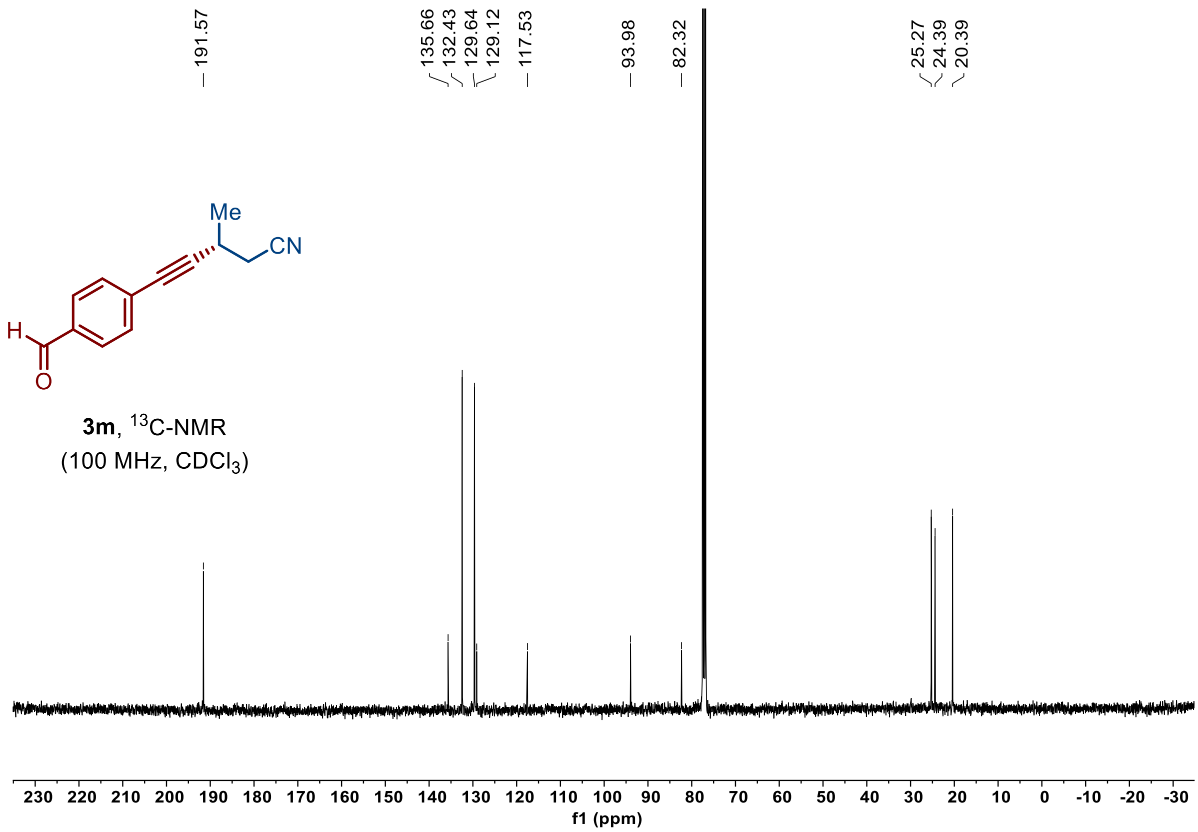

^{1}H NMR (400 MHz, $CDCl_3$) spectrum of 3n

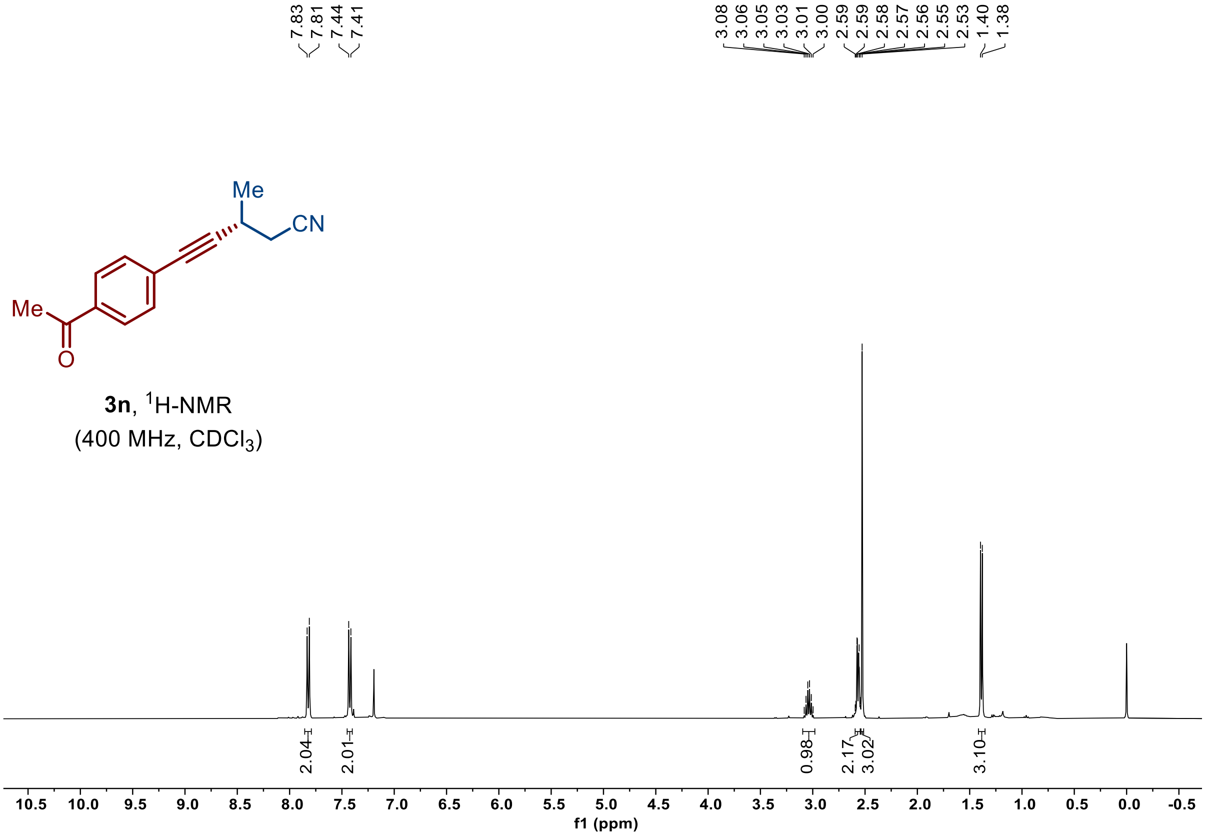

^{13}C NMR (100 MHz, $CDCl_3$) spectrum of 3n

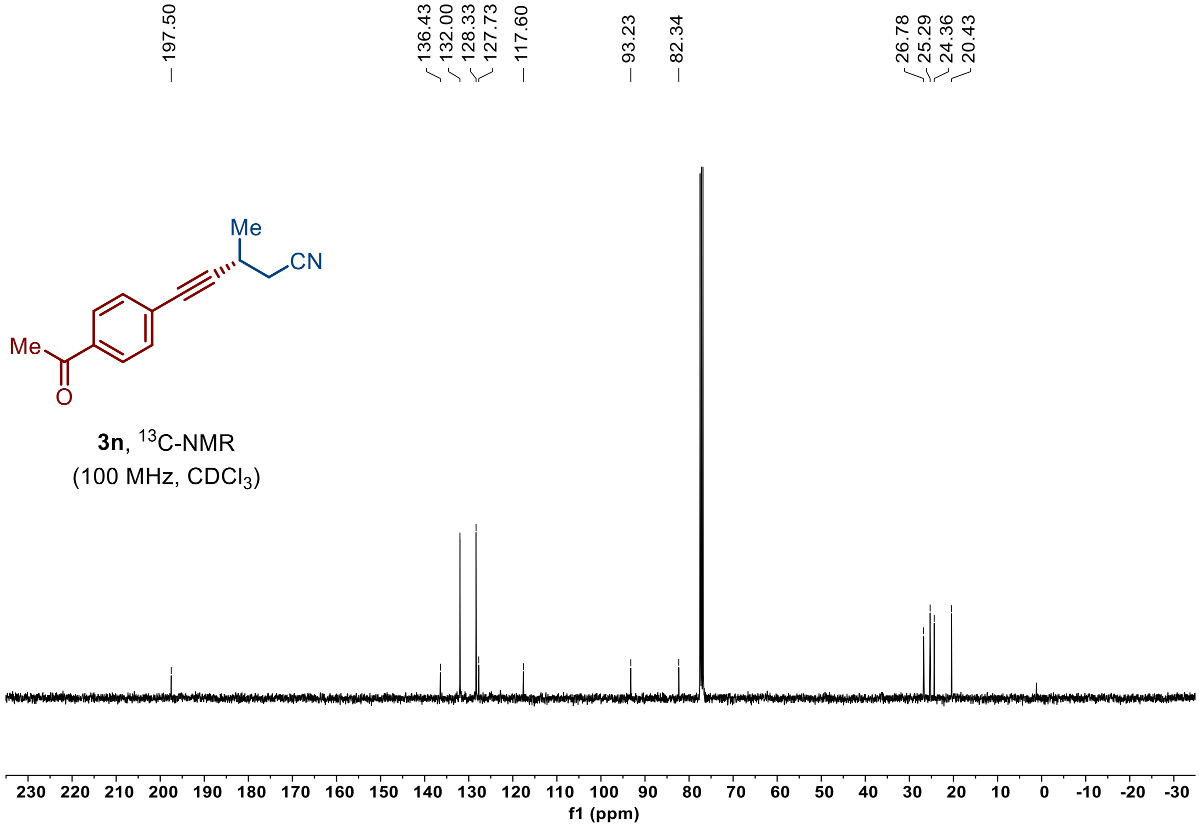

^{1}H NMR (400 MHz, $CDCl_3$) spectrum of 3o

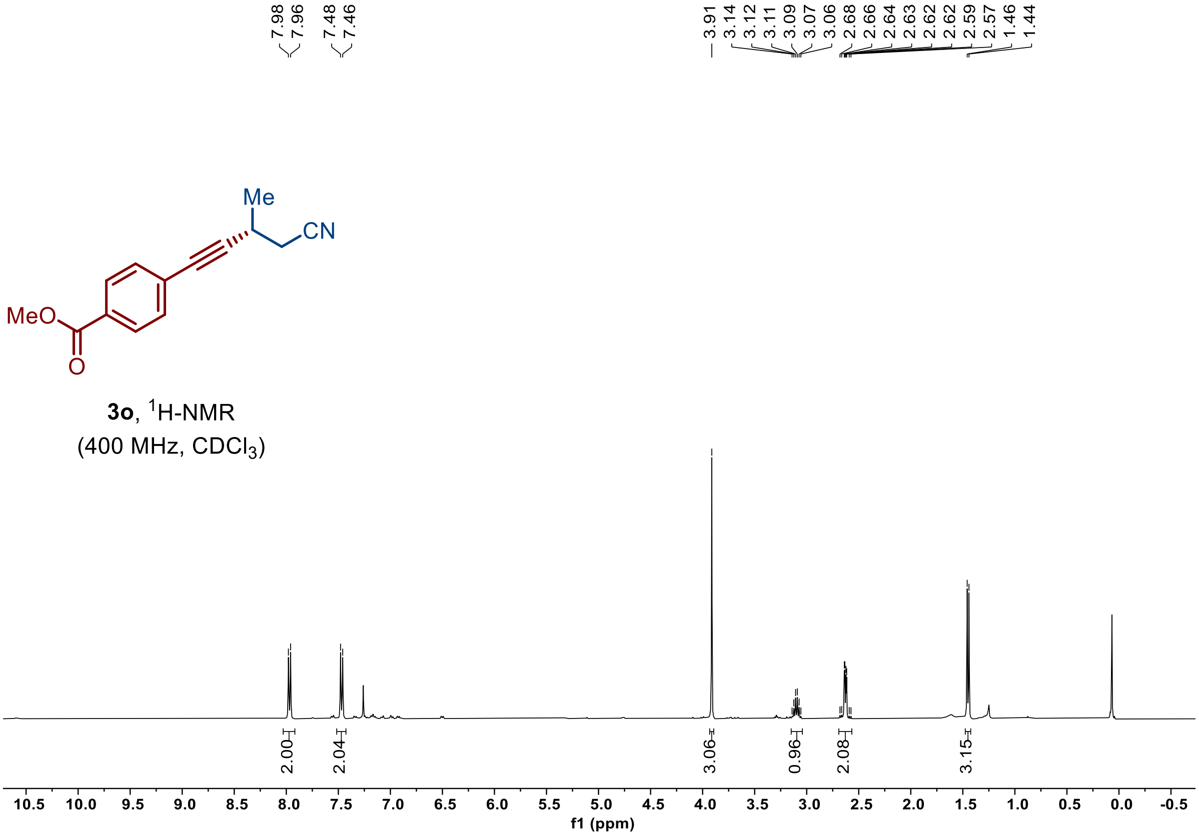

^{13}C NMR (100 MHz, $CDCl_3$) spectrum of 3o

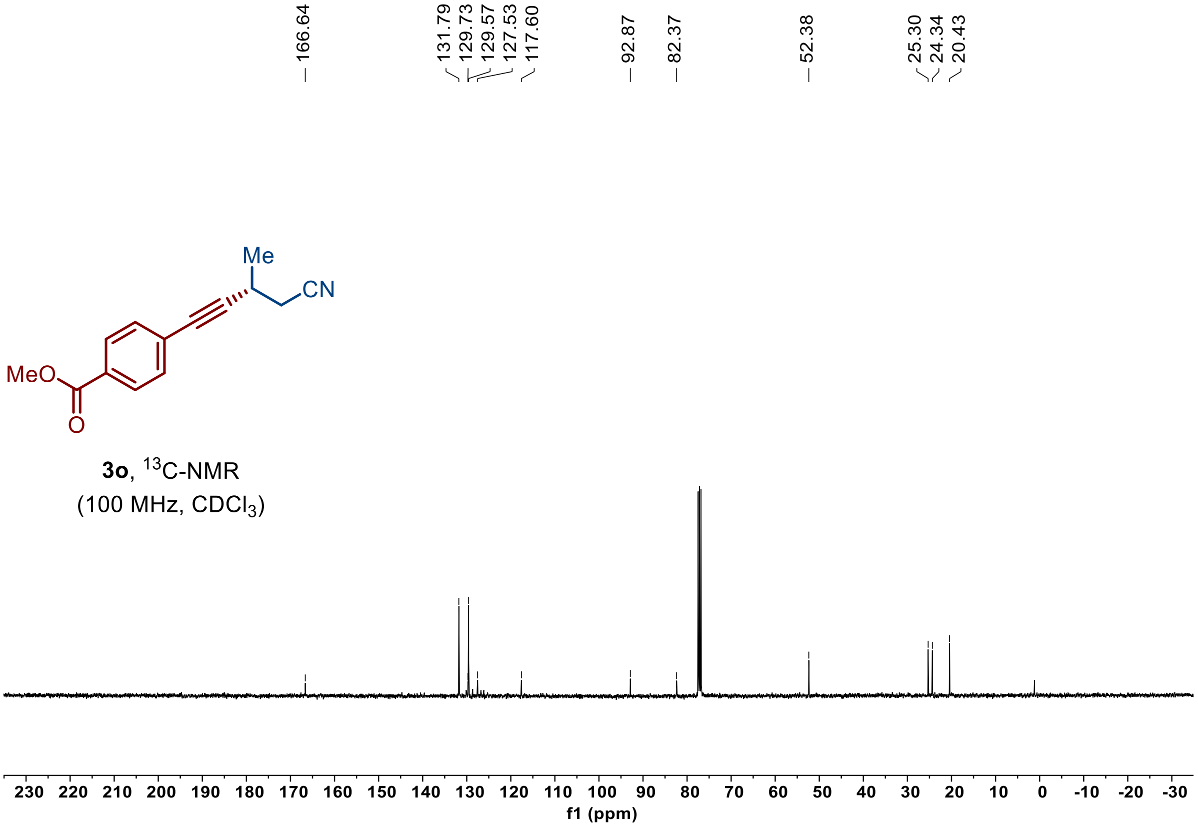

^{1}H NMR (400 MHz, $CDCl_3$) spectrum of 3p

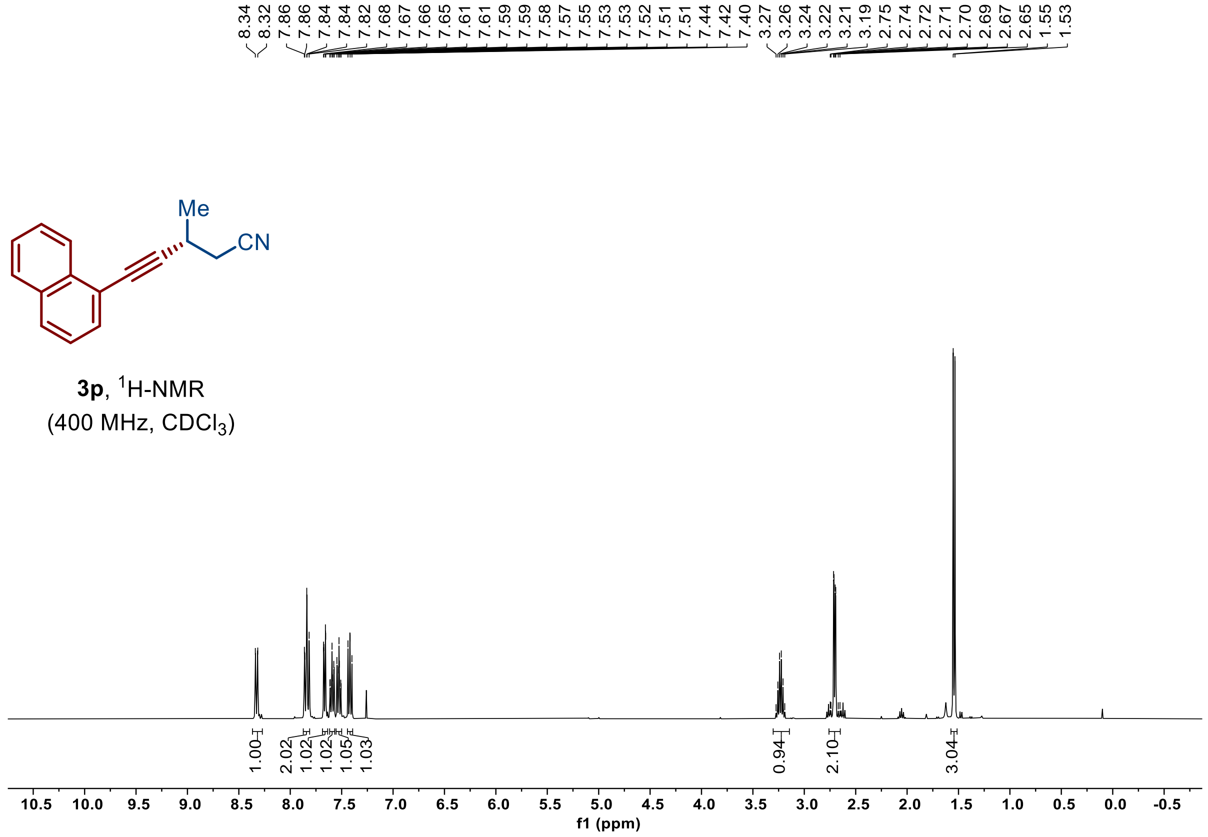

^{13}C NMR (100 MHz, $CDCl_3$) spectrum of 3p

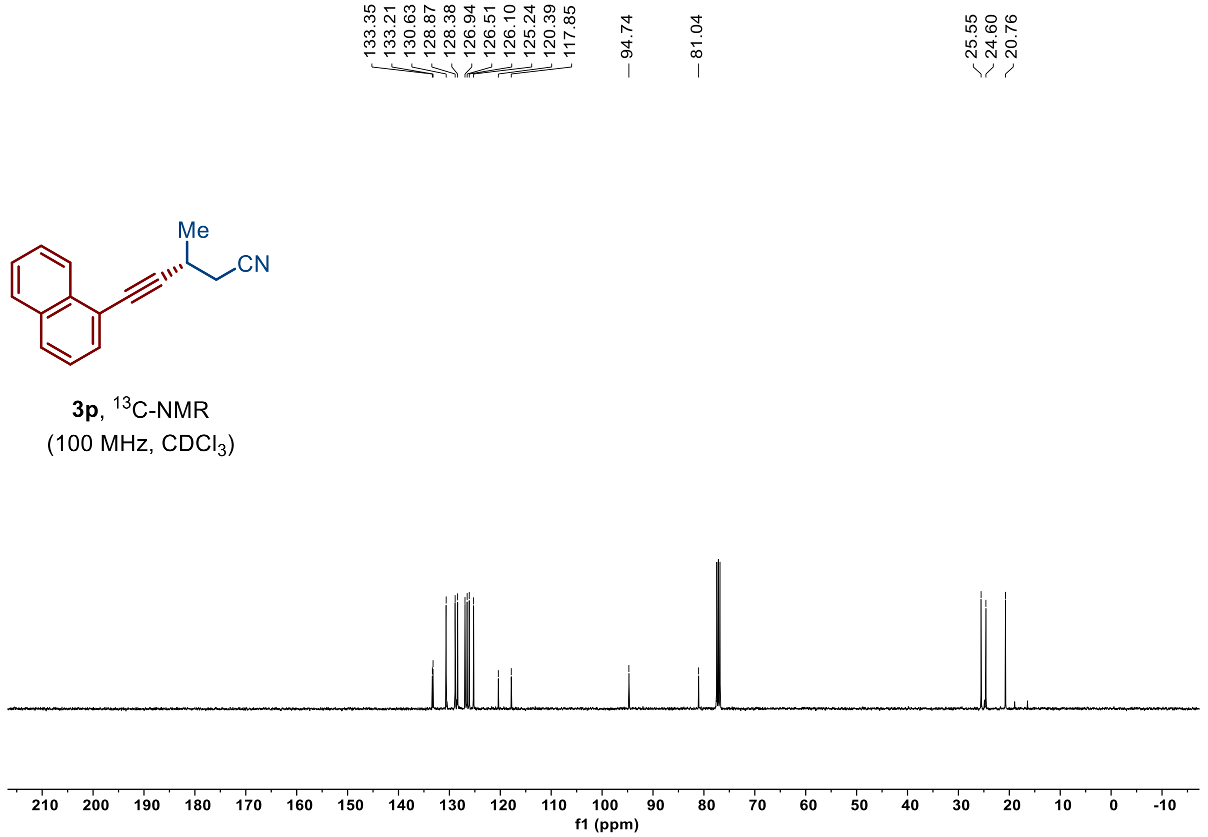

^{1}H NMR (400 MHz, CDCl₃) spectrum of 3q

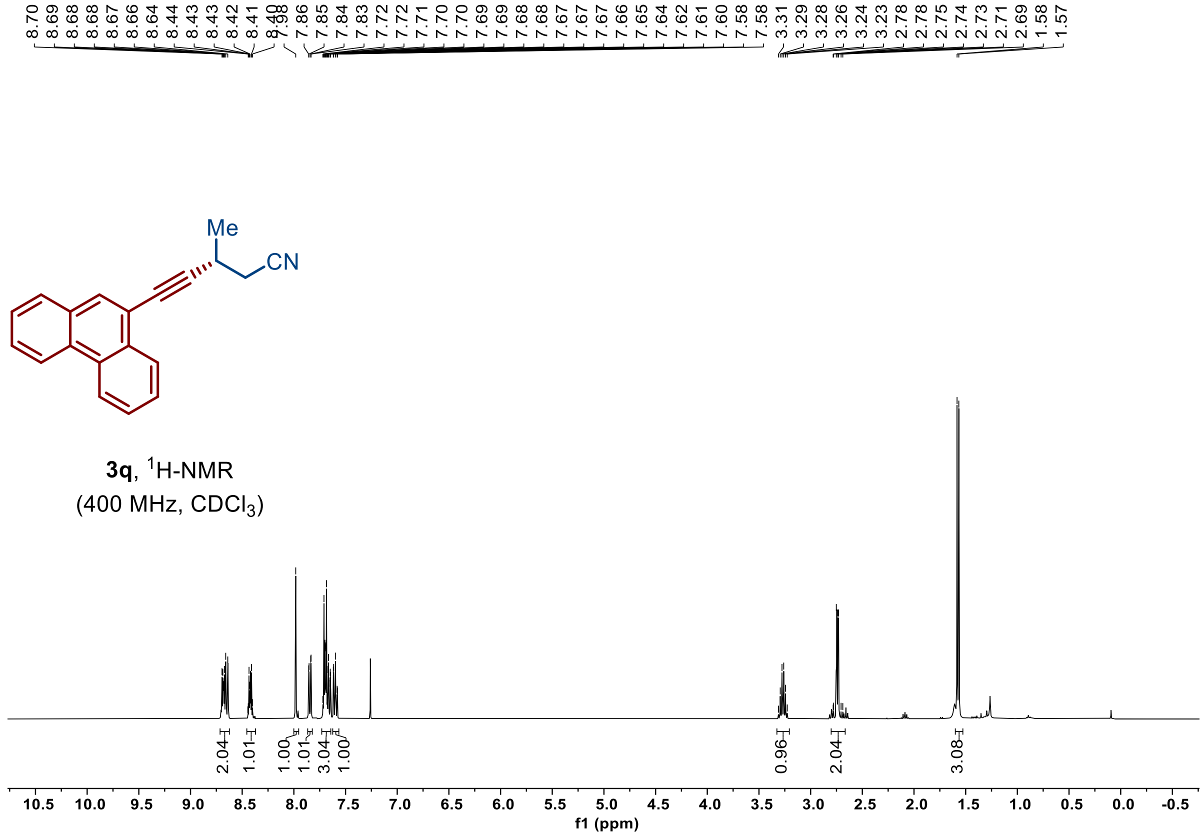

^{13}C NMR (100 MHz, CDCl₃) spectrum of 3q

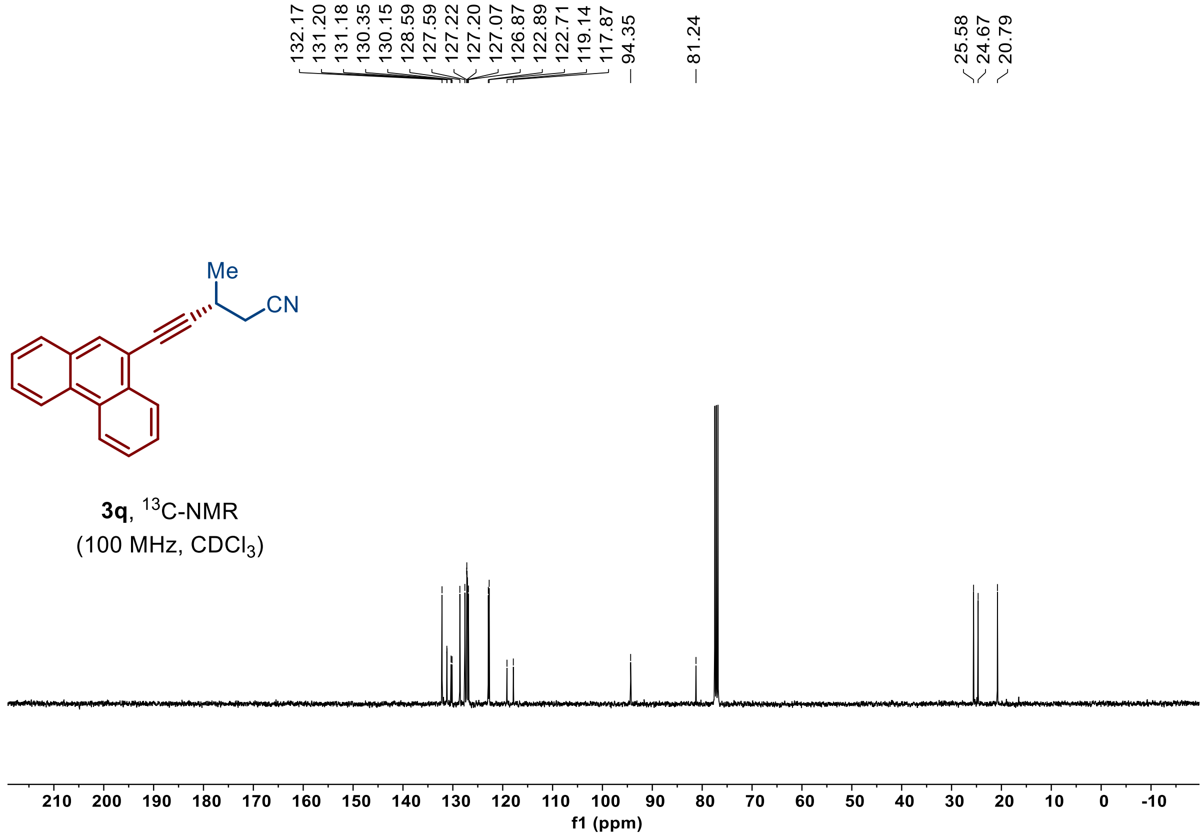

^{1}H NMR (400 MHz, CDCl$_3$) spectrum of 3r

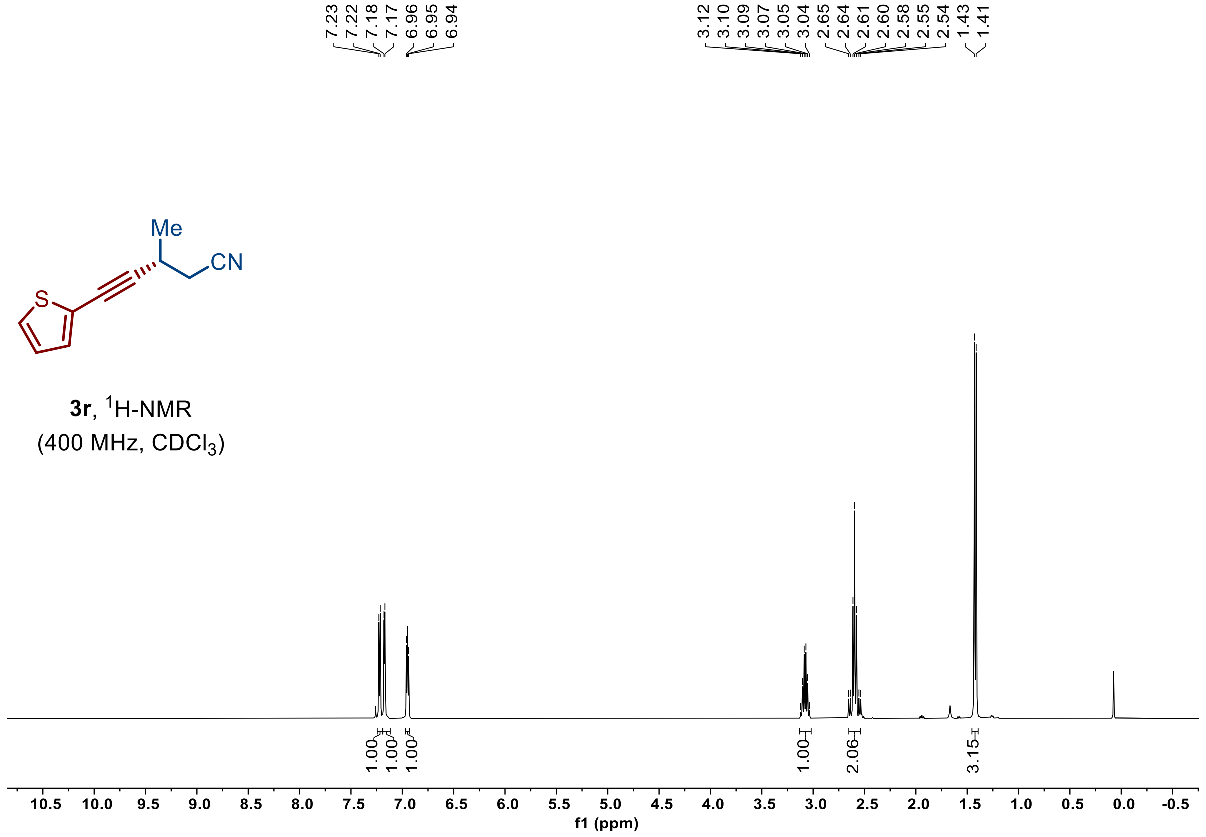

^{13}C NMR (100 MHz, CDCl$_3$) spectrum of 3r

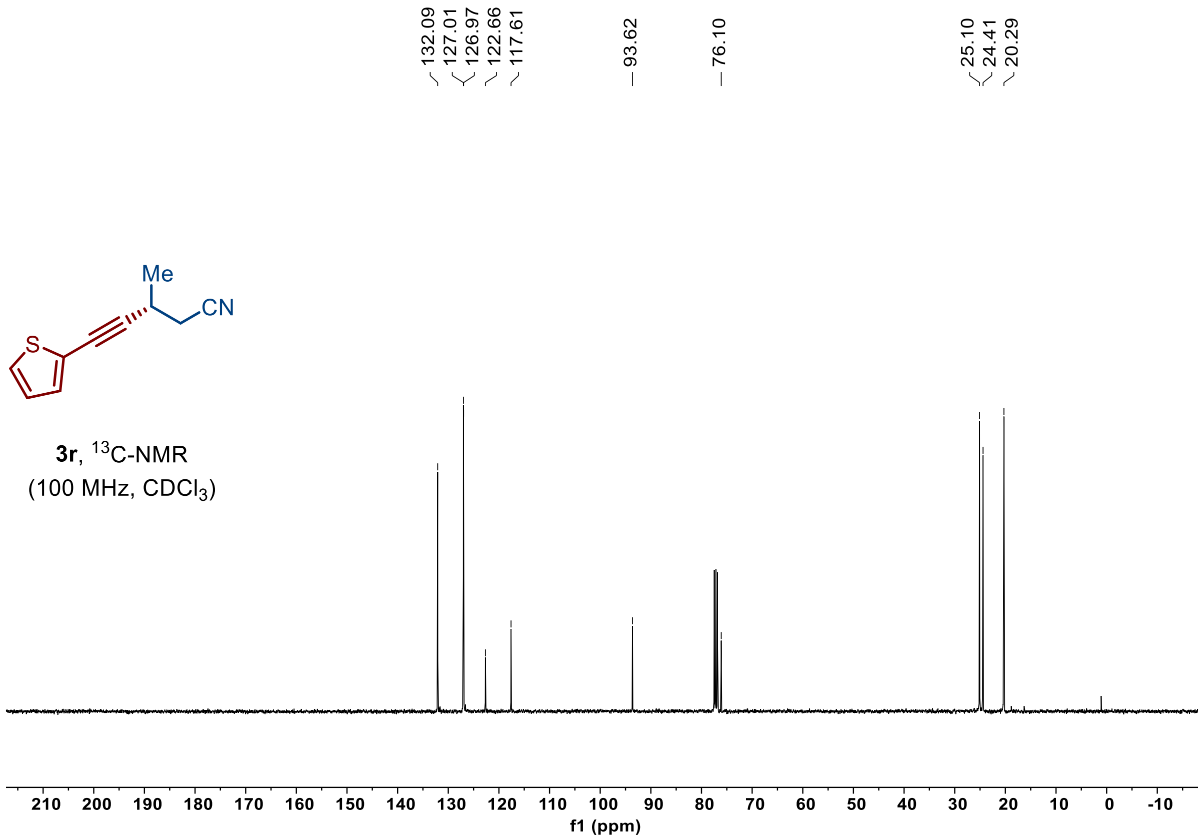

^{1}H NMR (400 MHz, $CDCl_3$) spectrum of 3s

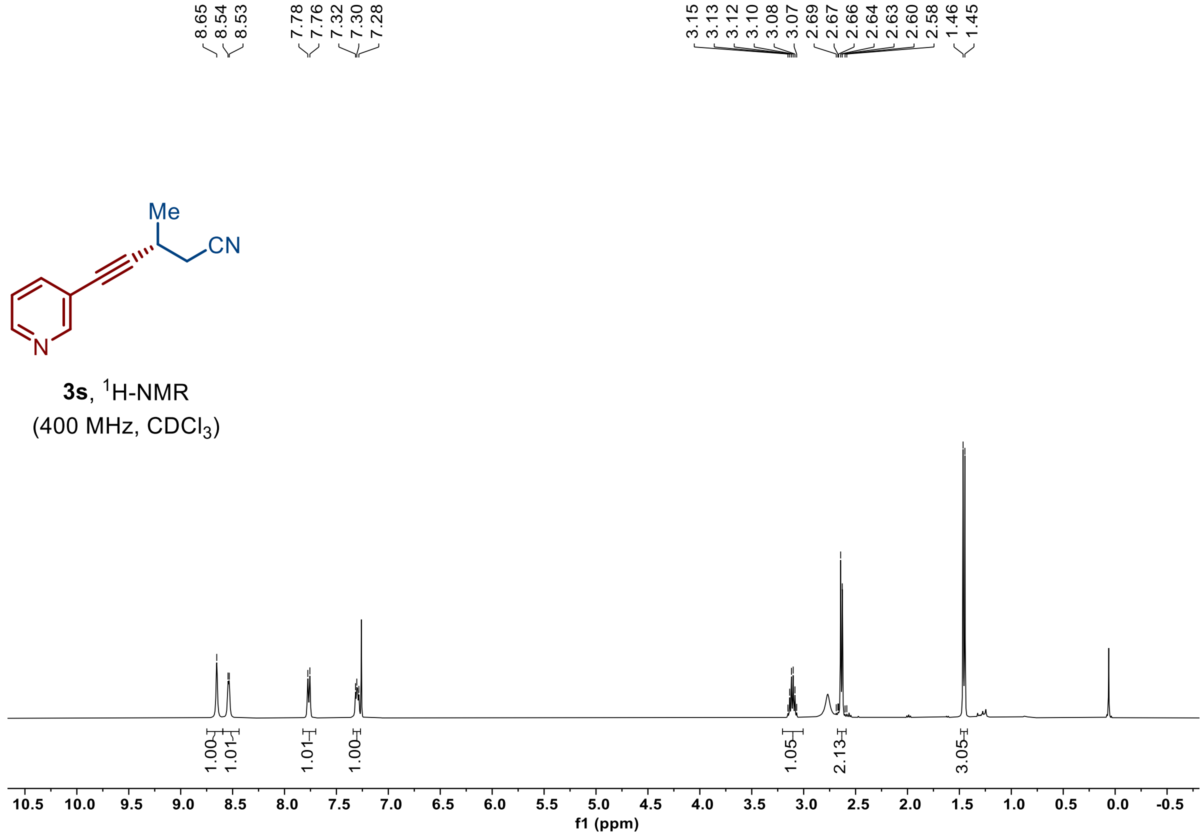

^{13}C NMR (100 MHz, $CDCl_3$) spectrum of 3s

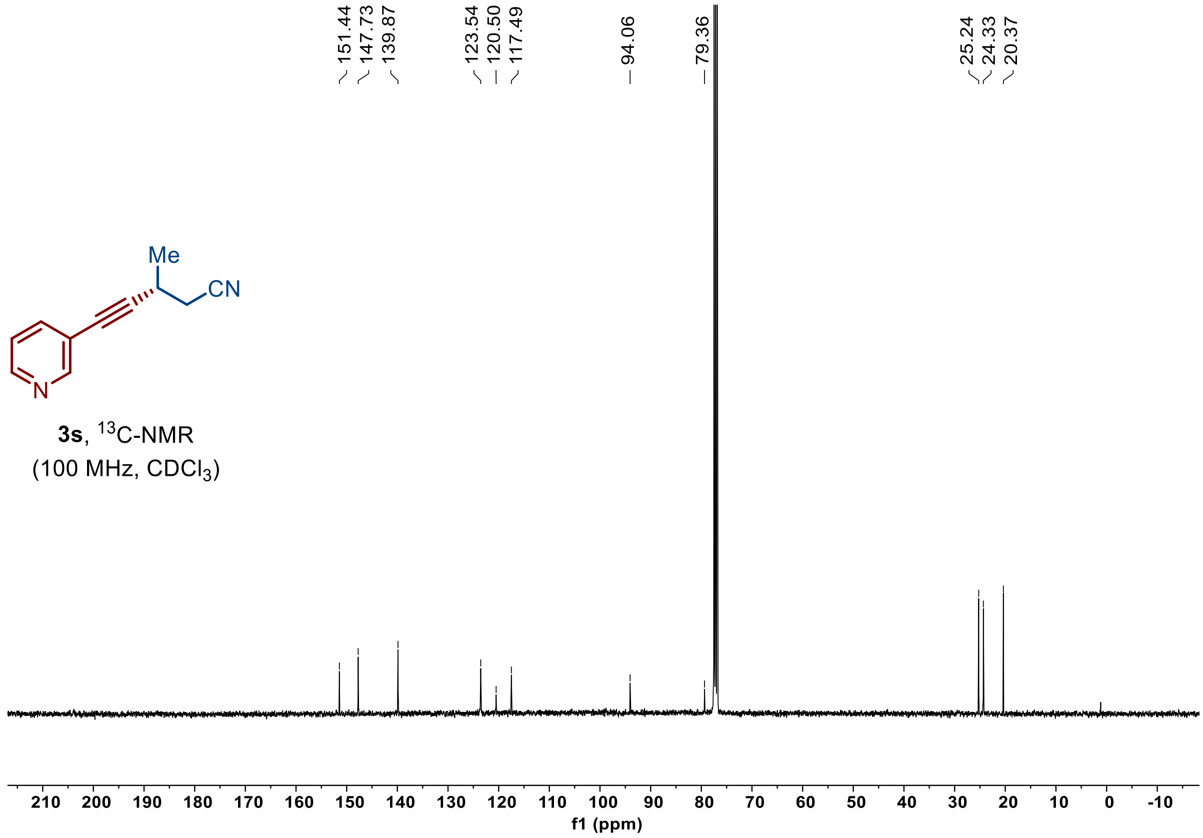

^{1}H NMR (400 MHz, CDCl$_3$) spectrum of 3t

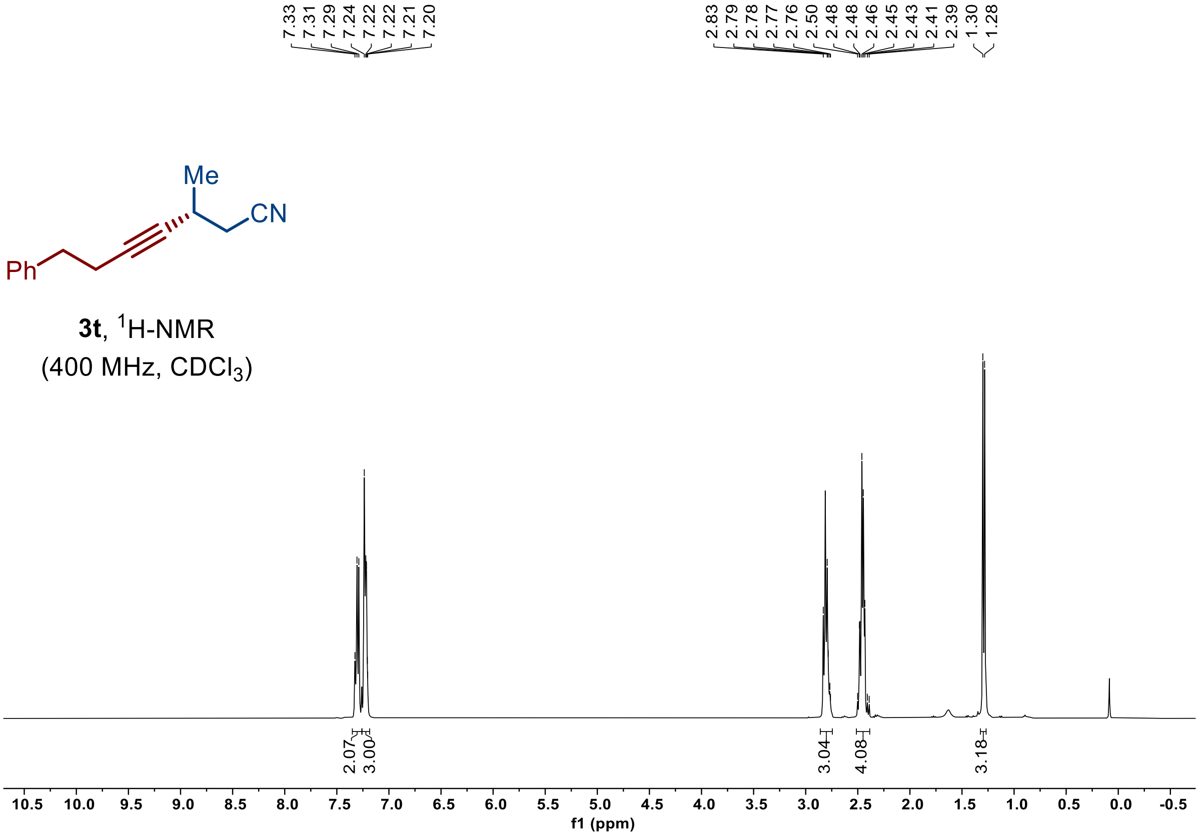

^{13}C NMR (100 MHz, CDCl$_3$) spectrum of 3t

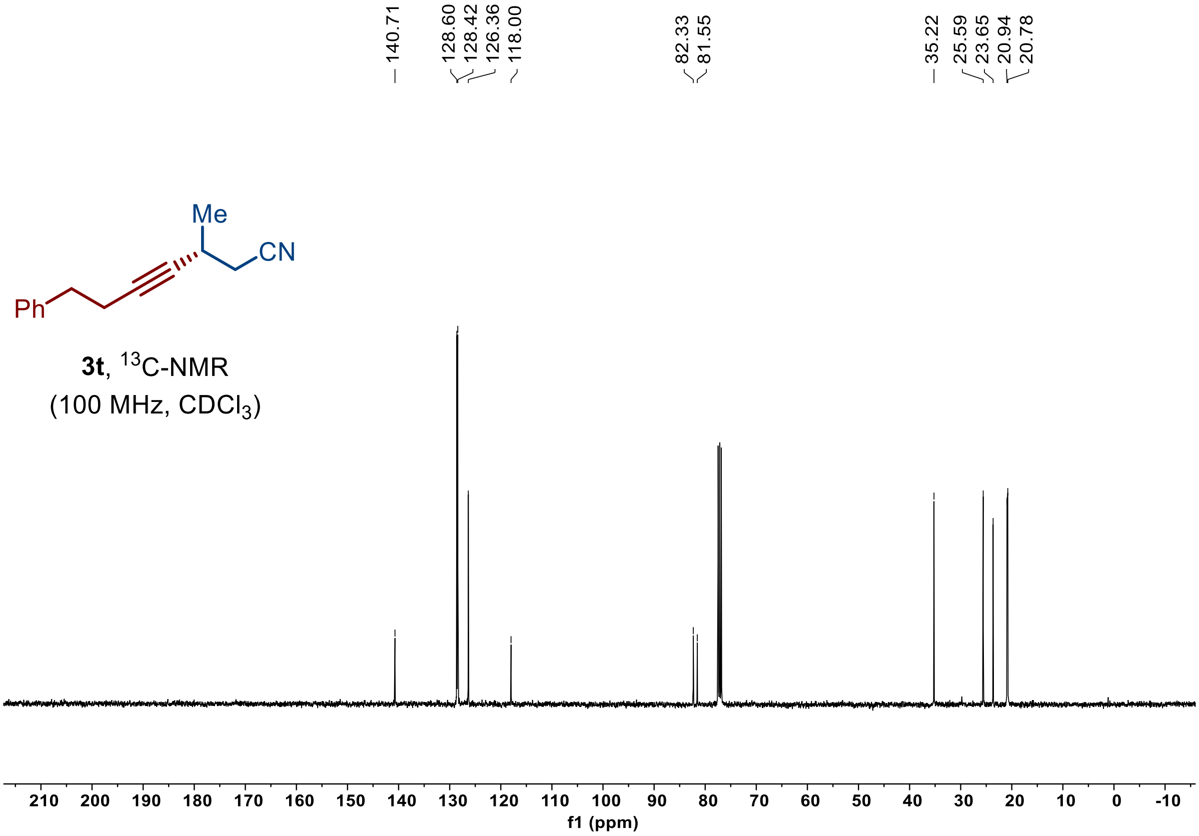

^{1}H NMR (400 MHz, $CDCl_3$) spectrum of 3u

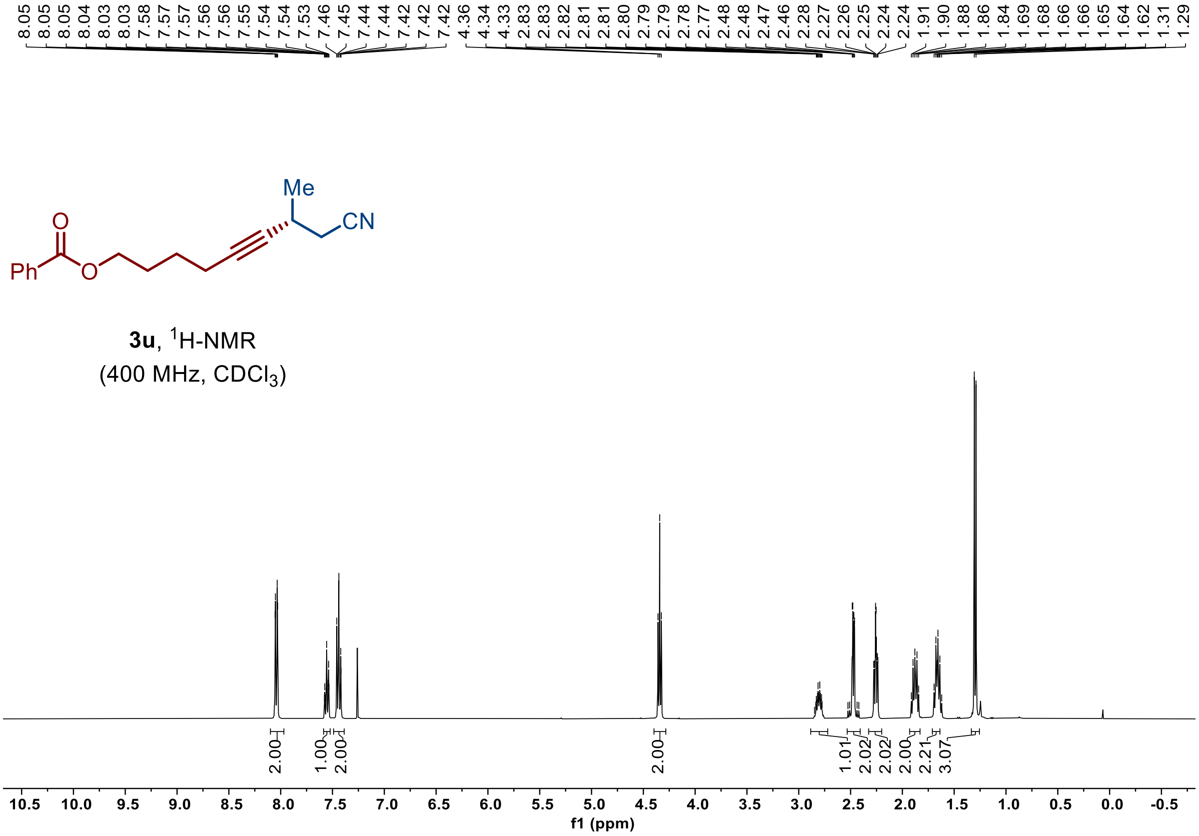

^{13}C NMR (100 MHz, $CDCl_3$) spectrum of 3u

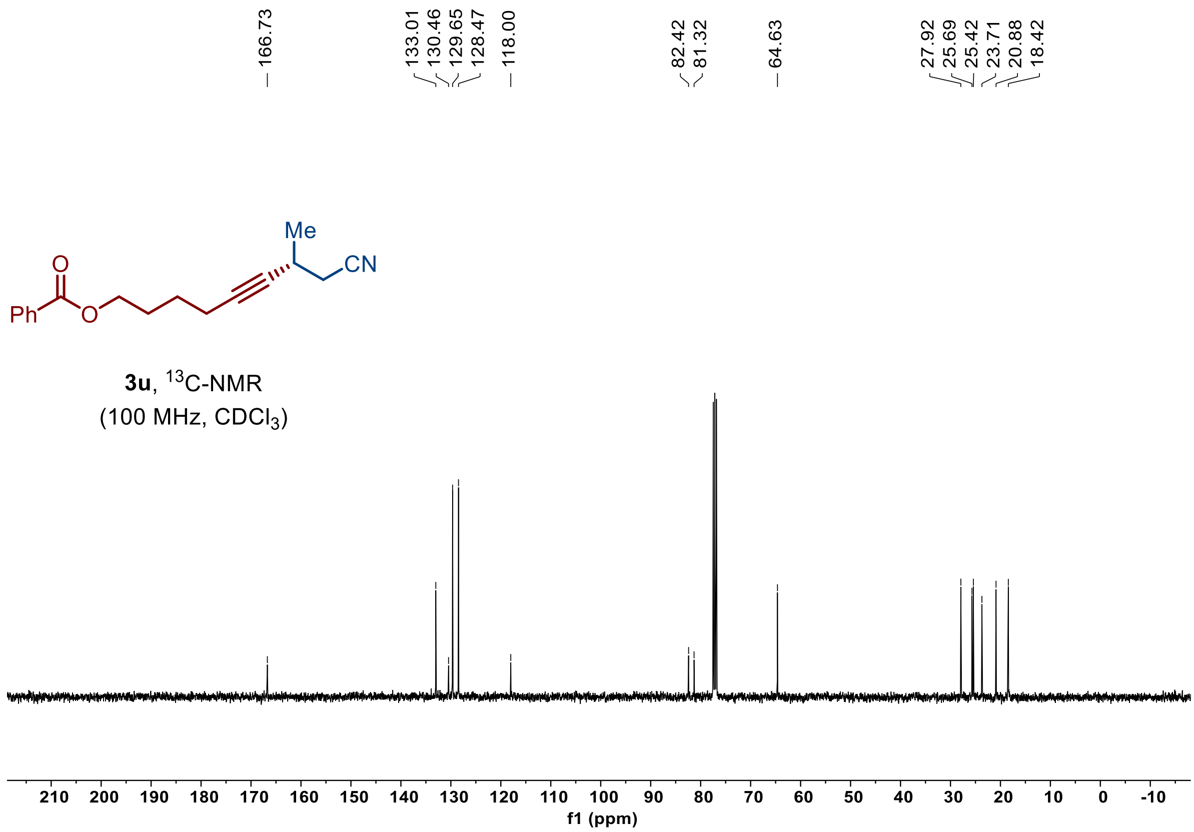

^{1}H NMR (400 MHz, CDCl$_3$) spectrum of 3v

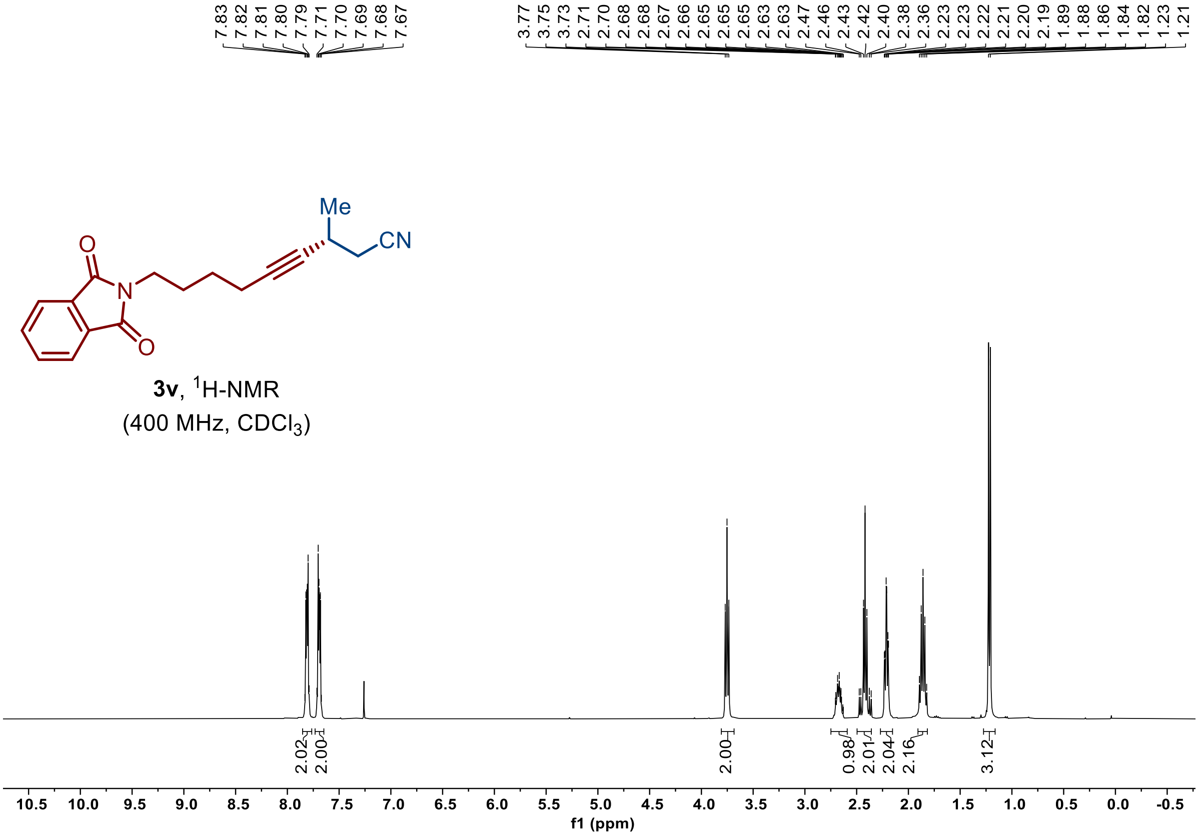

^{13}C NMR (100 MHz, CDCl$_3$) spectrum of 3v

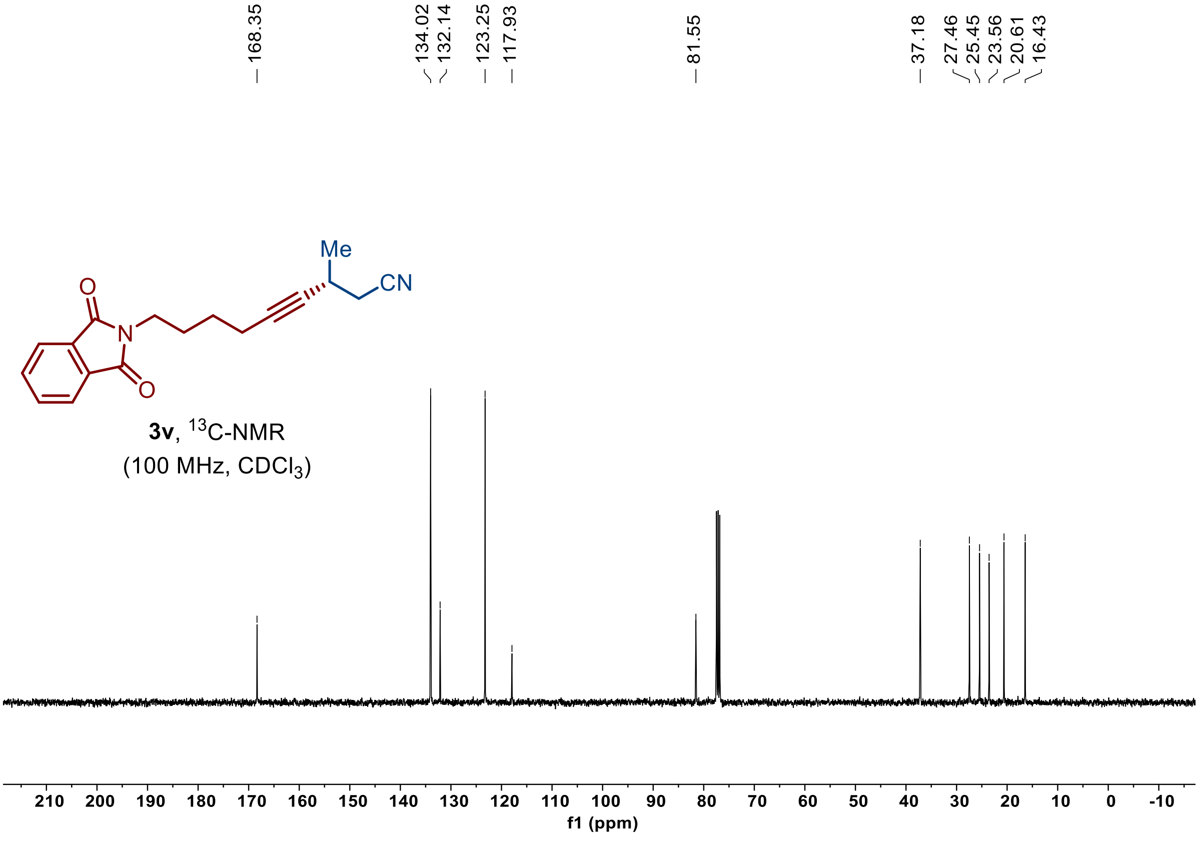

^{1}H NMR (400 MHz, $CDCl_3$) spectrum of 4a

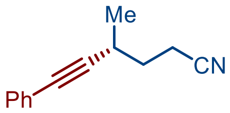

4a, ^{1}H-NMR
(400 MHz, $CDCl_3$)

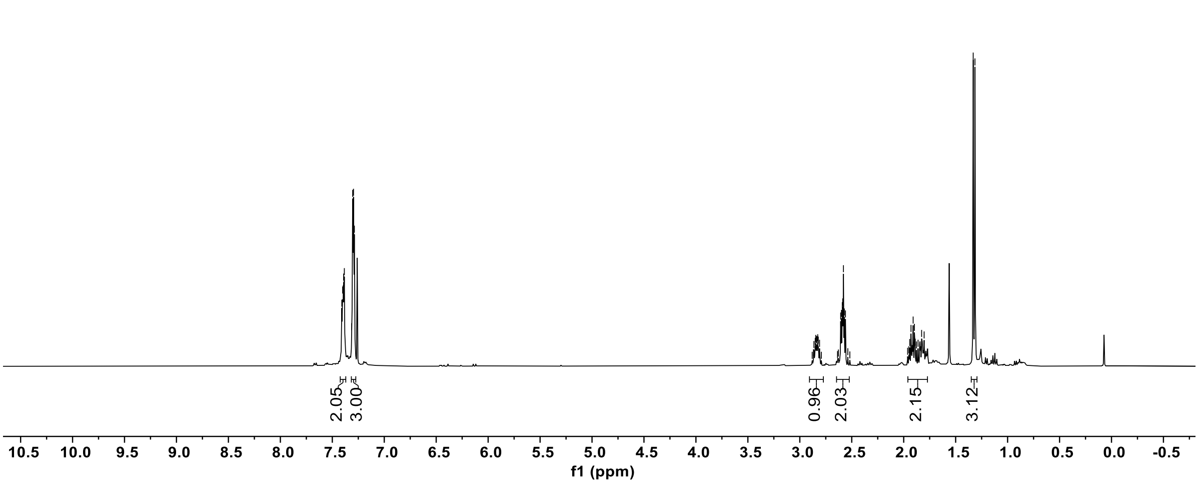

^{13}C NMR (100 MHz, $CDCl_3$) spectrum of 4a

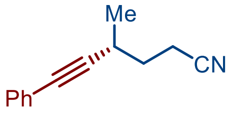

4a, ^{13}C-NMR
(100 MHz, $CDCl_3$)

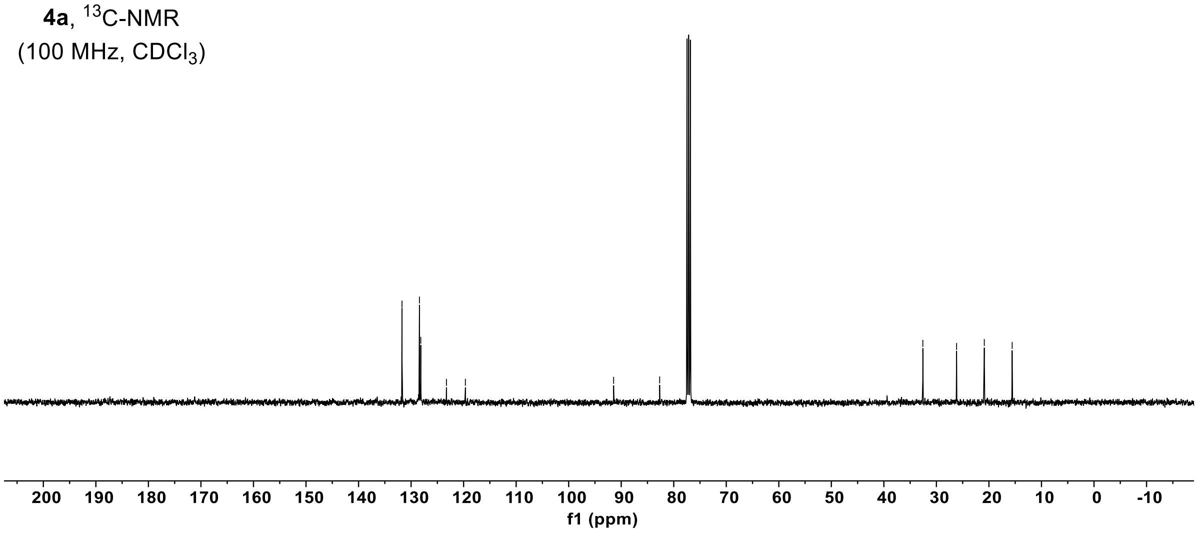

^{1}H NMR (400 MHz, $CDCl_3$) spectrum of 4b

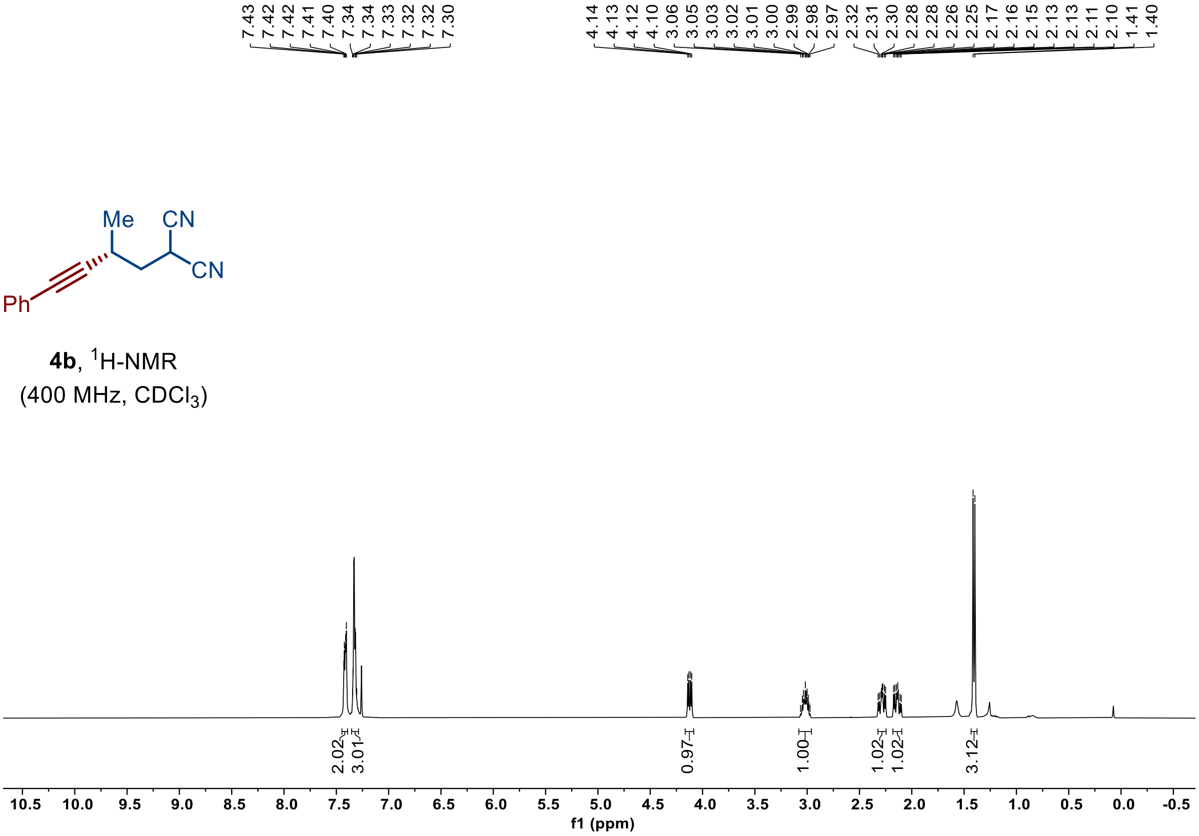

4b, ^{1}H-NMR (400 MHz, $CDCl_3$)

^{13}C NMR (100 MHz, $CDCl_3$) spectrum of 4b

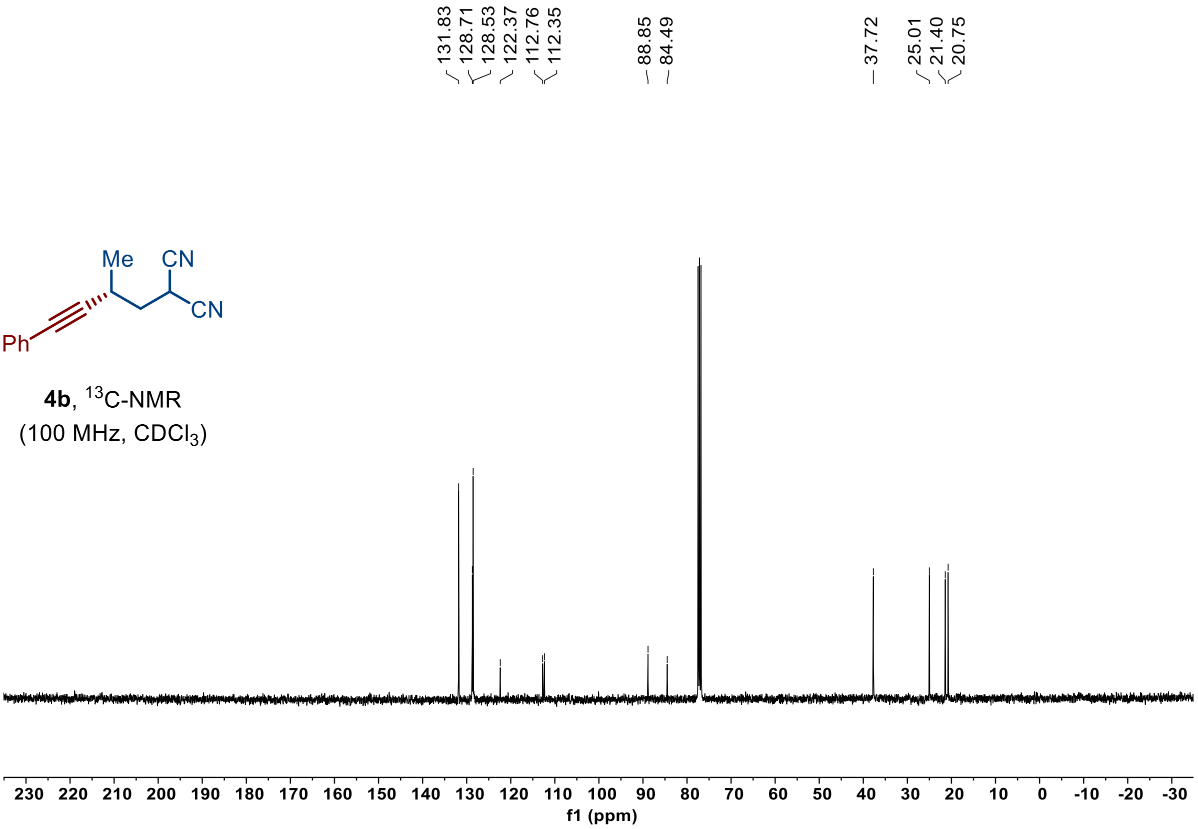

4b, ^{13}C-NMR (100 MHz, $CDCl_3$)

^{1}H NMR (400 MHz, $CDCl_3$) spectrum of 4c

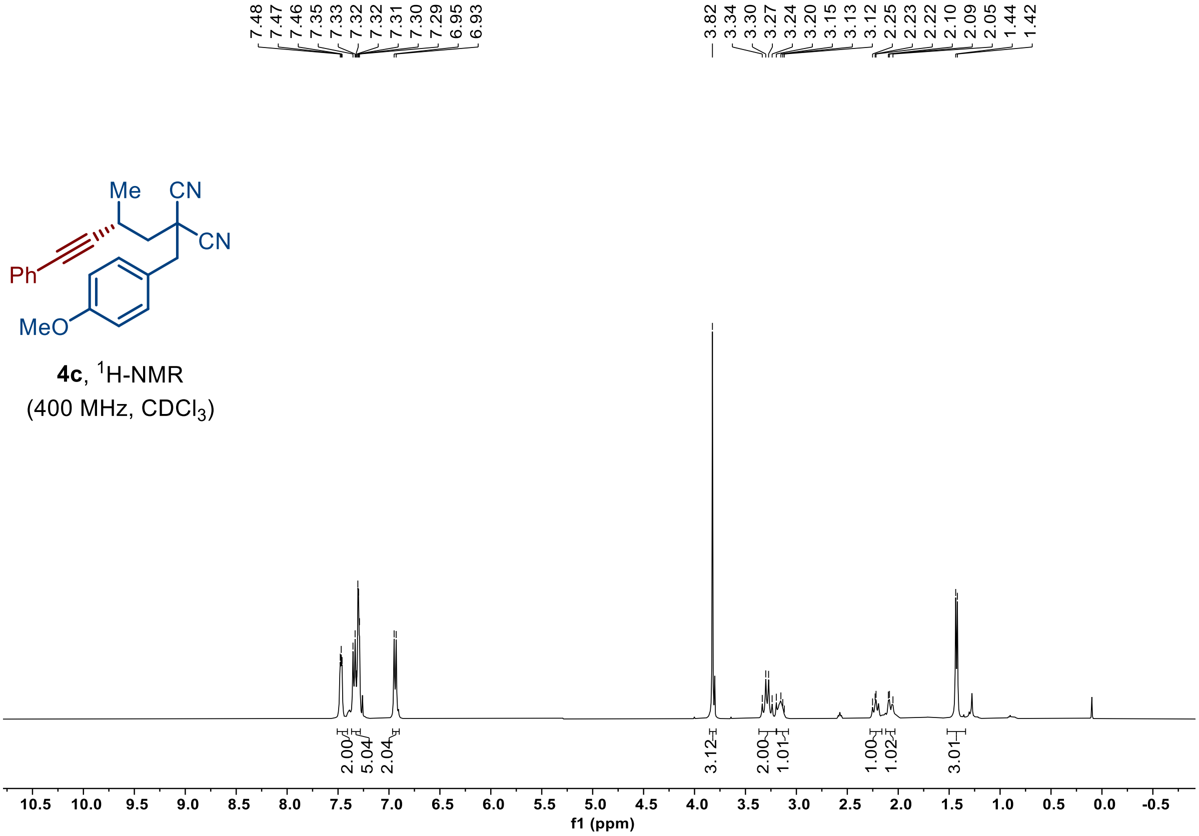

^{13}C NMR (100 MHz, $CDCl_3$) spectrum of 4c

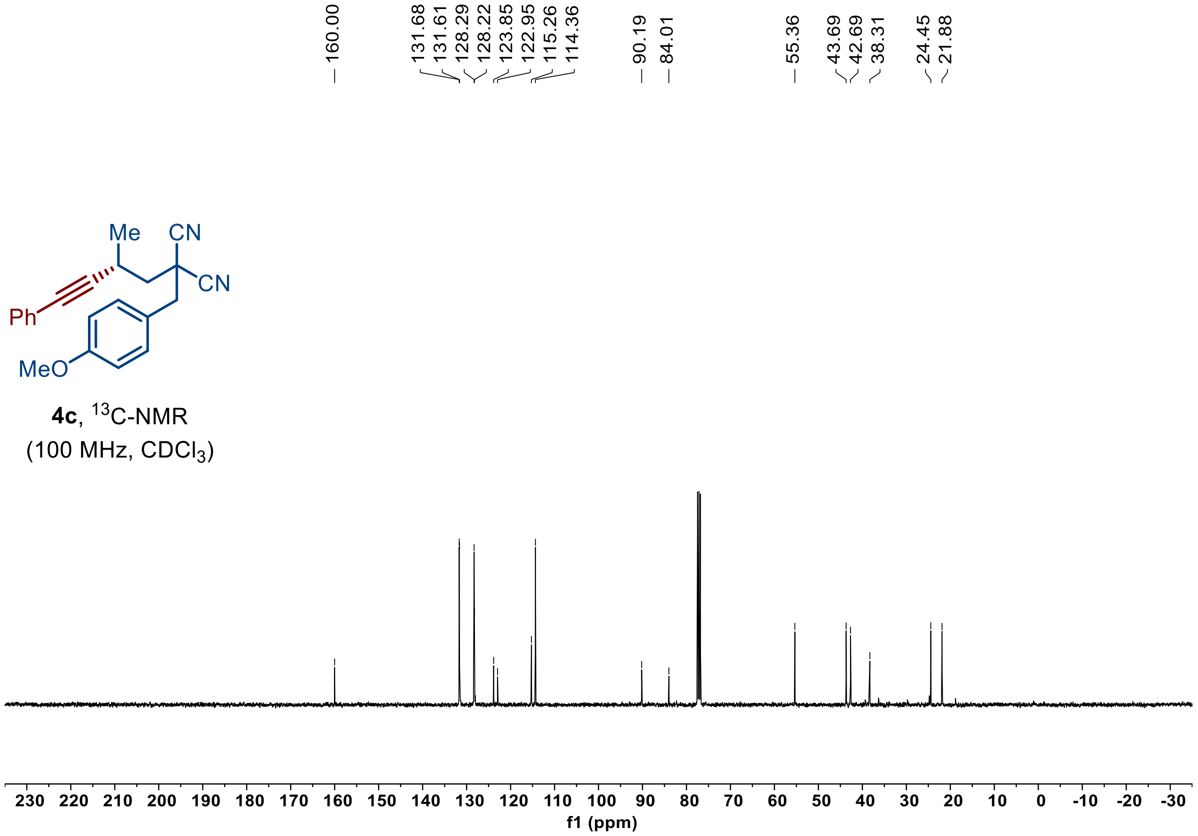

^{1}H NMR (400 MHz, $CDCl_3$) spectrum of 4d

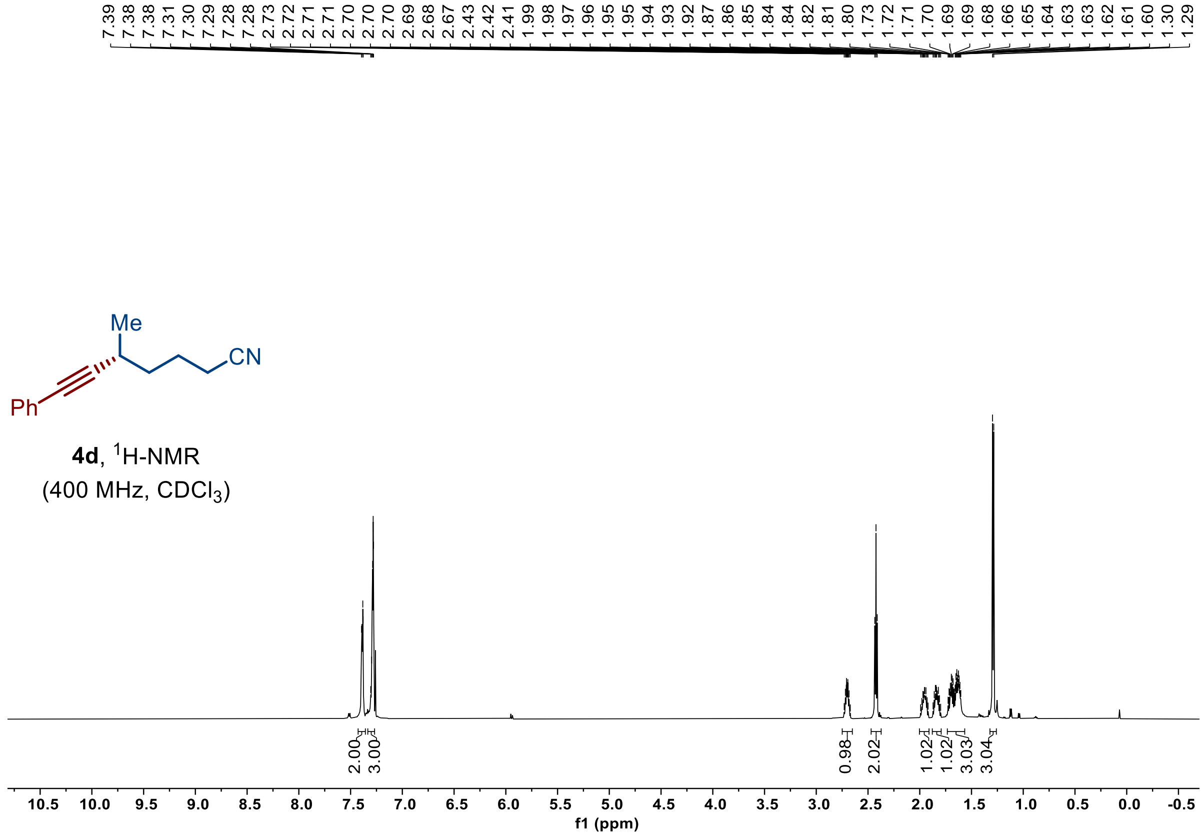

^{13}C NMR (100 MHz, $CDCl_3$) spectrum of 4d

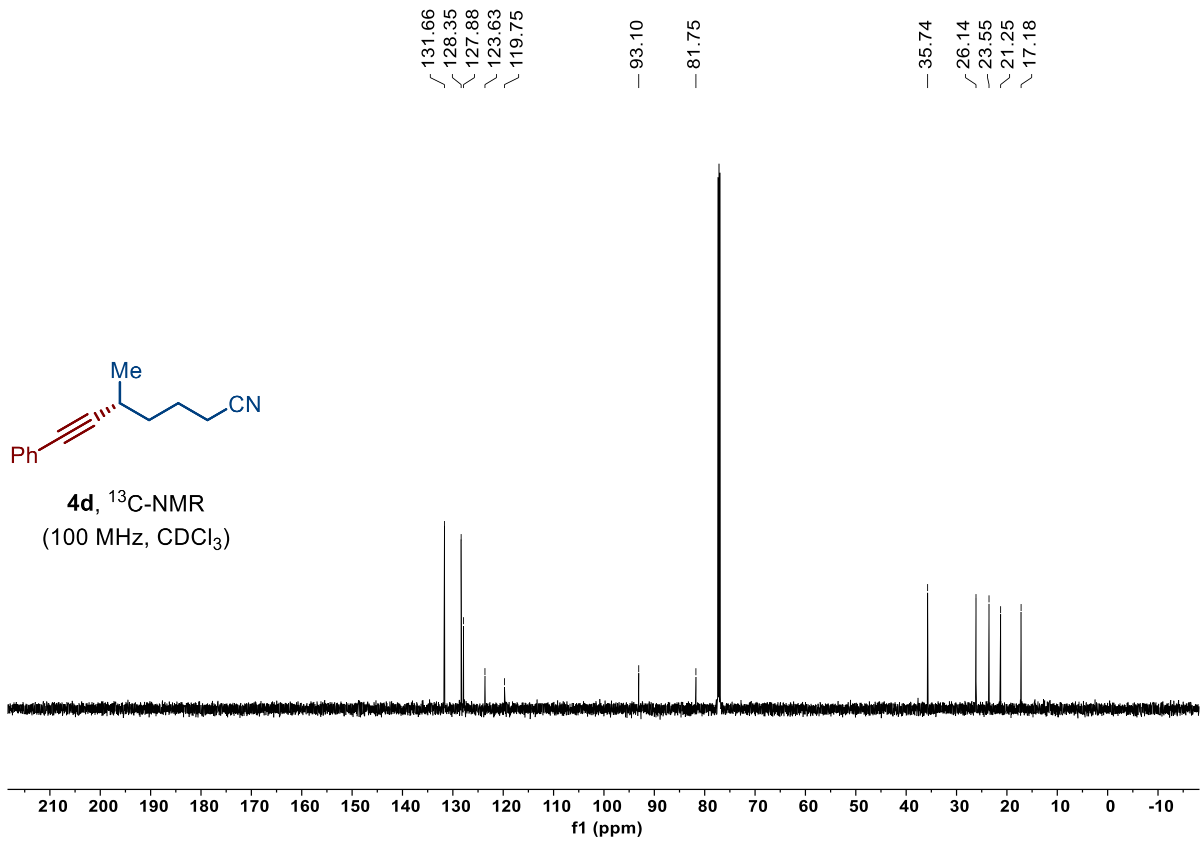

^{1}H NMR (600 MHz, $CDCl_3$) spectrum of 4e

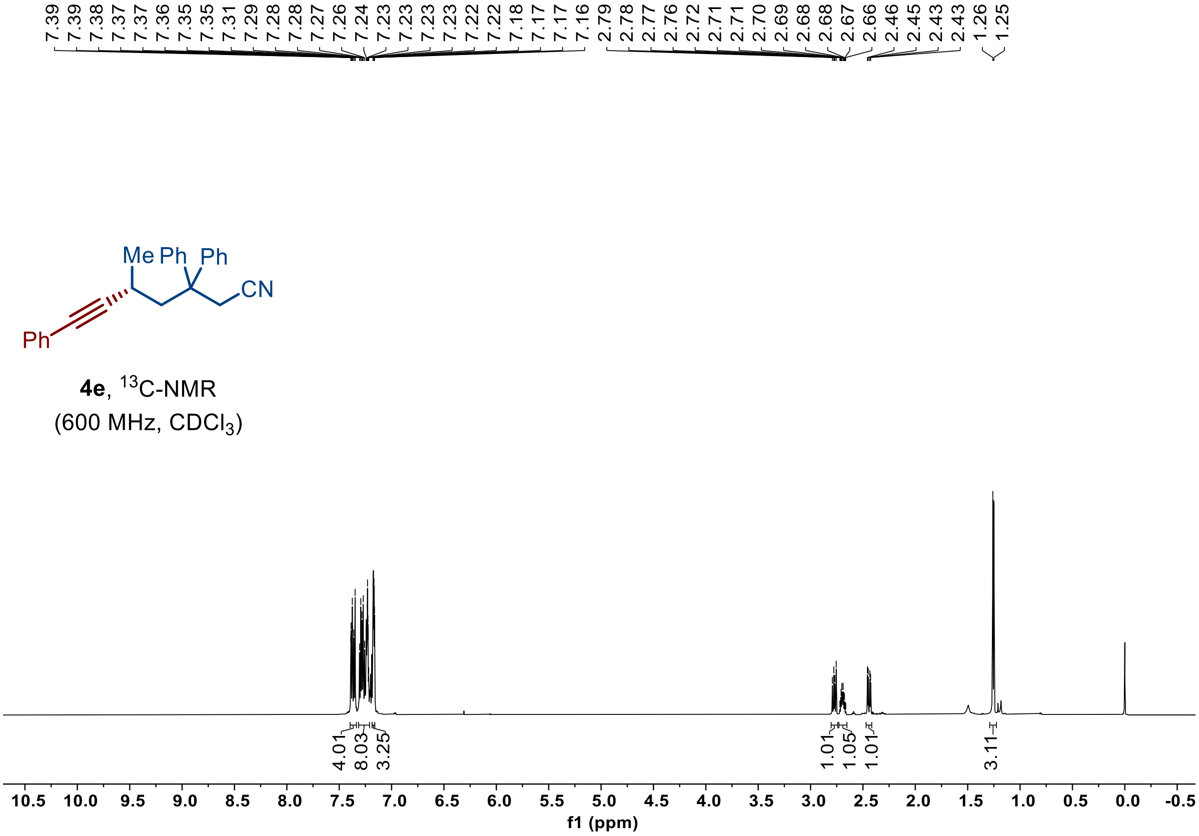

^{13}C NMR (150 MHz, $CDCl_3$) spectrum of 4e

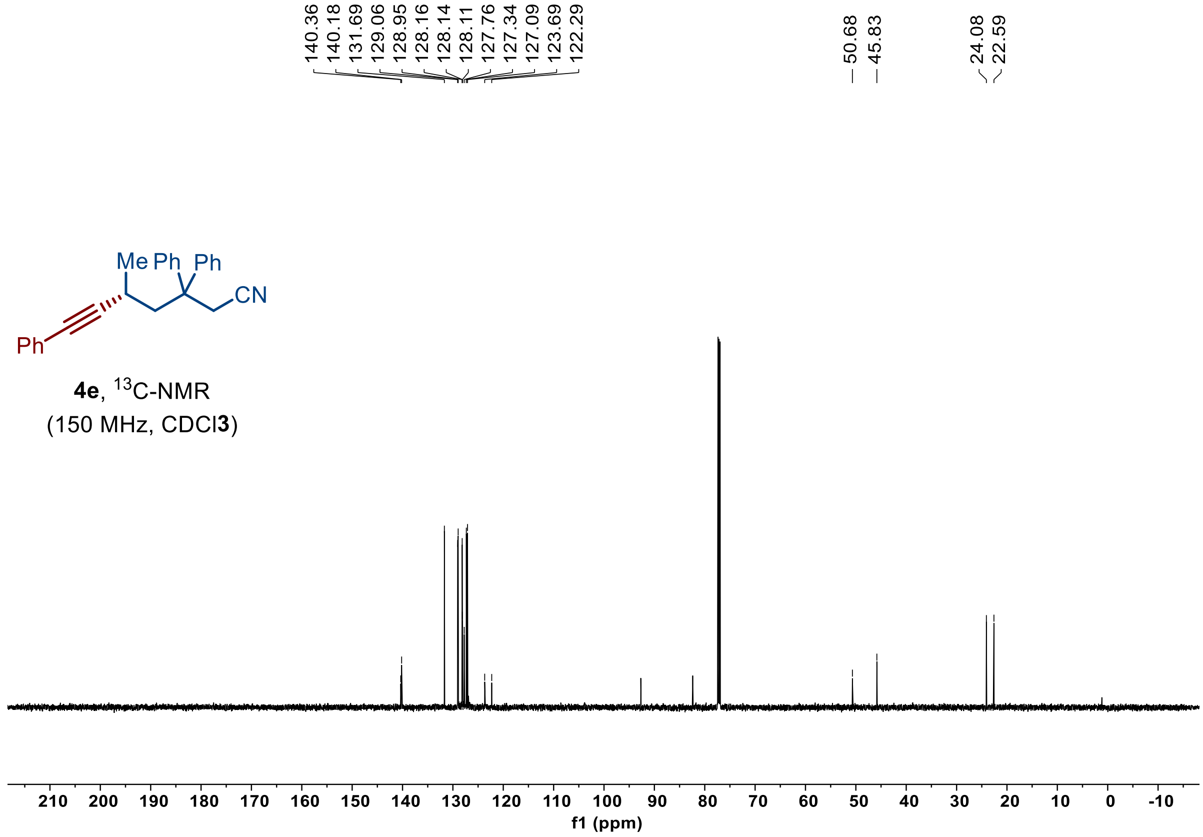

^{1}H NMR (400 MHz, $CDCl_3$) spectrum of 4f

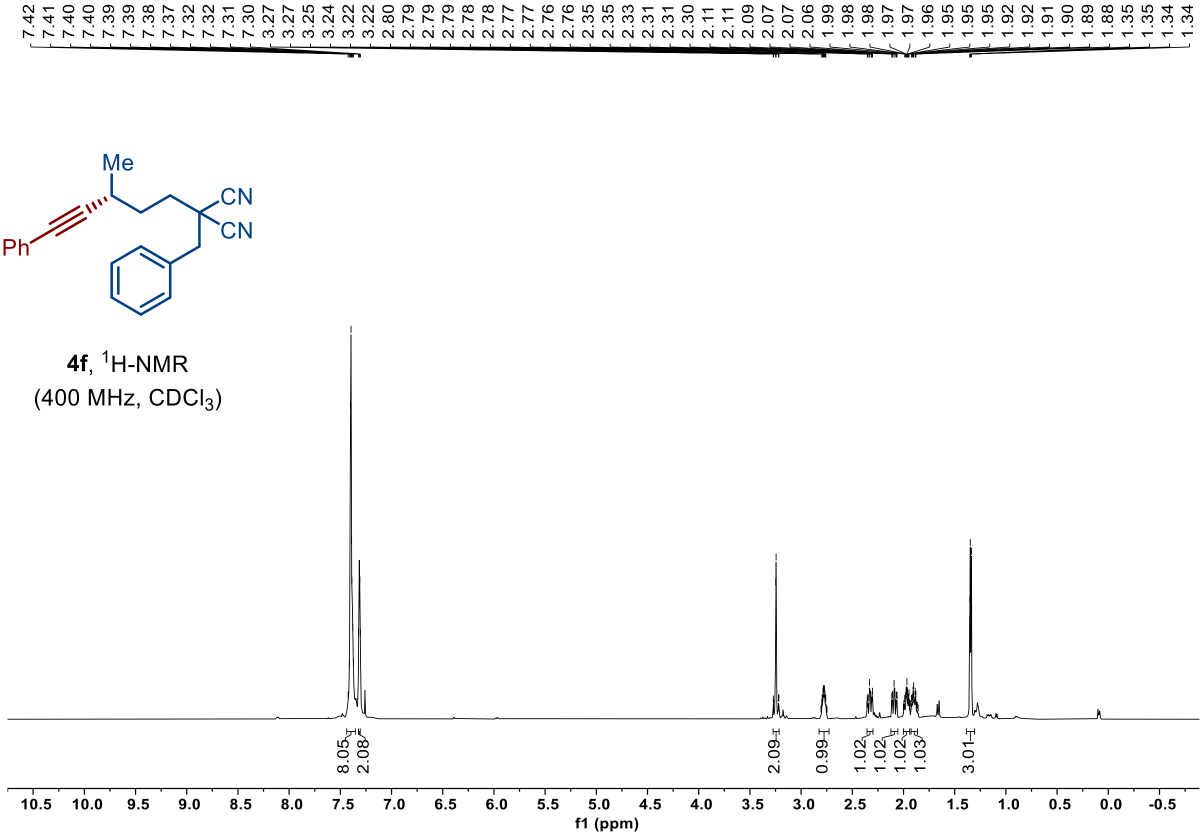

^{13}C NMR (100 MHz, $CDCl_3$) spectrum of 4f

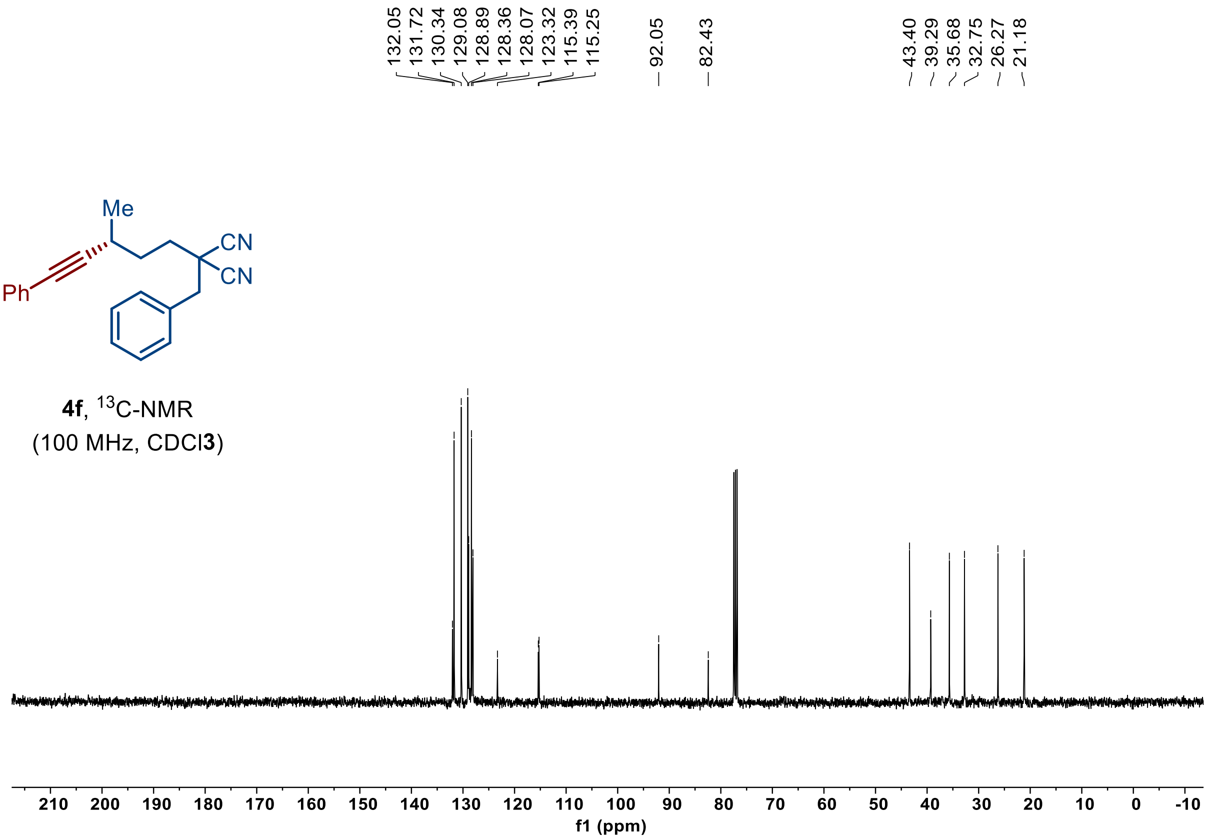

^{1}H NMR (400 MHz, CDCl$_3$) spectrum of 4g

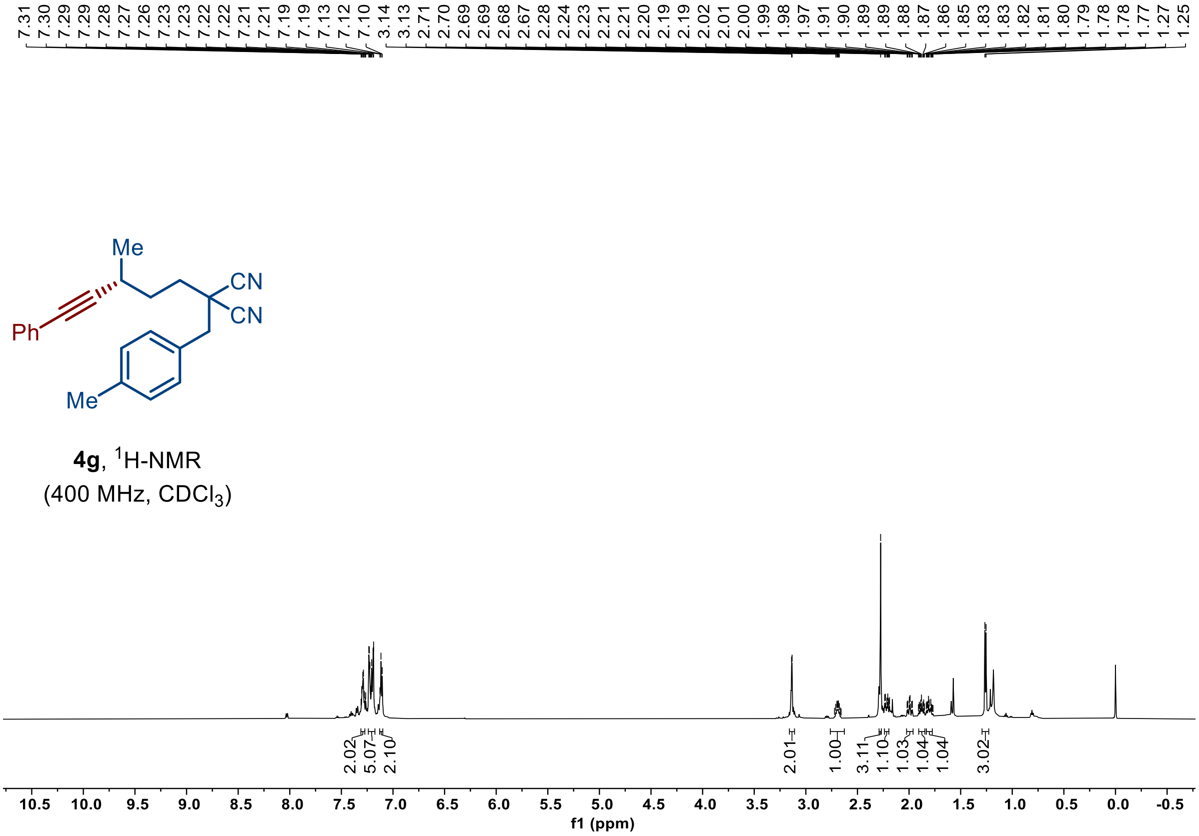

^{13}C NMR (100 MHz, CDCl$_3$) spectrum of 4g

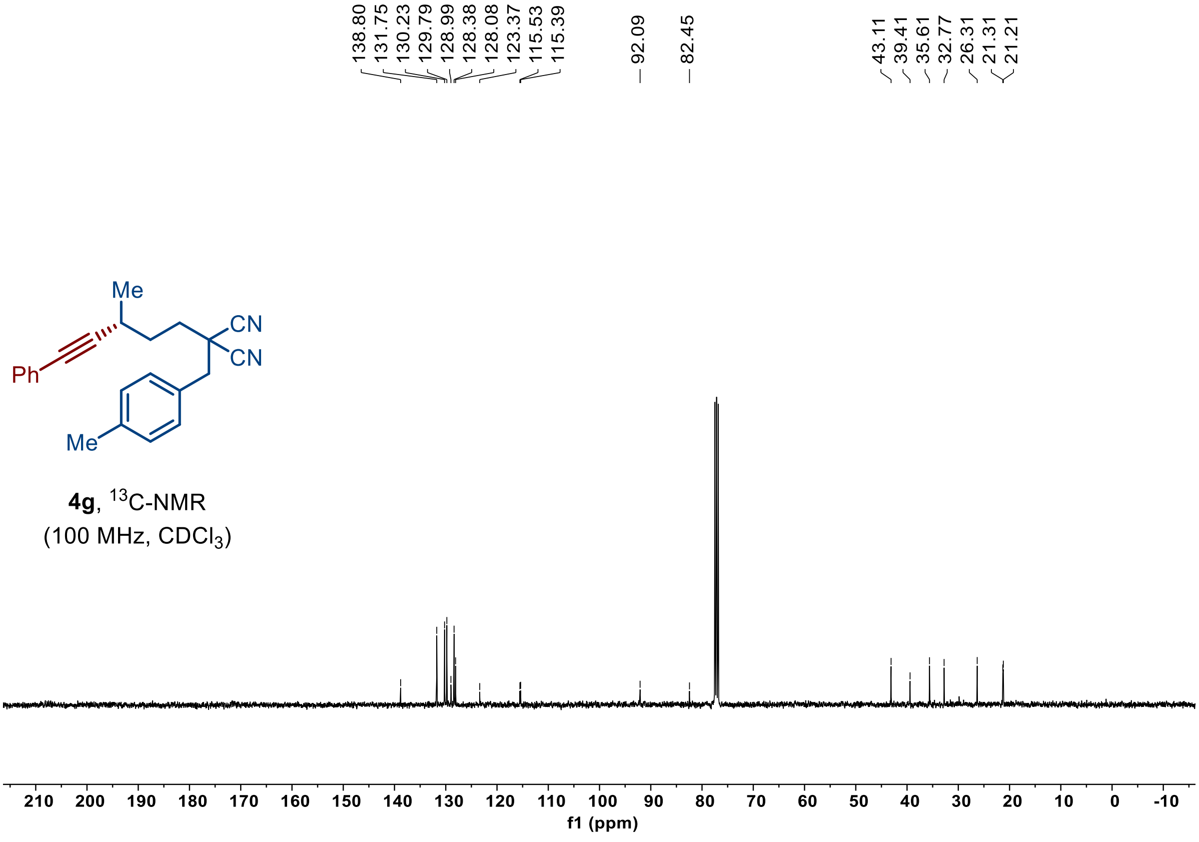

^{1}H NMR (400 MHz, $CDCl_3$) spectrum of 4h

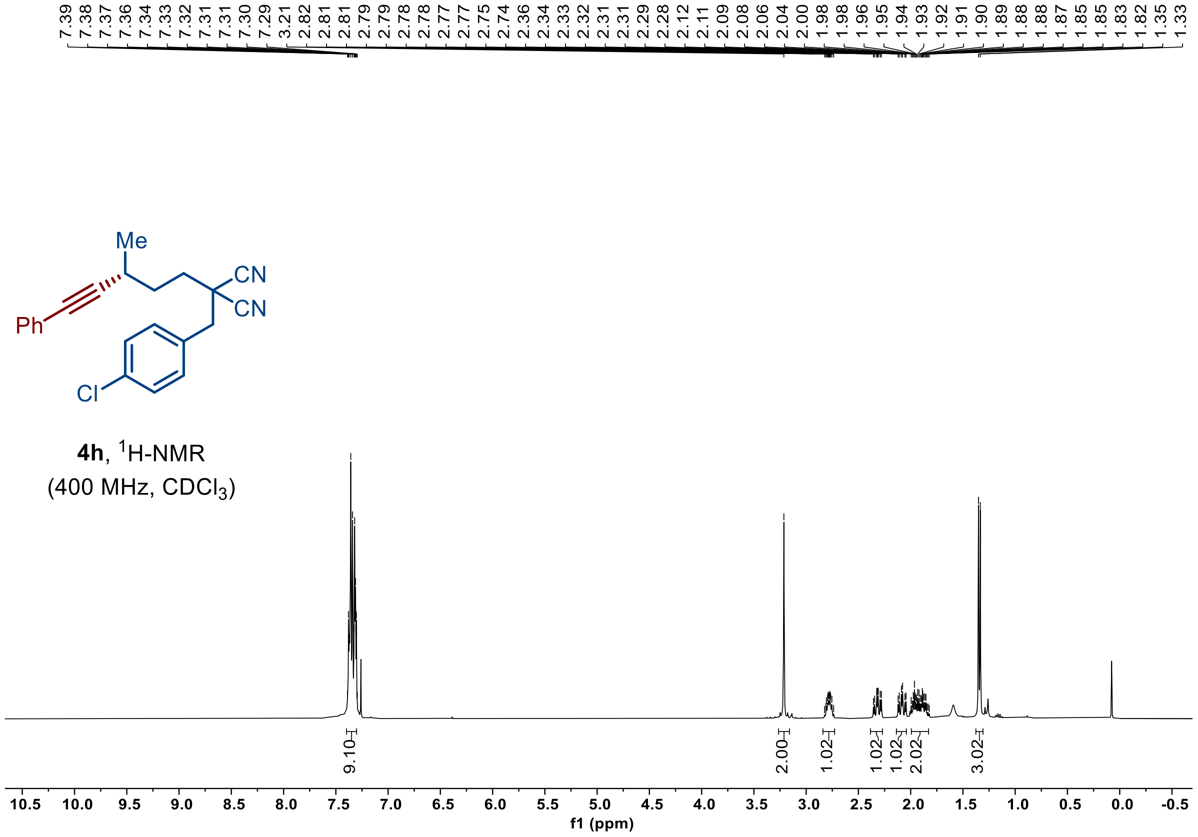

^{13}C NMR (100 MHz, $CDCl_3$) spectrum of 4h

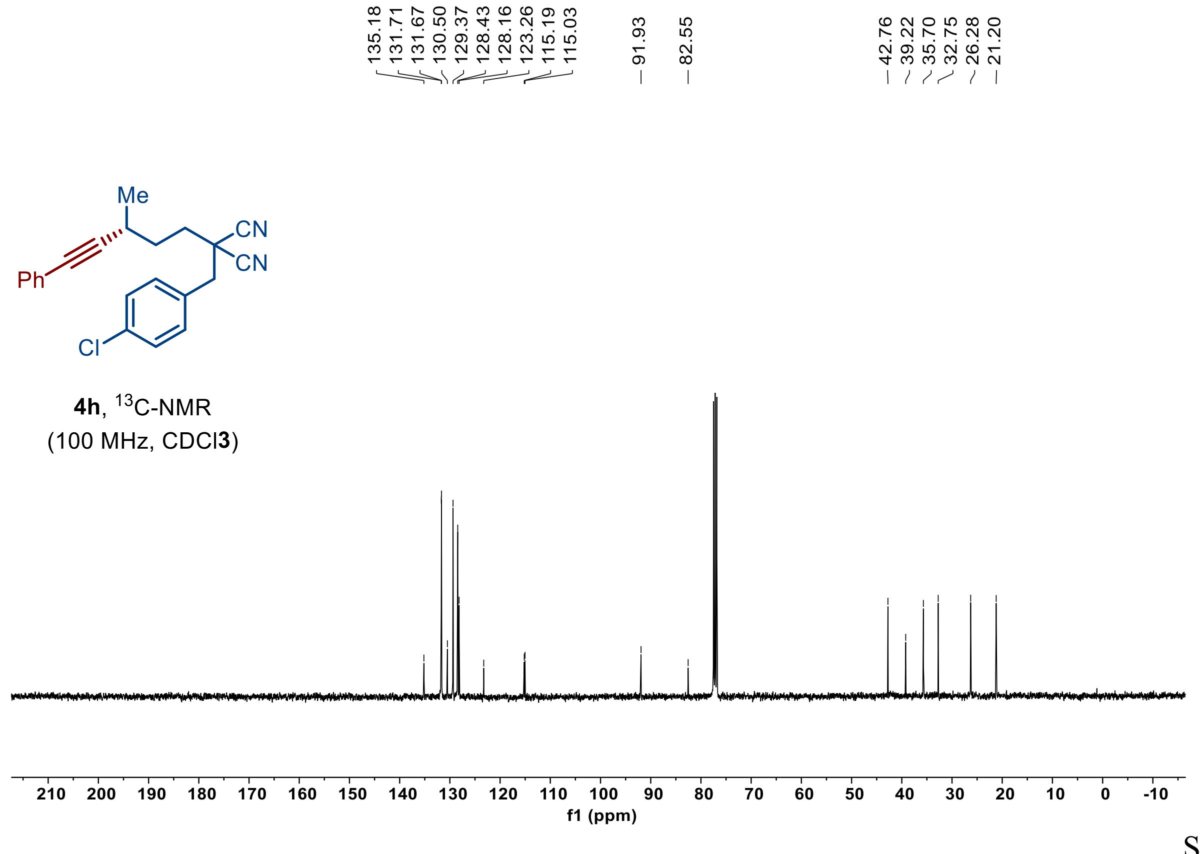

^{1}H NMR (400 MHz, CDCl$_3$) spectrum of 4i

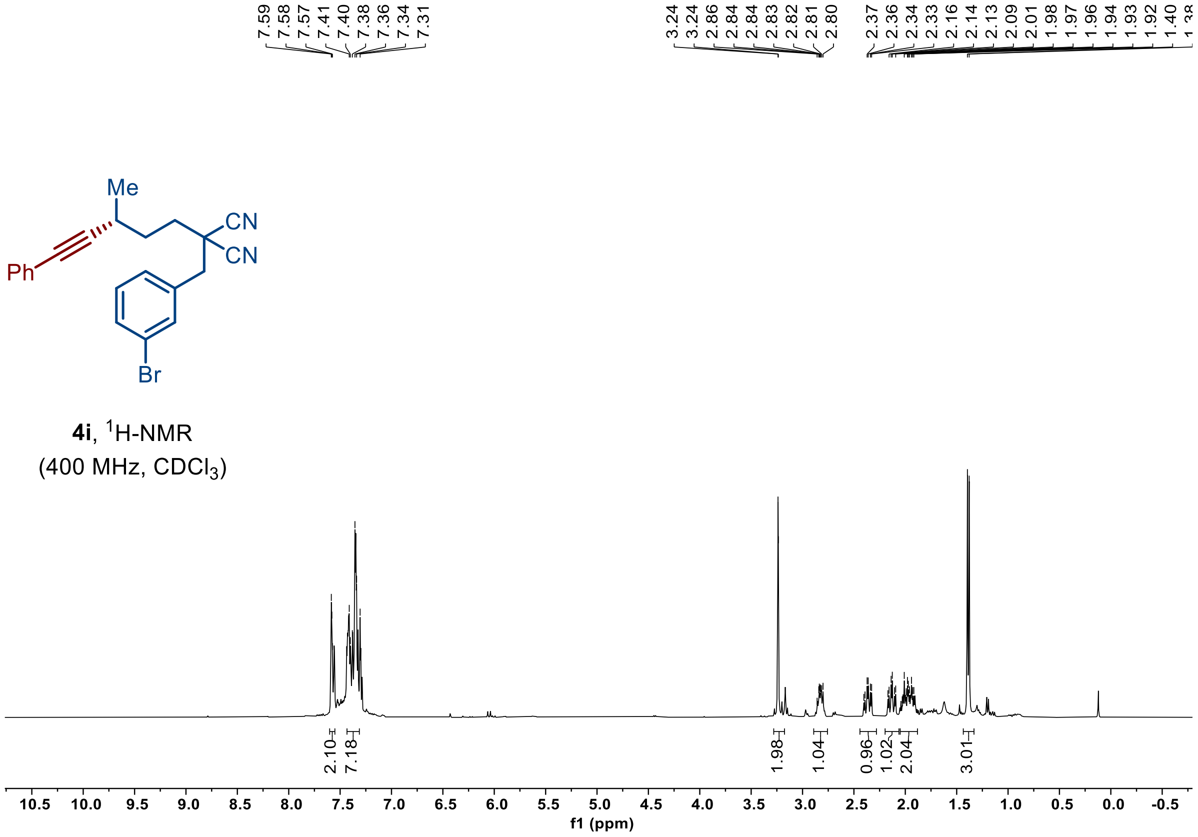

^{13}C NMR (100 MHz, CDCl$_3$) spectrum of 4i

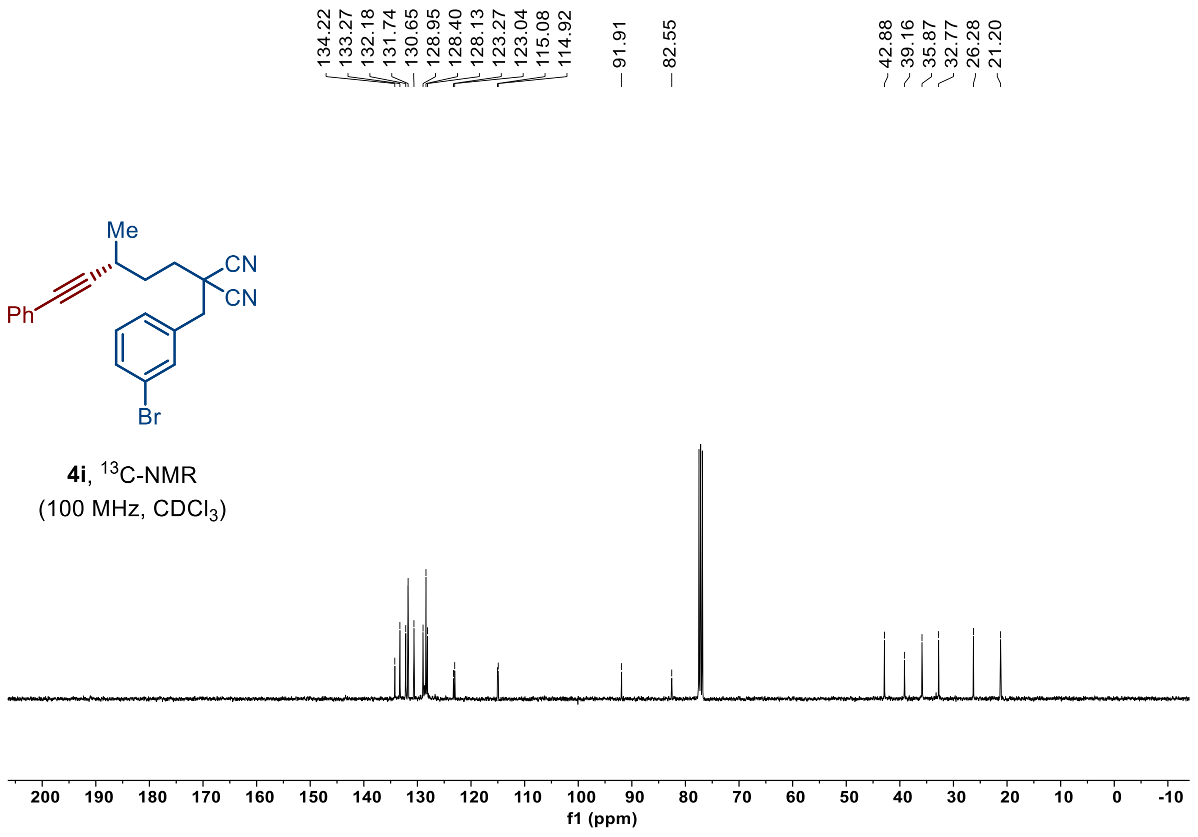

^{1}H NMR (400 MHz, $CDCl_3$) spectrum of 4j

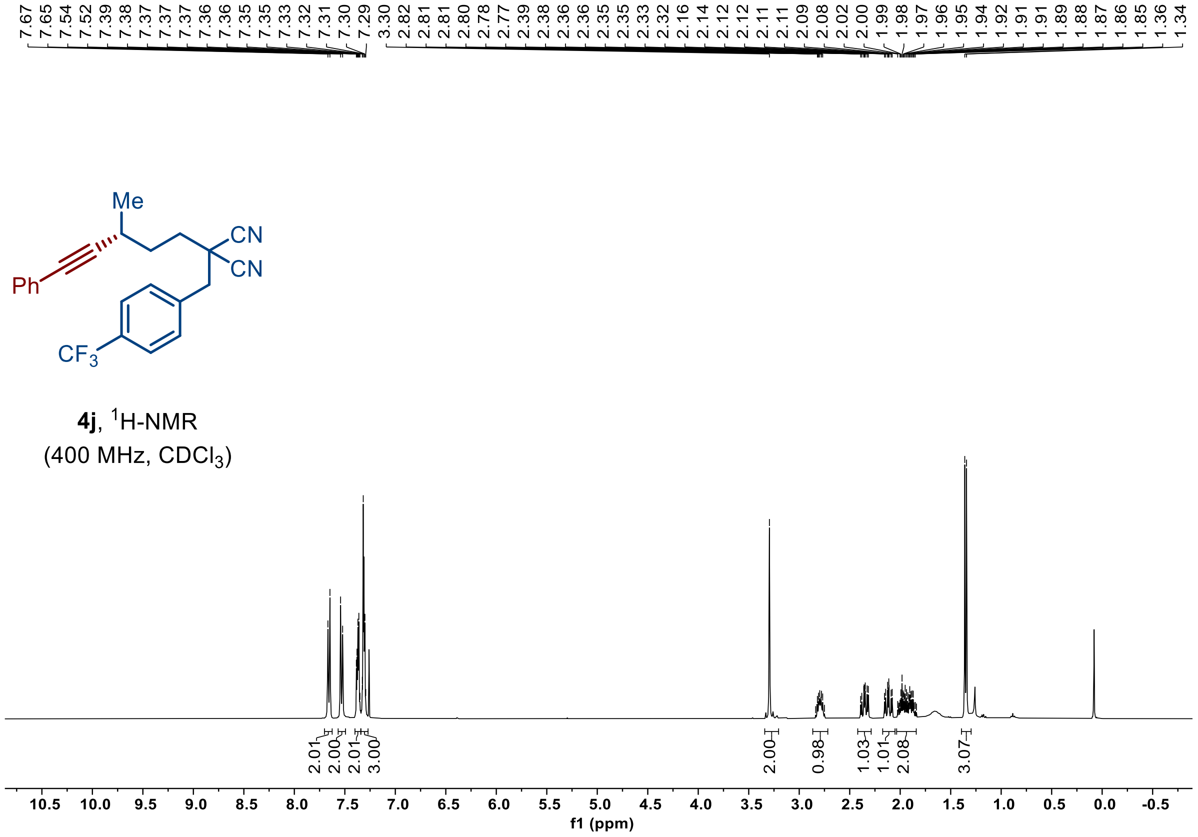

^{13}C NMR (100 MHz, $CDCl_3$) spectrum of 4j

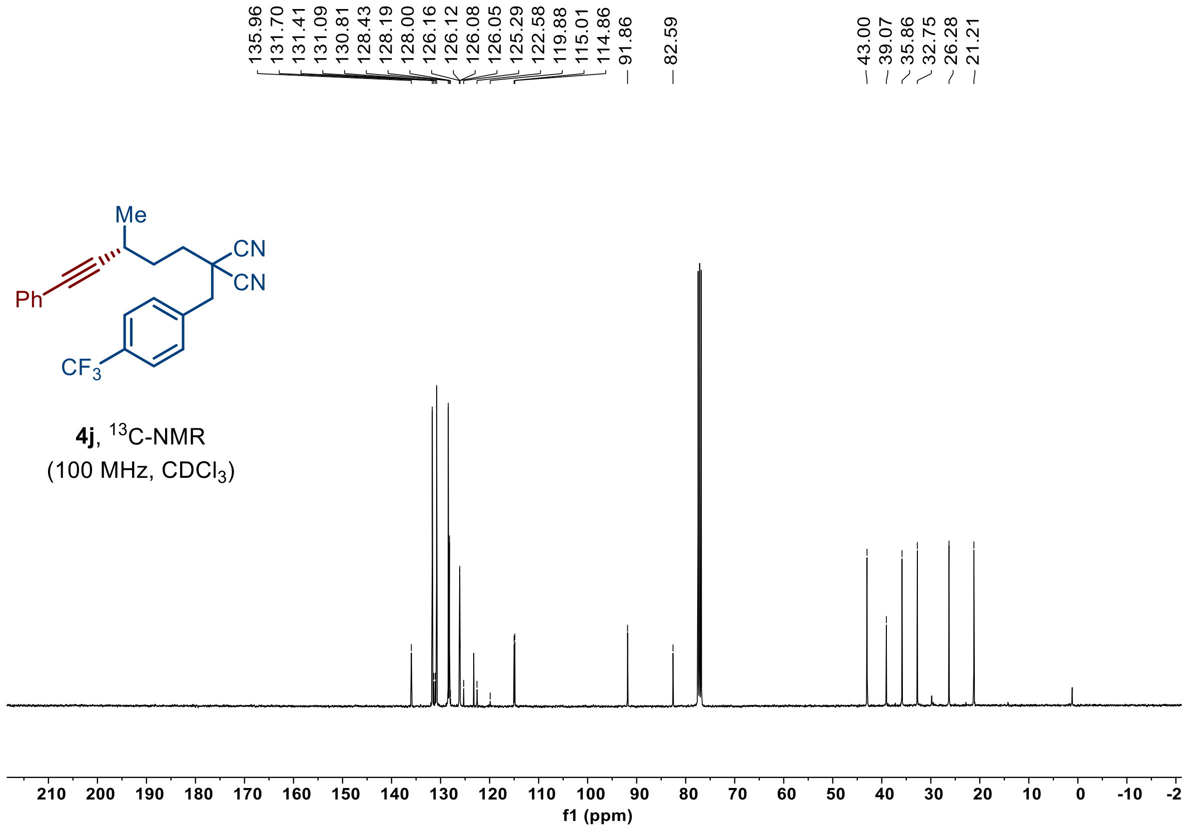

^{19}F NMR (376 MHz, $CDCl_3$) spectrum of 4j

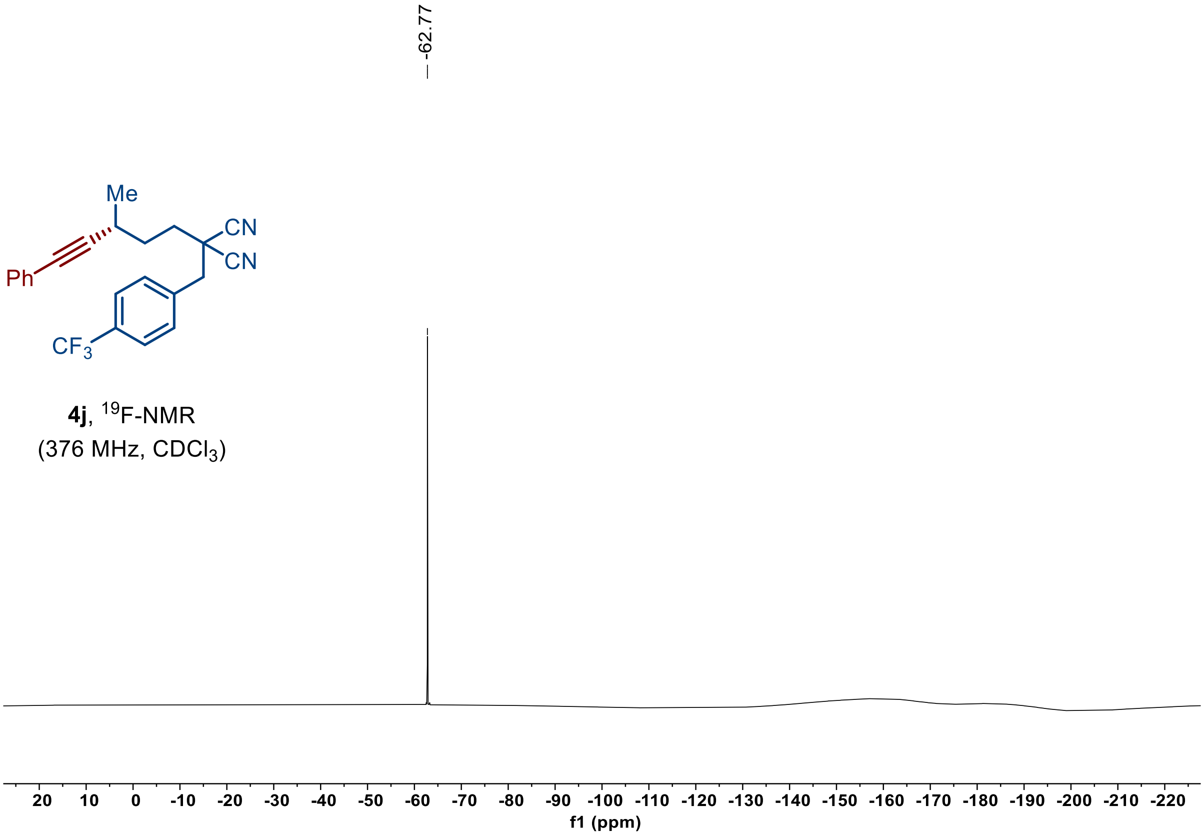

^{1}H NMR (400 MHz, CDCl₃) spectrum of 4k

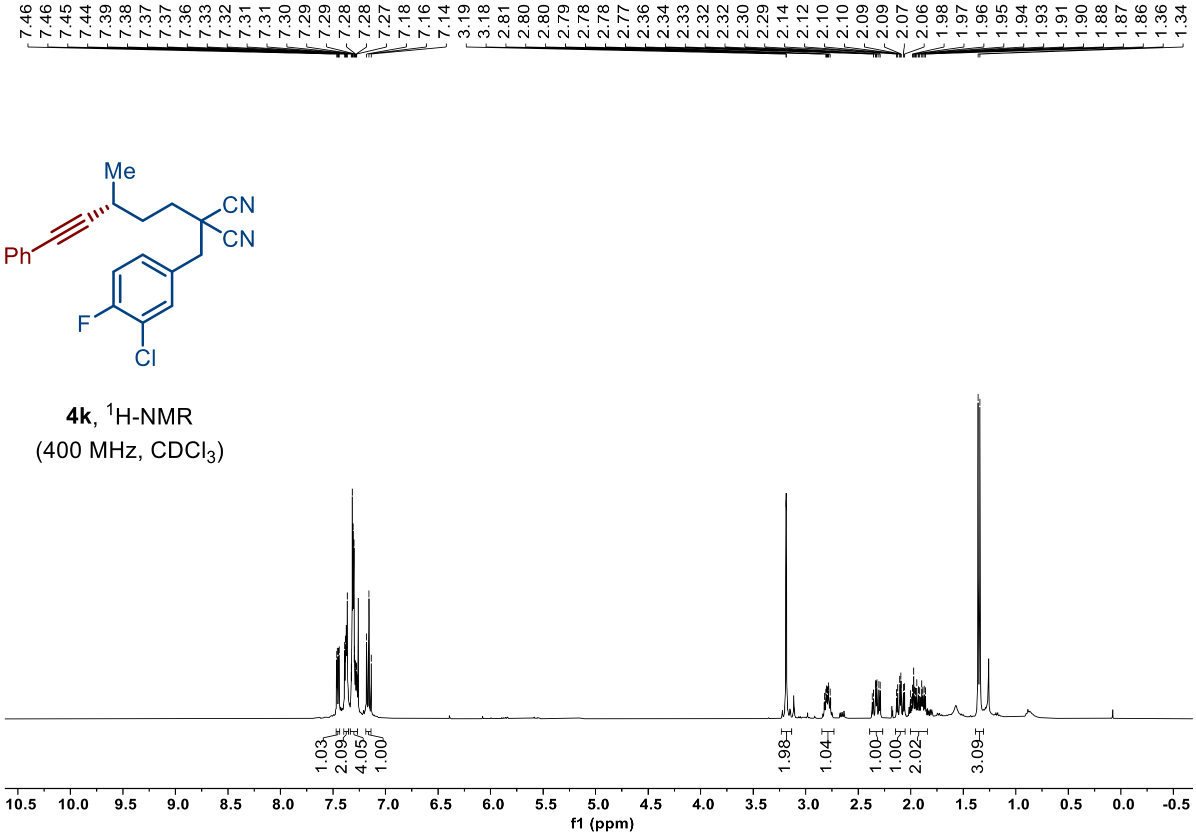

^{13}C NMR (100 MHz, CDCl₃) spectrum of 4k

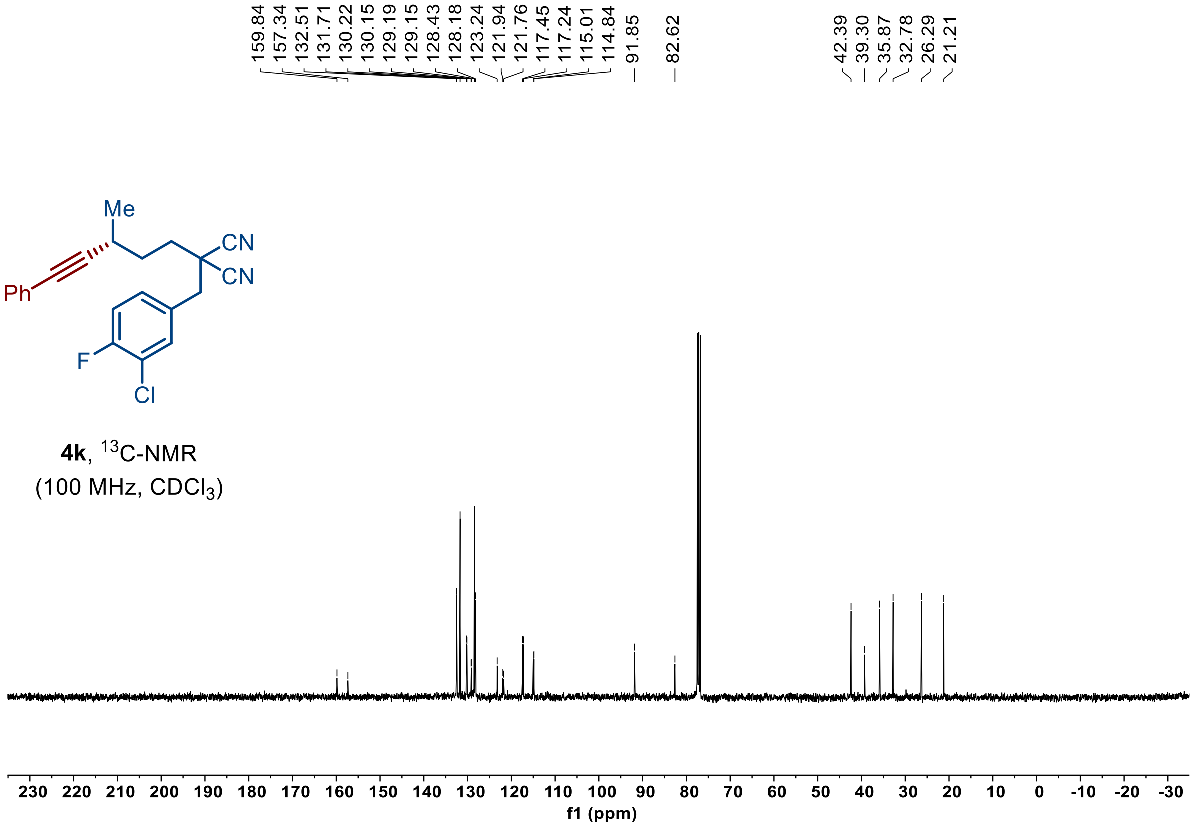

^{19}F NMR (376 MHz, CDCl$_3$) spectrum of 4k

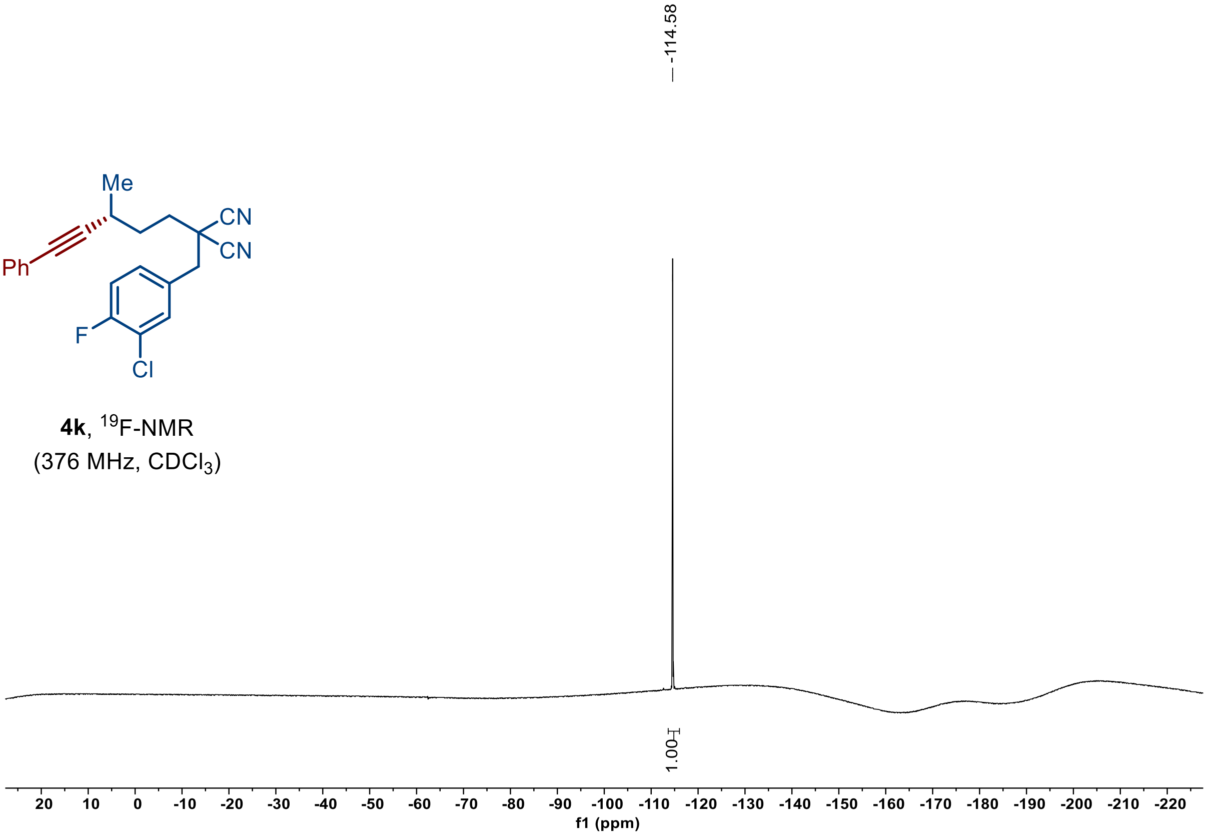

^{1}H NMR (400 MHz, CDCl$_3$) spectrum of 4l

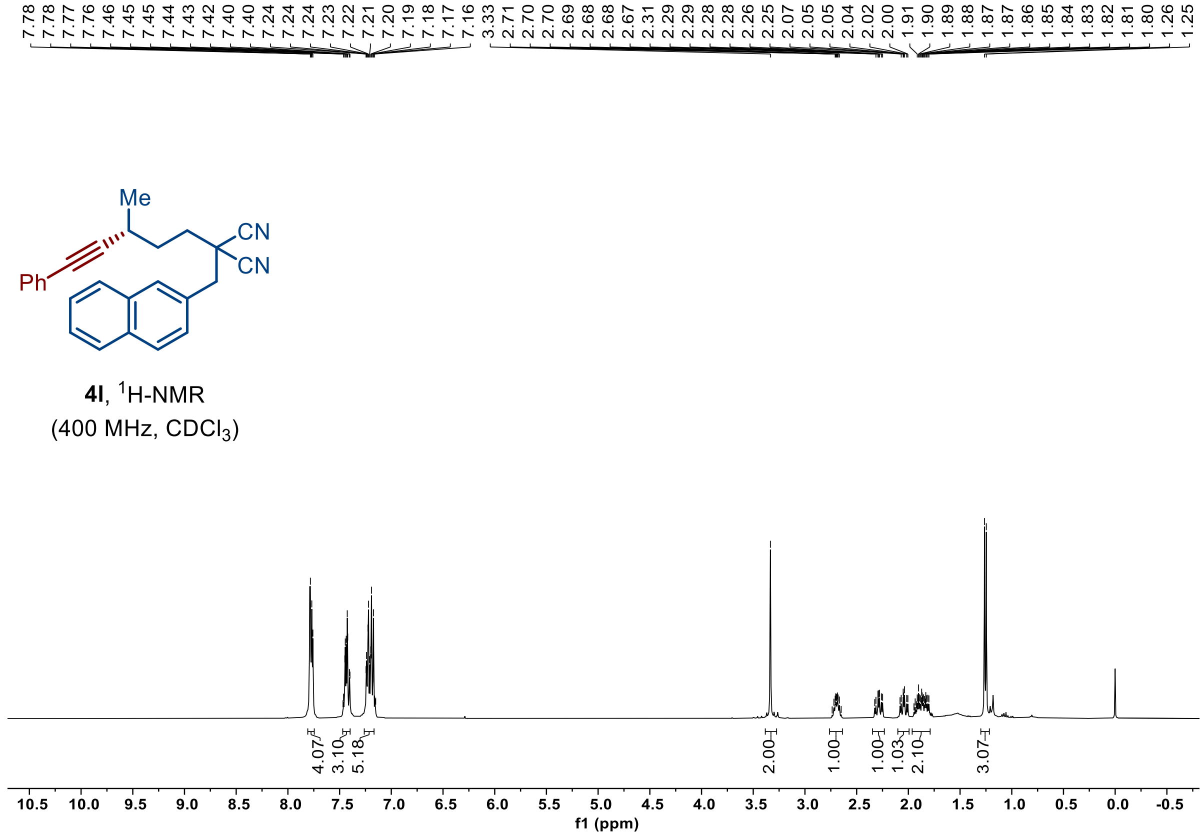

^{13}C NMR (100 MHz, CDCl$_3$) spectrum of 4l

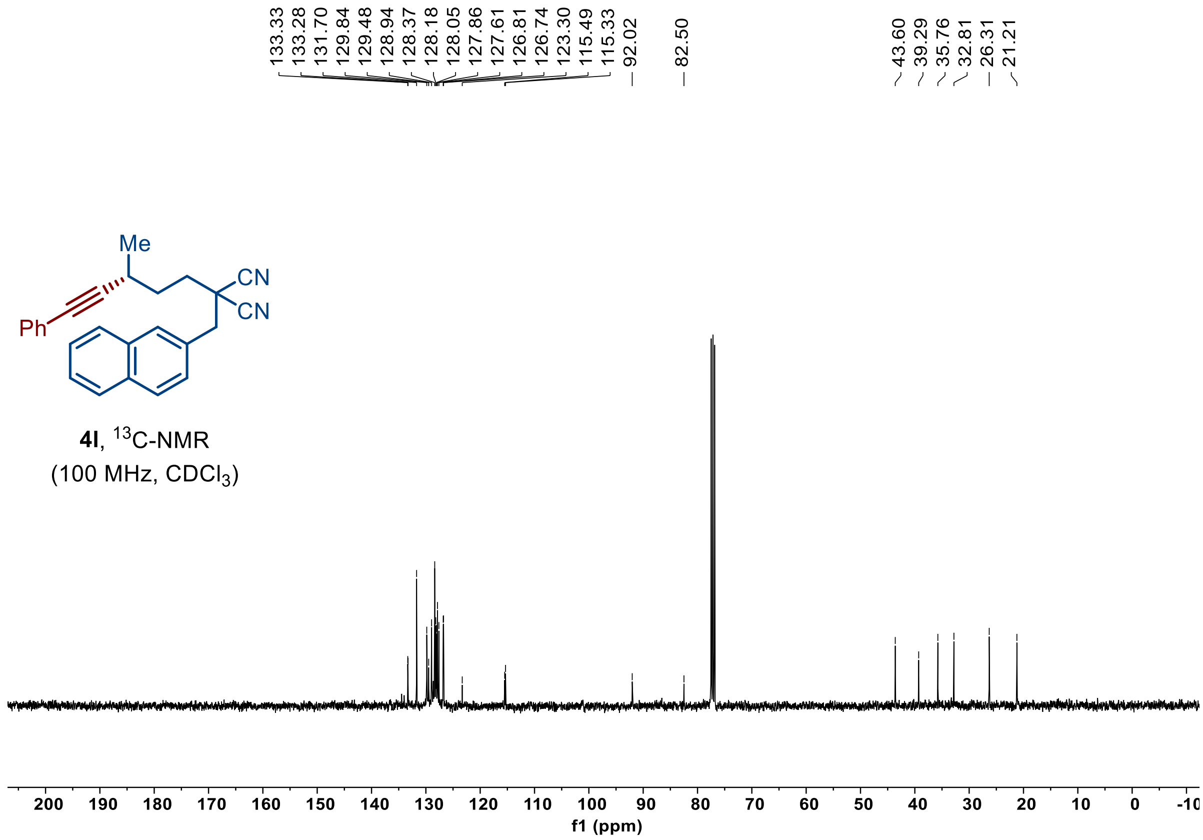

^{1}H NMR (400 MHz, CDCl3) spectrum of 4m

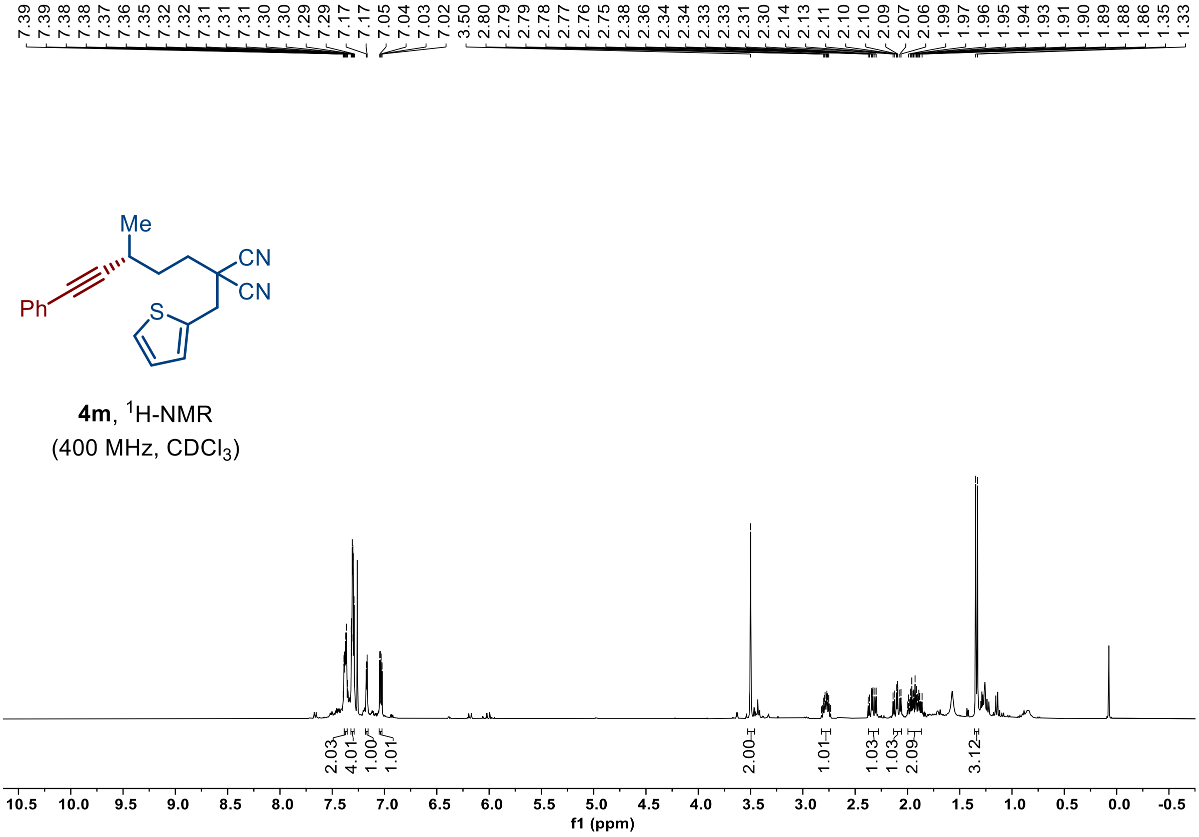

^{13}C NMR (100 MHz, CDCl3) spectrum of 4m

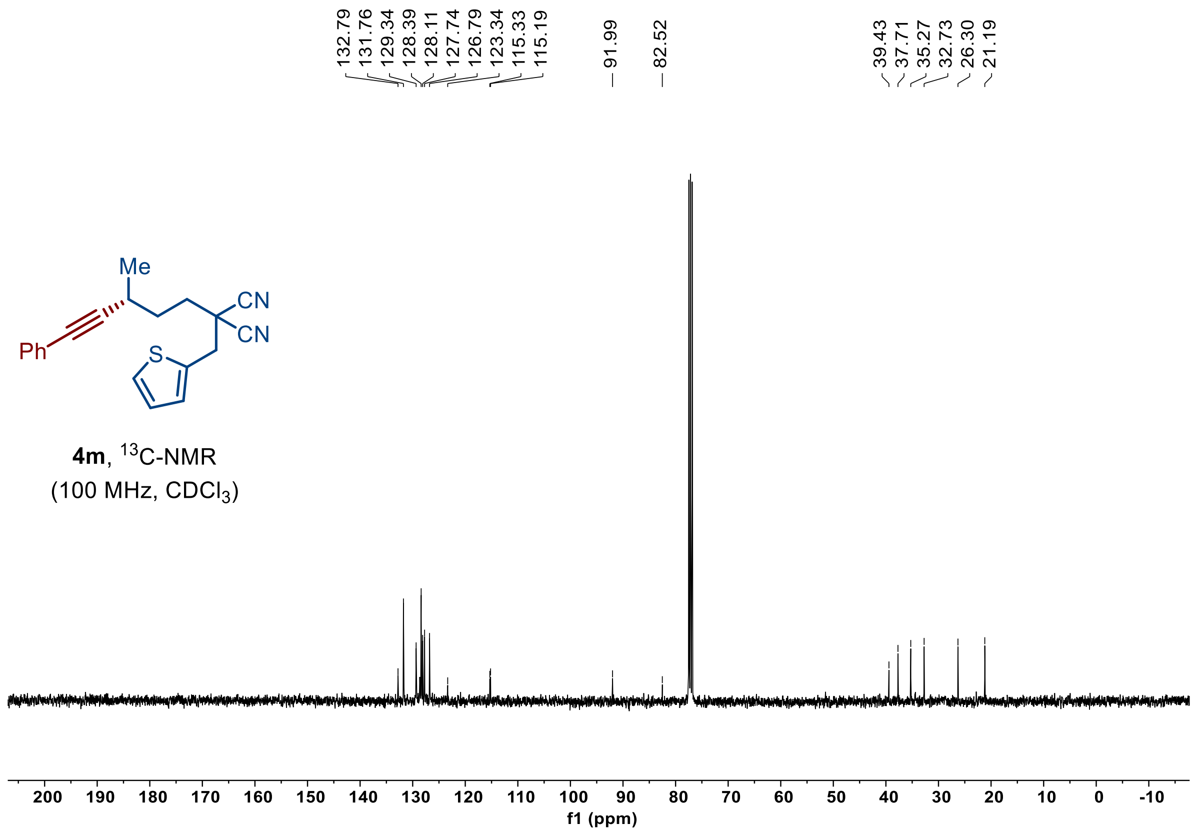

^{1}H NMR (400 MHz, $CDCl_3$) spectrum of 4n

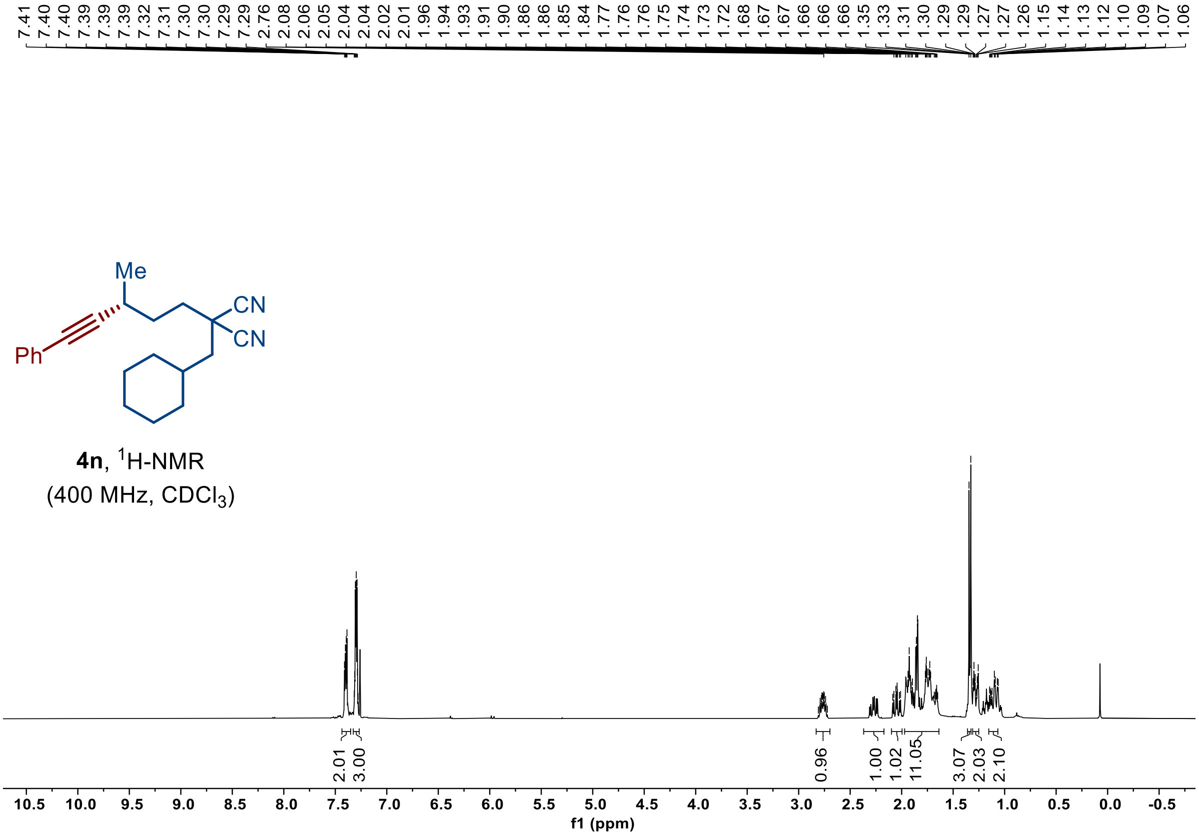

^{13}C NMR (100 MHz, $CDCl_3$) spectrum of 4n

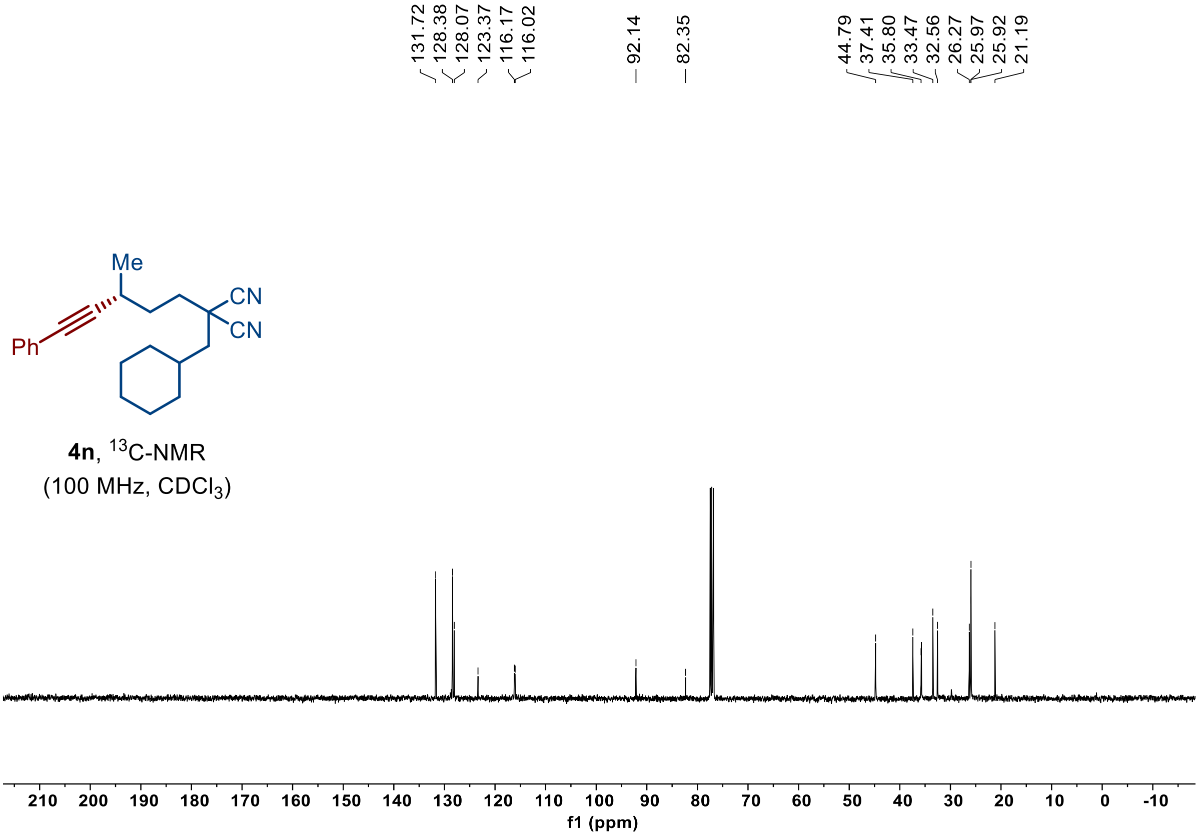

^{1}H NMR (400 MHz, CDCl3) spectrum of 4o

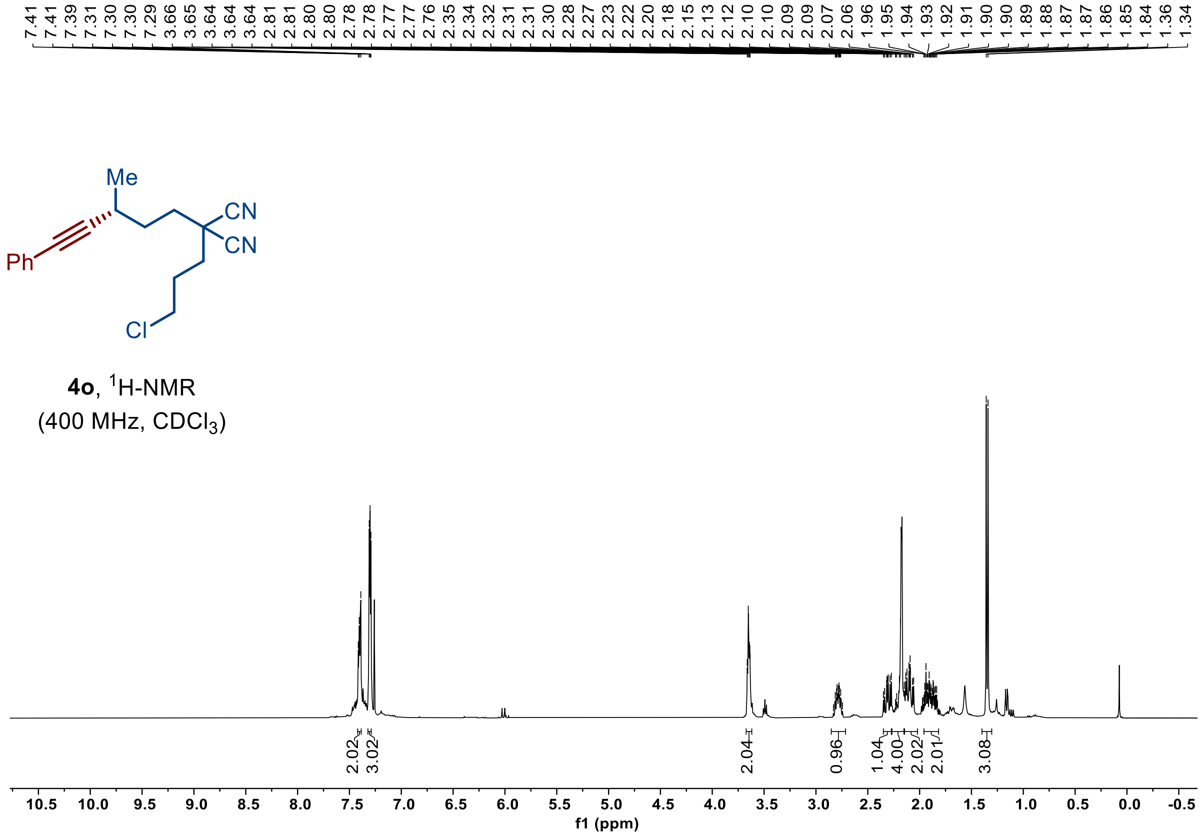

^{13}C NMR (100 MHz, CDCl3) spectrum of 4o

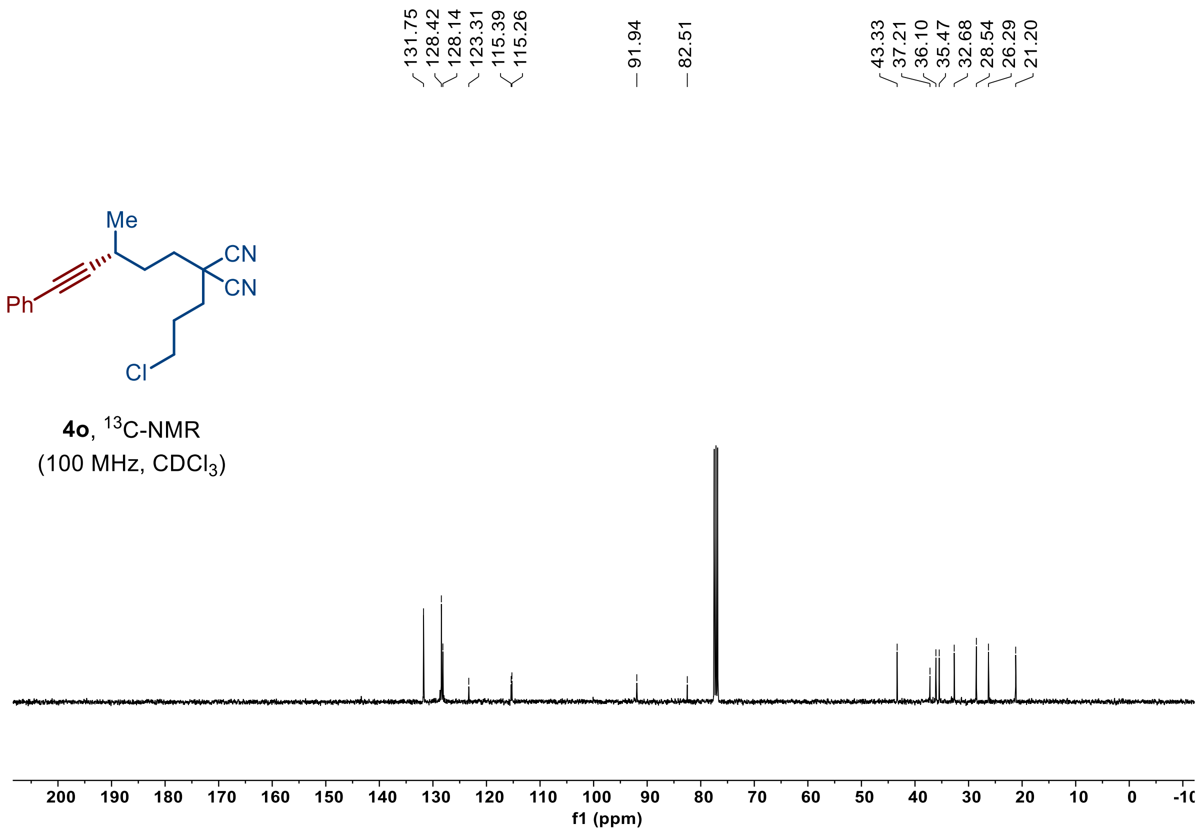

^{1}H NMR (600 MHz, $CDCl_3$) spectrum of 4p

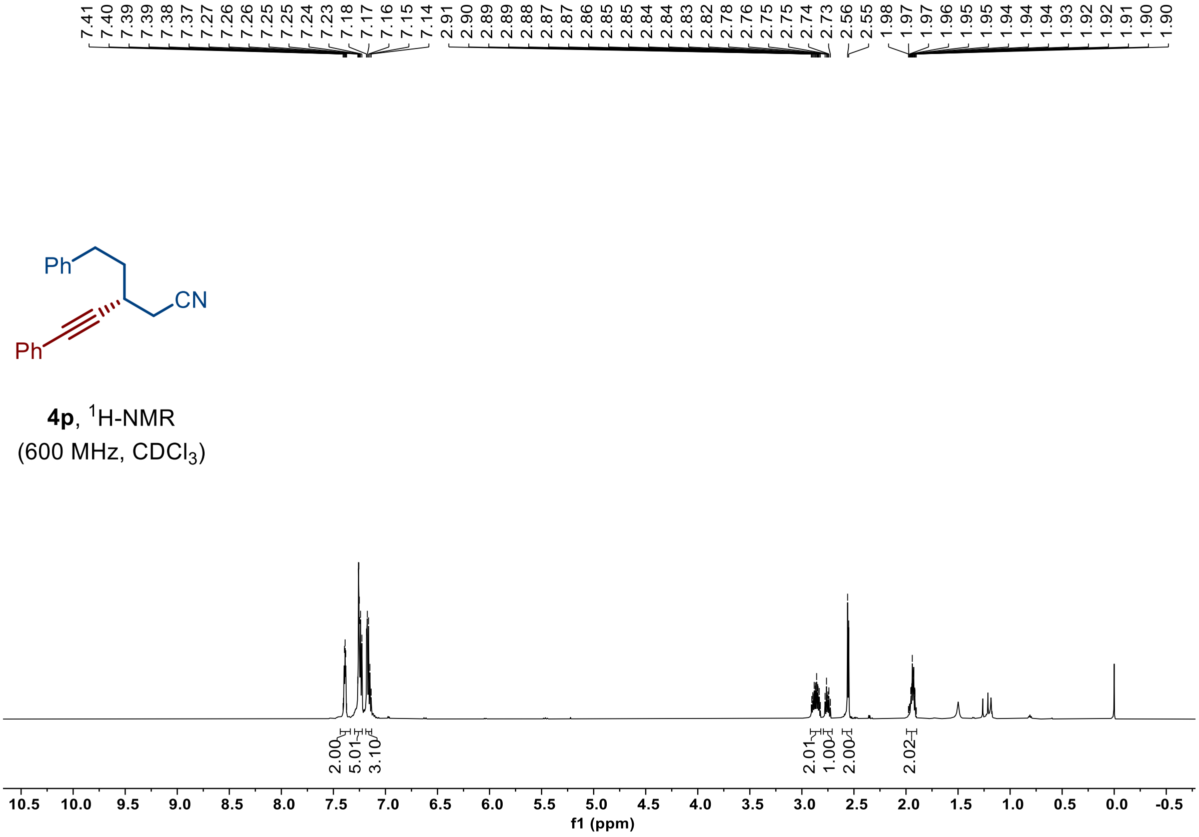

^{13}C NMR (150 MHz, $CDCl_3$) spectrum of 4p

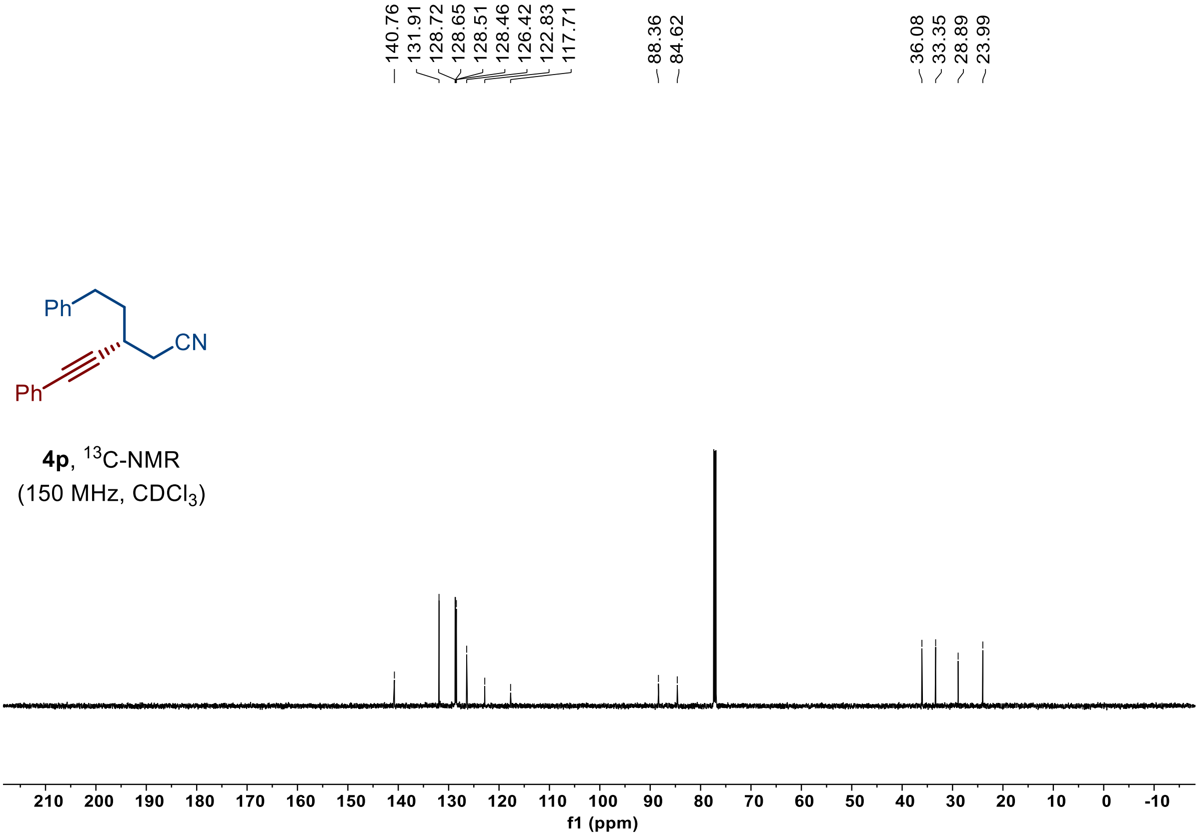

^{1}H NMR (600 MHz, $CDCl_3$) spectrum of 4q

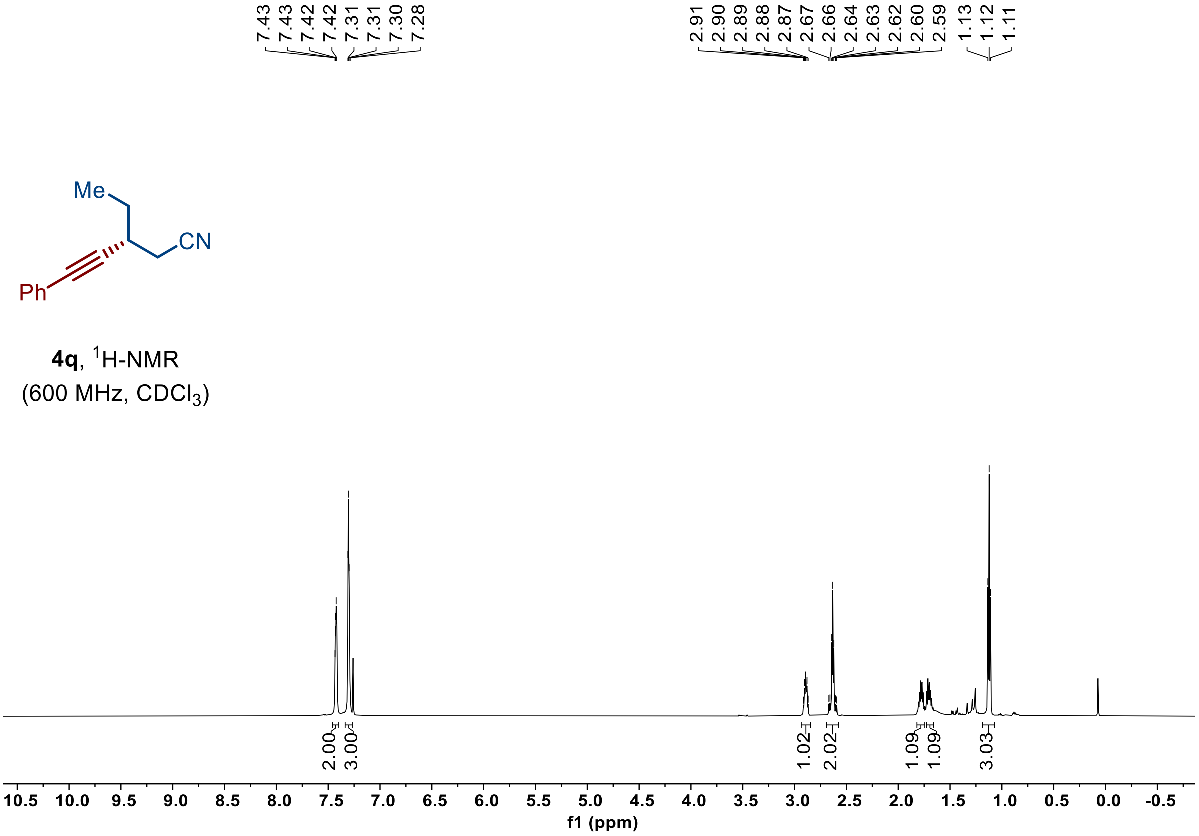

^{13}C NMR (150 MHz, $CDCl_3$) spectrum of 4q

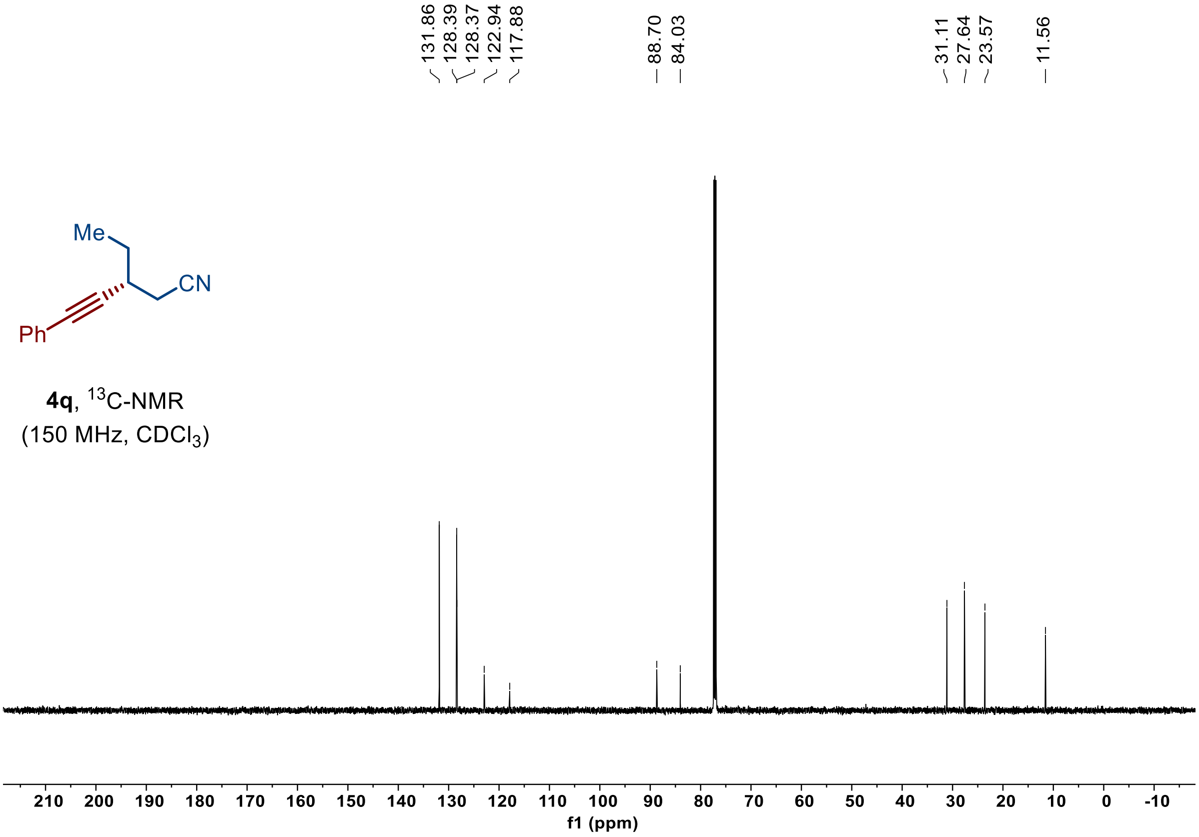

^{1}H NMR (600 MHz, $CDCl_3$) spectrum of 4w

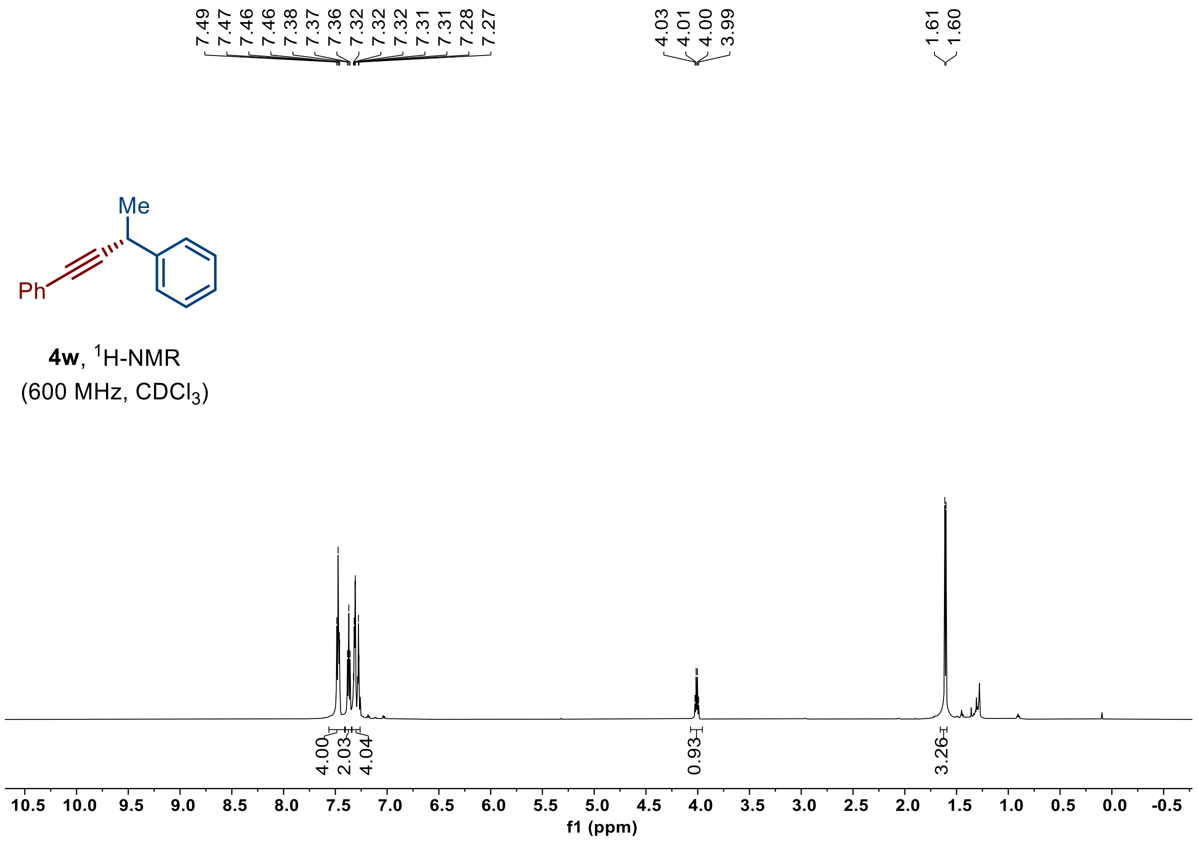

^{13}C NMR (150 MHz, $CDCl_3$) spectrum of 4w

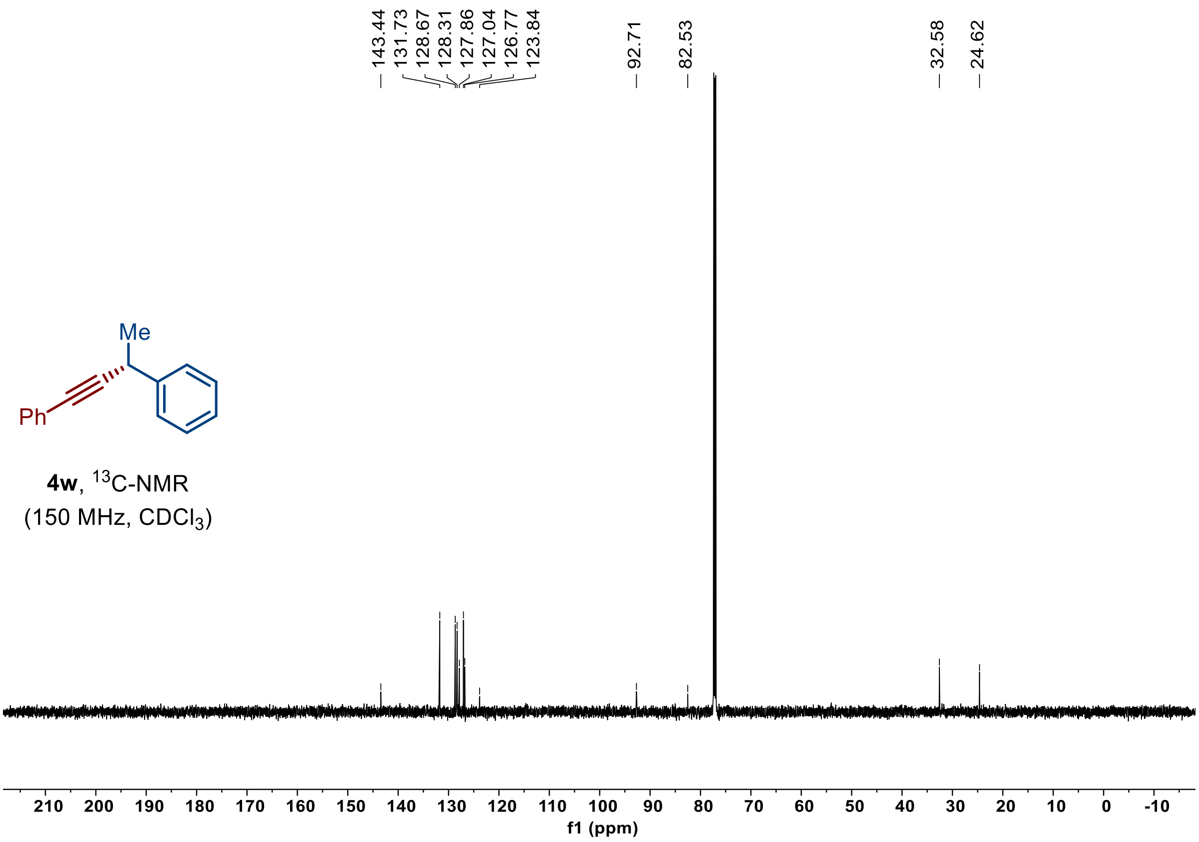

^{1}H NMR (400 MHz, $CDCl_3$) spectrum of 5a

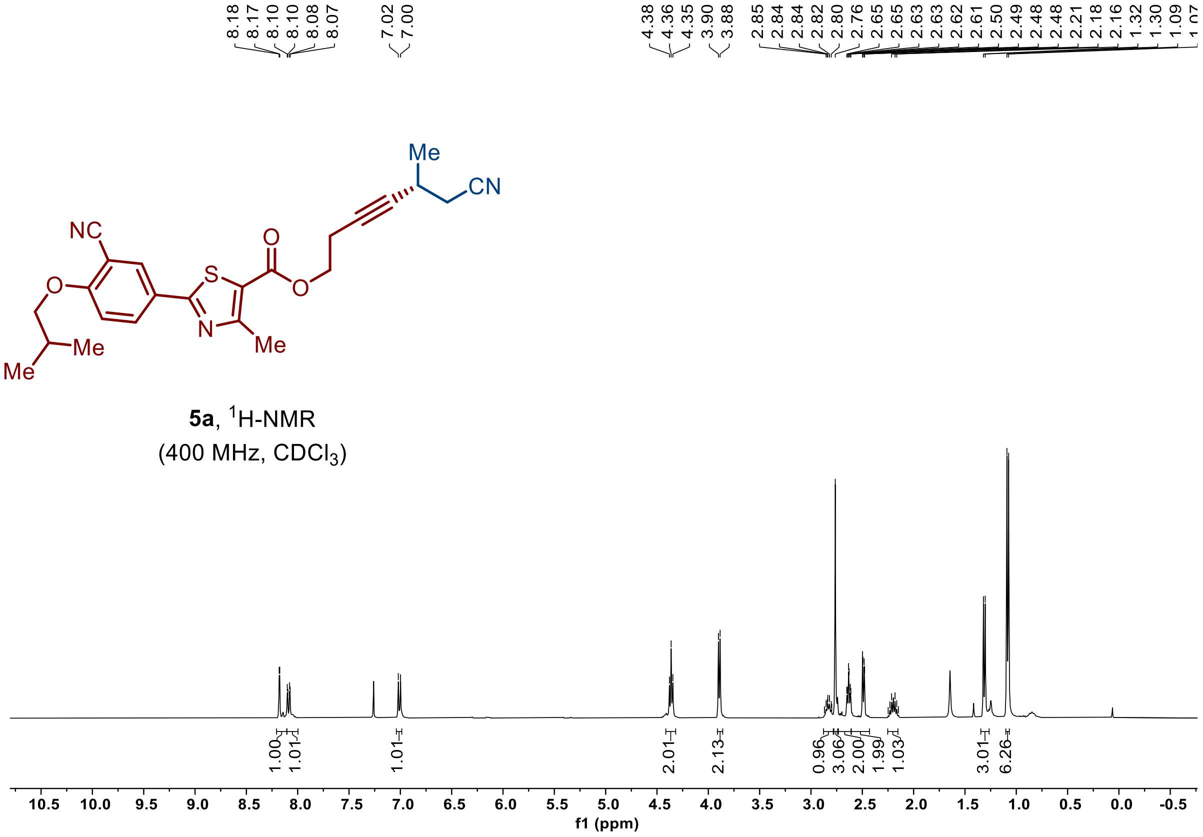

^{13}C NMR (100 MHz, $CDCl_3$) spectrum of 5a

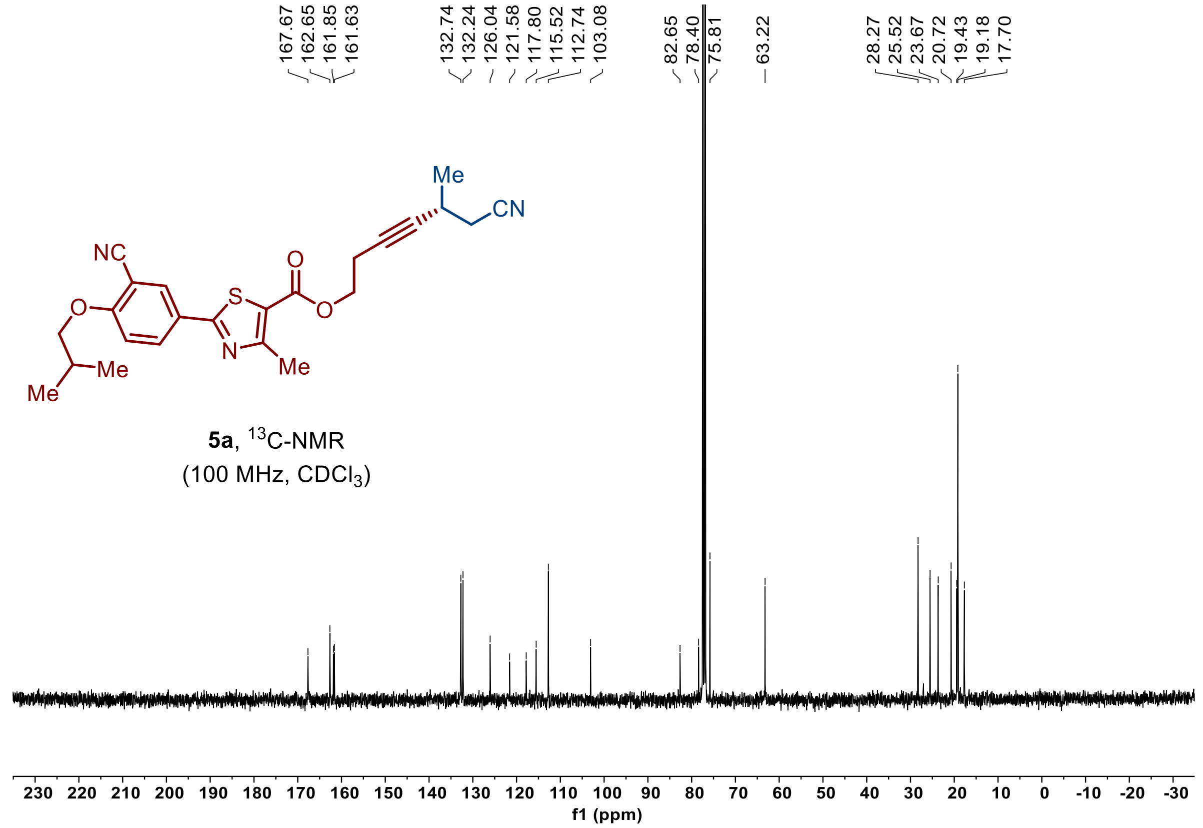

^{1}H NMR (400 MHz, $CDCl_3$) spectrum of 5b

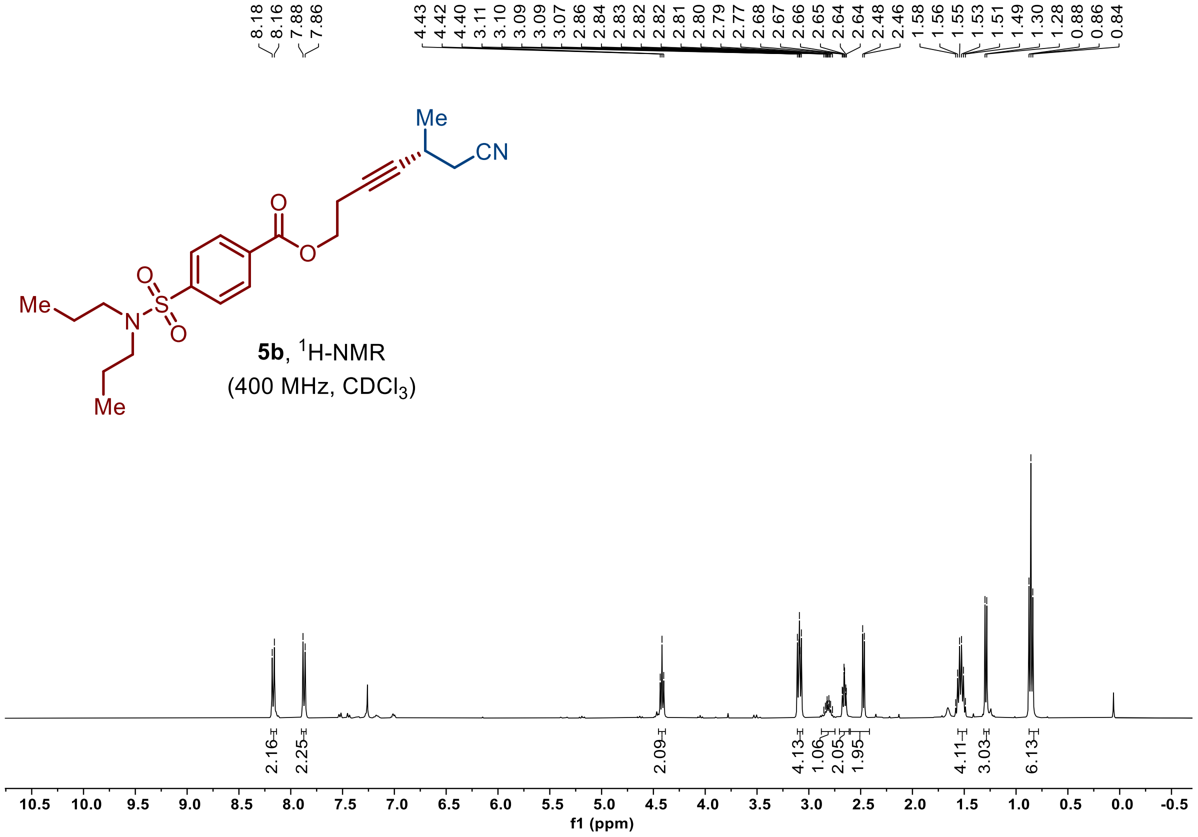

^{13}C NMR (100 MHz, $CDCl_3$) spectrum of 5b

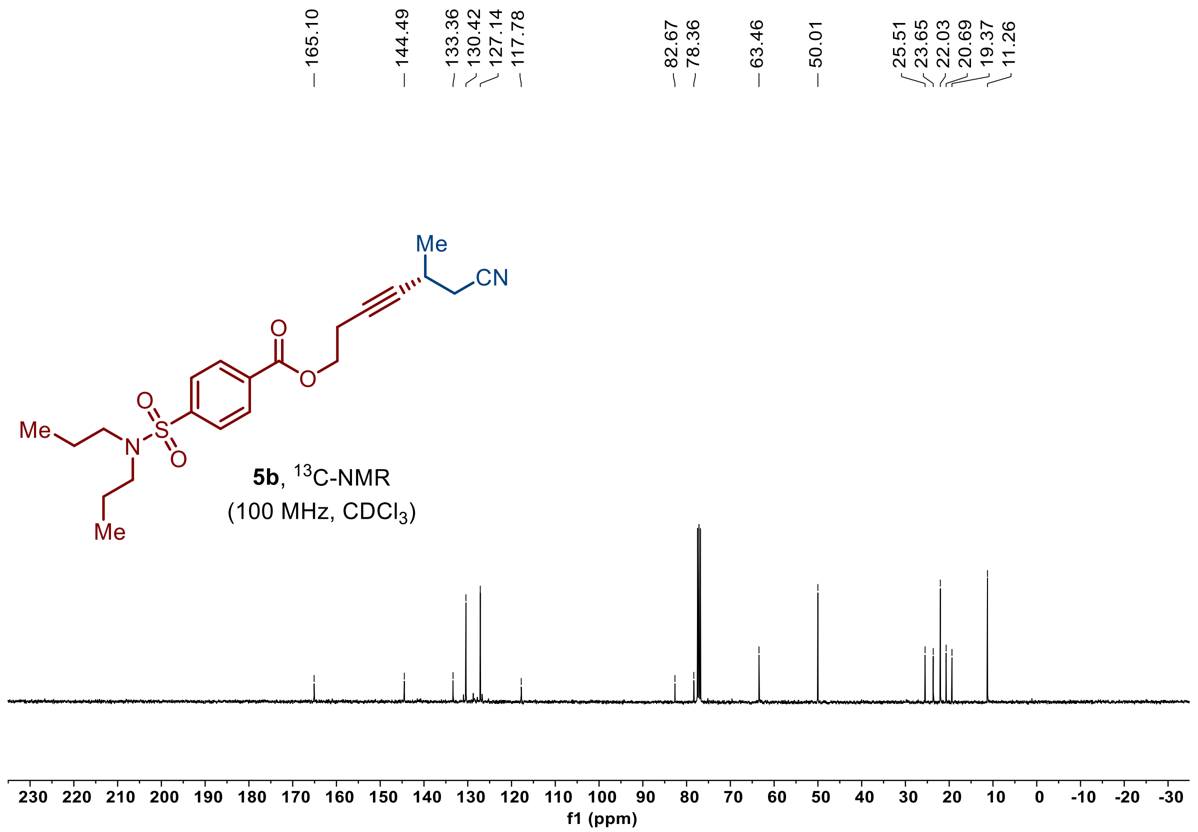

^{1}H NMR (400 MHz, $CDCl_3$) spectrum of 5c

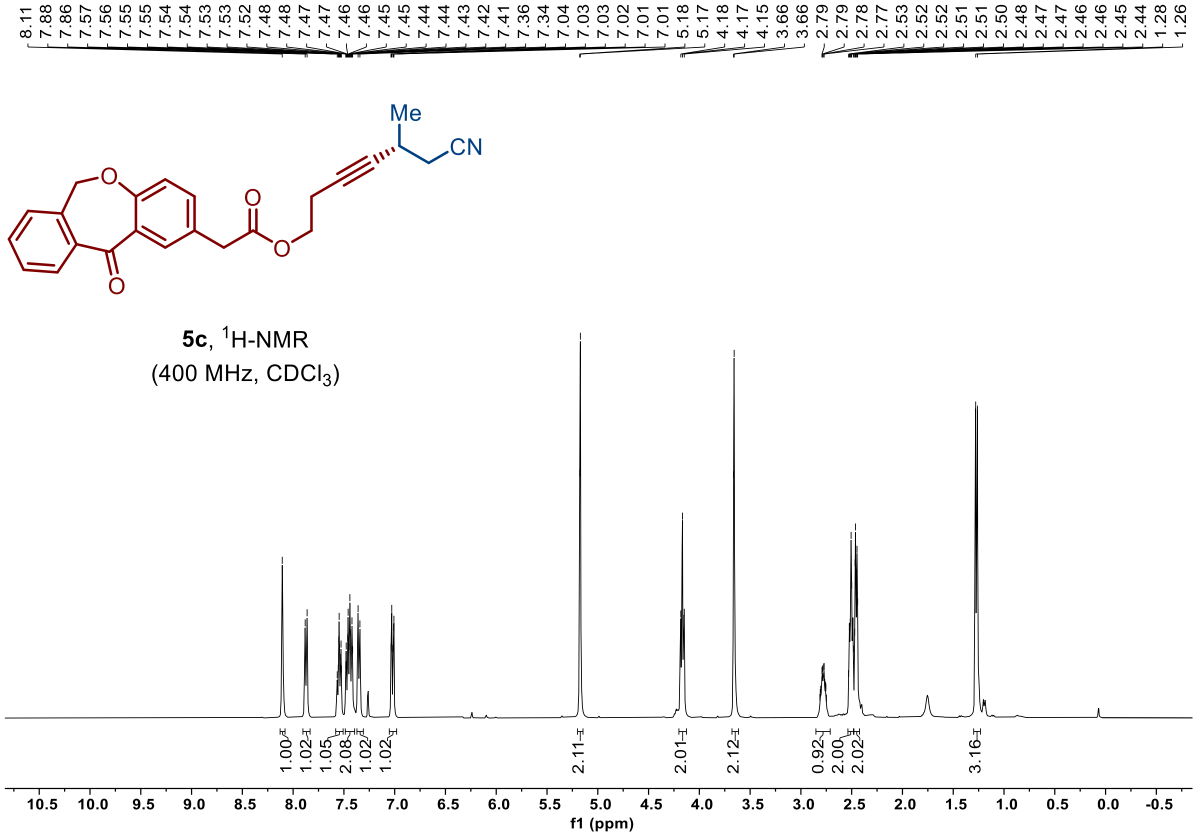

^{13}C NMR (100 MHz, $CDCl_3$) spectrum of 5c

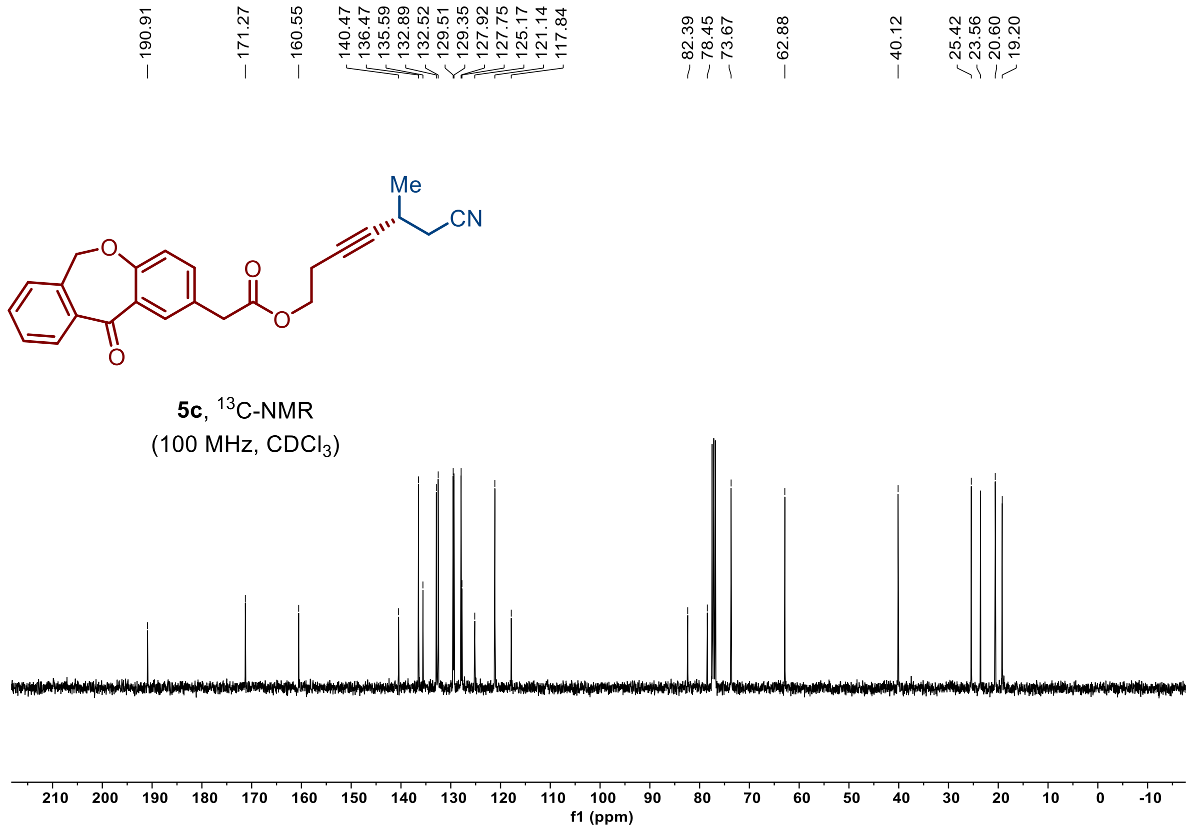

^{1}H NMR (400 MHz, $CDCl_3$) spectrum of 5d

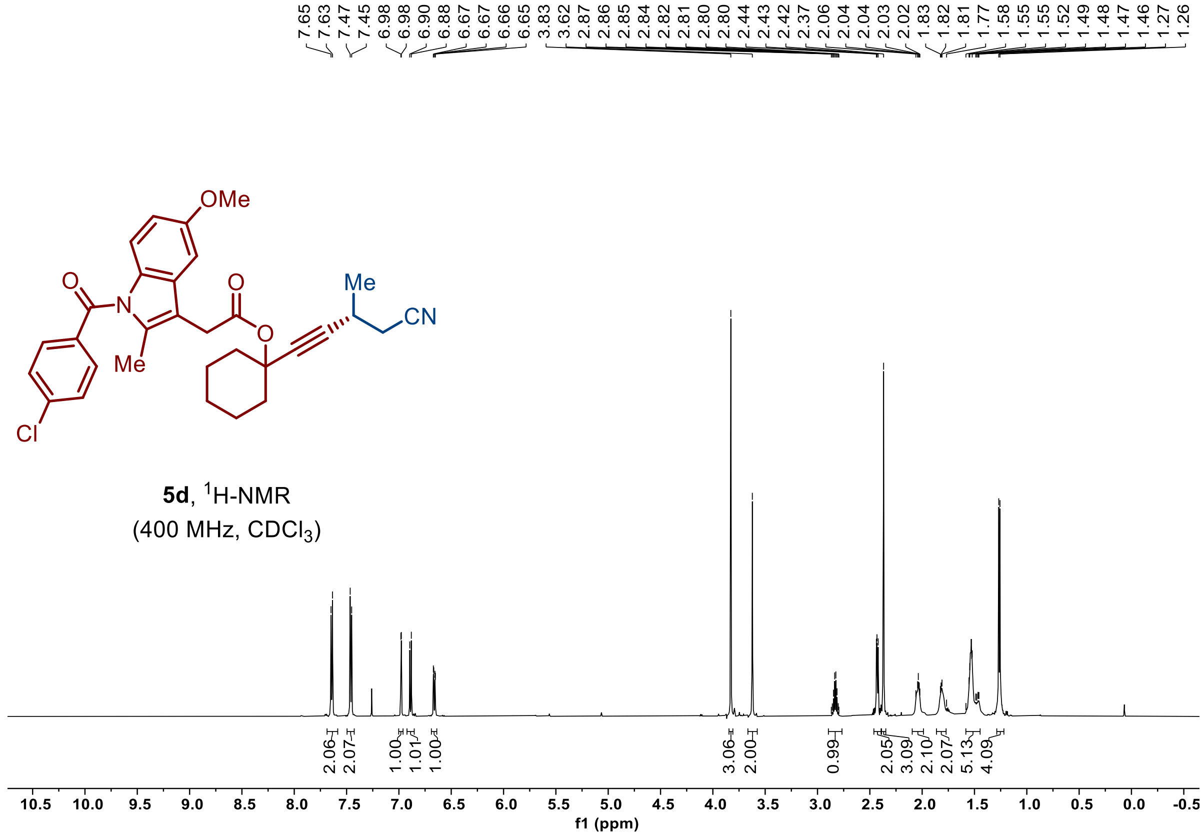

^{13}C NMR (100 MHz, $CDCl_3$) spectrum of 5d

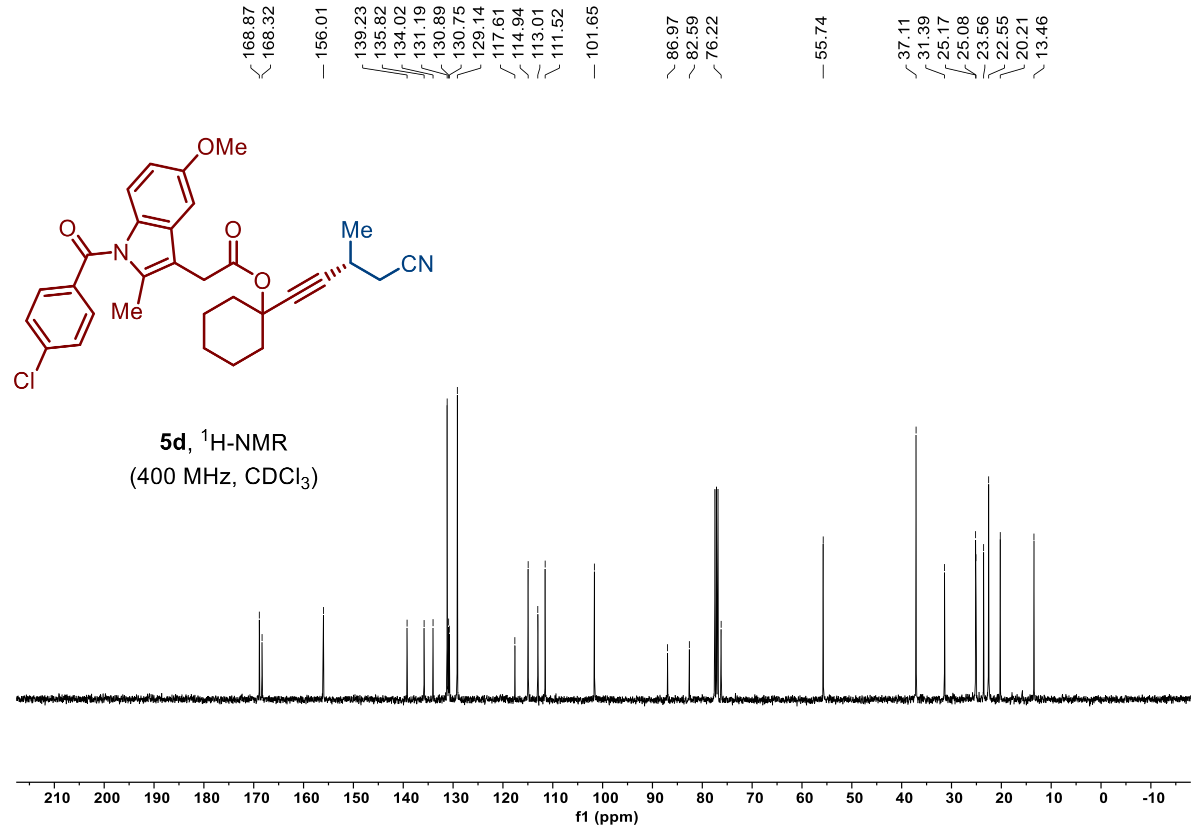

^{1}H NMR (600 MHz, $CDCl_3$) spectrum of 6a

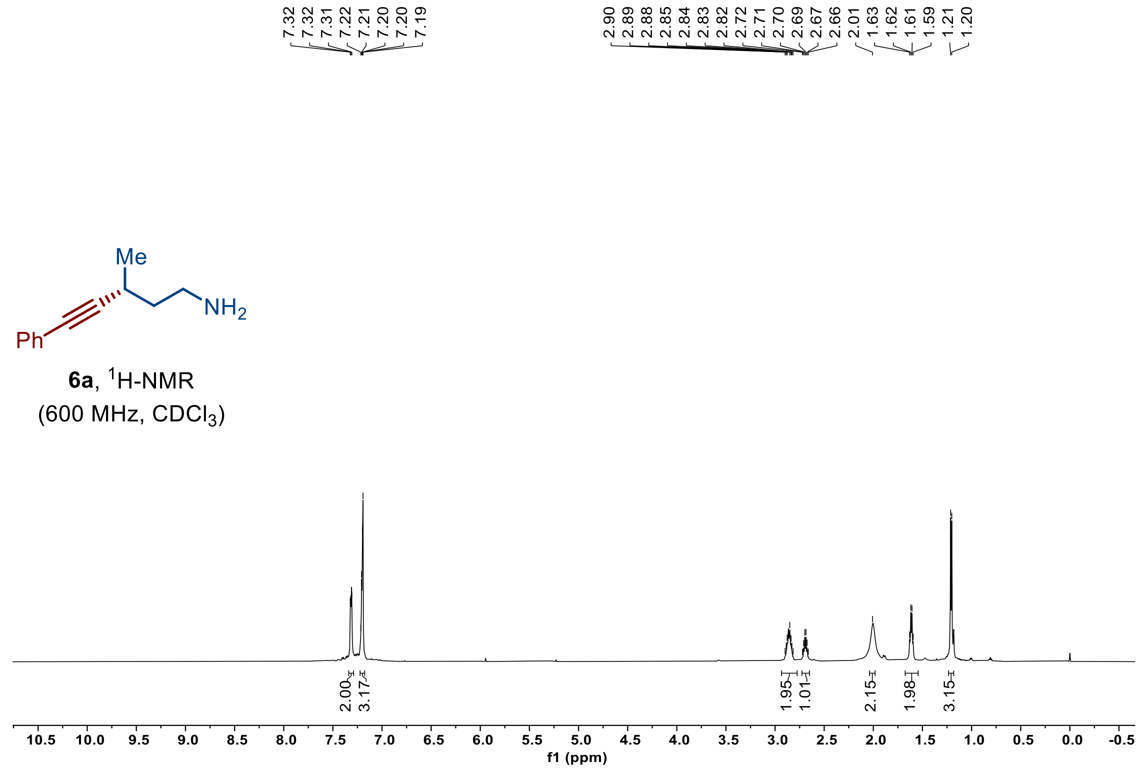

^{13}C NMR (150 MHz, $CDCl_3$) spectrum of 6a

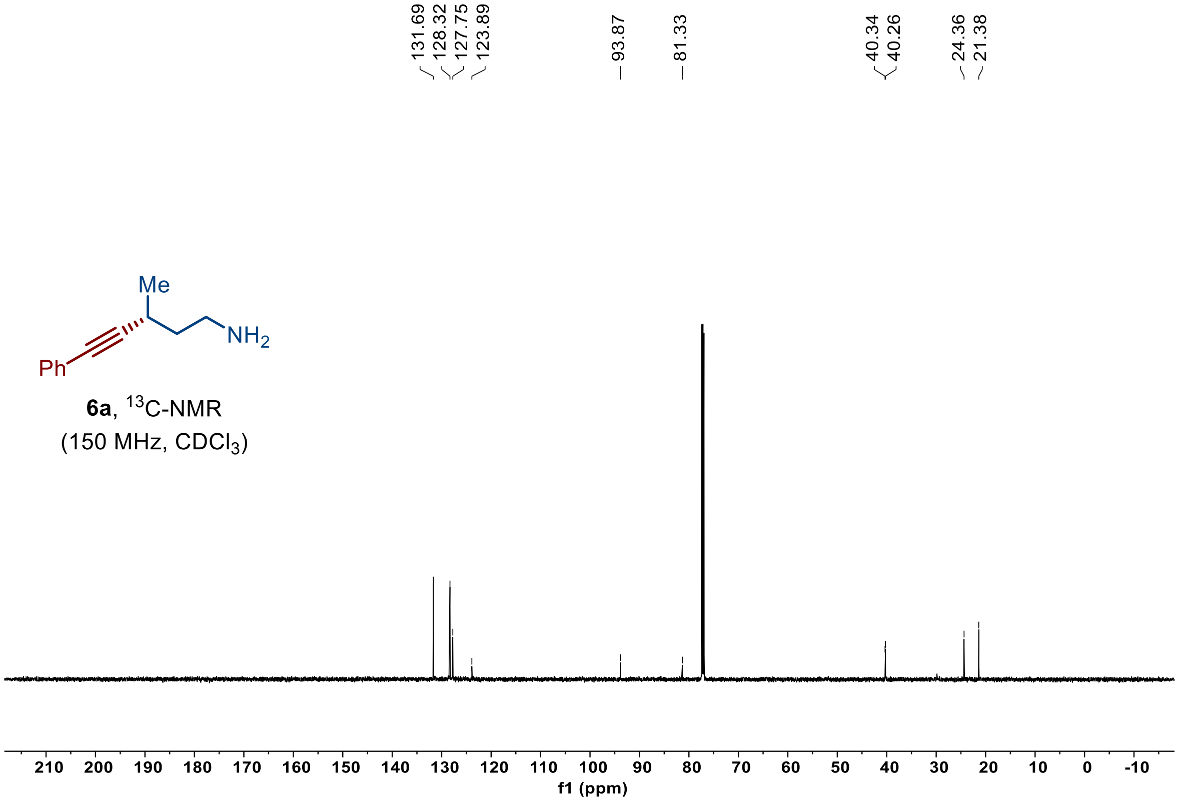

^{1}H NMR (400 MHz, $CDCl_3$) spectrum of 6a'

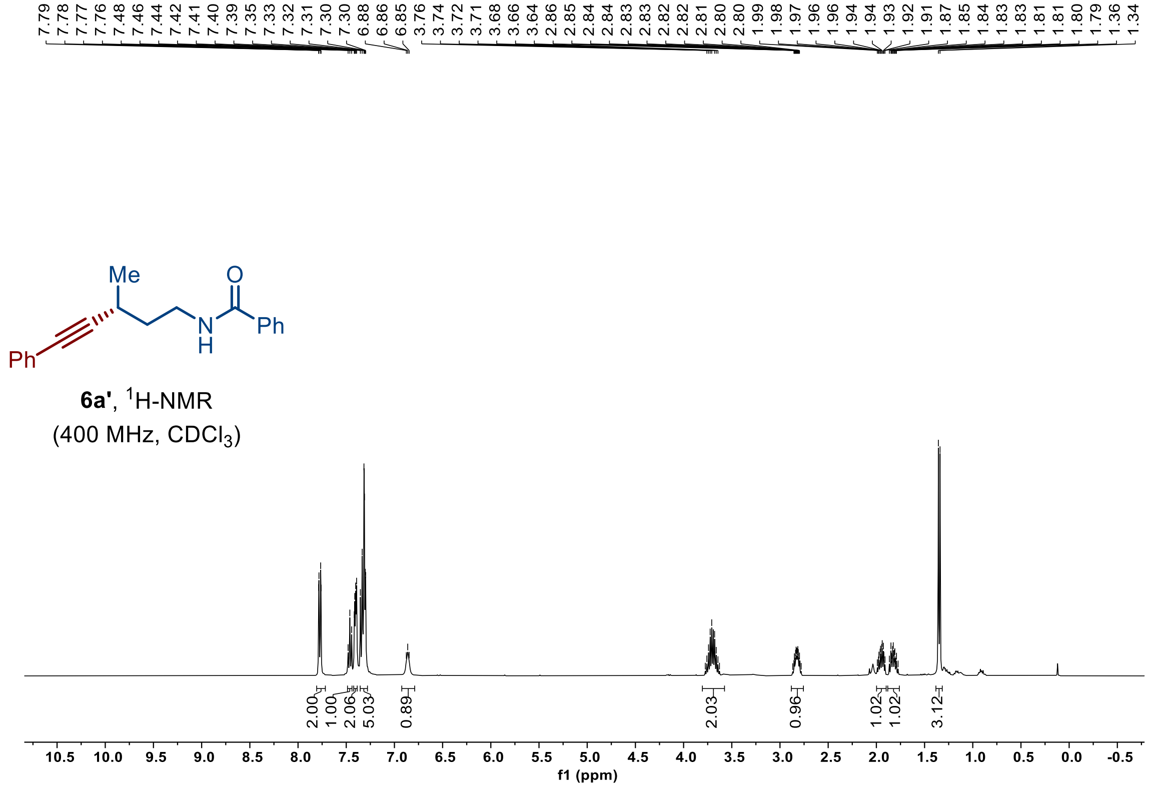

^{13}C NMR (100 MHz, $CDCl_3$) spectrum of 6a'

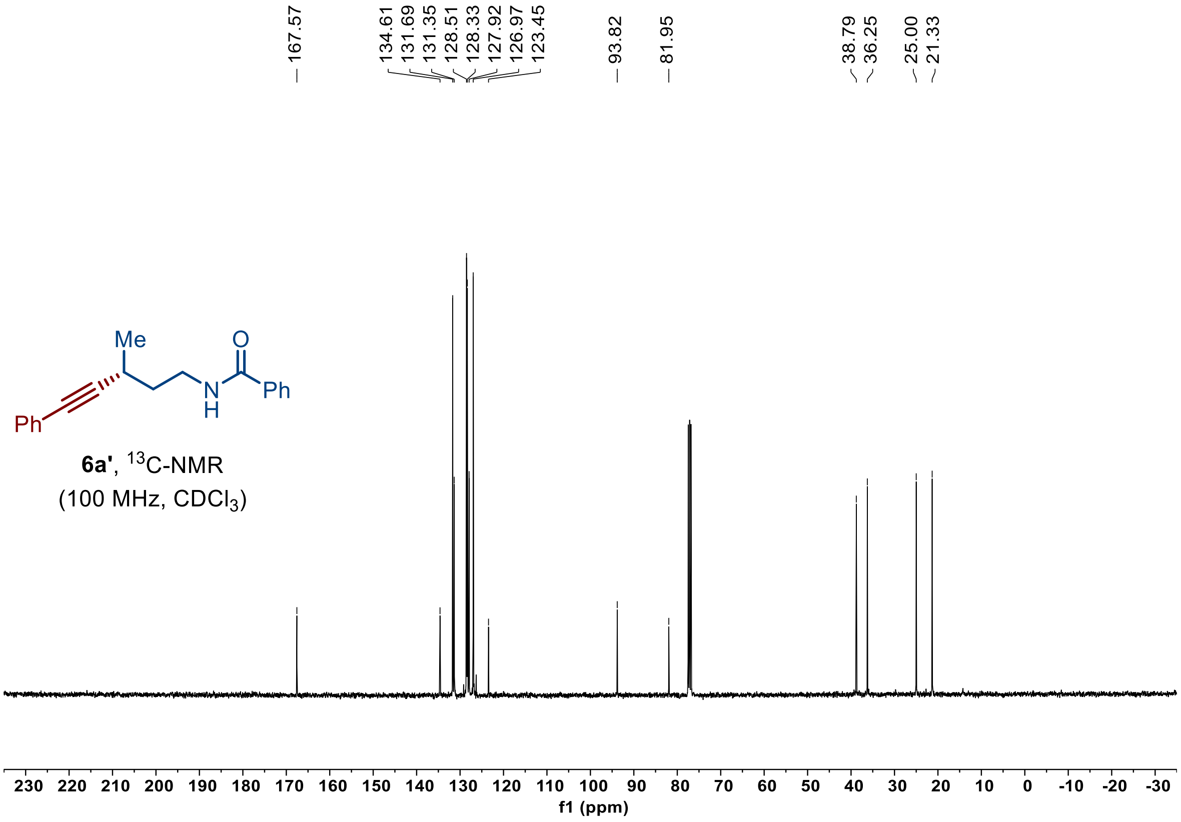

^{1}H NMR (400 MHz, $CDCl_3$) spectrum of 6b

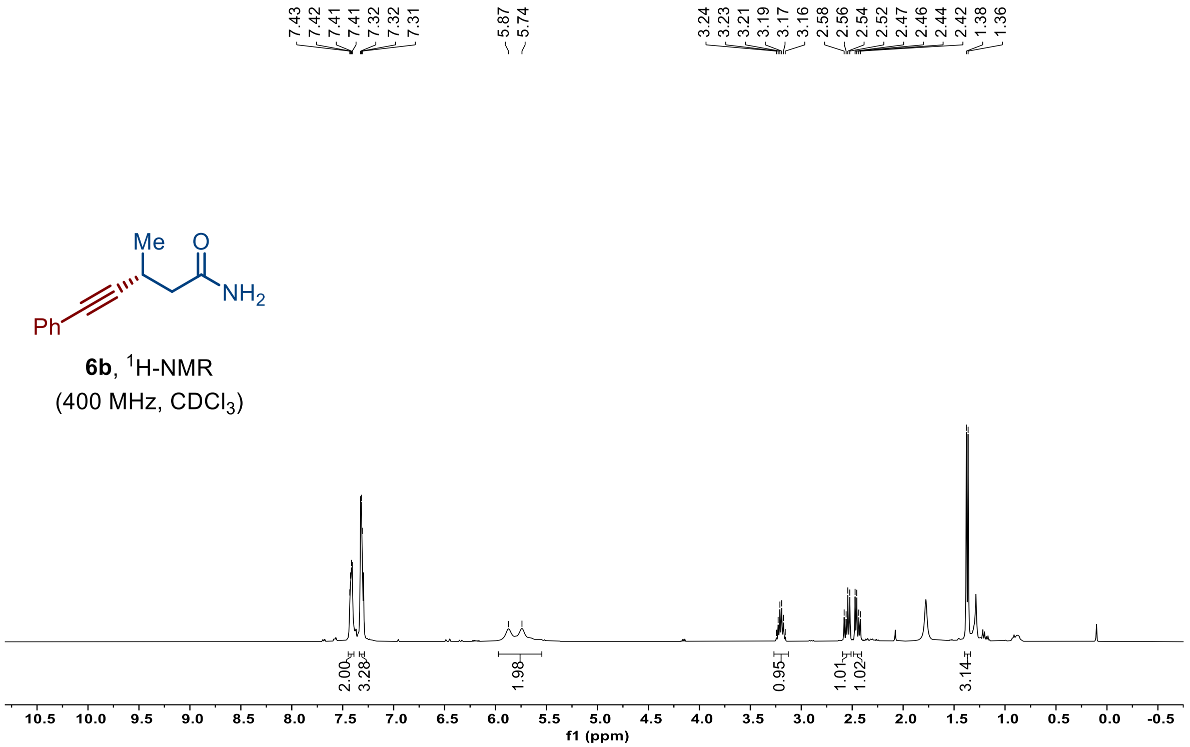

^{13}C NMR (100 MHz, $CDCl_3$) spectrum of 6b

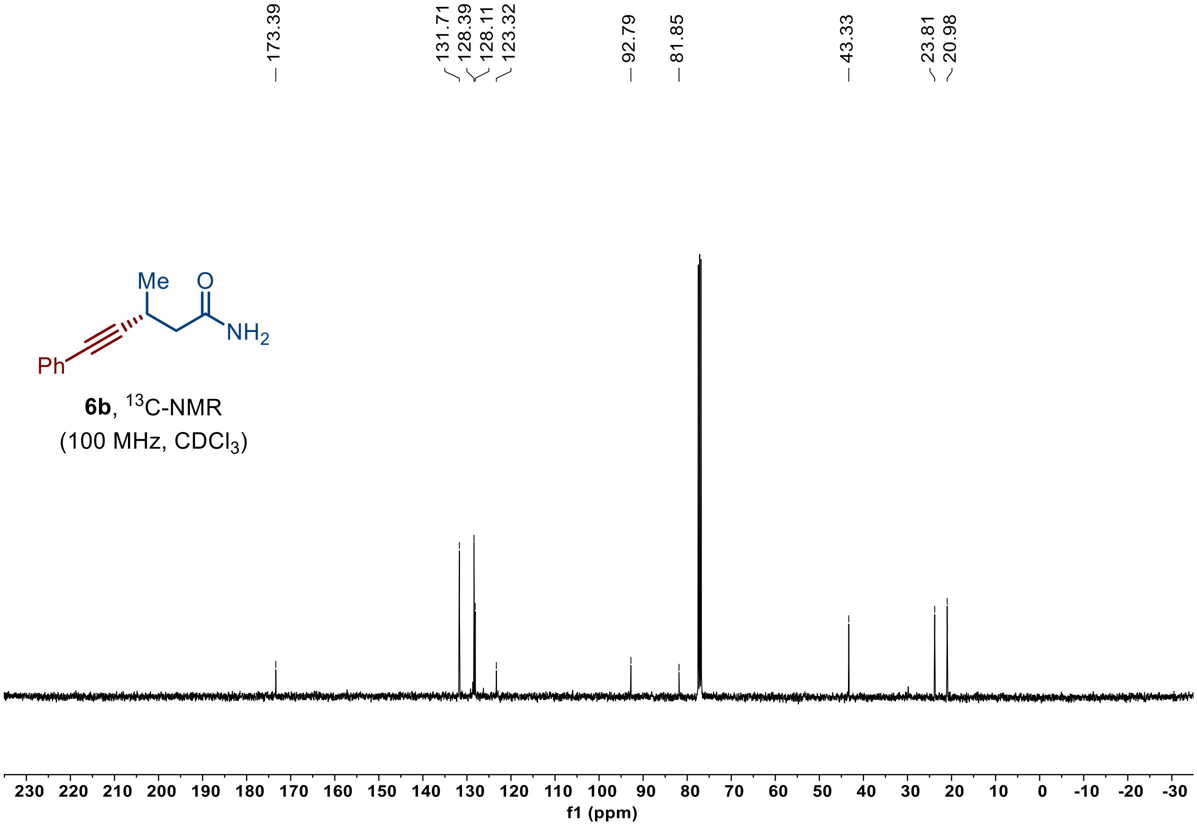

^{1}H NMR (400 MHz, CDCl$_3$) spectrum of 6c

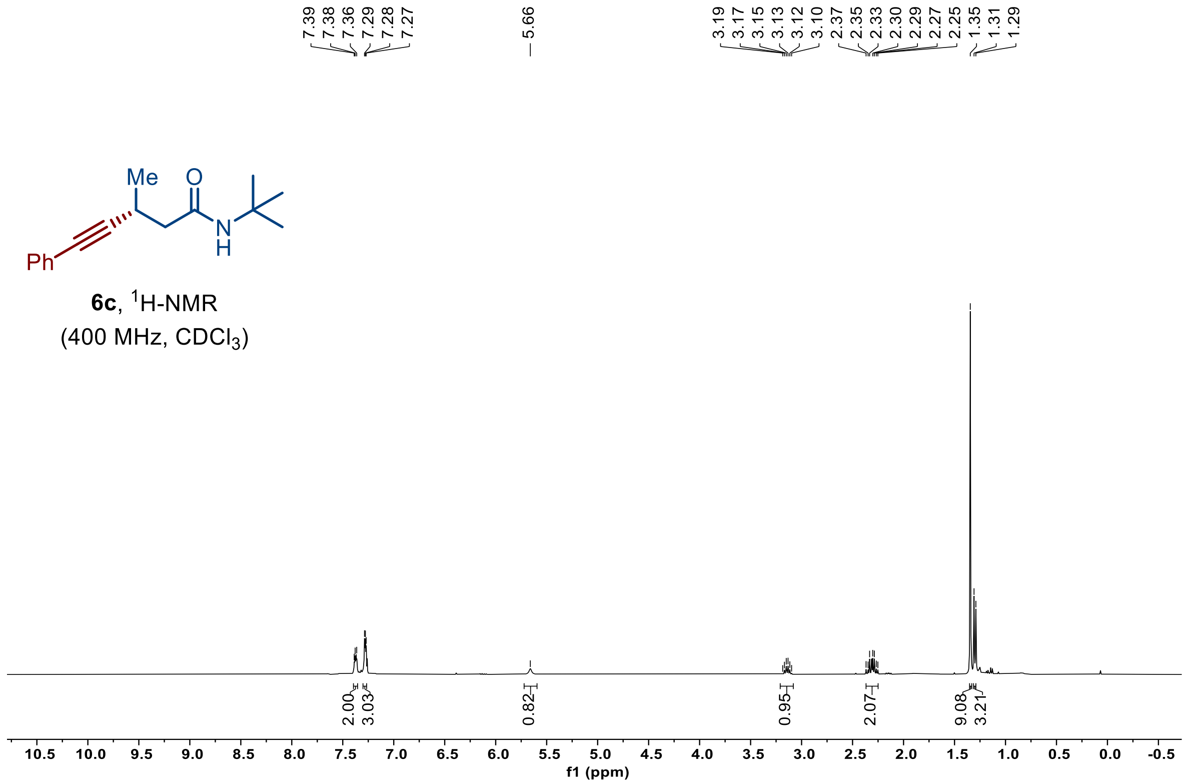

^{13}C NMR (100 MHz, CDCl$_3$) spectrum of 6c

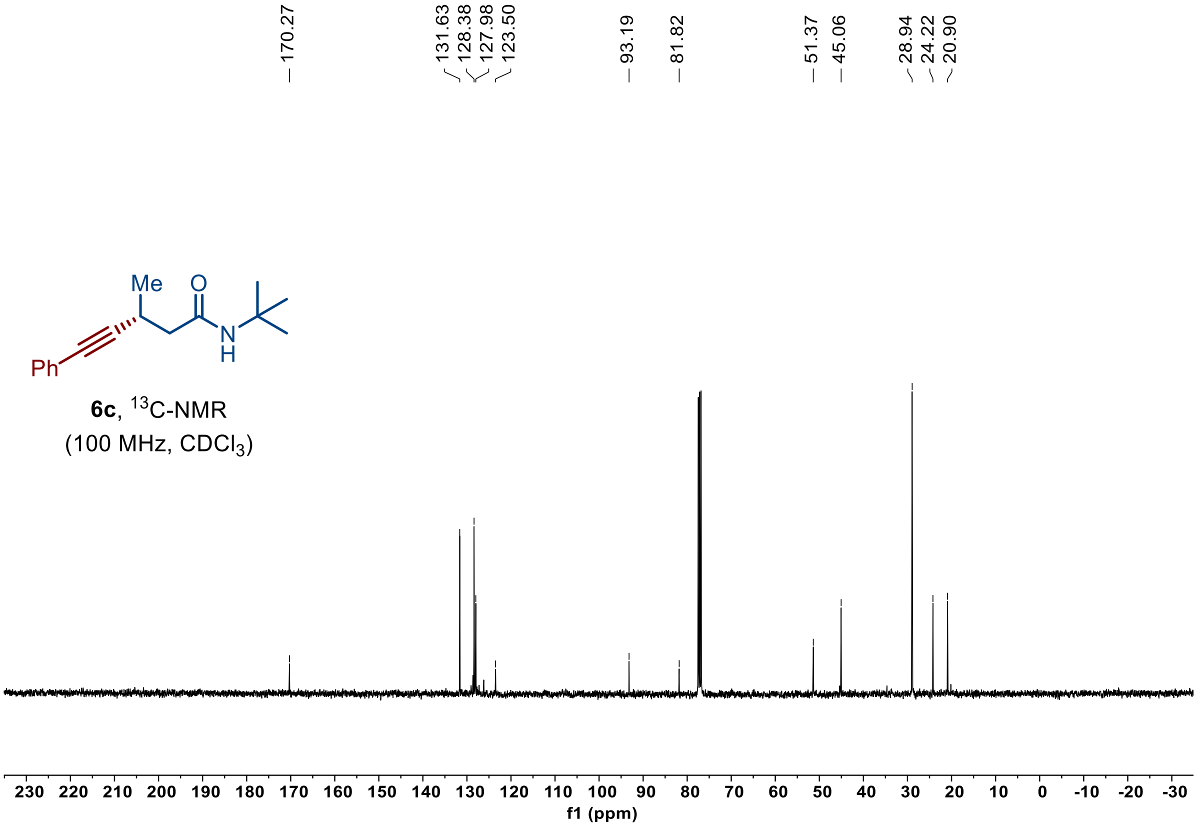

¹H NMR (400 MHz, $CDCl_3$) spectrum of 6d

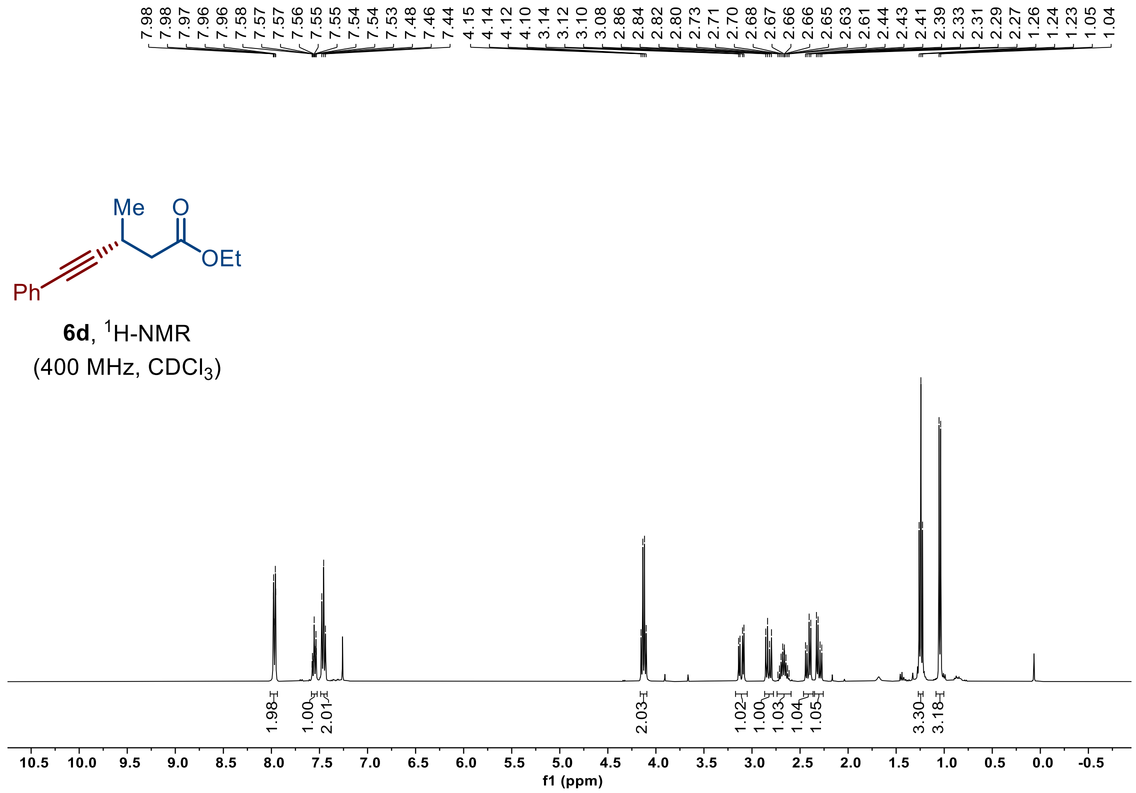

¹³C NMR (100 MHz, $CDCl_3$) spectrum of 6d

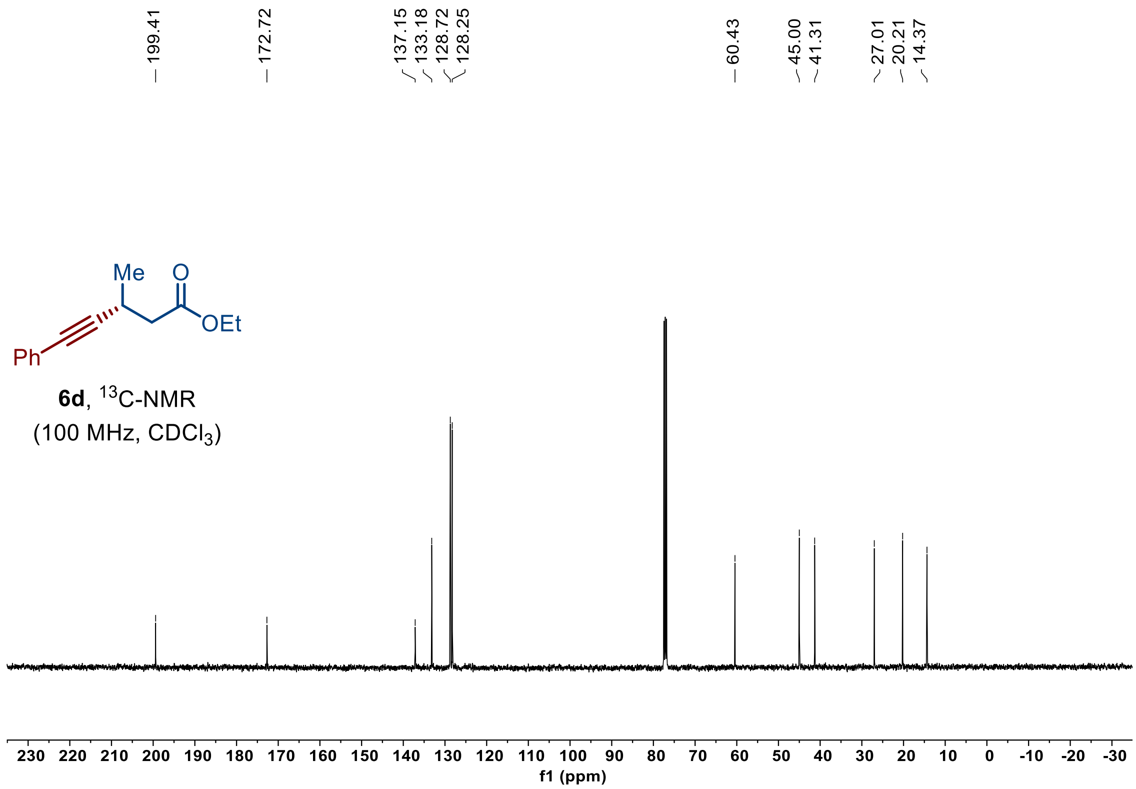

^{1}H NMR (600 MHz, $CDCl_3$) spectrum of 6e

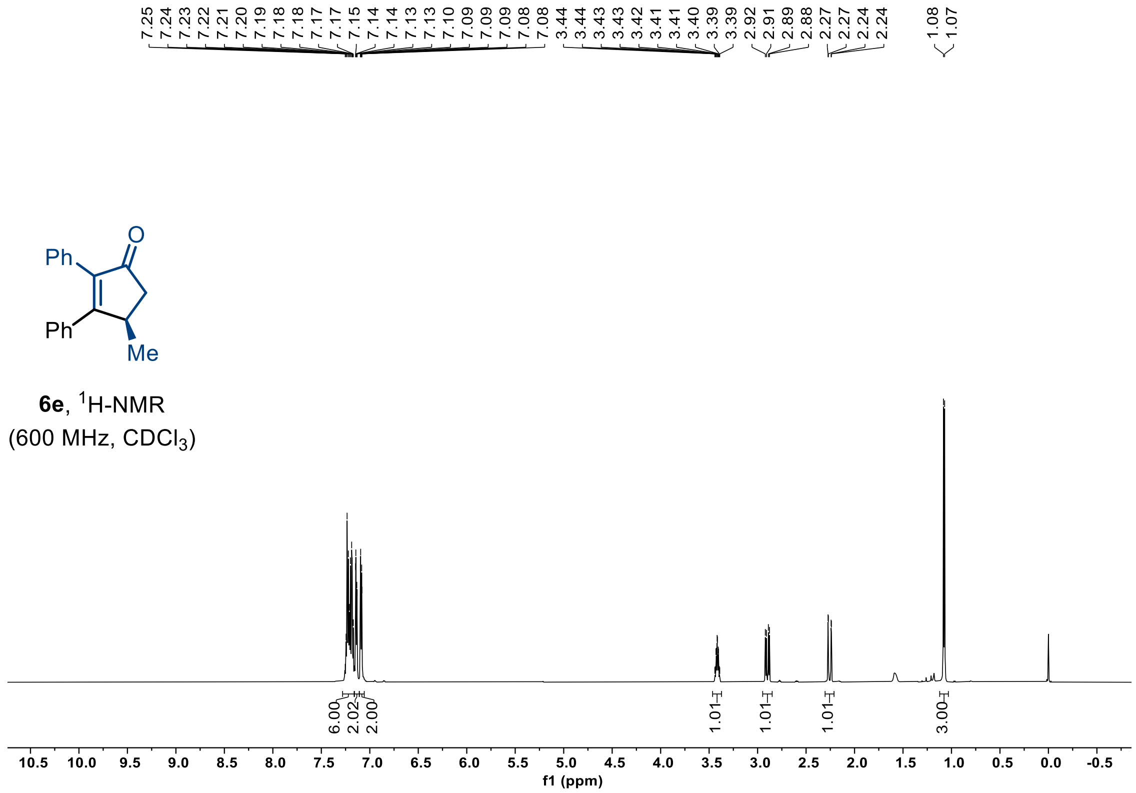

^{13}C NMR (150 MHz, $CDCl_3$) spectrum of 6e

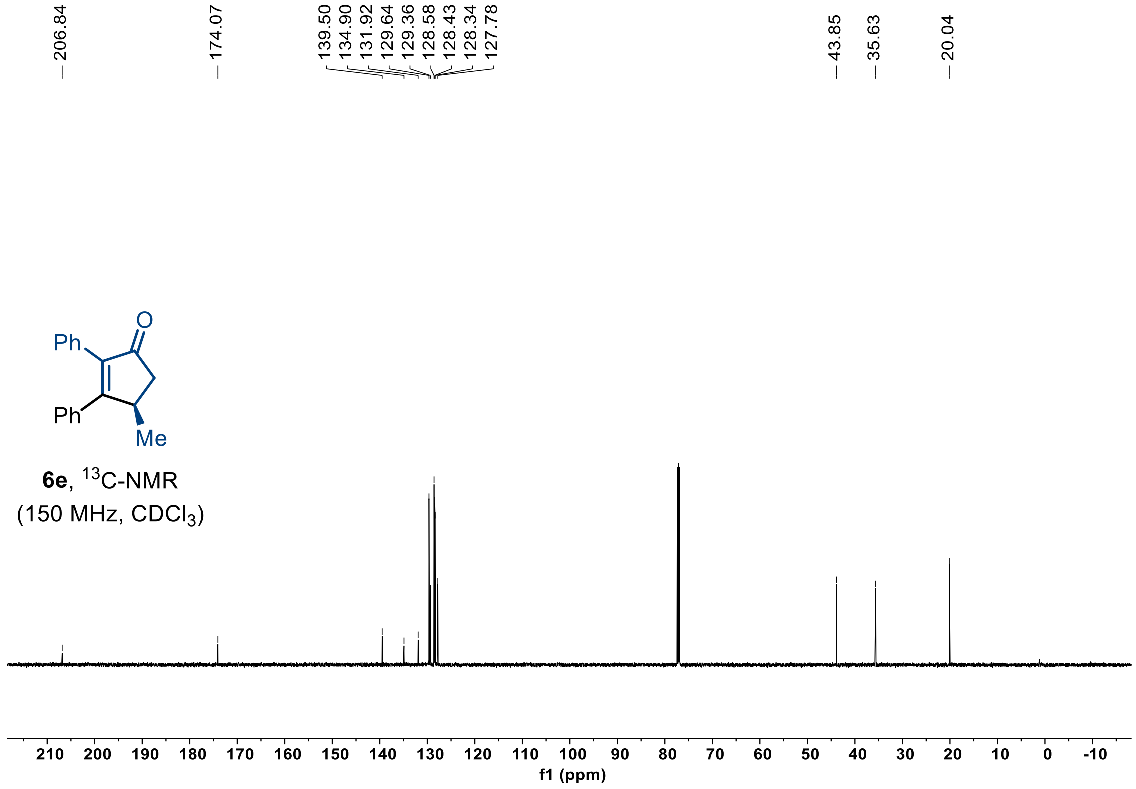

^{1}H NMR (600 MHz, $CDCl_3$) spectrum of 6f

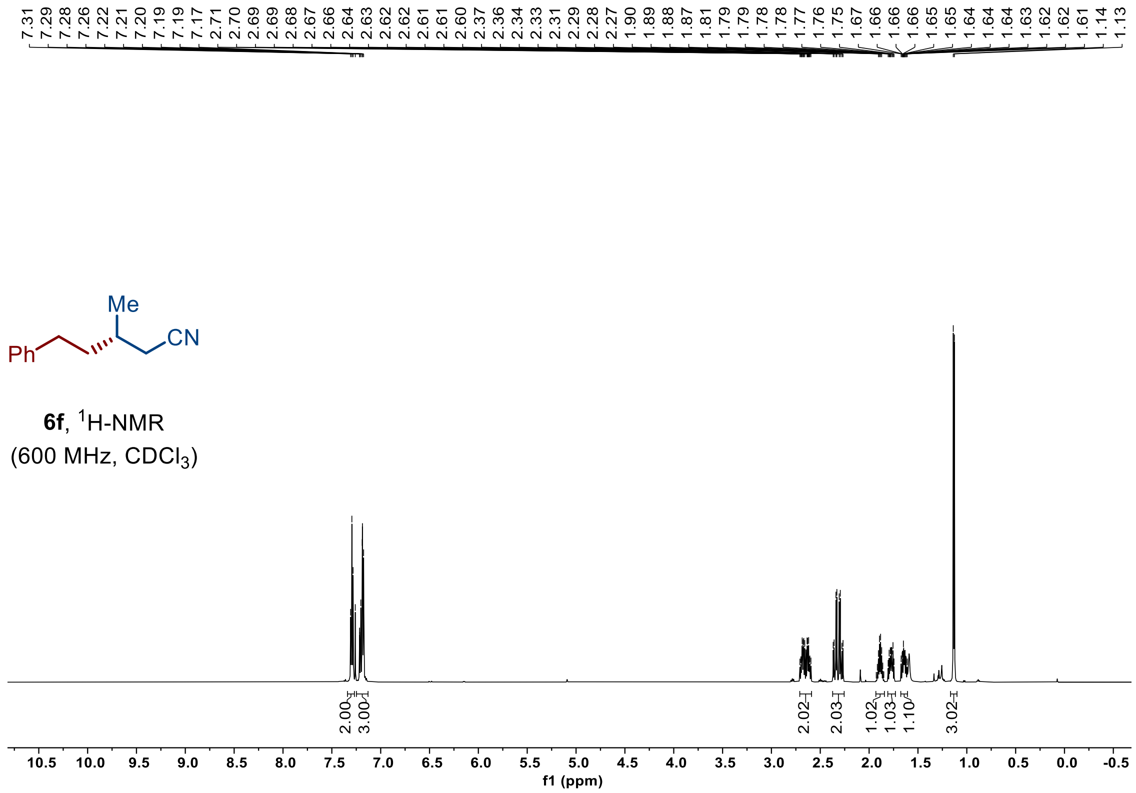

^{13}C NMR (150 MHz, $CDCl_3$) spectrum of 6f

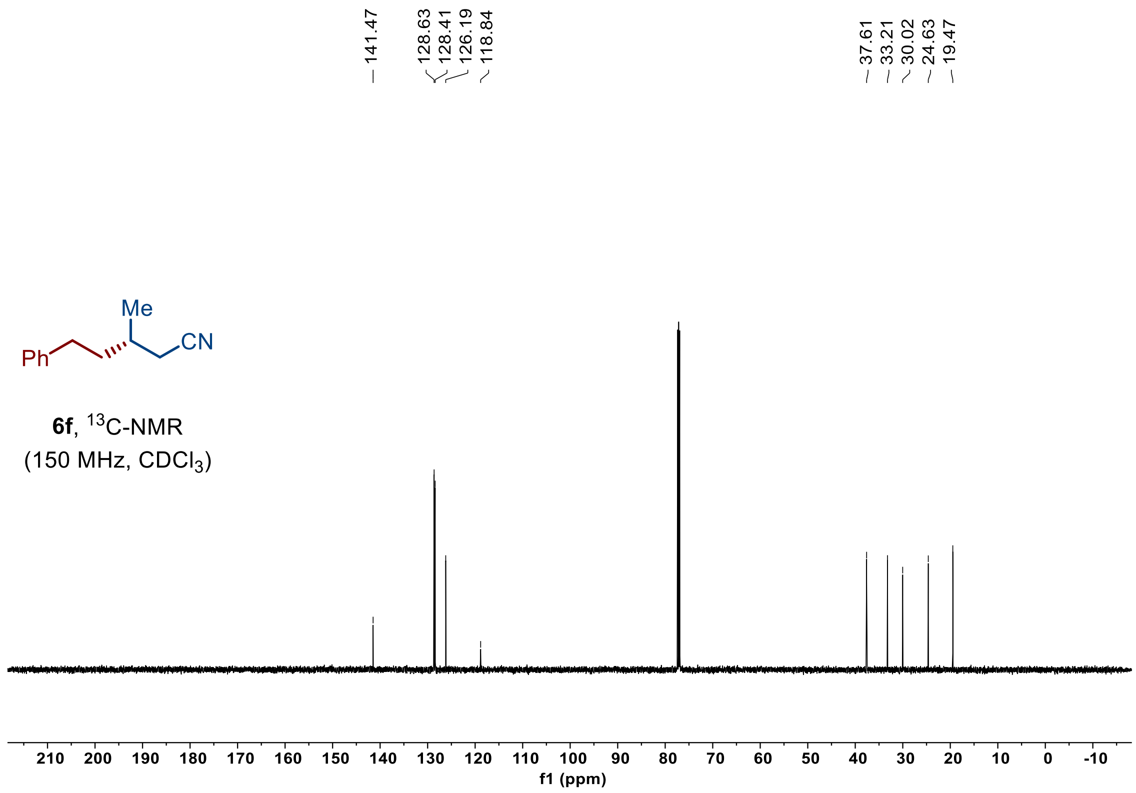

^{1}H NMR (600 MHz, $CDCl_3$) spectrum of 6g

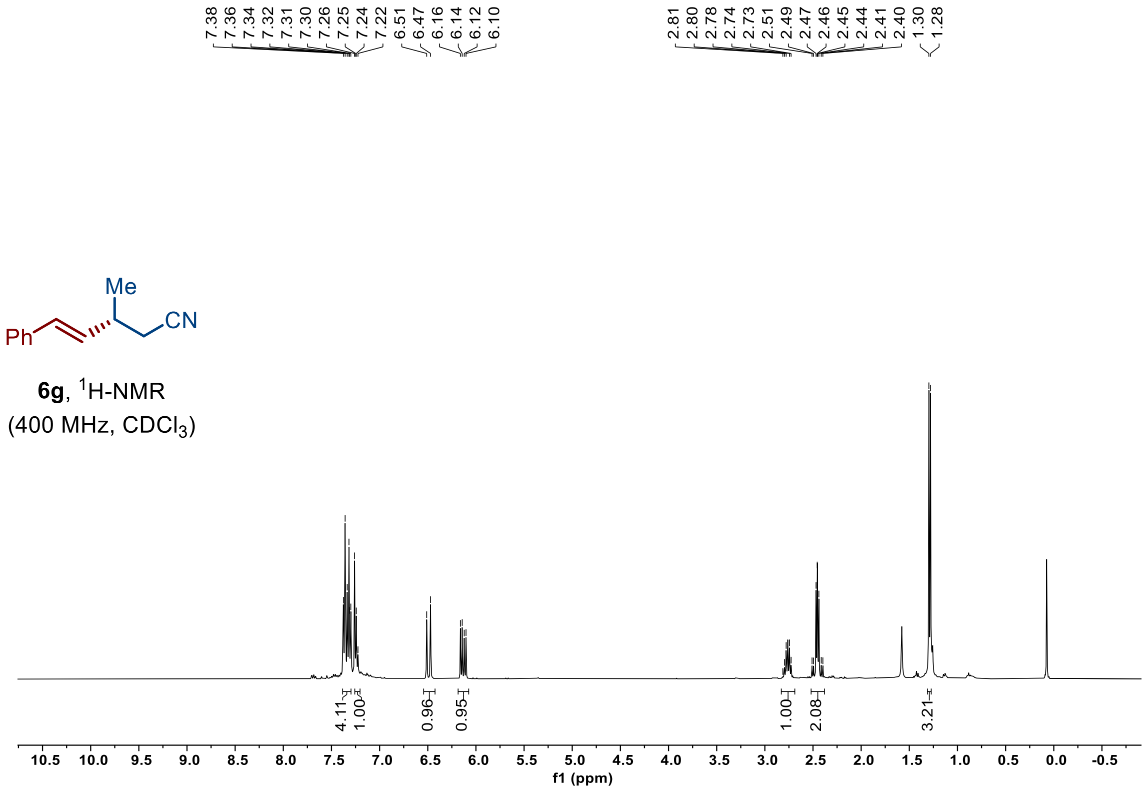

^{13}C NMR (150 MHz, $CDCl_3$) spectrum of 6g

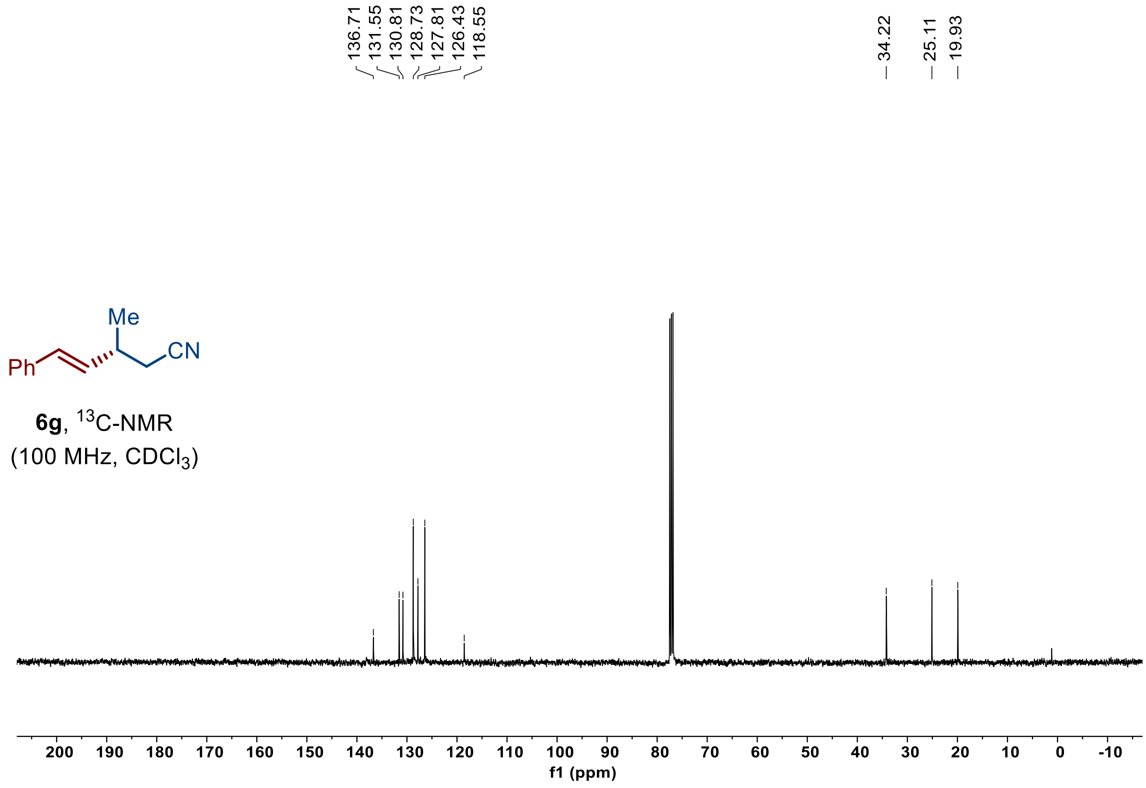

^{1}H NMR (600 MHz, CDCl$_3$) spectrum of 6f

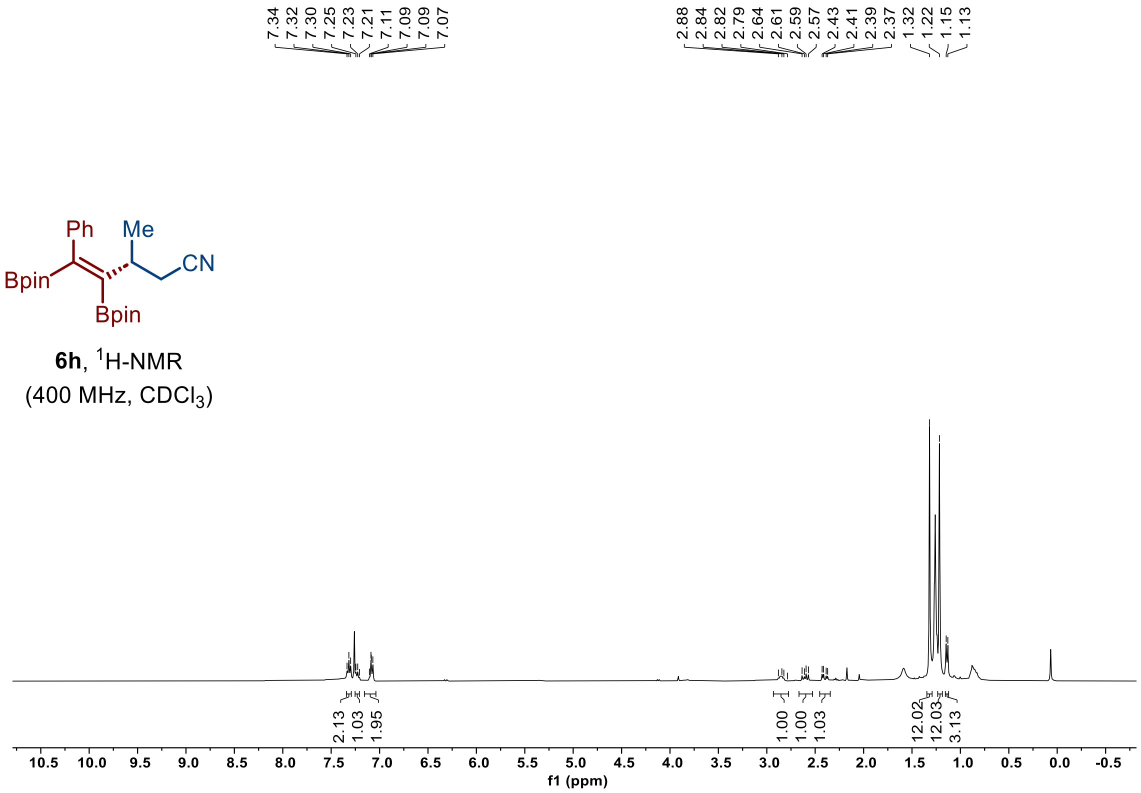

^{13}C NMR (150 MHz, CDCl$_3$) spectrum of 6h

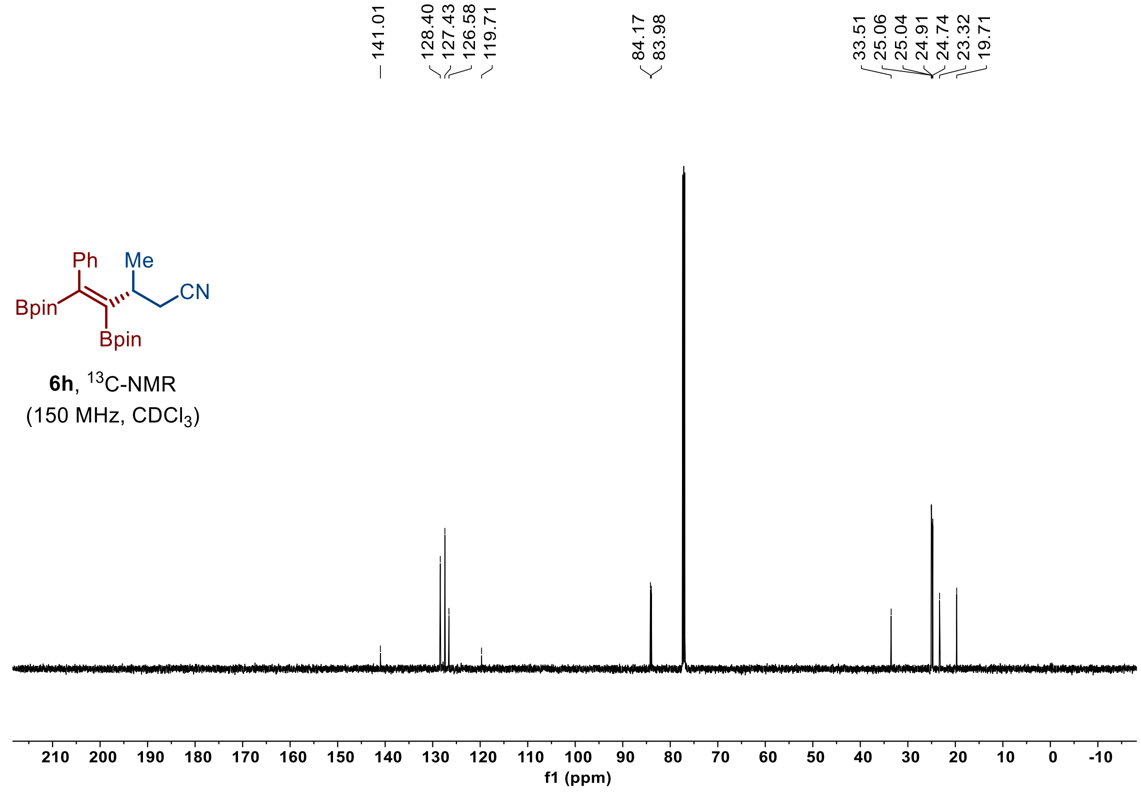

^{1}H NMR (400 MHz, $CDCl_3$) spectrum of 6i

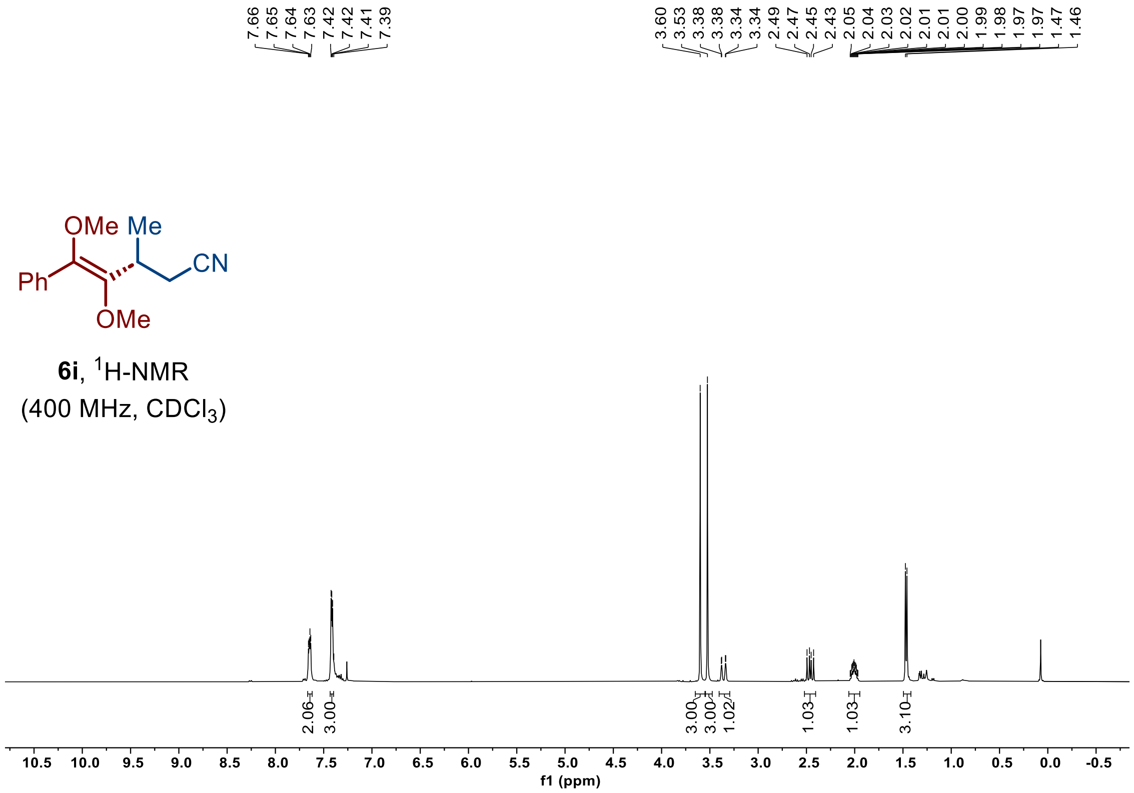

^{13}C NMR (100 MHz, $CDCl_3$) spectrum of 6i

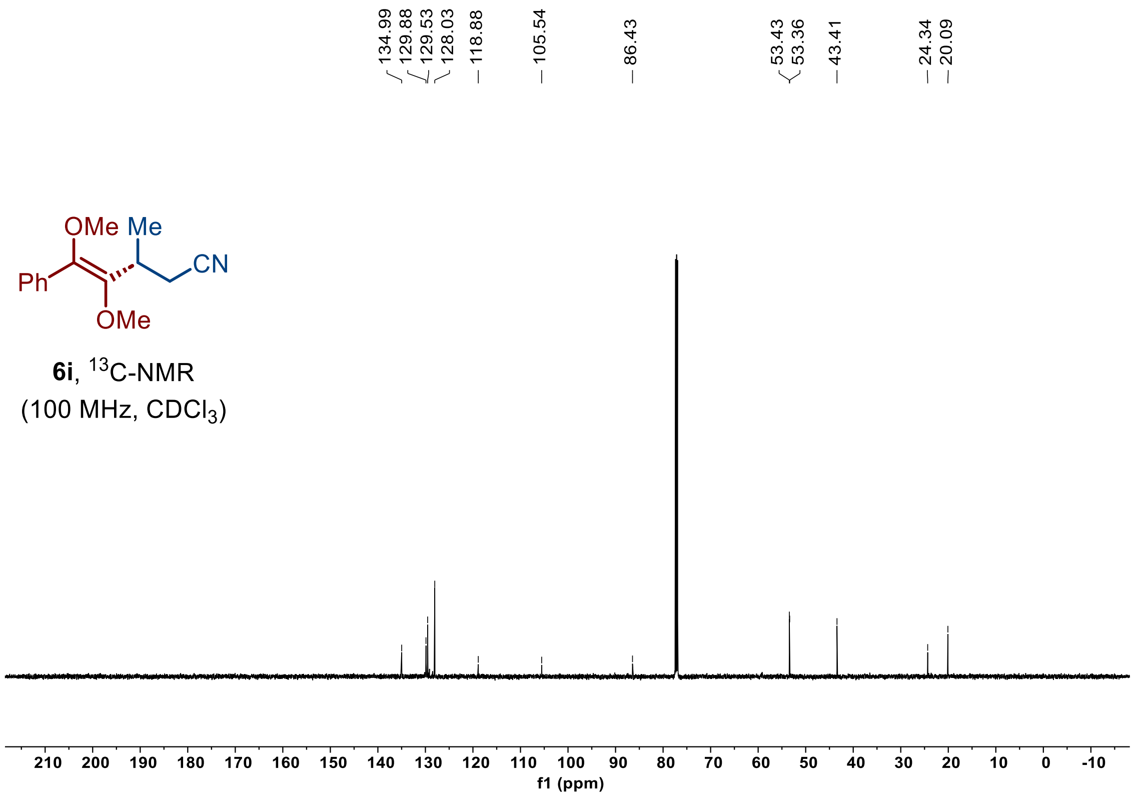

^{1}H NMR (400 MHz, $CDCl_3$) spectrum of 7a

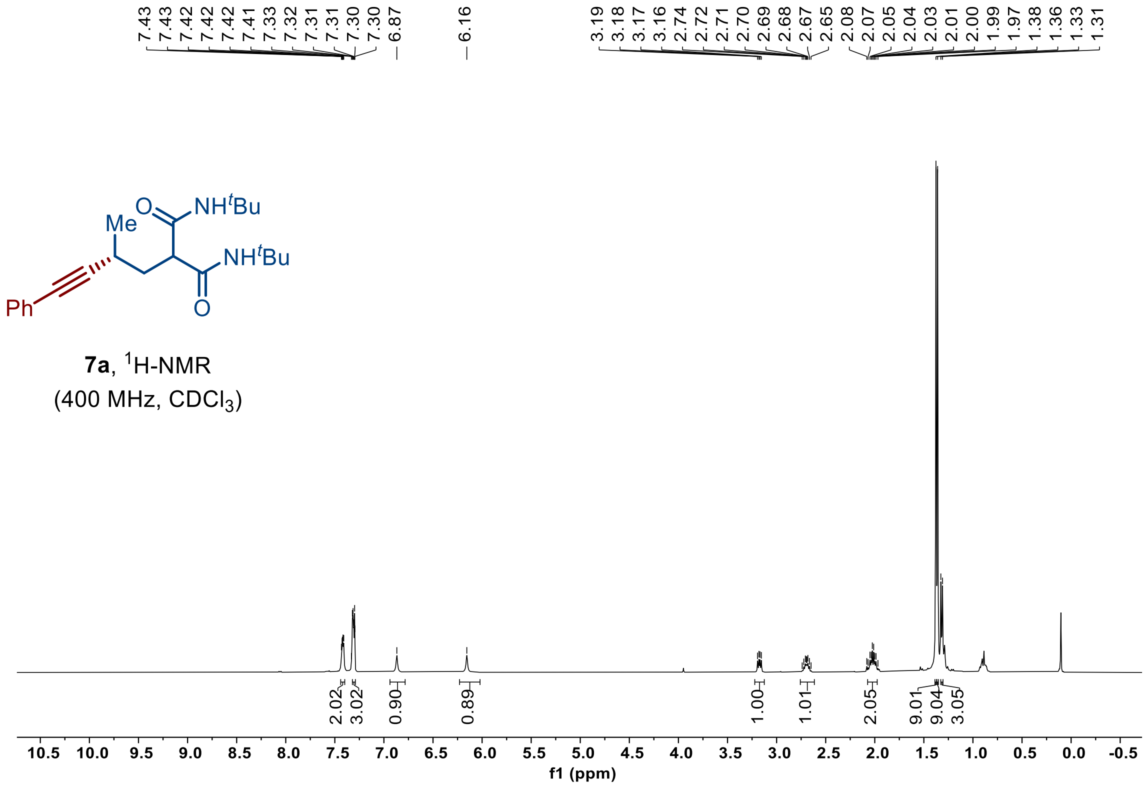

^{13}C NMR (100 MHz, $CDCl_3$) spectrum of 7a

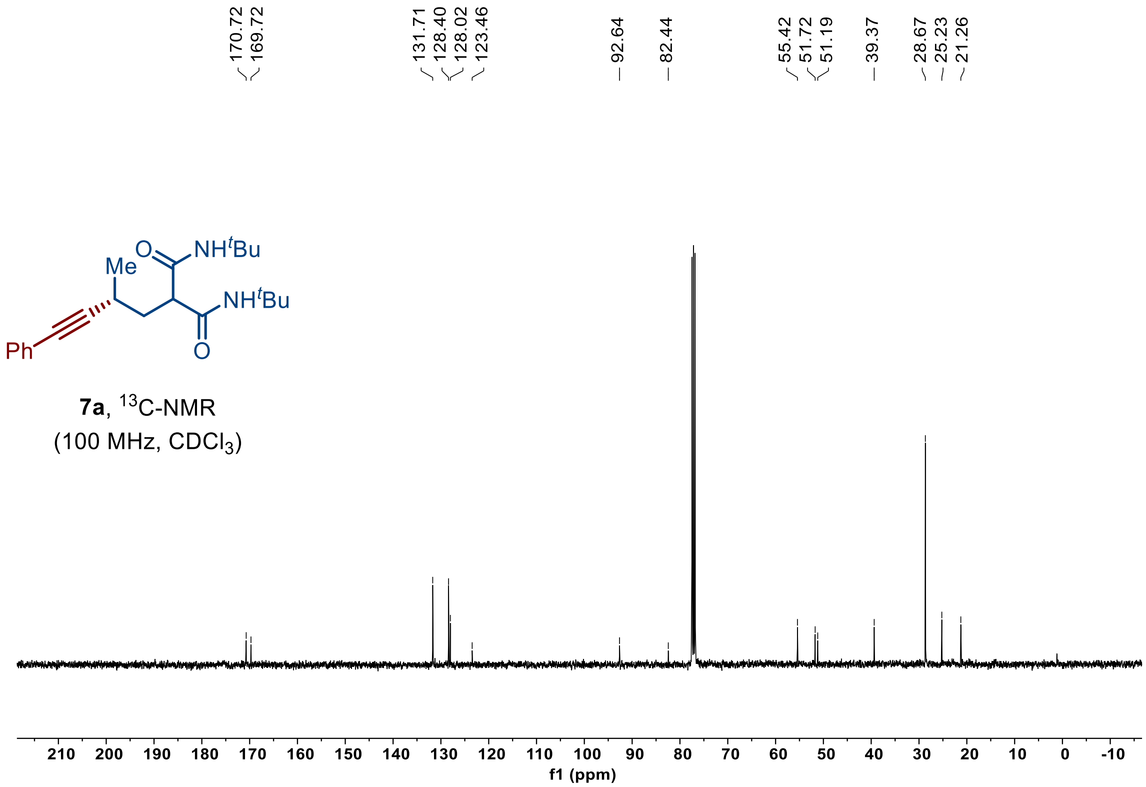

^{1}H NMR (600 MHz, $CDCl_3$) spectrum of 3a-*d*

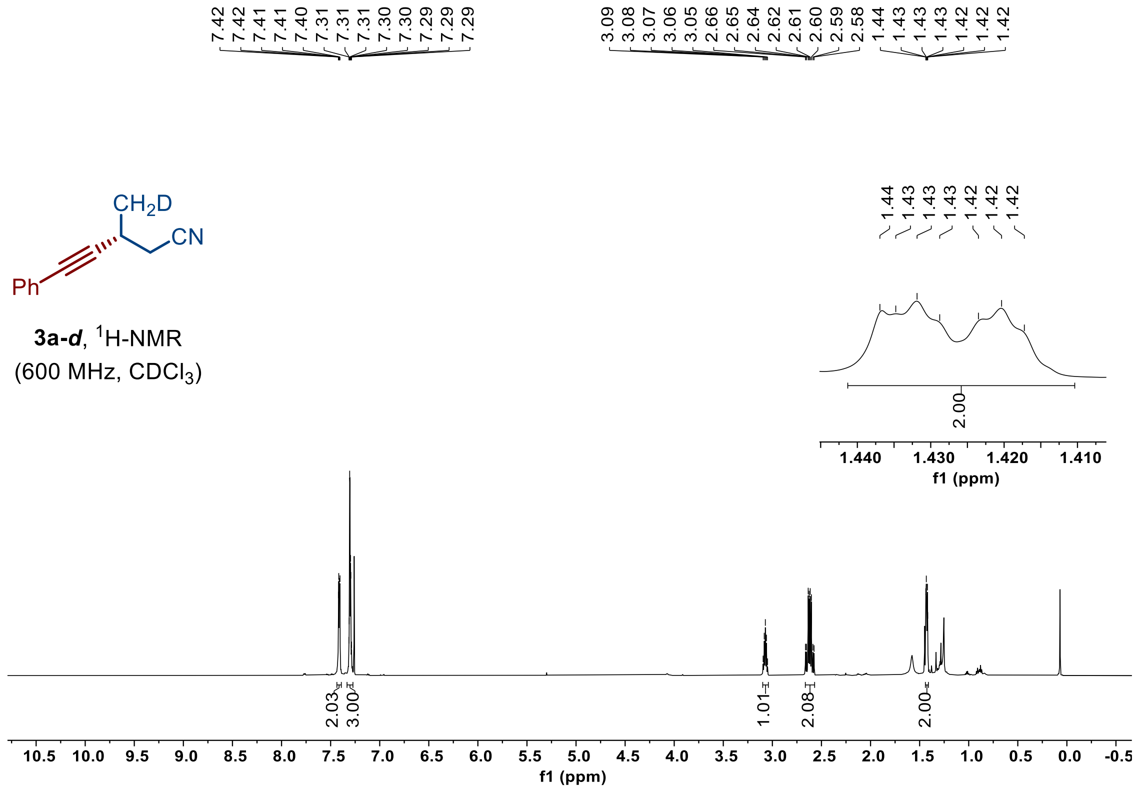

^{13}C NMR (600 MHz, $CDCl_3$) spectrum of 3a-*d*

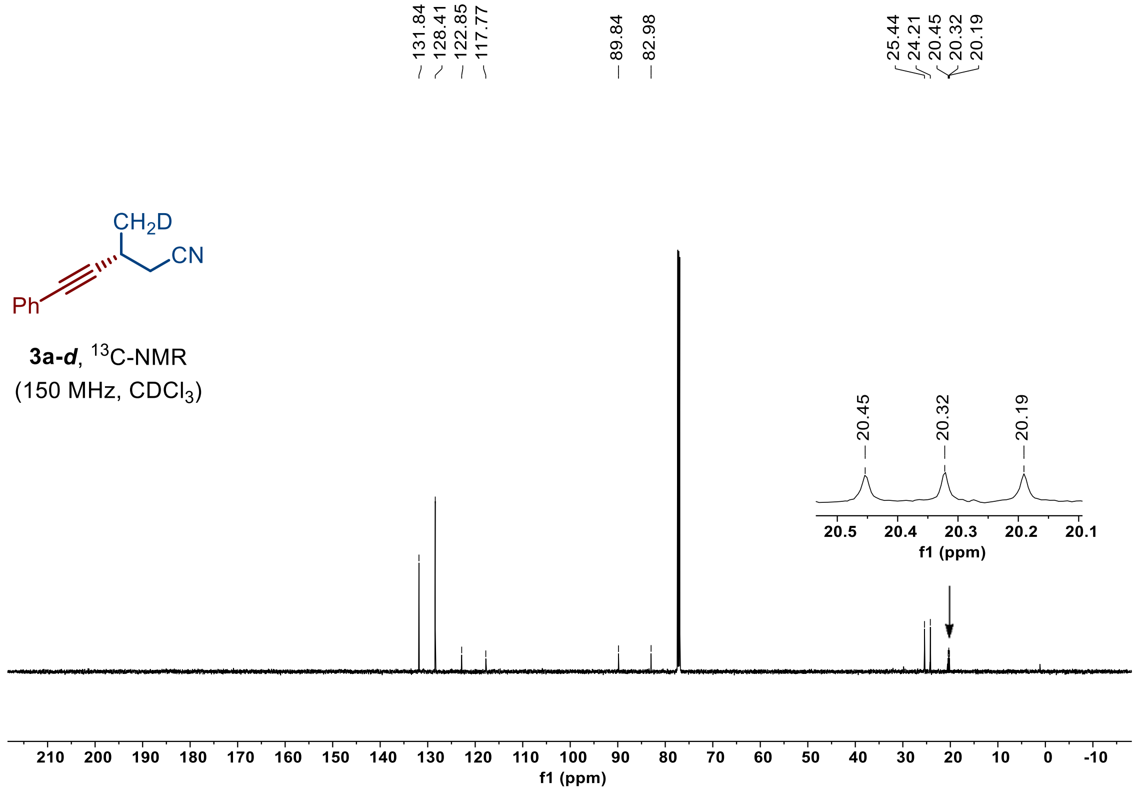

^{1}H NMR (400 MHz, $CDCl_3$) spectrum of 9a

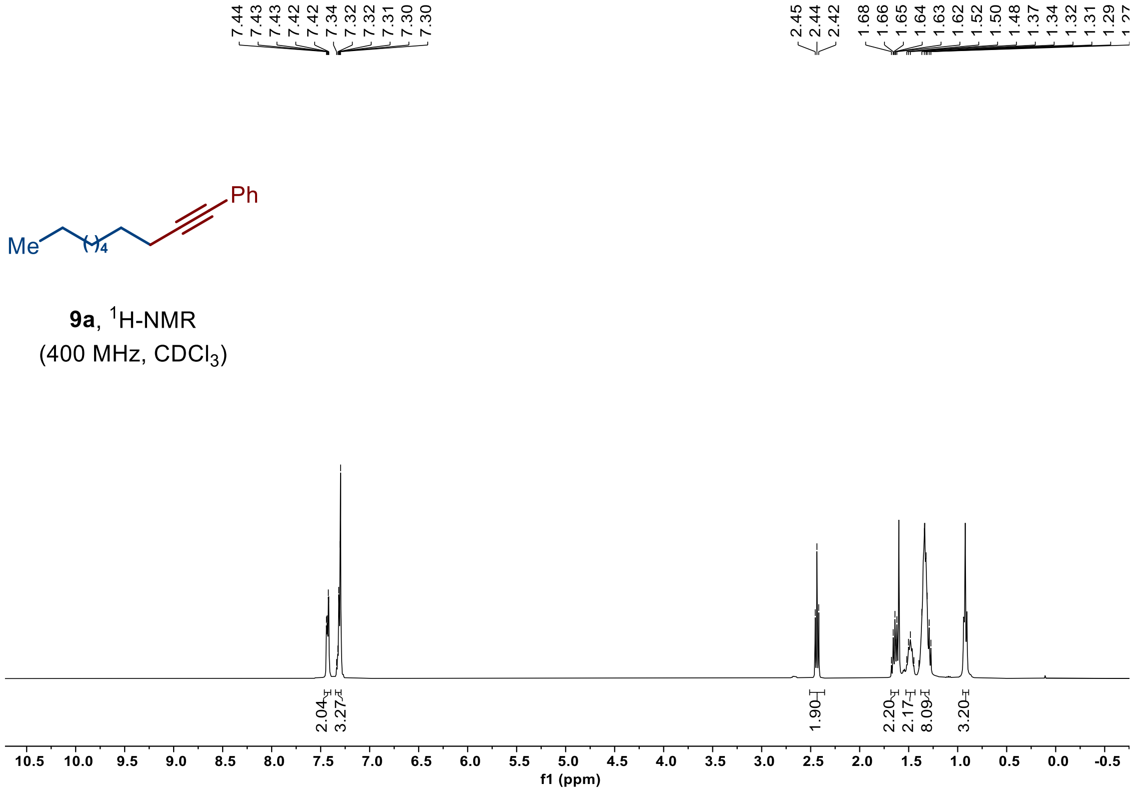

^{13}C NMR (100 MHz, $CDCl_3$) spectrum of 9a

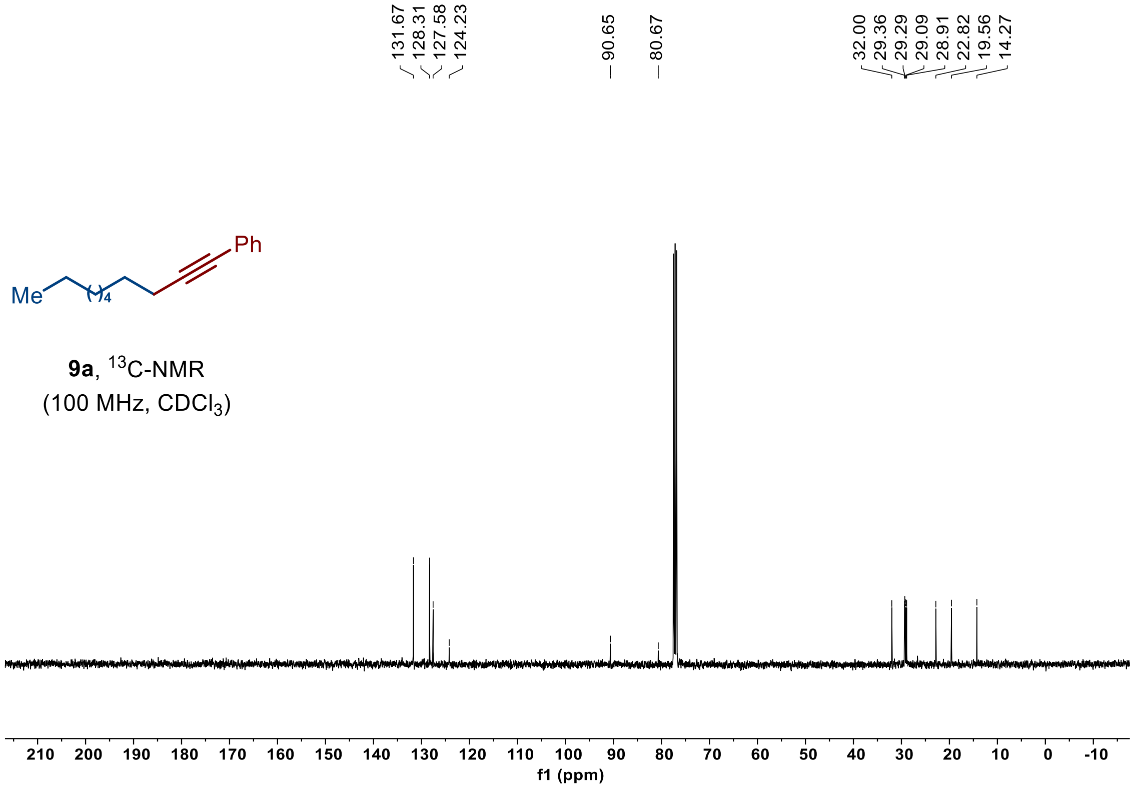

REFERENCES AND NOTES

1. R. K. Dhungana, S. Kc, P. Basnet, R. Giri, Transition metal-catalyzed dicarbofunctionalization of unactivated olefins. *Chem. Rec.* **18**, 1314–1340 (2018).

2. J. Derosa, O. Apolinar, T. Kang, V. T. Tran, K. M. Engle, Recent developments in nickel-catalyzed intermolecular dicarbofunctionalization of alkenes. *Chem. Sci.* **11**, 4287–4296 (2020).

3. Z.-L. Li, G.-C. Fang, Q.-S. Gu, X.-Y. Liu, Recent advances in copper-catalysed radical-involved asymmetric 1,2-difunctionalization of alkenes. *Chem. Soc. Rev.* **49**, 32–48 (2020).

4. X. Qi, T. Diao, Nickel-catalyzed dicarbofunctionalization of alkenes. *ACS Catal.* **10**, 8542–8556 (2020).

5. S. O. Badir, G. A. Molander, Developments in photoredox/nickel dual-catalyzed 1,2-difunctionalizations. *Chem* **6**, 1327–1339 (2020).

6. S.-Q. Zhu, X. Zhao, H. Li, L. Chu, Catalytic three-component dicarbofunctionalization reactions involving radical capture by nickel. *Chem. Soc. Rev.* **50**, 10836–10856 (2021).

7. J. Han, R. He, C. Wang, Transition metal-catalyzed asymmetric three-component dicarbofunctionalization of unactivated alkenes. *Chem Catal.* **3**, 100690 (2023).

8. J. A. Gurak Jr., K. S. Yang, Z. Liu, K. M. Engle, Regiocontrolled hydroamination of unactivated alkenes via protodepalladation. *J. Am. Chem. Soc.* **138**, 5805–5808 (2016).

9. P. Basnet, S. K. C. R. K. Dhungana, B. Shrestha, T. J. Boyle, R. Giri, Synergistic bimetallic Ni/Ag and Ni/Cu catalysis for regioselective γ,δ-diarylation of alkenyl ketimines: Addressing β-H elimination by in situ generation of cationic Ni(II) catalysts. *J. Am. Chem. Soc.* **140**, 15586–15590 (2018).

10. T. Yang, X. Chen, W. Rao, M. J. Koh, Broadly applicable directed catalytic reductive difunctionalization of alkenyl carbonyl compounds. *Chem* **6**, 738–751 (2020).

11. L. Xie, S. Wang, L. Zhang, L. Zhao, C. Luo, L. Mu, X. Wang, C. Wang, Directed nickel-catalyzed region- and diastereoselective arylamination of unactivated alkenes. *Nat. Commun.* **12**, 6280 (2021).

12. J. Wang, Z. Duan, X. Liu, S. Dong, K. Chen, J. Li, Salt-stabilized silylzinc pivalates for nickel-catalyzed carbosilylation of alkenes. *Angew. Chem. Int. Ed. Engl.* **61**, e202202379 (2022).

13. L. Zhao, Y. Zhu, M. Liu, L. Xie, J. Liang, H. Shi, X. Meng, Z. Chen, J. Han, C. Wang, Ligand-controlled NiH-catalyzed regiodivergent chain-walking hydroalkylation of alkenes. *Angew. Chem. Int. Ed. Engl.* **61**, e202204716 (2022).

14. J.-W. Wang, D.-G. Liu, Z. Chang, Z. Li, Y. Fu, X. Lu, Nickel-catalyzed switchable site-selective alkene hydroalkylation by temperature regulation. *Angew. Chem. Int. Ed. Engl.* **61**, e202205537 (2022).

15. P.-F. Yang, W. Shu, Orthogonal access to α-/β-branched/linear aliphatic amines by catalyst-tuned regiodivergent hydroalkylations. *Angew. Chem. Int. Ed. Engl.* **61**, e202208018 (2022).

16. C. Lee, H.-J. Kang, H. Seo, S. Hong, Nickel-catalyzed regio- and enantioselective hydroamination of unactivated alkenes using carbonyl directing groups. *J. Am. Chem. Soc.* **144**, 9091–9100 (2022).

17. J. Huang, X. Yan, X. Liu, Z. Chen, T. Jiang, L. Zhang, G. Ju, G. Huang, C. Wang, Enantioselective Ni-catalyzed 1,2-borylalkynylation of unactivated alkenes. *J. Am. Chem. Soc.* **146**, 17140–17149 (2024).

18. B. Cheng, P. Lu, H. Zhang, X. Cheng, Z. Lu, Highly enantioselective cobalt-catalyzed hydrosilylation of alkenes. *J. Am. Chem. Soc.* **139**, 9439–9442 (2017).

19. C.-L. Liu, X. Luo, H. Wang, M. J. Koh, Catalytic regioselective olefin hydroarylation (alkenylation) by sequential carbon–nickelation–hydride transfer. *J. Am. Chem. Soc.* **143**, 9498–9506 (2021).

20. H. Wang, C.-F. Liu, R. T. Martin, O. Gutierrez, M. J. Koh, Directing-group-free catalytic dicarbofunctionalization of unactivated alkenes. *Nat. Chem.* **14**, 188–195 (2022).

21. Z.-C. Wang, X. Luo, J.-W. Zhang, C.-F. Liu, M. J. Koh, S.-L. Shi, Enantioselective C–C cross-coupling of unactivated alkenes. *Nat. Catal.* **6**, 1087–1097 (2023).

22. B. C. Lee, C.-F. Liu, L. Q. H. Lin, K. Z. Yap, N. Song, C. H. M. Ko, P. H. Chan, M. J. Koh, N-heterocyclic carbenes as privileged ligands for nickel-catalyzed alkene functionalization. *Chem. Soc. Rev.* **52**, 2946–2991 (2023).

23. J. P. Wagner, P. R. Schreiner, London dispersion in molecular chemistry—Reconsidering steric effects. *Angew. Chem. Int. Ed. Engl.* **54**, 12274–12296 (2015).

24. S. E. Wheeler, T. J. Seguin, Y. Guan, A. C. Doney, Noncovalent interactions in organocatalysis and the prospect of computational catalyst design. *Acc. Chem. Res.* **49**, 1061–1069 (2016).

25. F. D. Toste, M. S. Sigman, S. J. Miller, Pursuit of noncovalent interactions for strategic site-selective catalysis. *Acc. Chem. Res.* **50**, 609–615 (2017).

26. A. J. Neel, M. J. Hilton, M. S. Sigman, F. D. Toste, Exploiting noncovalent π interactions for catalyst design. *Nature* **543**, 637–646 (2017).

27. H. J. Davis, R. J. Phipps, Harnessing non-covalent interactions to exert control over regioselectivity and site-selectivity in catalytic reactions. *Chem. Sci.* **8**, 864–877 (2017).

28. Y. Kuninobu, H. Ida, M. Nishi, M. Kanai, A meta-selective C–H borylation directed by a secondary interaction between ligand and substrate. *Nat. Chem.* **7**, 712–717 (2015).

29. Y. Saito, Y. Segawa, K. Itami, para-C–H borylation of benzene derivatives by a bulky iridium catalyst. *J. Am. Chem. Soc.* **137**, 5193–5198 (2015).

30. H. J. Davis, M. T. Mihai, R. J. Phips, Ion pair-directed regiocontrol in transition-metal catalysis: A meta-selective C–H borylation of aromatic quaternary ammonium salts. *J. Am. Chem. Soc.* **138**, 12759–12762 (2016).

31. R. Bisht, M. E. Hoque, B. Chattopadh-yay, Amide effects in C−H activation: Noncovalent interactions with L-shaped ligand for meta borylation of aromatic amides. *Angew. Chem. Int. Ed. Engl.* **57**, 15762–15766 (2018).

32. L. Yang, N. Uemura, Y. Nakao, meta-Selective C–H borylation of benzamides and pyridines by an iridium–Lewis acid bifunctional catalyst. *J. Am. Chem. Soc.* **141**, 7972–7979 (2019).

33. M. T. Mihai, B. D. Williams, R. J. Phipps, Para-selective C–H borylation of common arene building blocks enabled by ion-pairing with a bulky countercation. *J. Am. Chem. Soc.* **141**, 15477–15482 (2019).

34. G. R. Genov, J. L. Douthwaite, A. S. K. Lahdenperä, D. C. Gibson, R. J. Phipps, Enantioselective remote C–H activation directed by a chiral cation. *Science* **367**, 1246–1251 (2020).

35. J. Chaturvedi, C. Haldar, R. Bisht, G. Pandey, Meta selective C–H borylation of sterically biased and unbiased substrates directed by electrostatic interaction. *J. Am. Chem. Soc.* **143**, 7604–7611 (2021).

36. B. Ramadoss, Y. Jin, S. Asako, L. Ilies, Remote steric control for undirected meta-selective C–H activation of arenes. *Science* **375**, 658–663 (2022).

37. B. W. Li, J. Z. Chen, D. Liu, I. D. Gridnev, W. B. Zhang, Nickel-catalysed asymmetric hydrogenation of oximes. *Nat. Chem.* **14**, 920–927 (2022).

38. F. Wang, Y. Chen, P. Yu, G.-Q. Chen, X. Zhang, Asymmetric hydrogenation of oximes synergistically assisted by Lewis and Brønsted acids. *J. Am. Chem. Soc.* **144**, 17763–17768 (2022).

39. W. Zhao, W. Wang, H. Zhou, Q. Liu, Z. Ma, H. Huang, M. Chang, An Asymmetric hydrogenation/N-alkylation sequence for a step-economical route to indolizidines and quinolizidines. *Angew. Chem. Int. Ed. Engl.* **62**, e202308836 (2023).

40. B. G. Hejna, J. M. Ganley, H. Shao, H. Tian, J. D. Ellefsen, N. J. Fastuca, K. N. Houk, S. J. Miller, R. R. Knowles, Catalytic asymmetric hydrogen atom transfer: Enantioselective hydroamination of alkenes. *J. Am. Chem. Soc.* **145**, 16118–16129 (2023).

41. J. Guan, Y. Luo, Q. Wang, J. Chen, W. Zhang, Copper-catalyzed asymmetric hydrogenation of unsymmetrical ortho-Br substituted benzophenones. *Angew. Chem. Int. Ed. Engl.* **64**, e202416313 (2024).

42. M. Wang, S. Liu, H. Liu, Y. Wang, Y. Lan, Q. Liu, Asymmetric hydrogenation of ketimines with minimally different alkyl groups. *Nature* **631**, 556–562 (2024).

43. G. Lu, R. Y. Liu, Y. Yang, C. Fang, D. S. Lambrecht, S. L. Buchwald, P. Liu, Ligand−substrate dispersion facilitates the copper-catalyzed hydroamination of unactivated olefins. *J. Am. Chem. Soc.* **139**, 16548–16555 (2017).

44. Y.-H. Liu, P.-P. Xie, L. Liu, J. Fan, Z.-Z. Zhang, X. Hong, B.-F. Shi, Cp*Co(III)-catalyzed enantioselective hydroarylation of unactivated terminal alkenes via C–H activation. *J. Am. Chem. Soc.* **143**, 19112–19120 (2021).

45. L. Ge, H. Zhou, M.-F. Chiou, H. M. Jiang, W. J. Jian, C. Q. Ye, X. Y. Li, X. T. Zhu, H. G. Xiong, Y. J. Li, L. J. Song, X. H. Zhang, H. L. Bao, Iron-catalysed asymmetric carboazidation of styrenes. *Nat. Catal.* **4**, 28–35 (2021).

46. Y. Li, W. Nie, Z. Chang, J.-W. Wang, X. Lu, Y. Fu, Cobalt-catalysed enantioselective C(sp3)–C(sp3) coupling. *Nat. Catal.* **4**, 901–911 (2021).

47. C. Fan, U. Dhawa, D. Qian, D. Sakic, J. Morel, X. Hu, Regiodivergent and enantioselective synthesis of cyclic sulfones via ligand-controlled nickel-catalyzed hydroalkylation. *Angew. Chem. Int. Ed. Engl.* **63**, e202406767 (2024).

48. X. Wang, J. Xue, Z.-Q. Rong, Divergent access to chiral C2- and C3-alkylated pyrrolidines by catalyst-tuned regio- and enantioselective $C(sp^3)$–$C(sp^3)$ coupling. *J. Am. Chem. Soc.* **145**, 15456–15464 (2023).

49. L. Xie, J. Liang, H. Bai, X. Liu, X. Meng, Y.-Q. Xu, Z.-Y. Cao, C. Wang, Ligand-controlled NiH-catalyzed regiodivergent and enantioselective hydroamination of alkenyl amides. *ACS Catal.* **13**, 10041–10047 (2023).

50. F. F. Fleming, Q. Wang, Unsaturated nitriles: Conjugate additions of carbon nucleophiles to a recalcitrant class of acceptors. *Chem. Rev.* **103**, 2035–2078 (2003).

51. W.-B. Wu, J.-S. Yu, J. Zhou, Catalytic enantioselective cyanation: Recent advances and perspectives. *ACS Catal.* **10**, 7668–7690 (2020).

52. S. Pimparkar, A. Koodan, S. Maiti, N. S. Ahmed, M. M. M. Mostafa, D. Maiti, C–CN bond formation: An overview of diverse strategies. *Chem. Commun.* **57**, 2210–2232 (2021).

53. Y. Nakao, Metal-mediated C–CN bond activation in organic synthesis. *Chem. Rev.* **121**, 327–344 (2021).

54. M. Kondo, T. Nishi, T. Hat-anaka, Y. Funahashi, S. Nakamura, Catalytic enantioselective reaction of α-aminoacetonitriles using chiral bis(imidazoline) palladium catalysts. *Angew. Chem. Int. Ed. Engl.* **54**, 8198–8202 (2025).

55. G. Zhang, S. Zhou, L. Fu, P. Chen, Y. Li, J. Zou, G. Liu, Asymmetric coupling of carbon-centered radicals adjacent to nitrogen: Copper-catalyzed cyanation and etherification of enamides. *Angew. Chem. Int. Ed. Engl.* **59**, 20439–20444 (2020).

56. R. Yu, S. Rajasekar, X. Fang, Enantioselective nickel-catalyzed migratory hydrocyanation of nonconjugated dienes. *Angew. Chem. Int. Ed. Engl.* **59**, 21436–21441 (2020).

57. X.-L. Lai, M. Chen, Y. Wang, J. Song, H.-C. Xu, Photoelectrochemical asymmetric catalysis enables direct and enantioselective decarboxylative cyanation. *J. Am. Chem. Soc.* **144**, 20201–20206 (2022).

58. H. Lee, H. Nam, S. Y. Lee, Enantio- and diastereoselective variations on α-iminonitriles: Harnessing chiral cyclopropenimine-thiourea organocatalysts. *J. Am. Chem. Soc.* **146**, 3065–3074 (2024).

59. B. Zhang, T.-T. Li, Z.-C. Mao, M. Jiang, Z. Zhang, K. Zhao, W.-Y. Qu, W.-J. Xiao, J.-R. Chen, Enantioselective cyanofunctionalization of aromatic alkenes via radical anions. *J. Am. Chem. Soc.* **146**, 1410–1422 (2024).

60. M. Jiao, J. Long, J. Chen, H. Yang, T. Wang, X. Fang, Nickel-catalyzed regio- and enantioselective migratory hydrocyanation of internal alkenes: Expanding the scope to α,ω-diaryl internal alkenes. *Angew. Chem. Int. Ed. Engl.* **63**, e202402390 (2024).

61. A. Vasseur, J. Bruffaerts, I. Marek, Remote functionalization through alkene isomerization. *Nat. Chem.* **8**, 209–219 (2016).

62. H. Sommer, F. Juliá-Hernández, R. Martin, I. Marek, Walking metals for remote functionalization. *ACS Cent. Sci.* **4**, 153–165 (2018).

63. D. Janssen-Müller, B. Sahoo, S.-Z. Sun, R. Martin, Tackling remote sp^3 C−H functionalization via Ni-catalyzed "chain-walking" reactions. *Isr. J. Chem.* **60**, 195–206 (2020).

64. Y. Li, D. Wu, H.-G. Cheng, G. Yin, Difunctionalization of alkenes involving metal migration. *Angew. Chem. Int. Ed. Engl.* **59**, 7990–8003 (2020).

65. Y. Wang, Y. He, S. Zhu, NiH-catalyzed functionalization of remote and proximal olefins: New reactions and innovative strategies. *Acc. Chem. Res.* **55**, 3519–3536 (2022).

66. F. F. Fleming, L. Yao, P. C. Ravikumar, L. Funk, B. C. Shook, Nitrile-containing pharmaceuticals: Efficacious roles and mechanisms. *J. Med. Chem.* **53**, 7902–7917 (2010).

67. V. Bonatto, R. F. Lameiro, F. R. Rocho, J. Lameira, A. Leitão, C. A. Montanari, Nitriles: An attractive approach to the development of small-molecule drugs. *RSC Med. Chem.* **14**, 201–217 (2023).

68. A. Bauzá, D. Quiñonero, A. Frontera, P. Ballester, Reconciling experiment and theory in the use of aryl-extended calix[4]pyrrole receptors for the experimental quantification of chloride–π interactions in solution. *Int. J. Mol. Sci.* **16**, 8934–8948 (2015).

69. M. Y. Jin, Q. Zhen, D. Xiao, G. Tao, X. Xing, P. Yu, C. Xu, Engineered non-covalent π interactions as key elements for chiral recognition. *Nat. Commun.* **13**, 3276 (2022).

70. T. Nishimura, X.-X. Guo, N, Uchiyama, T. Katoh, T. Hayashi, Steric tuning of silylacetylenes and chiral phosphine ligands for rhodium-catalyzed asymmetric conjugate alkynylation of enones. *J. Am. Chem. Soc.* **130**, 1576–1577 (2008).

71. X. Dou, Y. Huang, T. Hayashi, Asymmetric conjugate alkynylation of cyclic α,β-unsaturated carbonyl compounds with a chiral diene rhodium catalyst. *Angew. Chem. Int. Ed. Engl.* **55**, 1133–1137 (2016).

72. A. K. Simlandy, T. M. Alturaifi, J. M. Nguyen, L. J. Oxtoby, Q. N. Wong, J. S. Chen, P. Liu, K. M. Engle, Enantioselective hydroalkenylation and hydroalkynylation of alkenes enabled by a transient directing group: Catalyst generality through rigidification. *Angew. Chem. Int. Ed. Engl.* **62**, e202304013 (2023).

73. T. F. Knöpfel, E. M. Carreira, The first conjugate addition reaction of terminal alkynes catalytic in copper: Conjugate addition of alkynes in water. *J. Am. Chem. Soc.* **125**, 6054–6055 (2003).

74. T. F. Knöpfel, P. Zarotti, T. Ichikawa, E. M. Carreira, Catalytic, enantioselective, conjugate alkyne addition. *J. Am. Chem. Soc.* **127**, 9682–9683 (2005).

75. E. Fillion, A. K. Zorzitto, Enantioselective rhodium-catalyzed conjugate alkynylation of 5-benzylidene Meldrum's acids with TMS-acetylene. *J. Am. Chem. Soc.* **131**, 14608–14609 (2009).

76. T. Sawano, A. Ashouri, T. Nishimura, T. Hayashi, Cobalt-catalyzed asymmetric 1,6-addition of (triisopropylsilyl)-acetylene to α,β,γ,δ-unsaturated carbonyl compounds. *J. Am. Chem. Soc.* **134**, 18936–18939 (2012).

77. R. Yazaki, N. Kumagai, M. Shibasaki, Direct catalytic asymmetric conjugate addition of terminal alkynes to α,β-unsaturated thioamides. *J. Am. Chem. Soc.* **132**, 10275–10277 (2010).

78. T. Nishimura, T. Sawano, T. Hayashi, Asymmetric synthesis of β-alkynyl aldehydes by rhodium-catalyzed conjugate alkynylation. *Angew. Chem. Int. Ed. Engl.* **48**, 8057–8059 (2009).

79. M. Shirakura, M. Suginome, Nickel-catalyzed asymmetric addition of alkyne C−H bonds across 1,3-dienes using Taddol-based chiral phosphoramidite ligands. *Angew. Chem. Int. Ed. Engl.* **49**, 3827–3829 (2010).

80. M. Shirakura, M. Suginome, Nickel-catalyzed, regio- and stereoselective hydroalkynylation of methylenecyclopropanes with retention of the cyclopropane ring, leading to the synthesis of 1-methyl-1-alkynylcyclopropanes. *J. Am. Chem. Soc.* **131**, 5060–5061 (2009).

81. X.-Y. Bai, Z.-X. Wang, B.-J. Li, Iridium-catalyzed enantioselective hydroalkynylation of enamides for the synthesis of homopropargyl amides. *Angew. Chem. Int. Ed. Engl.* **55**, 9007–9011 (2016).

82. X.-Y. Bai, W.-W. Zhang, Q. Li, B.-J. Li, Highly enantioselective synthesis of propargyl amides through Rh-catalyzed asymmetric hydroalkynylation of enamides: Scope, mechanism, and origin of selectivity. *J. Am. Chem. Soc.* **140**, 506–514 (2018).

83. W.-W. Zhang, S.-L. Zhang, B.-J. Li, Highly enantioselective synthesis of propargyl amide with vicinal stereocenters through Ir-catalyzed hydroalkynylation. *Angew. Chem. Int. Ed. Engl.* **59**, 6874–6880 (2020).

84. S.-L. Zhang, W.-W. Zhang, B.-J. Li, Ir-catalyzed regio- and enantioselective hydroalkynylation of trisubstituted alkene to access all-carbon quaternary stereocenters. *J. Am. Chem. Soc.* **143**, 9639–9647 (2021).

85. M. Liu, H. Shi, X. Meng, R. He, J. Huang, J. Wang, J. Lv, C. Wang, Asymmetric NiH-catalyzed regioselective hydroalkynylation of unactivated alkenes. *CCS. Chem.* **6**, 1929–1940 (2024).

86. X. Jiang, B. Han, Y. Xue, M. Duan, Z. Gui, Y. Wang, S. Zhu, Nickel-catalysed migratory hydroalkynylation and enantioselective hydroalkynylation of olefins with bromoalkynes. *Nat. Commun.* **12**, 3792 (2021).

87. Y. Zhang, J. W. Ma, J. Chen, L. P. Meng, Y. Liang, S. Zhu, A relay catalysis strategy for enantioselective nickel-catalyzed migratory hydroarylation forming chiral α-aryl alkylboronates. *Chem* **7**, 3171–3188 (2021).

88. Y. Zhu, Z. Feng, W. Li, J. Wang, G. Ju, C. Wang, Coordination-assisted Ni-catalyzed regio- and enantioselective 1,2-borylalkylation of unactivated alkenes. *Org. Lett.* **26**, 10622–10627 (2024).

89. D. Qian, S. Bera, X. Hu, Chiral alkyl amine synthesis via catalytic enantioselective hydroalkylation of enecarbamates. *J. Am. Chem. Soc.* **143**, 1959–1967 (2021).

90. Z. W. Lu, X.-D. Hu, H. Zhang, X.-W. Zhang, J. H. Cai, M. Usman, H. J. Cong, W.-B. Liu, Enantioselective assembly of cycloenones with a nitrile-containing all-carbon quaternary center from malononitriles enabled by Ni catalysis. *J. Am. Chem. Soc.* **142**, 7328–7333 (2020).

91. H. Choi, X. Lyu, D. Kim, S. Seo, S. Chang, Endo-selective intramolecular alkyne hydroamidation enabled by NiH catalysis incorporating alkenylnickel isomerization. *J. Am. Chem. Soc.* **144**, 10064–10074 (2022).

92. M. K. Ghorai, R. Talukdar, D. P. Tiwari, A route to highly functionalized β-enaminoesters via a domino ring-opening cyclization/decarboxylative tautomerization sequence of donor–acceptor cyclopropanes with substituted malononitriles. *Org. Lett.* **16**, 2204–2207 (2014).

93. X. Li, S. Wang, X. Fu, D. Xing, Z. Fu, Y. Deng, H. Jiang, L. Huang, Nickel-catalyzed arylcyanation of alkenes via cyano group translocation: Access to 1,n-dinitriles or 4-amino nitriles. *Sci. China Chem.* **67**, 2975–2981 (2024).

94. C. Chang, H. Zhang, X. Wu, C. Zhu, Radical trifunctionalization of hexenenitrile via remote cyano migration†. *Chem. Commun.* **58**, 1005–1008 (2022).

95. L. Zhao, X. Meng, Y. Zou, J. Zhao, L. Wang, L. Zhang, C. Wang, Directed nickel-catalyzed diastereoselective reductive difunctionalization of alkenyl amines. *Org. Lett.* **23**, 8516–8521 (2021).

96. FM. J. Frisch, G. W. Trucks, J. Schlegel, G. E. Scuseria, M. A. Robb, J. R. Cheeseman, H. B. Schlegel, G. Scalmani, V. Barone, B. Mennucci, G. A. Petersson, Gaussian 09, Revision E.01 (Gaussian Inc., 2013).

97. C. Lee, W. Yang, R. G. Parr, Development of the Colle-Salvetti correlation-energy formula into a functional of the electron density. *Phys. Rev. B.* **37**, 785–789 (1988).

98. A. D. Becke, Density-functional thermochemistry. III. The role of exact exchange. *J. Chem. Phys.* **98**, 5648–5652 (1993).

99. S. Grimme, J. Antony, S. Ehrlich, H. Krieg, A consistent and accurate ab initio parametrization of density functional dispersion correction (DFT-D) for the 94 elements H-Pu. *J. Chem. Phys.* **132**, 154104 (2010).

100. K. Fukui, Formulation of the reaction coordinate. *J. Phys. Chem.* **74**, 4161–4163 (1970).

101. K. Fukui, The path of chemical reactions—The IRC approach. *Acc. Chem. Res.* **14**, 363–368 (1981).

102. P. Pracht, F. Bohle, S. Grimme, Automated exploration of the low-energy chemical space with fast quantum chemical methods. *Phys. Chem. Chem. Phys.* **22**, 7169–7192 (2020).

103. S. Dohm, A. Hansen, M. Steinmetz, S. Grimme, M. P. Checinski, Comprehensive thermochemical benchmark set of realistic closed-shell metal organic reactions. *J. Chem. Theory Comput.* **14**, 2596–2608 (2018).

104. A. V. Marenich, C. J. Cramer, D. G. Truhlar, Universal solvation model based on solute electron density and on a continuum model of the solvent defined by the bulk dielectric constant and atomic surface tensions. *J. Phys. Chem. B* **113**, 6378–6396 (2009).

105. C. Y. Legault, *CYLView*, 1.0b; Université de Sherbrooke, 2009; http://cylview.org.

106. M. A. Spackman, P. G. Byrom, A novel definition of a molecule in a crystal. *Chem. Phys. Lett.* **267**, 215–220 (1997).

107. M. A. Spackman, D. Jayatilaka, Hirshfeld surface analysis. *CrstEngComm* **11**, 19–32 (2009).

108. J. J. McKinnon, M. A. Spackman, A. S. Mitchell, Novel tools for visualizing and exploring intermolecular interactions in molecular crystals. *Acta Cryst. B* **60**, 627–668 (2004).

109. C. Bissantz, B. Kuhn, M. Stahl, A medicinal chemist's guide to molecular interactions. *J. Med. Chem.* **53**, 5061–5084 (2010).

110. A. J. Neel, M. J. Hilton, M. S. Sigman, F. D. Toste, Exploiting non-covalent π interactions for catalyst design. *Nature* **543**, 637–646 (2017).

111. T. Lu, Q. Chen, Interaction region indicator: A simple real space function clearly revealing both chemical bonds and weak interactions. *Chem. Methods* **1**, 231–239 (2021).

112. T. Lu, F. Chen, Multiwfn: A multifunctional wavefunction analyzer. *J. Comput. Chem.* **33**, 580–592 (2012).

113. T. Lu, A comprehensive electron wavefunction analysis toolbox for chemists, Multiwfn. *J. Chem. Phys.* **161**, 082503 (2024).

114. W. Humphrey, A. Dalke, K. Schulten, VMD: Visual molecular dynamics. *J. Mol. Graph.* **14**, 33–38 (1996).
